# Carbon Nanodots from an In Silico Perspective

**DOI:** 10.1021/acs.chemrev.1c00864

**Published:** 2022-08-10

**Authors:** Francesca Mocci, Leon de Villiers Engelbrecht, Chiara Olla, Antonio Cappai, Maria Francesca Casula, Claudio Melis, Luigi Stagi, Aatto Laaksonen, Carlo Maria Carbonaro

**Affiliations:** † Department of Chemical and Geological Sciences, University of Cagliari, I-09042 Monserrato, Italy; ‡ Department of Physics, University of Cagliari, I-09042 Monserrato, Italy; § Department of Mechanical, Chemical and Materials Engineering, University of Cagliari, Via Marengo 2, IT 09123 Cagliari, Italy; ∥ Department of Chemistry and Pharmacy, Laboratory of Materials Science and Nanotechnology, University of Sassari, Via Vienna 2, 07100 Sassari, Italy; ⊥ Department of Materials and Environmental Chemistry, Arrhenius Laboratory, Stockholm University, SE-106 91 Stockholm, Sweden; + State Key Laboratory of Materials-Oriented and Chemical Engineering, Nanjing Tech University, Nanjing 210009, P. R. China; × Centre of Advanced Research in Bionanoconjugates and Biopolymers, PetruPoni Institute of Macromolecular Chemistry, Aleea Grigore Ghica-Voda 41A, 700487 Iasi, Romania; △ Division of Energy Science, Energy Engineering, Luleå University of Technology, Luleå 97187, Sweden

## Abstract

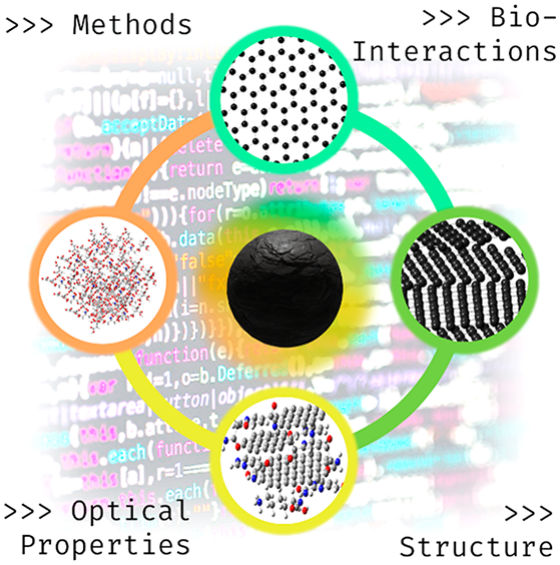

Carbon nanodots (CNDs) are the latest and most shining rising stars among photoluminescent (PL) nanomaterials. These carbon-based surface-passivated nanostructures compete with other related PL materials, including traditional semiconductor quantum dots and organic dyes, with a long list of benefits and emerging applications. Advantages of CNDs include tunable inherent optical properties and high photostability, rich possibilities for surface functionalization and doping, dispersibility, low toxicity, and viable synthesis (top-down and bottom-up) from organic materials. CNDs can be applied to biomedicine including imaging and sensing, drug-delivery, photodynamic therapy, photocatalysis but also to energy harvesting in solar cells and as LEDs. More applications are reported continuously, making this already a research field of its own. Understanding of the properties of CNDs requires one to go to the levels of electrons, atoms, molecules, and nanostructures at different scales using modern molecular modeling and to correlate it tightly with experiments. This review highlights different in silico techniques and studies, from quantum chemistry to the mesoscale, with particular reference to carbon nanodots, carbonaceous nanoparticles whose structural and photophysical properties are not fully elucidated. The role of experimental investigation is also presented. Hereby, we hope to encourage the reader to investigate CNDs and to apply virtual chemistry to obtain further insights needed to customize these amazing systems for novel prospective applications.

## 1 Introduction

Nanosized systems of low dimensions have inspired the new research area known as “nanotechnology” since the mid-70s, when the very term was coined by Norio Taniguchi on a conference of production engineering in Tokyo.^1^ Tiny semiconductor nanostructures could be produced in laboratories and were called “quantum wells”, “quantum wires”, or “quantum dots”. In quantum dots (QD) the electron gas is restricted in a semiconductor heterostructure by electrostatic gates creating a bowl-like potential trapping the conduction electrons making it an artificial atom structure. Their quantum properties could be modified and controlled by moving the gates, modifying the atomistic microstructure of the dot either geometrically or by doping with impurities, this way shifting the band gap, or by applying fields.^2^

Several types of nanodots have been introduced since the pioneering days, all having unique optical, chemical, and electronic properties. Today, carbon dots (CDs), which are carbon-based nanoparticles with a remarkable fluorescence, represent an intensively studied family of nanodots. The term is derived from its semiconductor-based counterpart (QD),^3^ whose optical properties primarily depend on size and shape.^4^

Since their serendipitous discovery in 2004 by Xu and colleagues during the purification of single-walled carbon nanotubes,^5^ CDs have received considerable attention because of their extraordinary properties and ease of syntheses.

Within the CD family, carbon atoms can arrange into one or more of the allotropes forms of carbon, from highly ordered graphene quantum dots (GQDs)^6,7^ to disordered carbonized polymer dots (CPDs).^8,9^ Carbon nanodots (CNDs) can be regarded somehow as intermediate between GQDs and CPDs in that CNDs share the characteristics both of the ordered quantum system of a few layers of sp^2^ graphene and of the disordered and cross-linked carbon chain of the conjugated polymer.^10−12^ Although there is not a clear-cut separation between different classes of dots, the shape of GQDs and CNDs differs in that the height of the former is smaller than the lateral size, while the latter can be nearly spherical.^13^

**Figure 1:**
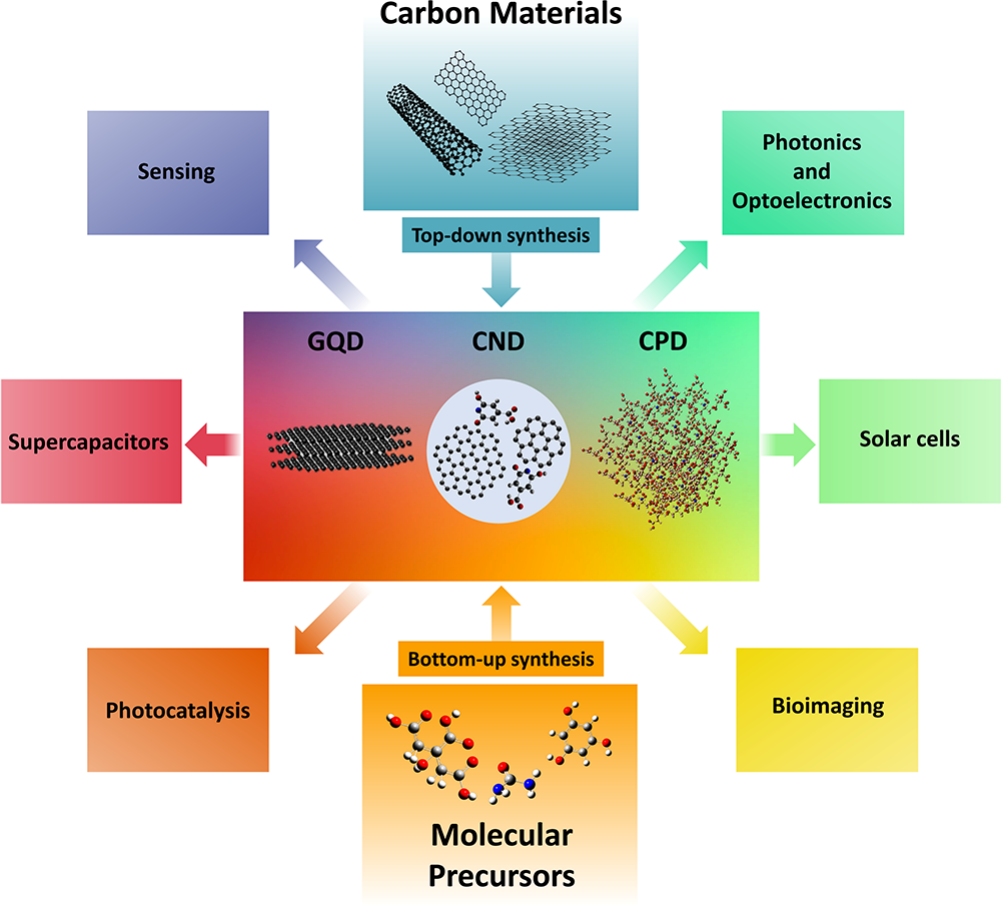
Synthetic approaches for the different forms of carbon dots (CND, carbon nanodot; GQD, graphene quantum dot; CPD, carbonized polymer dot) and main areas of application.

CNDs are carbon-based nanoparticles (NPs) or 0D nanostructures with all three dimensions <10 nm, and they are frequently described by the “core-shell” model, in which an sp^2^ ordered core structure is surrounded by a disordered sp^3^ shell. Disordered CND core structures with various sp^2^/sp^3^ ratios have also been reported, with the disorder degree being dependent on synthesis and environmental conditions, resulting in surface oxidation or elemental doping.^9^ The latter has been largely applied to boost and tune the optical features of CNDs, which are characterized by an efficient visible emission, typically ranging from the blue-green range of the visible spectrum with the peculiar feature that the observed emission is, in general, excitation dependent: the emission peak red-shifts as the excitation wavelength increases. These features, shared with GQDs and CPDs, although ascribed to different emission mechanisms,^13^ compete with those of inorganic quantum dots and organic dyes but are connected with unmatched low toxicity, chemical inertness, high compatibility with the biological environment, large photostability, and water solubility/dispersibility. These properties make CNDs excellent candidates as suitable materials for luminescence based technological applications, such as display LEDs,^14,15^ drug delivery,^16^ for sensors,^17^ detectors,^18^ photocatalysis,^19^ biosensing, cell labeling, imaging,^20^ and thermoelectrics^21^ (Figure 1).

In this review the main focus is on CNDs, probably the largest group of the CDs family. Due to the wide variety of CNDs, accounting for their chemical versatility and synthesis approaches, it can be difficult to find the correlation between morphology and properties. In particular, understanding the optical features of CNDs, how they are affected by the structural organization, elemental doping, system size, and other factors, is a fundamental step to rationally optimize their design and technological applications. On the other hand, the applications of CNDs as sensors and biosensors poses the question on how CNDs interact with other molecules and particularly with biomolecules. Obtaining the answers to these questions is generally hampered by the difficulties due to the complexity of CND structures. Indeed, the ordered (GQD) and disordered (CPD) systems represent a practical reference for the description of CNDs and will therefore also be considered in this review. In particular, the structural features are often discussed by pointing out similarities and differences with respect to the corresponding graphene structures.

Computational chemistry methods, i.e., electronic structure calculations, molecular quantum mechanics (QM) first-principles simulations, classical molecular mechanical force-field (FF) based simulations, coarse-grained simulations, to mention a few, are highly valuable tools in a wide range of scientific and technological fields. In many cases, they can provide microscopic information for which there are no experimental techniques available or are beyond the experimental possibilities. Application of computational chemistry techniques to CNDs is particularly challenging, due to the difficulty of assessing their structural organization at the atomistic level, and only a limited, albeit rapidly increasing number of investigations have been published in recent years. QM studies have been shown to be increasingly important to better understand the origin of the optical features of CNDs and to indicate the direction to fine-tune them. QM methods are essential to understand the mechanism of the excitation dependence of the CND emissions and to correlate it to their structural and morphological properties, where both ordered and amorphous carbon structure were observed and foreseen to play a role. Due to the high computational cost imposed by large system sizes, the model systems used to explain the optical properties of CND with QM methods are often reduced to smaller molecules or graphene-like layers. Looking through the recent literature, a very good review on state-of-art electronic structure and empirical methods for the CND-parent system graphene was reported by Otyepka and co-workers.^22^ More recently, they also did include computational achievements in explaining the emission properties of CNDs.^23^

Practical exploitation of the fascinating properties of CNDs requires a comprehension of their interactions with small organic molecules as well as with large biomolecules and with the solvating environment. Molecular modeling is of great value in these types of studies, but more approximated methods than first-principles QM are typically required, simply because by increasing the system size, QM methods become soon computationally too demanding and classical modeling techniques represent a compromise between computational cost and the model size and complexity. These techniques are the most reliable in simulating CNDs interaction with protein, nucleic acids, and lipids.

Elucidation of the composition and structure of CNDs surface and inner core, and their mutual interactions, represents typically the major experimental task. However, the details of the structural organization of these nanoparticles are still not completely disclosed by the commonly available experimental techniques, making it arduous to build a model able to properly reproduce the CND properties. To deal with this difficulty, computational chemists try to extract the relevant information from the available experimental findings as guidelines and test suitable structures that satisfy the physical and chemical properties such as vibrational and optical spectra, diffusion constant, reflection patterns, and so on.

In the present work, we have reviewed the published theoretical findings on CNDs, either from purely theoretical or combined experimental and theoretical studies, looking at the specific question of the simulations performed so far. A special focus is devoted to highlight the computational methods specifically chosen to address a key question or property as well as how the chosen computational chemistry model and method compare to corresponding experimental results. By raising up pros and cons of performed simulations, we want to define the present status and boundaries of the theoretical approaches applied to this rapidly emerging family of nanoparticles and inspire researchers with various backgrounds to exploit computational methods to achieve a thorough understanding of CNDs in view of their engineering and technological applications. To this end, the growing applications of molecular modeling, based both on quantum and classical mechanics, to better understand the properties of CNDs are reviewed here.

The review is organized as follows. In section 2, we introduce the reader to main “in silico” methodologies, describing the theory behind them and highlighting for each method what type of information they can obtain to interpret the experimentally observed properties of CNDs. We discuss the main strategies of how to build the model structures for CNDs and how to use atomistic models of CNDs, a particularly tricky issue due to the lack of detailed a priori structural information. We also give suggestions of computational techniques that could be used to study CNDs but that are used much yet or not at all at the moment in this field, whereas they have been used for other quantum dots or related nanoparticles. In this section we also summarize important experimental features of CNDs which can be used as targets properties and benchmarks to test and validate the computational models and methods.

In section 3, we review the studies concerning the structural and optical properties of undoped and doped CNDs as well as the modeling studies of CNDs interacting with other molecular systems, including inorganic nanocomposites, small molecules, and large biopolymers, fundamental to design CNDs to be used as sensors and for biological applications.

Section 4 is devoted for a broader perspective of the field by outlining the advantages and limitations of specific methods and providing suggestions on future directions for research in the field.

## 2 In Silico Methods for CND Studies

In this section, the basic theory behind the computational methods used to study CND is summarized. For each method, the inferable information about CND properties is reported. It is important to note that, since the structure of the target system and its relationships with the chemical and physical properties are very complex, as mentioned in the previous section, some of the reported methods are applied to simplified models of CNDs, such as GQDs or CPDs. We will show, however, that results from such simplified models are also relevant for the more complex CND system.

### 2.1 Quantum Mechanical Methods

The fundaments of quantum chemistry date back to late 1920s and the seminal paper of Dirac in 1929^24^ with the well-known and often cited statement: “The underlying physical laws necessary for the mathematical theory of a large part of physics and the whole of chemistry are thus completely known, and the difficulty is only that the exact application of these laws leads to equations much too complicated to be soluble.” The equation became numerically soluble almost half a century later, thanks to mainframe computers along with numerous approximations to simplify the physical and mathematical description. Table 1 and Table 2 give an overview of relevant computational studies performed with QM methods, together with the properties that were calculated.

**Table 1:** Overview of the QM Methods Used to Simulate the Ground State Properties of Different CD Models^a^

method family	method detail	basis set	computed data^b^	solvent model	dispersion/long-range corrected	system^c^	refs
DFT	B3LYP	3-21G	GO, FRQ		no	fluorophore derived from CZA + amino compounds	Wang et al., 2017^25^
		6-31G	GO	IEFPCM^α,β^	no	functionalized graphene layer (C_132_, C_168_, C_170_)	Zhao et al., 2014^26^
			GO		no	graphene layer and cyclo-1,4-naphthylene with repeated units	Zhu et al., 2013^27^
			GO, ESP, OA		no	ovalene-based functionalized with −COOH and −OH + donor or acceptor molecules	Srivastava et al., 2019^28^
			GO		no	graphene layer functionalized with -NH_2_	J. Wang et al., 2016^29^
			GO		no	N-doped hexagonal and rectangular single graphene layer	Lin, 2018^30^
			GO		no	large N-doped graphene layer	Ghadari, 2017^31^
			GO, FRQ		no	diamond shaped graphene layer	Das et al., 2016^32^
		6-31G(p)	GO, PC, OA	IEFPCM	no	graphene layer with termination of H, OH, O and COOH	Sadrolhosseini et al., 2019^33^
		6-31G(d)	GO,		no	IPCA and azapolycyclic molecules	Shamsipur et al., 2018^34^
			GO		no	graphene layer doped with N, B, P and S atoms	Feng et al., 2018^35^
			GO		no	graphene layer (C_168_ and C_114_)	Schumacher, 2011^36^
		6-31+G(d)	GO, OA		yes	coronene functionalized with -NH_2_	Liang et al., 2020^37^
			GO, OA	IEFPCM^α^	no	CPDs	Sau et al., 2018^38^
		6-31+G(d,p)	GO		no	coronene based functionalized with −COOH	Holá et al., 2014^39^
		6-31G(d,p)	SCFE, OA		no	graphene layer functionalized with −COOH	Bayoumy et al., 2020^40^
		6-31++G(d,p)	GO, FRQ		yes	pyrene and coronene based single/double layers doped with surface groups	Sarkar et al., 2016^41^
			GO, FRQ		yes	N-doped pyrene-based	Holá et al., 2017^42^
			GO, IE	C-PCM^α^	yes	N-doped coronene and pyrene-based	Wu et al., 2020^43^
		6-311G(d)	GO		no	triangular and square graphene layers functionalized with −OH and −COOH	Yuan et al., 2018^44^
		6-311++G(d,p)	GO, OA		no	l-propargylglycine	Ye et al., 2020^45^
			GO	IEFPCM	no	CZA-like	Nandy et al., 2019^46^
			GO, OA	IEFPCM^α^	no	CZA	Mura et al., 2020^47^
			GO	C-PCM^α^	yes	pyrene and coronene-based single and multilayers doped with surface groups	Sudolská et al., 2015^48^
			GO, FRQ, SCFE		no	GPTMS molecules (conformers)	Šapić et al., 2009^49^
			GO, FRQ, SCFE, NMR	IEFPCM^α,γ,δ,ε^	no	CZA ions	Mocci et al., 2021^50^
			GO, FRQ, OA	IEFPCM^α^	no	CZA	Cappai et al., 2021^51^
		DZ	GO, OA	COSMO ^β^	no	amino-functionalized pyrene and perylene	Kundelev et al., 2019^52^
		DZP	GO, OA		yes	PAHs attached to CD surface	Kundelev et al., 2020^53^
		cc-pVTZ	GO	PCM^α^	no	graphene layers functionalized with pyridinic oxide	Singh et al., 2020^54^
	CAM-B3LYP	def2-TZVP	GO	IEFPCM^α^	yes	IPCA	Siddique et al., 2020^55^
			GO, FRQ		yes	functionalized graphene layers	Algarra et al., 2020^56^
	PBE		GO		no	graphene layers doped with N, S or codoped	Xu et al., 2015^57^
			EP		no	graphene layers functionalized with −OH, −COOH, −NH_2_	Yuan et al., 2020^58^
			GO, FRQ, EP		no	N-doped graphene layer	Lazar et al., 2019^59^
		PW (29.4 Ry)	OA		no	diamond shaped graphene layer	Das et al., 2016^32^
			GO	PCM^α^	no	benzene-like aggregates functionalized with −OH, −COOH, −COO	Ambrusi et al., 2019^60^
		PW (36.7 Ry)	GO		yes	bare and B-doped coronene systems	Sen et al., 2019^61^
		PW (44.1 Ry)	GO, OA		yes	graphene hexagonal layers doped with N, P or codoped	Yashwanth et al., 2020^62^
		TZP	ED-A	PCM^α^	yes	N-doped graphene layer	Vatanparast and Shariatinia, 2019^63^
	LDA	DNP	GO		no	graphene layer functionalized with −COOH, −OH and -NO_2_	Choi et al., 2018^64^
			GO		no	graphene layer functionalized with -NH2	Jin et al., 2013^65^
	optB86b-vdW	PW (36.7 Ry)	GO, EP		yes	bare and B-doped coronene systems	Sen et al., 2019^61^
	BP86	def2-SVP	GO, OA		no	boron and boron oxides doped graphene layers functionalized with −OH	Jana et al., 2017^66^
	B97D	def2-SVP	GO	SCRF	yes	amide-capped single and double graphene layer	Strauss et al., 2014^67^
	PZ81	PW (60 Ry)	GO		yes	graphene layers functionalized with −NH_2_, −OH, −F, −CHO, −COCH_3_, and −COOH	Y. Li et al., 2015^68^
	ωB97X-D	def2-SVP	GO	SCRF	yes	amide-capped single and double graphene layer	Strauss et al., 2014^67^
		6-31G(d)	GO		yes	polyamide chains, focusing on dimer and decamer	Vallan et al., 2018^69^
		6-31+G(d)	GO, EP	SMD	yes	partially fluorinated ovalene	Chronopoulos et al., 2020^70^
		6-311G(d,p)	GO		yes	graphene layer containing one or two ether groups (C–O–C)	Chen et al., 2018^71^
		6-31++G(d,p)	GO, OA	C-PCM^α^	yes	Single and double pyrene layers functionalized/doped with – NH_2_ groups, pyridinic dopant atoms, and pyrrolic rings	Sudolská and Otyepka, 2017^72^
			GO	SMD^α^, QM/MM^α^	yes	IPCA monomer and dimer	Langer et al., 2021^73^
	M06-2X	6-31G(d)	GO, FRQ, EP	PCM^α^	yes	N-doped graphene layer	Vatanparast and Shariatinia, 2019^63^
		def2-SVP	GO	PCM	yes	N-doped graphene layer with edge functionalization	Supchocksoonthorn et al., 2019^74^
			GO	PCM	yes	N-doped graphitic layer functionalized with −OH, −COOH	Thongsai et al., 2019^75^
			GO		yes	N-doped graphitic layer with oxygen-containing functional groups (C_52_H_18_N_5_O_9_)	Prathumsuwan et al., 2019^76^
		def2-TZVPP	IE	PCM	yes	N-doped graphene layer with edge functionalization	Supchocksoonthorn et al., 2019,^74^
			IE	PCM	yes	N-doped graphitic layer functionalized with −OH, −COOH	Thongsai et al., 2019^75^
			GO		yes	N-doped graphitic layer with oxygen-containing functional groups (C_52_H_18_N_5_O_9_)	Prathumsuwan et al., 2019^76^
			GO		yes	C_70_H_22_ + different dopants (hydroxyls, carboxylic acid, epoxides, amines)	Sheardy et al., 2020^77^
SEMO	PM3		GO			fluorophore derived from CZA + amino compounds	W. Wang et al., 2017^25^
			GO			graphene layer functionalized with −COOH	Bayoumy et al., 2020^40^
	PM6	PM6-D3H4	GO	PCM		N-doped graphene layer with edge functionalization	Supchocksoonthorn et al., 2019,^74^
		PM6-D3H4	GO	PCM		N-doped graphitic layer functionalized with −OH, −COOH	Thongsai et al., 2019^75^
			GO	COSMO^α^		oxidized graphene layer	Liu et al., 2019^78^
	AM1		GO			amorphous CNDs	Margraf et al., 2015^79^
Hückel	normal		EP			functionalized graphene layer	Kwon et al., 2015^80^
			EP			graphite	Hjort and Stafström, 2000^81^
	extended		EP			pyrene-based	Tepliakov et al., 2019^82^
HF		6-31G(d)	GO, FRQ	cited for MD only		large N-doped graphene layer	Ghadari, 2017^31^
CPMD	PBE	PW (60 Ry)	DYN		no	graphene layer	Shekaari and Abolhassani, 2017^83^
	BLYP	PW (40 Ry)	DYN		no	amorphous carbon with sp, sp^2^ and sp^3^ hybridization	McCulloch et al., 2000^84^
post-HF CC	DLPNO-CCSD(T)	cc-pVTZ	GO, IE	IEFPCM^α^		IPCA	Siddique et al., 2020^55^

a The superscripted α, β, γ, δ, and ε indicate that the model was used to simulate water, toluene, ethanol (EtOH), dimethyl formamide (DMF), and dimethyl sulfoxide (DMSO) solvent, respectively.

b The following acronyms appear in the “computed data” column: GO stands for geometry optimization; FRQ stands for frequencies-derived properties calculation; ESP indicates the calculation of electrostatic potential; OA stands for orbital analysis; PC indicates the partial charges; SCFE indicates the calculation of SCF energies; IE stands for interaction energies; NMR stands for nuclear magnetic resonance (NMR) spectra calculations; EP indicates the electronic properties; ED-A means energy decomposition analysis; DYN stands for dynamics; and QM/MM indicates the hybrid quantum mechanics/molecular mechanics method.

c In the “system” column: CZA is for citrazinic acid; IPCA is for 5-oxo-1,2,3,5-tetrahydroimidazo-[1,2-α]-pyridine-7-carboxylic acid; and GPTMS is for (3-glycidyloxypropyl)trimethoxysilane.

**Table 2:** Overview of the QM Methods Used to Simulate the Excited State Properties of Different CD Models^a^

method family	method detail	basis set	computed data^b^	solvent model	dispersion/long-range corrected	system^c^	refs
TD-DFT	B3LYP	6-31G(d)	ESE		no	N-doped hexagonal and rectangular single graphene layer	Lin, 2018^30^
			ESE	C-PCM^α^	yes	N-doped coronene and pyrene-based	Wu et al., 2020^43^
			ABS		no	polyamide chains, focusing on dimer and decamer	Vallan et al., 2018^69^
			GO, ABS, FL	PCM^α,β^	no	functionalized graphene layer (C_132_, C_168_, C_170_)	Zhao et al., 2014^26^
			GO, HLC		no	fused aromatic rings and cyclo-1,4-naphthylene with repeated units	Zhu et al., 2013^27^
			GO		no	graphene layer functionalized with -NH_2_	Wang et al., 2016^29^
			ESE		no	N-doped hexagonal and rectangular single graphene layer	Lin, 2018^30^
			ESE	C-PCM^α^	yes	N-doped coronene and pyrene-based	Wu et al., 2020^43^
			GO, ABS, HLC		no	graphene layer doped with N, B, P and S atoms	Feng et al., 2018^35^
			GO, ABS		no	graphene layer (C_168_ and C_114_)	Schumacher, 2011^36^
		6-311G(d)	ABS		no	triangular and square graphene layers functionalized with −OH and −COOH	Yuan et al., 2018^44^
		6-311++G(d,p)	ESE	IEFPCM	no	CZA-like	Nandy et al., 2019^46^
		6-311++G(d,p)	ABS	IEFPCM^α^	no	CZA	Mura et al., 2020^47^
		6-311++G(d,p)	ABS	IEFPCM^α^	no	CZA	Cappai et al., 2021^51^
		6-311++G(d,p)	ABS	IEFPCM^α,γ,δ,ε^	no	CZA Ions	Mocci et al., 2021^50^
		DZ	FL	COSMO^β^	no	amino-functionalized pyrene and perylene	Kundelev et al., 2019^52^
		DZP	FL		yes	PAHs attached to the CD’s surface	Kundelev et al., 2020^53^
	CAM-B3LYP	6-31G(d)	ABS, HLC		yes	graphene layer functionalized with -NH_2_	Wang et al., 2016^29^
			ABS, FL		yes	IPCA and azapolycyclic molecules	Shamsipur et al., 2018^34^
		6-311++G(d,p)	ABS,	cited for MD only	yes	Large N-doped graphene layer	Ghadari, 2017^31^
		cc-pVDZ	ABS	cited for MD only	yes	Graphene layer	Osella and Knippenberg, 2019^85^
		def2-TZVP	ABS	SMD^α^, QM/MM^α^	yes	IPCA monomer and dimer	Langer et al., 2021^73^
			GO, ABS, FL, HLC, ESE	IEFPCM^α^	yes	IPCA	Siddique et al., 2020^55^
	OLYP	6-31G(d)	ABS		yes	amorphous CNDs	Margraf et al., 2015^79^
	BHHLYP	def2-SVP	ABS		no	boron and boron oxides doped graphene layers functionalized with −OH	Jana et al., 2017^66^
	PBE	PW (29.4 Ry)	ABS	Vaspsol^α^	no	benzene-like aggregates functionalized with −OH, −COOH, −COO	Ambrusi et al., 2019^60^
			HLC		yes	pyrene, coronene, larger graphene layer (C_62_H_20_)	Long et al., 2017^86^
	PZ81	PW (60 Ry)	GO, ABS, HLC		no	graphene layers functionalized with −NH_2_, −OH, −F, −CHO, −COAH_3_, and −COOH	Li et al., 2015^68^
	ωB97xD	6-31+G(d)	FL		no	coronene based functionalized with −COOH	Holá et al., 2014^39^
			ABS		yes	pyrene and coronene based single/double layers doped with surface groups	Sarkar et al., 2016^41^
			ABS, FL		yes	N-doped pyrene-based	Holá et al., 2017^42^
			ABS		yes	single and double pyrene layers functionalized/doped with −NH_2_ groups, pyridinic dopant atoms, and pyrrolic rings	Sudolská and Otyepka, 2017^72^
			ABS, FL	C-PCM^α^	yes	pyrene and coronene-based single and multilayers doped with surface groups	Sudolská et al., 2015^48^
		6-311G(d,p)	ABS, FL		yes	graphene layer containing one or two ether groups (C–O–C)	Chen et al., 2018^71^
		6-31++G(d,p)	ABS, FL	C-PCM^α^	yes	single and double pyrene layers functionalized/doped with −NH_2_	Sudolská and Otyepka, 2017^72^
		6-311++G(d,p)	ABS	IEFPCM^α^	yes	CZA	Cappai et al., 2021^51^
	M06-2X	def2-TZVPP	ABS	PCM	yes	N-doped graphitic layer functionalized with −OH, −COOH	Thongsai et al., 2019^75^
			ABS	PCM^α,γ,ζ,η,θ^	yes	N-doped graphene layer with edge functionalization	Supchocksoonthorn et al., 2019^74^
			ESE, ABS	PCM^α,γ,ζ,η,θ^	yes	N-doped graphitic layer with oxygen-containing functional groups (C_52_H_18_N_5_O_9_)	Prathumsuwan et al., 2019^76^
	NO-DATA	PW	ABS			C_70_H_22_ + different dopants (hydroxyls, carboxylic acid, epoxides, amines)	Sheardy et al., 2020^77^
ADC	ADC(2)-s, ADC(2)-x, ADC(3)	cc-pVDZ	ESE, OA	cited for MD only		graphene layer	Osella and Knippenberg, 2019^85^
SEMO	NDDO PM3 UNO-CIS		GO, ABS, FL	SCRF		amide-capped single and double graphene layer	Strauss et al., 2014^67^
	NDDO PM6 UNO-CIS		ABS	COSMO^α^		oxidized graphene layer	Liu et al., 2019^78^
	NDDO AM1 UNO-CIS		GO, TC, ABS, OA			amorphous CNDs	Margraf et al., 2015^79^
	INDO/S		GO, ABS			amorphous CNDs	Margraf et al., 2015^79^
Hückel	extended		GO, ABS, HLC, FL			pyrene-based	Tepliakov et al., 2019^82^
HF	OLCAO	LDA	ES, XABS			graphene layers doped with N, S or codoped	Xu et al., 2015^57^

a The superscripted α, β, γ, δ, ε, ζ, η, and θ indicate that the model was used to simulate water, toluene, ethanol (EtOH), dimethyl formamide (DMF), dimethyl sulfoxide (DMSO), methanol (MetOH), ceric ammonium nitrate (CAN), and hexane (HEX) solvent, respectively.

b The following acronyms appear in the “computed data” column: ESE stands for excited state energies; ABS stands for UV–vis absorption calculation; GO stands for geometry optimization; FL stands for fluorescence spectra calculations; HLC indicates the HOMO–LUMO calculation; OA stands for orbital analysis; QM/MM indicates the hybrid quantum mechanics/molecular mechanics method, TC indicates the calculation of thermoelectric data; ES stands for electronic structure; and XABS stands for X-ray absorption calculation.

c In the “system” column, CZA is for citrazinic acid; and IPCA is for 5-oxo-1,2,3,5-tetrahydroimidazo-[1,2-α]-pyridine-7-carboxylic acid.

#### 2.1.1 Early Ideas of Quantum Chemistry and the Hückel Model

As there were no computational machines in the early years of quantum mechanics, there was time to think and lay a solid ground for molecular quantum mechanics. Early spectroscopic measurements did give the needed guidance. Ideas like the molecular orbital (MO) and valence bond (VB) models had their supporters and developers among the great pioneers of theoretical physics and chemistry.^87^ At the early stages, the complicated chemical and physical systems were addressed by very simple models like the Lewis dots in the valence shell electron pair repulsion model (VSEPR) for molecular geometries, MO diagrams for chemical bonding, or the free electron model for conjugated molecules assuming delocalized π-electrons. Knowledge of intermolecular and intramolecular interactions grew from these studies, all contributing to understanding the nature of chemical bonding, electronic and molecular structures, and condensed phases. The first quantum chemical calculations on molecules of chemical interest were possible to be done solely with paper and pen by introducing approximation to the Schrödinger equation based on the combination of phenomenological, classical, and quantum mechanical models. Aromatic molecules and conjugated hydrocarbons, in particular, constituted ideal systems to test molecular quantum mechanics by applying the MO method proposed by Hückel (HMO).^88,89^ These types of molecules have regular topological structures and only one π-electron per atom. Additional σ-bonds could be attached for substituted derivatives. HMO introduces one-electron molecular orbitals as a linear combination of atomic orbitals to build molecular orbitals (LCAO-MO). However, without the spin function, there is no Pauli Exclusion Principle and there was originally no Pauli repulsion between the electrons. Despite the approximation level, HMO or the “simple Hückel method” (SHM) were successful theories in organic chemistry revealing details of quantum phenomena as well as being a solid framework to many more elaborate models. SHM was dramatically improved by Roald Hoffmann in his extended Hückel method (EHM)^90^ to treat both organic and inorganic molecules, inspired by a work of Wolfsberg and Helmholz,^91,92^ who used a decade earlier a Hückel type of MO theory in their pioneering work to compute spectra and electronic structure for transition metal complexes rather than applying the ligand-field theory. In spite of the generalizations, the EHM model with simple constant parameters was not enough to meet the complexity of generic molecular systems and to give accurate results, as it is a single electron method. The Hückel Hamiltonian is a simple starting model for tight-binding (TB) methods frequently used in materials science (*vide infra*). Historically, there have been a few attempts to improve the Hückel Hamiltonian and, in particular, to include sigma-electrons. For example, the Pariser–Parr-Pople (PPP) method included the Coulombic interactions between the atoms.^93^ PPP has two-electron interactions but uses so-called zero-differential overlap (ZDO) approximation, which eliminates most of the two-electron integrals and determines the rest of them empirically.

The Hückel method was applied to model ordinary and hydrogenated vacancies in large graphite-layers (from 114 to almost 4000 atoms)^81^ and to study the photoluminescence of functionalized CNDs.^80^ More recently, the extended Hückel method has been effectively applied to describe the optical properties of CNDs by Tepliakov et al.^82^ In their CND model, a disordered sp^3^ core was surrounded by partially hybridized sp^2^ islands (pyrene or perylene units). The computed absorption and emission properties match very well the experimental ones, relating the excitation-dependent emission to the sp^2^-sp^3^ relative content. Although the EHM, relying only on valence electrons, is limited in the determination of the structural geometry of large organic molecules and its predictiveness largely depends on the parameters applied to calculate the electronic properties,^94^ the inverted core–shell model proposed by Tepliakov^82^ is worth mentioning and was further explored by other authors.^53,79,95^

#### 2.1.2 From Particle-in-a-Box to Quantum Dots

One of the simplest models to understand the quantization of energy in chemistry is the one-dimensional particle-in-a-box (PiB) model where a particle can move freely in a constrained linear space. This simple model demonstrated the failure of classical physics in describing many phenomena in the world of the tiny building blocks of atoms and molecules. PiB consists of a moving particle with a translational kinetic energy and zero potential energy inside the box, allowing the particle to move there unhindered, while it has a finite (or infinite) value at the two borders. Therefore, the particle is trapped inside the box and can only escape by tunneling. The solution of the Schrödinger equation gives the quantized energy levels and the corresponding wave functions. It can be solved analytically and exactly within the model. The energies are shown to depend both on the mass of the particle and the length of the box (well). The narrower is the space and lighter is the particle, the larger the gaps between energy levels become. Wave functions can be used to compute the probabilities to find the particle in the box in its ground and excited states.

The model becomes chemically relevant when we assume that the length of the box corresponds to that of a conjugated polyene and the particles are the delocalized π-electrons (of the carbon atoms) moving along the molecular chain. The model can now be used to describe electronic excitations and explain electron spectra. By equaling the length of the box to the bond lengths and with suitable end corrections, the model can give surprisingly good results. For example, it can predict the color of the conjugated system or chromophore (longer chains will appear in the visible spectrum). The 1D PiB model can be made more realistic by applying different types of potentials inside the box and making the electron less free to move.

The electrons in QD can be described as particles in a 3D box, with spherical shape. In this case, the (side) length of the box (*L*_*x*_, *L*_*y*_, and *L*_*z*_) has to be replaced by a radius *R*. In a 3D box of lengths *L*_*x*_, *L*_*y*_, and *L*_*z*_, the energy levels for 3D PiB can be given as
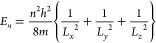
1

Brus^96,97^ did suggest a similar equation for the energy levels of confined particles in a spherical nanocrystal. The energy of the first excited state (in vacuum) becomes
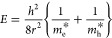
2where *m*_e_^*^ and *m*_h_^*^ are the effective masses of the electron and the positive hole constituting the exciton and depend on the considered quantum dot, whereas *r* is the radius of the nanoparticle. Both the effective masses turn out to be only fractions of the electron mass which can be explained by the Coulombic attraction between the negative electron and positive hole that is strongly screened.^96,97^ The equation above has to be corrected to be valid for the QD inside a bulk crystal by adding the bandgap energy of the semiconductor *E*_gap_ to it. This energy again depends on the given nanocrystal. The wavelength λ of the emission of the QD can be calculated from
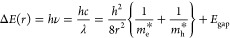
3

Several research and tutorial papers apply PiB to quantum dots. The fundamental modeling work for the excited electronic states is done by Brus^96,97^ for semiconductor crystallites of ∼5 nm in diameter and Nirmal and Brus^98^ for even smaller nanocrystals. In a number of educational papers, the 3D PiB is applied to explain the theory behind quantum dots. Boatman et al. present a synthesis of CdSe quantum dot nanocrystals explaining the transition energies using the PiB model.^99^ Rice and Giffin studied several quantum dots in a polyurethane/acrylic acid polymer composite^100^ using PiB calculations to rationalize the results. Bauer et al.^101^ employed PiB to determine the effective size of quantum dots as sensitizers in solar cells. Onyia et al. applied PiB for several QDs, finding a reasonable prediction of the energy at all sizes of radius.^102^ Venitucci and Niquet used a simple PiB model for hole spin qubit in static electric, magnetic, and radiofrequency electric fields outlining all trends using the model.^103^ Jolie et al.^104^ studied graphene quantum dots grown on Ir(1,1,1) using scanning tunneling microscopy (STM) and applied the relativistic PiB model to investigate the linear dispersion relation between *E*(*k*) and *k*.

#### 2.1.3 Nonempirical Wave Function Methods

Nonempirical methods in quantum chemistry are also referred to as *ab initio* methods, assuming that no empirical information goes into calculations although there are approximations in the physical models to deal with the many-body character of the interactions as well as correlation effects, etc. The quantized energy of a system is computed by solving the Schrödinger equation which, in the time-independent and nonrelativistic framework, can be written as
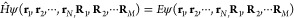
4with *Ĥ* being the Hamiltonian for a system of *N* electrons and *M* nuclei, with positions **r** and **R**, respectively, and ψ is the wave function containing all information of the system. The Hamiltonian can be written in atomic units as
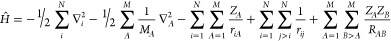
5where ∇^2^ is the Laplacian operator and *M*_*A*_ the mass of nucleus *A* expressed as multiples of the mass of an electron and *Z*_*A*_ its atomic number. By virtue of the Born–Oppenheimer approximation, and considering the nuclei effectively fixed in space while the electrons are moving, the Hamiltonian can be reduced to the following electronic form:
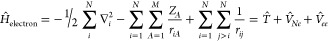
6where *T* and *V* are the kinetic and potential terms. The continuous development of quantum-chemistry consists in finding computationally affordable and accurate enough approximations to the exact solutions of the Schrödinger equation, which can be solved exactly only for trivial chemical systems (such as the hydrogen molecule ion in elliptical coordinates).

A fundamental approximation is the Hartree–Fock (HF) scheme, in which the *N*-electrons wave function of eq 4 is approximated to an antisymmetrized product (Slater *N* × *N* determinant, Ψ_SD_) of one-electron wave functions χ_*i*_ (**x**_*i*_), called spin orbitals:
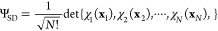
7with **x**_*i*_ being a spatial-spin coordinate. The energy of the system is expressed as
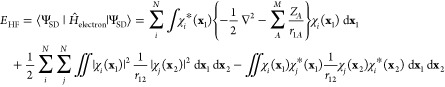
8which is conventionally expressed in a simplified form
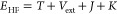
9where *T* comprises the kinetic energy of each electron and *V*_ext_ the Coulomb attraction energy with the nucleus of charge *Z*; *J* represent the Coulombic interaction between two electrons and *K* the exchange integral arising from the antisymmetry of the Slater determinant.

According to the variational principle, *E*_HF_ is always larger than the exact ground state energy (*E*_0_); the difference between the two is due to the lack of electron-correlation description in HF approximation where each electron experiences the average density of all the other electrons (mean field approximation). Within the e-e correlation description, it is worth distinguishing between dynamic and static (also called nondynamical) electron correlation. Intuitively, the former refers to the instantaneous correlation between electrons occupying the same orbital accounting for electron repulsion according to the relative separation, while the latter mainly regards electrons of different spatial orbitals. The latter becomes significant for different orbitals with similar energies (nearly degenerate) and is important for describing, for example, bond formation and breaking or excited states. To account for the static correlation, two or more determinants can be used to describe the wave function.^105^

To improve the HF formalism, a multideterminant trial wave function
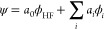
10is used. ϕ_HF_ is the HF one-determinant wave function. In the case of predominantly dynamic correlation, *a*_0_ is typically close to one and the other determinants ϕ_*i*_ appear as a small perturbation. The different electron correlation methods differ in the way the other coefficient *a*_*i*_ are calculated. Static correlation plays a crucial role in the description of the excited states of several molecular systems where the orbitals are close in energy. Although applied to carbon dots to a lesser extent than other methods, the application and computation of electron correlation in so-called post-HF methods are nowadays a vivid research field in quantum chemistry.^105^

The lack of electron–electron correlation in the HF theory is known to lead to several limitations, such as the underestimation of bond energies or deviations from experimental results. Different post-HF methods exist that start from an HF calculation and then apply some correction for the correlation; among these “correlated methods”, the most used are the Møller–Plesset (MPn) methods, the Configuration Interaction method (CI), the multiconfigurational self-consistent field (MCSCF), or the coupled cluster method (CC). MPn^106^ is a perturbative method that corrects the classic HF and expresses wave functions and perturbed energy as a power series. According to the order, one can have MP2, MP3, or MP4.^107^ Because of convergence problems this technique has not found application in CDs problems. In CI, the electronic wave function results in a linear combination of the different configuration state functions. Due to the high computational cost, the CI method is not usually applied to complex systems such as CDs or large clusters. As in the case of CI, CC expresses the wave function as a sum of ground state determinant and those describing the excitation of electrons into virtual molecular orbitals.^108^ The correlated wave function is given by
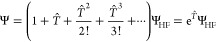
11with *T̂* = *T̂*_1_ + *T̂*_2_ + *T̂*_3_ + ... The excitation operators *T̂*_1,_*T̂*_2_, *T̂*_3_, promote the electrons to virtual spin orbitals. One can have coupled cluster doubles (CCD), coupled cluster singles and doubles (CCSD), or coupled cluster singles, doubles, and triples (CCSDT) depending on the number of terms in the summation.^108^

In general, the inclusion of the electron correlation leads to a large increase in the computational time, and the applications of these post-HF methods is limited to rather small systems.

As can be seen from Table 1 and Table 2, HF methods find rather limited applications in the CDs field, with some use in the geometry optimization,^31^ while post-HF methods are used to calculate the optical properties.^57^ Ghadari has utilized HF/6-31G(d) to optimize the geometry of three different doped and undoped nanographene structures. In particular, graphitic, pyrrolic, and pyridinic clusters have been tested to investigate the adsorption of nucleobases, nucleotides, and their derivates on the nanographene framework as a function of nitrogen doping. This approach has permitted the modeling of the ground state configuration of large graphene quantum dots with crystalline structure in combination with molecular dynamics (MD) and advanced QM methods for biological purposes. Siddique and co-workers^55^ have made use of post HF CCSDT of domain-based local pair natural orbital (DLPNO) to investigate the interaction energies of 5-oxo-1,2,3,5-tetrahydroimidazo-[1,2-α]-pyridine-7-carboxylic acid (IPCA) dimers, a fluorophore that is considered as a potential photoluminescence source of citric acid (CA) and ethylenediamine (EDA) derived CNDs. The results have been compared with density functional theory (DFT) calculations at CAM-B3LYP-D3 level of theory allowing one to identify the most alike dimeric configuration that can occur in aqueous solutions.

Despite the achievements in the description of excited states after the introduction of time-dependent DFT (TDDFT), problems in describing some excited state phenomena are still unsolved.^109^ In this framework, the algebraic diagrammatic approximation (ADC) scheme^110^ proved to effectively describe the excited state effects, such as charge transfer, Rydberg, and doubly excited states.^109,111^ Although it may be considered as a niche approach to most, its implementation in commercial packages has allowed its wider use in the investigation of some molecular systems.^109^ The scheme is based on the polarization propagator describing the time evolution of the polarization of a multielectron system, well explained by quantum many-body theory.^110^ In the propagator are contained the creation and annihilation operators **Ĉ**, for the promotion or annihilation of an electron into the corresponding one-electron state. In a more convenient intermediate states treatment, the excited state basis {ψ_*J*_^0^} can be obtained by the application of the excitation operator {*Ĉ*_*J*_} containing the (single, double, etc.) excitations to the ground-state wave function: ψ_*J*_^0^ = *Ĉ*_*J*_*ψ*_0_. The excited states must be Gram–Schmidt orthogonalized to provide the orthogonalized intermediated state basis {*ψ̃*_*J*_}. Finally, the excited state can be obtained by the diagonalization of the matrix representation
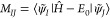
12where *M*_*IJ*_ is the intermediate state representation of the shifted Hamiltonian *Ĥ*. Since the exact ground state is not known, the scheme refers to ground-states obtained by the MPn approach.^109^

The use of post-HF methods in the CND field is presently very limited compared to the much less costly DFT based methods, described below, which can be used to a large extent also in the calculation of the excited states. However, it is worth highlighting the growing success of post-HF and related methods in the study of small molecular systems used as a model for the description of the properties of excited states in carbon dots.^112−114^ These include SOS-ADC(2)/SOS/MP2, applied for example to polycyclic aromatic hydrocarbons, in their dimeric or doped forms, as discussed in Section 2.1.11 (Table 3.

**Table 3:** Overview of the Benchmarking Studies Performed for the Calculation of CD Properties

method family	method details	basis set	computed data^a^	system	refs
DFT, MPn	B3LYP, CAM-B3LYP, SOS-MP2	SV(P), TZVP, def2-TZVP	GO	pyrene and coronene based	Shi et al. 2019^160^
					
					
TD-DFT, DFT/MRCI, ADC, NEVPT	SOS-ADC(2), CAM-B3LYP, BHLYP, SC-NEVPT2	SV(P), TZVP, def2-TZVP	ES, EI	pyrene and coronene based	Shi et al. 2019^160^
					
					
					
DFT, MPn	B3LYP, CAM-B3LYP, MP2	SV(P), def2-TZVP	GO	pyrene and coronene based	Shi et al. 2019^236^
					
					
TD-DFT, DFT/MRCI, ADC, NEVPT	SOS-ADC(2), CAM-B3LYP, BHLYP, SC-NEVPT2	SV(P), def2-TZVP	ABS	pyrene and coronene based	Shi et al. 2019^236^
					
					
					
TD-DFT, DFT/MRCI, ADC	SOS-ADC(2), CAM-B3LYP, B3LYP, BHLYP	SV(P), def2-TZVP	EE, SS	pyrene and coronene based	Shi et al. 2019^114^
					
					
					
DFT, MPn, DLPNO-CCSD(T)	D3-B3LYP, CAM-B3LYP, SOS-MP2	def2-TZVP	GO	N-doped pyrene-based	Shao et al. 2020^240^
					
					
TD-DFT, DFT/MRCI, ADC, NEVPT	SOS-ADC(2), CAM-B3LYP, B3LYP, BHLYP, SC-NEVPT2	SV(P), def2-TZVP	ABS	N-doped pyrene-based	Shao et al. 2020^240^
					
					
					
					
DFT, MPn	D3-CAM-B3LYP, SOS-MP2	SV(P), TZVP, def2-TZVP	GO	F-doped pyrene-based	Liu et al. 2020^113^
					
					
TD-DFT, DFT/MRCI, ADC	SOS-ADC(2), CAM-B3LYP	SV(P), def2-TZVP	EE, SS	F-doped pyrene-based	Liu et al. 2020^113^
					
					
DFT, MPn,	SOS-MP2, ωB97X-D, LC-DFTB2	def2-SVP, def2-SV(P)	GO	tetracene dimer	C. A. Valente et al. 2021^112^
					
					
ADC, TD-DFT, DFT/MRCI	SOS-ADC(2), ωB97X-D, DFTB, LC-DFTB2, CAM-B3LYP, ADC(2), BH-LYP D3(BJ)	def2-SVP, def2-SV(P), ob2-1-1, 3ob-3-1	EI, CTI	tetracene dimer	C. A. Valente et al. 2021^112^
					
					
					
					
					
					
					
					

a The following acronyms appear in the “computed data” column: GO stands for geometry optimization; ES stands for excited states; EI for excitonic interactions; ABS stand for UV–vis absorption calculation; EE stands for emission energies; SS stands for Stokes Shift; and CTI stands for charge transfer interactions.

#### 2.1.4 Semiempirical Methods

Roothaan’s early matrix formulation of the SCF HF method was easy to program and became dominating for decades in computational quantum chemistry.^115^ As nonempirical *ab initio* HF calculations of many-electron molecules were very time-consuming and did need large storage space for the two-electron integrals in the iterative process, so-called semiempirical (SEMO) methods appeared as an affordable alternative. In these methods a large part of the many-center electron integrals were approximated or fitted to experimental estimates or most often even set to zero as they were assumed to give only minor contributions or largely cancel out.

SEMO methods have been developed since the early days of computational chemistry. Most of them start from HF-Roothaan equations and were initially developed to tackle many-atom molecular systems without bottlenecks and also to increase the calculation speed. They are still evolving with the goal to increase the accuracy of the results, eliminate unpredictable errors and overcome limitations of the HF methods.^116^ They all use frozen core approximation and molecular orbitals based on linear combination of valence atomic orbitals (LCVAO-MO). Some integrals in the HF equations, usually three- or four-center integrals, are neglected, while other integrals are calculated approximately, and some are replaced by empirical parameters.

Three schemes were developed in Pople’s group in mid-1960s: CNDO which stands for complete neglect of differential overlap, INDO which comes from intermediate neglect of differential overlap and the neglect of diatomic differential overlap (NDDO). Most of the next generation of SEMO methods are based on NDDO. These include MNDO (modified neglect of diatomic overlap) by Dewar and Thiel^117^ and Austin Model 1 (AM1) by Dewar et al.^118^ and Parametric Method 3 (PM3) and 6 (PM6) by Stewart.^119,120^ OMX (X = 1,2,3) are NDDO-based schemes with orthogonalization corrections from Thiel.^121^

SEMO methods are computationally much faster than *ab initio* methods but significantly slower than corresponding molecular mechanics (MM) calculations; they can be applied on relatively large molecular systems and biomolecules or to screen the properties of thousands of compounds. Several examples from different areas and comparisons of methods are found in refs (122−126). As semiempirical, they can be used well as the QM part in QM/MM calculations of biomolecular systems in solution. They can give semiquantitative molecular properties using very modest computer resources. However, they have some typical problems, relative energies are often not fully reliable and the errors tend to be unsystematic. Importantly, for a reliable description of noncovalent interactions, correction terms should be included,^127,128^ as the D3H4 correction, for proper description of dispersion and hydrogen bond (H-bond) terms. Tu et al. did propose an extended NDDO (ENDDO) scheme where the original NDDO model (the basis for most currently used SEMO schemes) can be considered as zeroth-order approximation to accurate electron–electron interactions.^129^ After adding the first-order correction into the zeroth order, Coulomb interactions and the total energies were significantly improved. The error was reduced while the total energies were consistently slightly higher than in corresponding *ab initio* calculations.

SEMO methods can be used also to study optical properties. A scheme, based on the INDO approximation and originally suggested by Ridley and Zerner more than 50 years ago, is still widely used to explore electronic transitions in large molecular systems,^130^ including CNDs, and is referred to as the INDO/S (or ZINDO) method.^131^

NDDO-based methods using unrestricted natural orbitals (UNOs) as the reference for CI calculations (UNO–CIs) can give good results in the prediction of optical band gaps of carbon based materials at a reasonably low cost.^132^ The UNO-CIS methods, where only single excitation is used to calculate the excited state, has been tested using several SEMO methods (AM1, PM3, PM6, MNDO) and provided an overestimation of the band gaps by 0.1–0.5 eV. Since SEMO methods are much faster than TDDFT, the former can be used to calculate electronic transitions in systems not easily amenable with the latter.

As summarized in Table 1 and Table 2, SEMO methods are finding new useful applications in the field of CNDs. They have been used to optimize the entire structure, or that of relevant portions of the CNDs, and even for modeling their interactions with other molecules. The SEMO structures can be used as starting configurations to perform more accurate *ab initio* geometry optimization and/or electronic structure and electron transition (ET) investigations.

ETs can be investigated either at a proper SEMO level or at higher levels of theory; see, for example, the works of Supchocksoonthorn et al.^74^ and Thongsai et al.^75^ that investigated CNDs alone or interacting with other molecules. They did first optimize the geometries with PM6-D3H4, thereafter using DFT and finally calculated the optical absorptions using TDDFT.

Due to their modest computational costs, SEMO methods can be employed in QM studies of CDs interacting with large macromolecules, such as biopolymers,^40^ or to sample various degree of functionalization and their effect on the optical properties in relatively large CND. An example of the latter is the work of Liu et al.^78^ applying the semiempirical PM6 Hamiltonian to calculate the lowest energy structure for their model of CNDs, representing the system at various degrees of oxidation. The initial structures were optimized with Force Field methods, using the COMPASS FF.^133^ From the generated structures, the optical absorption spectra were calculated by means of the configuration interaction with all single excitations.

Strauss et al.^67^ followed a different approach in combining DFT and SEMO methods to study the possible relationships between structure and optical properties of the CNDs they had synthesized. They used either a DFT approach to optimize the structure of their CND models or sampled the conformational space in classical molecular dynamics simulations. The optical properties of selected sampled conformations were studied with the UNO-CIS methods, a much faster alternative to TDDFT, allowing one to treat large systems or a large number of systems or configurations.

The structure and heat of formation of amorphous CNDs were calculated by Margraf et al.^79^ using AM1 and optical properties with AM1-UNO-CIS and INDO/S schemes and compared the results from TDDFT for the smaller particles. The used SEMO methods were found to estimate reasonably well the band gaps even for large and amorphous CNDs.^134^^135^

Most likely many of these robust semiempirical schemes, initially considered compromises between speed and accuracy, will get a second life parametrizing them using machine learning (ML), the same way as molecular mechanical FF and EAIP for metals.^134^ We have already seen this in parametrizing repulsive potentials density functional tight-binding.^135^

#### 2.1.5 Quantum Density Functional Theory (DFT)

Among the quantum mechanical methods applied to the study of the optical properties of CDs, DFT methods are by far the most used. Compared to pure wave function theory methods such as HF, DFT methods has a greater capability of coupling reasonable accuracy with relatively low computational cost. DFT has the benefit to incorporate some of the correlation among electrons, with a much lower computational cost than correlated post HF wave function methods.

DFT methods derive from the 1964 Hohenberg and Kohn theorems,^136^ stating that all the ground-state properties of an N-electron system are uniquely determined by the total electron density. In the later Kohn–Sham DFT formulation (by far the most used among those existing), the total energy of the ground state is expressed as a sum of exact terms, and an important (although small) contribution to the energy is given by the exchange-correlation term *E*_XC_:
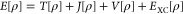
13where *T* is the noninteracting electron kinetic energy, *J* the Coulomb energy, and *V* is the energy due to the external field generated by the nuclei. The first three terms can be computed exactly, while the exact *E*_XC_ functional form is not yet known except that for a uniform electron gas, and only approximate forms can be used. An increasing number of functionals, each one with its strength and limitations and differing in the way they approximate the exact XC term, is available in the most common quantum mechanics codes.

The simplest approximation of the XC term is the local density approximation^137^ (LDA) and its generalization including electron spin LSDA (local spin density approximation). The energy is typically separated to an exchange and in a correlation part:
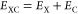
14While LDA has been widely used for studying bulk properties in solid state physics, it is not appropriate to study surfaces or molecules, since it overestimates the bond energies and produces too short bond lengths.

A more sophisticated approximation emerged in the 1980s, making use of both the spin densities and of their gradients (GGA, generalized gradient approximation); typically, but not necessarily, the exchange and correlation terms are separated. The two main approaches to GGA make use of parameters obtained either by a fitting to some data sets, as in the B86 Becke approach,^138^ or derived using theoretical conditions, as in the Perdew and co-workers approach.^139,140^ The development of these functionals was fundamental for DFT to enter into chemistry.

A broadly used variant of the GGA, largely applied to the study of the structure and properties of CDs, is constituted by the hybrid-GGA methods, which typically combine the HF exchange integrals with a GGA exchange functional. These types of functionals are commonly referred as “global hybrids” since they are applied on the whole spatial domain without any truncation in short- and long-range domains as in the case of range-separated (RS) hybrids. The popular B3LYP, developed by Stephens and co-workers,^141^ is an example of global hybrid functional, and it was derived from the three-parameter hybrid GGA functional B3PW91,^142^ obtained in 1993 by Becke, by replacing in the PW91^143^ the correlation terms with the LYP GGA.

There is a large and increasing number of hybrid functionals, and popular quantum mechanical software packages, like Gaussian^144,145^ and NWChem,^146^ allow one to choose among dozens of them or even to tune them through a flexible combination of their HF and GGA components.

The most used functionals in chemistry and material science are those based on the B3LYP and PBE functionals,^147^ respectively. However, there are several limitations in using these functionals, such as the reproduction of dispersion forces and the incorrect behavior of the XC functional at long-range, which have relevant impact on the charge-transfer excitation. A promising approach is represented by the RS hybrids functionals in which, differently from the above-described functionals, the XC functional is divided into short-range and long-range contributions.^148,149^ The popular CAM-B3LYP and ωB97XD belong to this class.

Another DFT related approach which has been recently improved is the so-called multireference configuration interaction (DFT/MRCI) method.^150,151^ In this approach, the total electronic correlation is described by properly adding to the truncated multireference expansion, a DFT contribution in order to take into account the dynamic correlation. However, as shown in Table 3, the applications of DFT/MRCI methods to CDs systems are still very limited and mainly focused on benchmarking. In particular, the DFT/MRCI can outperform DFT in the case of (i) emission energies calculations, (ii) correct ordering of the excited states, and (iii) open-shell or double excited electronic configurations (see section 2.1.11 for a detailed description). However, this approach lead to an overall increase in the computational cost, due to its intrinsic multireference nature.^152^

Focusing on the specific challenge of including the dispersion in DFT calculations, it must be noted that an important source of error is due to the inaccurate description of van der Waals interactions.^153^ In particular, LDA or GGA functionals and the majority of hybrids originating from a simple linear combination of them (as B3LYP) lack in the description of the long-range van der Waals interactions, therefore missing the accurate description of the typical attractive 1/*R*^6^ (where *R* is the internuclear distance) contribution.^154^ A straightforward solution to this problem is to add an empirical pairwise term proportional to *C*_6_/*R*^6^, which depends only on the internuclear distance. The main issue related to this approach is the actual choice of the tabulated *C*_6_ coefficients that are usually dependent on the chemical environment. This approach has been fully extended by Grimme, and the whole periodic table has been covered in order to describe different types of interactions.^155^ These kind of functionals are usually denoted as DFT-D. Finally, a fundamental source of error in standard DFT is the so-called “self-interaction error” (SIE) arising from the impossibility to distinguish two-body electrostatic interactions from spurious self-interaction contributions.^156^ Even if suitable procedures have been developed in order to partially reduce SIE, pure exchange correlation functionals cannot completely remove SIE, which is still an open problem in the DFT field.

The inclusion of a significative portion of HF exchange into the hybrid exchange-correlation functional is considered as the best solution to deal with SIE even if a complete SIE cancellation cannot be obtained.^156^ Alternative solutions involve the extension of the Perdew–Zunger self-interaction correction (SIC) to DFT,^157^ the use of localized orbital scaling corrections,^158^ or multiconfiguration pair-density functional theory (MC-PDFT).^159^

Concerning the applications of DFT methods in CNDs studies, from the data reported in Table 1 and Table 2, where the most relevant computational details on the calculations performed with the QM method on CDs are summarized, it can be clearly seen that most investigations have focused on understanding CNDs structure and properties using small molecular models make use of DFT methods and that B3LYP is the most used functional. The target feature is in general the optical absorption since the computations of the emission properties are not always straightforward. Sometimes the density of states (DoS) or the vibrational spectra are also calculated and compared to the experimental results. It is important to note that the fact that the B3LYP functional is the most used does not imply that it is always to be recommended, and concerning this point, we refer the reader to the studies performed to compare the performance of different functionals in the system of interest for CNDs.^113,160^ A detailed description on benchmarking applied to CNDs can be found in section 2.1.11.

#### 2.1.6 Density Functional Tight-Binding

DFT is currently the method of choice for molecular systems up to several hundreds of atoms with still a reasonably accuracy. SEMO methods that were described in section 2.1.4 allow very much larger systems to be studied but have fundamental weaknesses, and the results depend largely on the parametrization. An alternative to SEMO methods in materials science is empirical TB, which is a simple scheme for electronic structure calculations but critically dependent on tedious fitting of the parameters for each individual system.^161^ One approximate parameter-free electronic structure calculation method which has gained much popularity is the *ab initio* DFT-like tight-binding (DFTB), first proposed by Sankey and Niklewski^162^ based on Harris-Foulkes functionals^163,164^ originally a nonself-consistent approximation to Kohn–Sham functionals.^165^ It was improved by Lewis et al.^165^ using GGA. Since then, the development of DFTB schemes has been vital. Tu et al.^166^ proposed a highly accurate, reliable, and robust self-consistent DFTB method which starts from a simplified Harris–Foulkes functional where the Kohn–Sham electron–electron interactions are expanded in series with respect to reference densities neglecting the second and higher-order corrections. DFTB today has grown to a very powerful and popular scheme for many reasons, and it can be widely applied from nanomaterials to biological systems. It can be used as an MD engine by calculating the forces from energy gradients. Also, it exists as time-dependent TD-DFTB^167^ for spectroscopic and catalytic studies. Early developments and a tutorial to DFTB is found in a paper by Koskinen and Mäkinen.^168^ An excellent summary of the theoretical foundations is given by Seifert and Joswig,^169^ and a complete review of their applications is given by Christensen et al.^170^ DFTB schemes are now implemented in many popular software packages, and several special-purpose programs are available. The group of Frauenheim has developed a package called DFTB+ since the mid-1990s with frequent improvements throughout the years. Recently, collective efforts in the development work are gathered in a general atomistic quantum mechanical simulation package with many options and facilities.^171^ Certain tight-binding methods are developed also for magnetic materials and spin dynamics.^172^ They are very much used for carbon materials and nanomaterials in general. The semiempirical so-called extended TB methods (xTB) can currently treat up to 86 elements of the periodic table.^173^ Although not yet used for carbon nanodots per se, they are already widely applied for GQD and carbon clusters, for example, in a combination tight binding/Hartee-Fock/configuration interaction, where electron–electron correlations are important for the properties.^174^ For CND, the extended Hückel tight-binding method is more used. Zheng et al.^175^ did use a TB model to create the molecular-like CND structure, fitting it to match the experimental energy gap between HOMO and LUMO energy levels (HL gap) in their work to describe a new fluorescence/spectro-electrochemical method to study both the photoluminescence and wavelength dependent photocurrent of CNDs. Both the UV–vis absorption and electrochemistry were applied to quantify the energy gap of the CNDs to calibrate their computational model for CNDs’ electronic energy levels. Their tight-binding model for individual CND molecules combines the conjugated π states with the functional groups (COO, C–O, and COOH) associated with the surface electronic states. Their combined experimental and theoretical investigation of CNDs provides a new insight on the optoelectronic properties of CNDs for use in biomedicine, chemical sensing, and photoelectric devices.

#### 2.1.7 Time Dependent Density Functional Theory

TDDFT is an increasingly popular method to treat the electronic excited states and study the optical properties of a variety of molecular and periodic systems. It solves numerically the time-dependent Schrödinger equation.^176,177^ Excitation energies, photoabsorption spectra, and frequency-dependent response properties can be calculated by analyzing the time response of the systems subject to an external time-dependent potential.^178,147^

As DFT, also TDDFT is based on the idea of replacing the real interacting many-body electronic system by a noninteracting one having the same electron density. However, the construction of such noninteracting system is much more complex for TDDFT, mainly because the time-dependent effective potential at any given time depends on the value of the electronic density at all previous instances.

TDDFT is based on the theorem of Runge and Gross (RG)^179^ and a time-dependent equivalent to that of Hohenberg–Kohn,^136^ and for an initial electronic wave function ψ_0_, it can be shown that there is a 1:1 correspondence between the time-dependent external potential *v*(**r**,*t*) of a many-body system and its time-dependent density ρ(**r**,*t*). Therefore it is possible to write the external potential *v*(**r**,*t*) as a functional of the density:
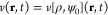
15

The RG theorem allows one to substitute the time-dependent many-body wave function with the corresponding time-dependent electron density. This reduces the number of variables from 3*N* down to the 3 (*x, y, z*) Cartesian variables, *N* being the number of electrons.

As the potential of a functional of the density, the time-dependent Hamiltonian also becomes a density functional; therefore, the time-dependent wave function ψ and all the observables *O*(*t*) are given as
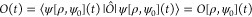
16

Similarly, as for DFT, the final step of TDDFT becomes the determination of an analogous many-body noninteracting system of electrons, thereby having the same density as the real interacting system. Following DFT, this system is known as the time-dependent Kohn–Sham system.^179^

Likewise, the exact time dependent density, ρ(**r**,*t*), can then be calculated from a noninteracting system of *N* single-particle orbitals:
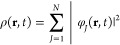
17

The orbitals φ_*J*_ (**r**,*t*) are the solutions of the time-dependent Kohn–Sham equation:
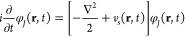
18where the time-dependent effective potential *v*_s_ (**r**,*t*) is given by
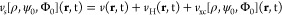
19and where *v*(**r**,*t*) is the time-dependent external potential, *v*_xc_ is the exchange and correlation functional and *v*_H_ (**r**,*t*) is the time-dependent Hartree potential:
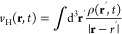
20

The main applications of TDDFT are the calculations of excited states energies of isolated systems. The excitation energies are obtained under the assumption of a linear response of the electronic density, applying an external time-dependent potential. It is therefore possible to estimate the excitation energies as poles of specific linear response functions.^180^

To setup an accurate TDDFT calculation, the following requirements must be satisfied: (i) a reliable functional form for the time-dependent exchange and correlation potential, (ii) an efficient numerical solution of the Kohn–Sham equations, and (iii) an accurate numerical procedure to estimate all the meaningful physical observables from the time-dependent density.

Since in most applications the main interest is on systems lying initially in the ground state, the XC functionals can be rewritten as dependent on the electronic density. However, the density dependence of the XC potential is tricky and also nonlocal, with the XC potential dependent on the densities at all the other points in space and at all previous times. One simple solution to this problem is to use an adiabatic approximation in which the XC potential of the ground-state is evaluated at the instantaneous time-dependent density,^181^ exact only in the case of a slowly varying ground state density.^182,183^

Due to its simplicity and ease of implementation, most time-dependent Kohn–Sham calculations use the adiabatic LDA (ALDA),^184^
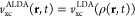
21or any adiabatic GGA defined in a similar way, by replacing the ground-state density with the instantaneous time-dependent density.^185^

In principle, TDDFT can provide the exact time-dependent density ρ(**r**,*t*) while all the corresponding observables can be extracted from the density. The electron density and the total energy are estimated in this way, while other observables such as the state-to-state transition probabilities or the photoelectron spectra can easily become more cumbersome to calculate.

TDDFT has been implemented in several major quantum chemistry computer codes, e.g., Octopus,^186^ Gaussian,^144^ NWChem,^146^ and TURBOMOLE.^187^ In general, two important features are needed for an efficient TDDFT code: (a) the Kohn–Sham orbitals and the corresponding density, must be represented in the real space. A suitable basis set must be implemented on a spatial grid using finite-element or finite-difference discretization schemes; (b) time must be discretized and the time-dependent Kohn–Sham equations propagated forward in time at different time-steps ensuring norm conservation.

In general, many-body systems are subjected to relatively small perturbations and do not strongly deviate from their ground states, for example, in most spectroscopy applications, where the response to a weak perturbation is used to determine the spectral properties of a system. In this case, perturbation theory can be applied within the linear response regime. The aim is to directly calculate the variation of a certain observable to first order in the perturbation, without explicitly calculating the corresponding variation of the wave function.

Within the linear-response regime, the variation of the Hartree and the XC functional can be expanded with respect to the density variation giving rise to a specific response equation named the Dyson equation of TDDFT. It is in principle possible to derive the excitation energies of the system by numerically solving this equation. Some alternative linear-response schemes are, for example, the Casida formalism^188^ and the Sternheimer equation.^189^

To conclude this brief introduction on TDDFT, some limitations of this method should be also mentioned. First, the accuracy of TDDFT and DFT from which it originates, is dramatically dependent on the adopted XC approximation.

Besides this issue, TDDFT is also characterized by its own very specific problems. In fact, while in the case of DFT calculations, some suitable XC functionals can be developed using some specific properties of the ground state as a reference; in the case of TDDFT, this approach is severely limited by the short lifetime of excited states.^190^ In addition, the excited states can be very sensitive to the presence of a solvent environment. In order to consider its presence, more refined and computationally expensive techniques must be used, e.g., a linear-response approach.^191^

In the general conclusions drawn in the monumental review of Laurent and Jacquemin^190^ they report that the amount of exchange included in the XC functional is usually related to a corresponding increase of the transition energy.^190^ In addition, the presence of charge transfer plays a great role in determining the choice of the functional. In this case, the use of long-range corrected functional or double hybrids seems to be recommendable.^190,192−194^ In particular, from large benchmark calculations performed on more than 700 excited singlet states, it was found^194^ that the pure functionals are almost always a poor choice. This is confirmed by a rapid inspection of Table 2, where only very few works on CDs make use of pure functionals.

In the case of global hybrid functionals, Adamo and co-workers^194^ found that X3LYP, B98, PBE0, and mPW1PW91 outperform the others as well as the use of LC-ωPBE for neutral molecules or BHHLYP when vibronic effects must be included.

For an extensive review of the TDDFT methods the reader is referred to refs (190 and 194−197). Concerning the applications of the TDDFT methods to explain the properties of CNDs, inspection of the data reported in Table 2 reveals that they are the most used methods in the study of the excited state of molecular models of CNDs. Indeed, TDDFT is currently the most applied method to calculate optical absorption and, rarely, emission of CNDs molecular models. Considering that the optical properties are a key feature of CNDs, we dedicated an entire subsection (section 2.1.11) to the study of the comparison of the methods aimed at their understanding, where several examples of TDDFT to CNDs molecular models are discussed, comparing the results with those obtained with other methods.

#### 2.1.8 Car–Parrinello Dynamics

The Car–Parrinello (CP) method is a computational technique proposed by Roberto Car and Michele Parrinello in 1985,^198^ which combines molecular dynamics simulations and density-functional theory allowing, in principle, to explicitly simulate chemical reactions in a large variety of organic and inorganic materials.^199^ The CP method is considered a computationally cheaper alternative to the common Born–Oppenheimer (BO) molecular dynamics methodology, where the calculation of the forces for the classical motion of the nuclei is performed by explicitly solving (usually at DFT level) the time-independent Schrödinger equation. On the other hand, in the Car–Parrinello method, the electrons are explicitly treated as degrees of freedom (DoF) by using fictitious dynamical variables.^200^

The CP method starts by initially calculating, usually by means of DFT, the ground-state electronic density corresponding to the initial nuclear configuration. Then, the forces on the nuclei are estimated (based on the ground state density previously calculated) in order to numerically integrate the classical equation of motion for the nuclei and update their trajectory. At the same time, the electronic orbitals are allowed to evolve with the nuclei by treating them as a set of extra DoF with an associated (fictitious) mass whose value is critical for the stability of the whole dynamics.

In the CP method, the calculations of the forces acting on the nuclei is in principle not exact (as in the BO dynamics), since the corresponding electronic density is not exactly on the ground state during the dynamics. However, it is possible to control this uncertainty by appropriately choosing the value of the fictitious masses.^198−200^

CP molecular dynamics simulations are computationally very expensive in comparison with conventional MD so the size and time scale of the CP simulations are limited to few thousand of atoms and hundreds of picoseconds, respectively.^199,200^ To study computationally large systems with a reactive region, a common strategy is to use combined QM/MM approaches where the QM simulation (MD or MC) is performed on the reactive and smaller part of the system which is embedded in a bigger environment treated with classical molecular simulations.^201^ QM/MM has not, however, become a mainstream method as it is still difficult to treat the QM/MM coupling correctly. We discuss QM/MM methods in more detail in section 2.2.2.5 below. In the field of CDs, CP molecular dynamics have been exploited to study the structure and band gap of amorphous carbon as a function of the overall mass density focusing on the sp^2^/sp^3^ fractions’ ratio.^84^ CP molecular dynamics work to explore the melting dynamics of GQDs, composed by 6 and 10 carbon atoms, respectively, has been reported.^83^

#### 2.1.9 Basis Sets

The results of QM calculation depend both on the used method and for each method on the used basis set, i.e., the set of functions that are used to describe the electronic wave function.^202−204^ The most commonly used basis sets are (i) plane waves (PW) that are primarily used within the solid-state community^205^ or (ii) atomic orbitals (AO) that are mainly used by the quantum chemistry community.^206^ Several types of AO are typically used: Gaussian-type orbitals (GTO),^207^ Slater-type orbitals (STO),^208^ or numerical-type orbitals (NTO). Within these three, Gaussian-type orbitals are by far the most popular ones, since they allow the most efficient implementations of density functional theory and Hartree–Fock calculations.^209^

The accuracy of the PW basis sets is specified by a single parameter, i.e., the electronic kinetic energy (*E*_kin_) cutoff: the higher the *E*_kin_, the more accurate the basis set. The value of *E*_kin_ is strictly related to the actual number of plane wave functions being utilized as basis functions, since an infinite number of basis functions is computationally unfeasible.^210^

In the case of GTO basis sets, an increase in accuracy at a reasonable computational cost is obtained by varying not only the number but also the intrinsic parameters of the primitive function. The computationally cheaper basis sets are usually named *minimal basis sets* in which a single basis function per orbital is used in a Hartree–Fock calculation on the free atom. The most used minimal basis set is STO-*n*G,^211,212^ where the integer *n* is the number of Gaussian functions used to represent each Slater-type orbital. The low computational cost of the minimal basis sets is strictly related with their intrinsically low accuracy which usually make them not sufficient for research-quality publications.

A standard procedure to improve the description of the long-range tail of electronic wave functions is represented by the inclusion in the basis set of the so-called *diffuse functions*:^213^ an additional set of atomic orbitals (usually, but not limited to, s and p orbitals) having suitable selected exponents in order to adequately describe the electronic wave function in the outer region of each atom better accounting for long-range interactions.^213^

Along with diffuse functions, another set of functions is usually added to the minimal basis set, the so-called *polarization functions*,^214^ which are particularly important for the correct description of covalent bonds. The idea supporting the use of polarization functions is that the addition of higher harmonics to each spatial orbital can significantly increase the flexibility of the wave function in adapting to more complex geometries by mimicking the effect of polarization of specific bonds. In this perspective, d and f contributions can be added to the p atomic orbitals as well as p and d contributions that are included in s orbitals, finally improving the accuracy in the description of covalent and hydrogen bonding.^215^

A paradigmatic example illustrating the crucial importance of diffuse and polarizations functions is represented by the studies involving energy calculations on stacked benzene dimers,^216,217^ where the inclusion of diffuse and polarization functions was proved to be mandatory for a correct description of the system equilibrium structure.^217^ In the case carbon dots, a correct description of both geometries as well as hydrogen bonding can be obtained by explicitly including polarization and diffuse contributions,^218^ with the latter providing a non-negligible contribution to the overall polarizability.^218^

Moreover, the use of diffuse and polarization functions is highly recommendable for a correct description of electronic excited states since, in this case, molecular orbitals display a natural increase in their spatial extension. The use of diffuse and polarization functions guarantees significant improvements in the quality of the results even in smaller systems, such as ethylene,^219^ and allows the correct description of dipole binding interaction^220^ as well as the UV–visible spectrum.^221^

Indeed, as a rule of thumb and general statement, the accuracy in predicting vertical transition energies increases when a larger basis set is used. This conclusion seems not to be restricted to the case of Gaussian type orbitals: in fact, it was shown by Fülscher et al.^222^ that even in a system constituted by pyrazine described by atomic natural orbitals, the use of a smaller basis set proved to be detrimental and an increase in the deviation of predicted vertical transition energies was evidenced.

Moreover, in larger molecules, such as bicyclic chromogens, Jacquemin et al.^223^ clearly evidenced that diffusion functions are not only beneficial but indeed necessary to obtain spectroscopic data in good agreement with the experiments while vibrational energies appear to be less affected by this choice.

In any case, the inclusion of diffuse functions in the basis set is a critical point that should always be carefully addressed since, as found by Truhlar et al.^224^ in the case of conjugated molecules, such as butadiene, the correlation between accuracy and number of diffusion functions is not necessarily monotonic, demonstrating that the inclusion of a higher number of diffusion functions can eventually degrade the overall quality of the results.

Another possible strategy to increase the GTO accuracy is the use of multiple basis functions (named split-valence basis sets) corresponding to each valence atomic orbital called valence double, triple, quadruple-ζ basis sets. Different split-valence basis sets are identified using the notation X-YZg, where X is the number of primitive Gaussians for each core atomic orbital, Y and Z, instead specifying that each valence orbital is composed by two basis functions: a linear combination of Y and Z Gaussian functions. The two numbers after the hyphens indicate a split-valence double-ζ basis set. Triple- and quadruple-ζ basis sets are also commonly used.^225^

In the case of CNDs, as shown by data in Table 1 and Table 2, most of the DFT calculations have been performed using GTO 6-31G and 6-311G basis sets. In the case of PW calculations on CNDs, different *E*_kin_ values were used depending on the adopted pseudopotentials (see Table 1 and Table 2) ranging from a minimum value of 30 Ry in the case of ultrasoft pseudopotentials^32,57,60^ to a maximum value of 60 Ry for norm-conserving pseudopotentials.^83^

An important theoretical reason which makes GTO orbitals more suitable for the study of CDs is given by the fact that the use of PW basis sets precisely require periodic boundary conditions^226^ in contrast to GTO basis sets in which these conditions can be eventually limited to two, one, or zero directions when dealing with low dimensional systems.^210^

Finally, we mention that GTO basis sets suffer from the basis set superposition error (BSSE) which is a consequence of the finite expansion of basis functions affecting the relative energies when comparing configurations having a different number of bonds. There are several ways of dealing with BSSE, the most popular strategy is the counterpoise method; another possibility is to simply add more and more basis functions. On the other side, PW calculations are BSSE free because they are spatially organized in a lattice independently from the ion positions.^227^

#### 2.1.10 Solvent Description

Structural and electronic properties can be affected by the solvating environment. To include solvent effects in QM calculations of molecular properties or spectra, there are many methods and philosophies.

Schematically, the solvent can be treated either explicitly, by surrounding the solute with one or more layers of the solvent molecules surrounding the system of interest, or by using an implicit solvent model (ISM), representing the solvent as a continuum medium (as opposed to an ensemble of discrete molecules) and describing its effect analytically. Various explicit and implicit methods have been developed. In explicit solvent approaches, both the solute and the solvent can be modeled at the QM level, or some hybrid scheme combining various QM methods and MM methods can be used, with the solvent molecules usually treated at a lower level of theory (see section 2.2.2.5 for QM/MM methods). Since the ensemble of solvent molecules can adopt a large number of intermolecular configurations even around a very simple solute,^228^ it is necessary to calculate the property of interest considering a proper number of solvent configurations. A relatively affordable option is to choose these configurations from classical MD simulations, calculate the property of interest for each chosen configuration (with a suitable number of solvent molecules surrounding the solvent), and then to take the average over the all configurations considered. A much affordable alternative is to use an implicit self-consistent reaction field model (SCRF),^229^ where solvation effects come from a continuum representation of the solvent which “reacts” to the electron distribution of the molecule. This type of ISM consists of polar and nonpolar contributions. The nonpolar part consists of cavity and dispersion contributions. The polar part can be accounted for by different models, such as the polarizable continuum models (PCM) from the Tomasi group,^230^ or the conductor-like boundary condition screening models COSMO/COSMO-RS,^231^ or the solute electron density (SMD).^232^ In these models, a cavity is created following the shape of the solute molecule. A nonpolar contribution consists of cavity and dispersion parts. Different methods are used to calculate the cavity based on the van der Waals radii of the solute atoms or solvent accessible surface. The solute is inserted in the cavity while the solvent surrounding consists of a continuum medium interacting with charges, the solute gives rise to, placed on the surface of the cavity. The area of solvation models is wide and fast developing, and the readers are referred to excellent reviews.^233−235^ As indicated by the data reported in Table 1 and Table 2 and as further discussed in section 3.5, the treatment of the solvent in the computational studies of CND models has been so far mostly limited to ISM models. In few cases, the effect of the solvent on the conformational behavior of the CND, and thus (indirectly) on the dependent properties, has been studied using an explicit solvent in the conformational sampling, followed by the calculations of optical properties by QM methods; at this level, the solvent was however treated implicitly. Only very recently, the effect of the environment on the optical properties of a CND model has been studied explicitly including the media surrounding the fluorophore in the QM calculation by using a QM/MM approach.^73^ As expected with H-bond forming molecules, the effect of the aqueous solvent on the absorption and emission energy varies largely with the particular configuration, and averaged values should be considered. The explicit inclusion of the surrounding media leads to accurate PL property prediction.

#### 2.1.11 Benchmarking Excited States

TDDFT is currently the most common choice to deal with the excited states in quantum chemistry. However, as soon as the size of the system increases, the computational cost also increases, and some compromises are to be made in choosing the basis set or select the pseudopotential. The latter is also relevant when considering the interactions among molecules while forming aggregates or those among π-conjugated systems. Another important issue is whether the considered system presents an open-shell electronic structure, with the description of the molecular orbitals and calculation of the system energy levels a cumbersome computational problem. The typical example is polyaromatic hydrocarbons (PAHs), where the straightforward application of the TDDFT method with the most popular B3LYP-based pseudopotential can lead to a noncorrect ordering of the bright and dark states, at least for pyrene-based compounds.^236^ The cited example is very relevant in the context of the present review. Indeed, larger PAHs are exploited as models of graphitic regions in CNDs and are also considered as possible emitting centers in CNDs. It was reported, for example, that consideration of a combination of PAHs and their possible aggregates are able to explain their peculiar excitation-dependent emission feature .^237^ Benchmarking of different basis sets and DFT and TDDFT methods was carried out on a single layer of 168 conjugated C atoms.^36^ With a 6-31G(d) basis set, the geometries of ground and first excited states and the absorption transitions were calculated with different functionals, such as hybrid GGA ones (B3LYP and X3LYP), M06-2X meta-GGA hybrid functional, long-range corrected CAM-B3LYP, and nonhybrid local functional (M06-L). All the tested functionals gave the same results concerning the ordering of the electronic levels. On the other hand, the estimated energy gap was found largely dependent on the applied level of theory, with the best performance in mimicking the experimental data^238,239^ achieved with B3LYP. A further work of benchmarking on different functionals in DFT and TDDFT calculations was reported by Zhao et al.,^26^ that considered the absorption and emission features of large single layer graphene systems. The tested functionals included pure and hybrid GGA functionals (PBE, PBE0, B3LYP, and TPSSh) and long-range corrected functionals (CAM-B3LYP and LC-ωPBE) with a fixed 6-31G(d) basis set. The effect of increasing the size from 132 to 168 and 170 conjugated C atoms was already experimentally observed,^238,239^ and edge functionalization with OH groups was also considered. The agreement between experimental and theoretically calculated spectroscopic features indicated the B3LYP functional as the best performing, supporting its large application at least for a large single layer of graphene. This result indicates that the inclusion of a dispersion term not necessarily leads to the improvement of the predicted properties and confirm that the benchmarking is necessary to verify the quality of the results. A comparison of different quantum calculations to evaluate spectroscopic features of amorphous CNDs was proposed by Margraf and co-workers.^79^ After sampling using Monte Carlo simulations among different geometries with 128 C atoms, the optical properties of the nanoparticles were calculated with semiempirical techniques and TDDFT methods. In the first case, AM1-(UNO)-CIS and INDO/S were applied for larger dots, and in the second, the O-LYP functional and 6-31G(d) basis set with and without long-range corrections was considered, just for the smallest CNDs. Semiempirical techniques tend to underestimate vertical excitation energies, leading to absorption bandgaps in the optical and infrared region of the spectrum, with a large size-dependence for the smaller dots. TDDFT calculations with long-range corrections allowed avoiding the well-known drawback in computing charge transfer excitations, thus providing reliable results at least for small systems and suggesting that semiempirical techniques may underestimate energy gaps up to 1 eV.

**Figure 2:**
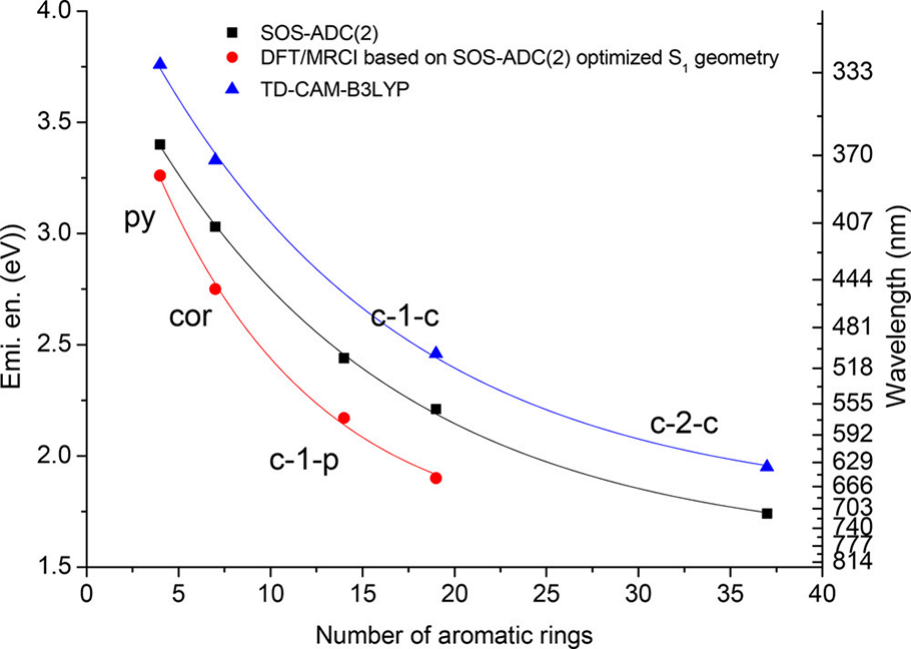
Emission energies vs number of aromatic rings. Reproduced with permission from ref (114). Copyright 2019 American Chemical Society.

With reference to PAHs systems, an extended work of benchmarking was recently performed (see Table 3 and refs (112−114, 160, 236, and 240)) aiming to compare the performance of single reference methods, such as scaled opposite-spin-algebraic diagrammatic construction to second-order [SOS-ADC(2)], time-dependent (TD)-B3LYP, and TD-Coulomb-attenuating method (CAM)-B3LYP, to multireference methods, as DFT/MRCI,^150^ n-electron valence state perturbation theory to second order (NEVPT2),^241,242^ and spectroscopy oriented configuration interaction (SORCI).^243^ The study was performed on “circular” PAHs, like pyrene, coronene and their extended models, and linear systems as tetracene molecules, also considering their aggregates. Relevant doping atoms, such as nitrogen or fluorine, were also included. In the whole set of studied systems, DFT/MRCI was shown to be the winner, performing better than the other methods in reproducing the character and the position of the excited states. For this reason, DFT/MRCI was applied as reference when experimental data were not available and to study how the electronic properties scale with the size of the PAH system or in exploring the configurational space of aggregates. An example of the results obtained in the benchmark work on the emission properties of different PAH model is reported in Figure 2.

All multireference methods agree quite well, showing that at least two configurations are required to correctly characterize most of the lower electronic states. In general, more configurations are required for the higher states. Among single reference methods, the best one for small PAHs systems was SOS-ADC(2), but even the popular DFT/B3LYP, despite wrong ordering of the first bright and dark states in the case of pyrene, did perform quite well. DFT/CAM-B3LYP was the worst method for small systems but performed reasonably well at an acceptable computational cost when increasing the size, whereas DFT/B3LYP strongly underestimates the excitation energy.^236^ Also, in the case of PAH dimers, the multireference DFT/MRCI method performed very well, retrieving the experimental optical features in the case of pyrene and coronene. Single-reference SOS-ADC(2) and TD-CAM-B3LYP methods provide reasonably good results, although with wrong ordering states.^114,160^ The performances of different methods were also tested on doped models. In the case of nitrogen doping in pyrene, single reference methods describe reasonably well the system but fail in describing the higher excited states because of their multireference character, thus suggesting a possible bias in the evaluation of internal conversion processes.^240^ Similar results were obtained for fluorine doping where the SOS-ADC(2) method performed better than CAM-B3LYP because of a better compromise in the description of charge transfer and π–π* states. In addition, the method overperforms DFT/MRCI in providing analytic energy gradients but fails in describing open shell ground situations and doubly excited states, where DFT/MRCI was still best. In the case of excitonic and charge transfer states in aggregates of tetracene, the benchmarking was extended to include long-range corrected (LC) time-dependent second-order density functional tight-binding (DFTB2).^244,245^ All the methods described well the ground state of the monomer and aggregated systems and, despite of some state-order inversions, also the excited states agree with the description of spectral shape, state character, and potential energy curves obtained with DFT/MRCI. These results suggest that the single reference and tight binding methods can describe well linear PAHs and their aggregates. However, DFT/MRCI still remains the reference method to achieve correct ordering of electronic states and to predict state character, as for excimer S_1_ state of tetracene dimer.^112^

It is worth noting that in their benchmark studies, the group of Lischka also underlined that the multireference calculations produce good results but at a high computational cost.^113,114,160,236,240^ These results were also reported in a very recent review of Otyepka and co-workers, on computational approaches to understand photoluminescence of CDs.^23^ Among the conclusions, the authors underlined the need for close collaboration between experimentalists and theoreticians to develop a multiscale cost-affordable approach to explain and predict the structural and optical properties of CNDs, a statement that also inspires the present review.

### 2.2 Force Field-Based Methods

In many cases, the size of the system to be simulated, or of its configurational or conformational space, requires the use of an approximation beyond the quantum mechanical calculations. One is to abandon the electronic DoF. Of course, for CNDs and related quantum nanoparticles, the electrons are highly important as their transitions give the photoluminescence spectrum. However, in many applications of CNDs, we also need to know how they interact with their environment and large biomolecular systems, such as lipid membranes, proteins, and nucleic acids. To do this, we use computational methods entirely based on classical physics, for example, when any statistically relevant sampling of the conformational space of explicit solvent molecules or large flexible biomolecules is not amenable with even the cheapest QM methods. In this case, we use empirical and conceptually simple FFs where inter- and intramolecular interactions are assumed to be additive contributions to the total energy. FFs do not give absolute energies as there is no common energy reference state for all the different terms and many of the potential functions are more like penalty functions of harmonic wells. Classical conventional atomistic MD simulations use FFs from which the forces to move molecules are taken as negative spatial derivatives. Other particle-based simulation methods such as Monte Carlo and dissipative particle dynamics, as examples, can also use force fields but most often more specific interaction potentials are used as energy terms. Again, as with QM methods, there is a space/time limit when atomistic simulation methods become too expensive computationally. Then we go over to mesoscale methods. The reduction of the number of DoF is among the most common strategies used to reduce computing time in mesoscale particle-based methods. This is typically realized by reducing the number of particles to be considered by grouping/combining them into larger, “coarser” particles and by modeling their interactions using simpler and softer energy terms. These latter models, also known as coarse-grain models, are caricatures of molecules and molecular systems and allow modeling of very large nanostructures on the mesoscale up to micrometers. We will now discuss the classical methods starting by atomistic molecular mechanical force-field methods going successively to coarse-grain models and methods. All are highly relevant in CND studies.

#### 2.2.1 Fundamental Concepts of Force Fields

In classical molecular models, the nuclei and electrons are merged into larger entities called atoms. While apparently simple, the concept of atoms is not so trivial when the atoms are part of molecules, not to mention artificial entities, e.g., “united atoms” or “coarse-grained beads”.

To simulate molecular systems and processes without electronic DoF, the more than 5-decades-old FF modeling concept is still commonly used. The FF is a manmade molecular mechanical idea of how atoms interact through space and along covalent bonds. It expresses the interaction energy as a function of geometrical parameters, and it is parametrized using data from QM calculations and experiments. To illustrate how an atomistic FF is constructed, consider two atoms approaching each other. QM calculations can be used to obtain attraction energies as a function of the distance, *r*, separating the atoms and the results fitted to a curve where the attraction follows a physical power law ∼1/*r*^6^; the repulsion at small *r* is found to decay exponentially with increasing *r*. This generally applies to noncovalent interactions, where valence electrons of the two atoms are *not* shared (i.e., not bonded). If electrons are shared, covalent bonds are formed, and the interaction around the equilibrium bond length can be conveniently modeled by a harmonic potential, which well describes the vibrational population at normal temperatures and pressures. At conditions beyond normal, we need an anharmonic potential with a steeper repulsive wall at short distances, and a softer wall at larger *r*, leading to a plateau, thereby allowing for dissociation (bond breaking). Standard all-atom FFs do not allow dissociation as they use harmonic potentials with infinite walls. The two parameters for covalent bonds, the bond length, and the force constant, can be obtained by fitting the results from QM calculations. Together, this information gives us a pairwise potential energy function *U*(*r*_*ij*_) between two nonbonded particles *i* and *j*, combining the so-called van der Waals attraction (negative term) and Pauli repulsion (positive term):
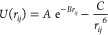
22where *A*, *B*, and *C* are adjustable constants. There is a mathematically and computationally simpler form of eq 22 known as the “Lennard–Jones (L–J) 12–6” pair potential, where the exponential term is replaced with a ∼ 1/*r*^12^ power term, chosen for its numerical efficiency,^246^ describes closely enough the exponential soft repulsive wall, not too far from the equilibrium distance of two nonbonded atoms:
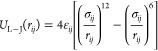
23

This potential has two parameters: σ_*ij*_, the collision distance, and ε_*ij*_, the depth of the potential well. If the particles are charged, they are assumed to interact according to the Coulomb law:
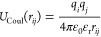
24where *q*_*i*_ and *q*_*j*_ are the partial charges of particles *i* and *j*, ε_0_ is the vacuum permittivity (4π is a constant needed for SI units), while ε_r_ is the relative permittivity or dielectric which depends on the dielectric medium. It is set to 1 when an explicit solvent model is used in the simulation, while in the case of implicit solvent it is set to the temperature-dependent dielectric constant of the solvent medium at the simulation temperature. For example, in primitive electrolyte model for aqueous ionic solutions ε_r_ = 78 can be used for implicit water at room temperature to effectively screen the Coulombic interactions between charged particles. As the bulk dielectric constant model is not valid at surfaces or in solvation layers close to a solute, many alternative approaches have been developed, from simple distance-dependent dielectric constants to the advanced MM-PBGB and MM-PBSA methods. For details and evaluations of these methods, the reader is referred to refs (247 and 248).

The atomic charge *q* is not a quantum mechanical observable, which means that there is no unique way to obtain it. It can be determined using different schemes, from Gasteiger’s classical,^249^ to Mulliken and other related population analysis of electron density,^250^ or by fitting the electrostatic potential (ESP) to the atoms,^251^ of which we mention the most commonly used restrained ESP scheme.^252,253^

When using eq 23 for a pair of unlike atoms, the ε_*ij*_ and σ_*ij*_ cross parameters are regularly determined by using combining rules. The choice of combining rules, an issue sometimes ignored in the molecular simulation community, is highly important in chemical engineering using equation-of-state based models.^254,255^ The most commonly used combining rules are the geometric mean (Berthelot rule) for ε_*ij*_, and the arithmetic mean (Lorentz rule) for σ_*ij*_:
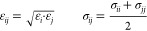
25

The Berthelot rule has been shown to overestimate the potential well depth.^256^ Alternative mixing and combining rules do exist and are frequently used in engineering modeling. The are many alternatives to the L–J 12–6–1 potential, which we will not discuss here, but we would like to mention the so-called bond-order potentials,^257^ as they are used in the CND modeling community.^258^

**Figure 3:**
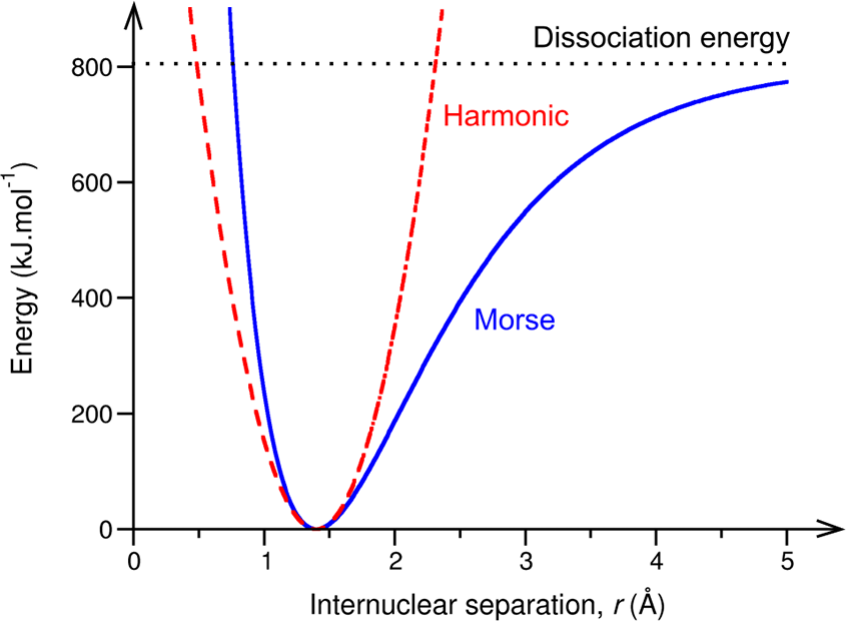
Harmonic potential (dashed red line) and anharmonic Morse potential (solid blue line) superimposed. The corresponding experimental bond dissociation energy is shown by the horizontal dotted black line.

In nearly all FFs, the stretching and compressing motion of covalent bonds is described with Hooke’s law, which gives a harmonic symmetric potential energy (see Figure 3):
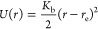
26where *K*_b_ is the force (spring) constant and *r*_e_ is the equilibrium bond length. In eq 26, *K*_b_ is the main adjustable parameter, increasing in magnitude from single to triple bonds, resulting in a stiffer spring connecting the masses. The other adjustable parameter, *r*_e_, can be obtained from QM calculations or spectroscopic studies. As can be seen in Figure 3, near the equilibrium distance, the harmonic potential overlaps well with the more realistic (and computationally much more expensive) Morse potential.

A similar harmonic potential is also used to give the potential energy for angle bending (not shown), with an adjustable force constant for the angle bending motion around an equilibrium angle. A further “bonded” term is the rotation around the bonds by considering the torsional angle of a consecutive A–B–C–D sequence of bonded atoms in a molecule, measured as the (dihedral) angle ϕ between the A–B–C and D–C–B planes. The corresponding potential energy is a periodic function and can be expressed as
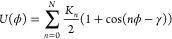
27where *K_n_* is an adjustable force constant that can be related to the barrier height (hindrance of the rotation) if there is only one term in the sum; *n* is the multiplicity (number of energy minima in every 360° rotation of a plane), while γ is the phase factor and specifies the minimum energy. When four bonded atoms are to be kept in the same plane, the so-called improper torsion term is used:
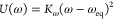
28where ω is an out-of-plane angle of one atom deviating from the plane occupied by all four atoms in their equilibrium structure, identified by ω_eq_. This potential term is important, for example, for conjugated rings and multiple ring systems (like graphene).

A general FF can be expressed as follows by adding up the potential functions described above:
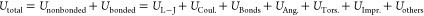
29

All the contributions in the typical FF are assumed to be additive functions. This arbitrary division is conceptually simple and appealing, making the FFs robust, and all terms have very simple mathematical functions with very few adjustable parameters. This form of a sum of simple additive functions was already proposed in 1966 by Bixon and Lifson^259^ in their studies of cycloalkanes, while many of the terms were already proposed decades earlier. Also, there is colossal amount of early work done by Allinger.^260^ The computational cost in using eq 29 increases linearly with the number of atoms (*N*) in the system for the bonded interactions, while nonbonded interactions have quadratic (*N*^2^) dependence and are thus computationally much more expensive.

There exists no single method or rule to parametrize and optimize the FFs. It is mainly done by combining data from QM calculations and all available experimental data. Sometimes educated guesses can contribute too. There are many families of FFs available, either developed tightly together with a particular simulation software, or independently. They all have roots in the same family tree going back to work done at the Weizmann Institute by Lifson and co-workers.^261,262^ Users are referred to the review of Allinger for details of the early evolution of MM force fields.^260^ Nevertheless, different FFs have different parametrization strategies (level of theory, where QM calculations are involved, or choice of experimental target properties, etc.). To calculate new parameters for a force field, it is also important to follow the original philosophy used to create the FF. How good a particular FF is can only be determined by using it, and if it provides consistent results for several properties in agreement with experiments, it is of good quality. The simulation results are not expected to agree perfectly with experiment, as FFs are very simple empirical products but also because simulations do not always fully correspond to the experimental conditions. However, they often reproduce trends very accurately, provided an appropriate FF is chosen.

The potentials discussed above are of rigid-charge type, i.e., the partial atomic charges remain fixed at the atomic centers. Such fixed charges provide effectively some polarization effects to the molecules and, although simple, their benefit is that they are reasonably transferable from one system to another, depending on the data used in the parametrization. Still, all FFs should always be tested and validated before starting to use them on a new system. If the FF is flexible enough and gives good results compared to several independent experiments, it does not matter how the FF looks like and what it contains as details. It is the results it produces that matters. Popular FFs for small molecules were recently reviewed by Lin and Mackerell,^263^ providing a detailed description of different strategies to develop FFs.

Methods based on FFs have been used to study the structure and dynamics of various CD types in solution as well as their interactions with biological macromolecules, e.g., proteins and nucleic acids. Selected examples of the use of FFs in studies of CDs are presented below, serving as a brief overview of the applicability of this method family and as an indication of the specific FFs used in different type of investigations; these and other studies are described in greater detail in section 3. The FF parameters describing the CD are often taken or adapted from an established FF, e.g., CHARMM,^264,265^ OPLS,^266^ or AMBER,^267,268^ especially when they are used to model the CDs interacting with biological macromolecules; alternatively, special CD parameters may be developed. Elvati et al.,^269^ for example, used the CHARMM General Force Field (CGenFF)^270^ to study the effect of size and edge functionalization of GQDs on their aggregation in water, while Paloncýová et al.^271^ studied the stability, structure, and internal dynamics of spherical, multilayer CDs using an OPLS-AA-based FF.^266,272^ In fact, Otyepka and co-workers also studied the self-assembly of such multilayer CDs from graphene layers (GQDs) in the presence of a molecular fluorophore;^55,95^ CDs were modeled by the AMBER ff99^273^ FF (with refined parameters)^272^ and the fluorophore molecules by General AMBER Force Field (GAFF).^274^

Concerning the interactions of CDs with biological macromolecules, FFs based methods have been applied to study the adsorption of CDs to proteins and DNA fragments. For example, Yang et al.^275^ recently reported AMBER-based^273^ MD simulations (*vide infra*) aimed at understanding the mechanisms by which CDs inhibit human insulin fibrillation. Martín et al.^276^ used the OPLS-AA FF^266,271^ to study interactions between model GQDs and human peroxidase enzymes, as relevant to understanding their biodegradation, while Liang and co-workers^277^ studied the effect of GQD size on the adsorption and structure of the HP35 model protein as part of their assessment of GQD cytotoxicity;^278^ GQDs were described by specially developed parameters and the protein by the CHARMM FF.^265^ Finally, FFs have also been used to study the interactions between various CDs and lipid bilayers, as highly relevant to understanding their cellular internalization mechanisms. A number of these studies^279−281^ employed previously described parameter sets for graphene^282^ to model GQDs, with lipid bilayers described by the CHARMM36 FF;^283^ others^63,284,285^ made use of the CGenFF/CHARMM36,^270,283^ GROMOS,^286^ or OPLS-AA FFs.^266^

We should mention a valence force field for layered double hydroxide materials called LDHFF.^287^ It is based on the family of consistent force fields from the group of Hagler.^288^ LDHFF has a double-well potential to describe the oxygen–metal–oxygen bending. All other intramolecular terms including cross terms are fine-tuned using DFT calculations. Liu et al.^289^ did use LDHFF in their work of N-CD intercalated LDH composites to simulate the 2D structure of ultrathin N-doped carbon dots. They could regulate the photoluminescence quantum yield (QY) based on the amount of N, where MD simulations were used to study the doped CD structures.

In Table 4 are summarized the relevant information concerning the methods used for studying CNDs and their interaction with other molecules.

**Table 4:** Overview of Classical Simulation Methods Used to Study the Interactions of CDs

method family	CD FF	environment FF^a^	computed data^a^	system	refs
AA MD	OPLS-AA	TIP3P, DMF (OPLS-AA)	structural analysis (including H-bonding, layer separation) and stability of CNDs; average rotation time of layers	spherical graphene multilayer model with O-containing surface functional groups	Paloncýová et al.^271^
		water	identification of interaction sites of GQDs on peroxidases; aggregation of GQDs; interaction energies between GQD and enzyme; RMSD of enzyme	single- and double-graphene layer	Martín et al.^276^
		TIP3P	structure and dynamics of LB permeation of CNDs: H-bond analysis, bilayer structure, various diffusion coefficients; free energy profile of CND translocation	spherical graphene multilayer model functionalized with −OH	Erimban et al.^285^
	OPLS-AA	SPC	mainly analysis of protein structure and dynamics: structural deviations, radius of gyration, secondary structure, interacting amino acids (with CND)	polymeric model (partial)	Maity et al.^290^
	CHARMM CGenFF	TIP3P	structural analysis of GQD aggregation: relative orientations, interlayer separation, H-bonding (to some extent)	single-layer graphene; stacking observed	Elvati et al.^269^
		TIP3P, POPC LB (CHARMM 36)	extensive structural, dynamic analysis of LB; dynamics (average time of permeation) of GQDs	single-layer curved graphene: −OH and cysteine-terminated (edge functionalized)	Liu et al.^284^
	adapted from Titov et al.,^291^ CHARMM CGenFF for edge functional groups	SPC/E, POPE/POPG 3:1 LB described by Berger lipid FF^292^	mechanism of GQD detachment from LB	single-layer rectangular graphene layer with −OH, −SO_3_^–^, −NHNH_3_^+^ functionalization	Yao et al.^293^
	CHARMM, CDOCKER protocol^294^	water; DNA fragment (CHARMM)	interaction configurations with DNA	single-layer graphene; single amino-, hydroxyl-, carbonyl-, and carboxyl-functionalized edge sites.	Xu et al.^295^
	CHARMM	SPC/E	GQD/drug–lipid interaction energies, free energy profiles of translocation; structural analyses.	single-layer hexagonal graphene	Xue et al.^296^
		water; PSMα1 peptide dimer (CHARMM)	fffect of GQD on PSMα1 monomer assembly, distance between monomers monitored; changes in secondary structure.	single-layer hexagonal graphene with −COOH functionalization	Wang et al.^297^
		TIP3P	structural and energetic analysis of ss-DNA adsorption on GQDs: contact area and vdW attraction force	single-layer square graphene, functionalized with −OH, −CO, −COOH	Jeong et al.^298^
	GAFF	TIP3P	ligand binding energies (docking)	N-doped graphene layer	Ghadari^31^
		TIP3P	solution behavior, self-association and conformational distributions	IPCA	Siddique et al.^55^
	AMBER 99ff, GAFF	SPC/E	self-association and interactions of IPCA with CNDs	IPCA, PAHs ,and CNDs with −OH edge functional groups	Langer et al.^95^
	GROMOS 54A7	TIP3P, DPPC LB and gemcitabine drug molecules (GROMOS 54A7)	GQD–drug interaction energies, force-displacement profiles from steered MD (pulling structures across LB), GQD-lipid headgroup interaction energies.	N-doped single-layer hexagonal graphene	Vatanparast et al.^63^
	user-defined	TIP3P	hydration shell structure (RDFs).	single-layer rectangular graphene	Dalosto et al.^299^
	Universal Force Field, Forcite Plus code^300^	SPC/E, double-stranded DNA fragment	MSD, translational diffusion coefficients, interaction modes and energies.	single-layer graphene, pristine and partially surface oxidized (epoxy-groups)	Wang et al.^301^
	COMPASS	water	sampling the conformational space accessible to the CND, use of the sampled configurations to calculate optical properties at the UNO-CIS level.	bilayer of an amide-capped graphene layer	Strauss et al.^67^
	adapted from Cohen-Tanugi and Grossman^282^	TIP3P, POPC LB (CHARMM36)	structure and dynamics of GQDs interacting with LBs; orientation of bilayer-permeating GQDs; GQD self-association in general sense; PMF associated with bilayer permeation	single-layer, pristine, circular graphene	Liang et al.^280^
		TIP3P, protein villin headpiece (HP35) (CHARMM27)	HP35-GQD adsorption structure (distance), protein RMSD/RMSF	single-layer pristine hexagonal/circular graphene layer	Zhou et al.^277^
		TIP3P, POPC LB (CHARMM36)	structural analysis, PMF, and hydration free energy calculations	single-layer circular graphene layer with modifiable edge atomic partial charges	Tang et al.^281^
		TIP3P, Poly(A-T)_20_ and poly(G-C)_20_ (Amber03) DNA fragments	identification of GQD adsorption sites on DNA fragments, interacting nucleotide bases; GQD aggregation (qualitative)	pristine circular single-layer graphene layer	Kong et al.^302^
	adapted from Tu et al.^303^	TIP3P, ubiquitin (CHARMM)	structure (amino acid residue composition) of ubiquitin-GQD adsorption interface	pristine graphene single layer (infinite)	Fang et al.^304^
					
	not specified	TIP3P, POPC LB (CHARMM36)	energy barriers associated with initial piercing of GQD into bilayer	single and multilayer graphene pristine and functionalized	Li et al.^279^
CG MD	Martini 2.0	Martini P4-type CG water bead, POPC LB (Martini)	graphene binding (extraction) energy inside bilayer, bilayer thickness	single- and multilayer rectangular graphene sandwiched inside a POPC LB	Titov et al.^291^
	Martini	CG explicit (Martini), POPC LB (Martini)	interaction modes and energies; PMF associated with GQD-bilayer translocation	rectangular graphene layer: pristine, partially edge-oxidized, and stacked (multilayer structures)	Wang et al.^305^
DPD	CG (user-defined)	CG explicit, LB	dynamics of GQD translocation into LB, specifically identification of preferred orientations.	single and multilayer graphene layer of various shapes	Li et al.^279^
	CG (user-defined)	not specified, LB	graphene sheet translocation mechanism: coordination numbers and bilayer thickness monitored; bilayer bending energy	single-layer rectangular graphene: pristine, partially edge- and basal plane-oxidized	Mao et al.^306^
	CG (user-defined)	CG explicit, phosphor-LB	entry/adsorption modes and size dependence of GQDs into LB	single-layer pristine hexagonal graphene	Dallavalle et al.^307^
	CG (user-defined)	CG single bead water, LB	translational diffusion patterns, structure, and interaction energies	single-layer rectangular graphene	Chen et al.^308^

a The following acronyms appear in the “environment FF” and “computed data” columns: LB stands for lipid bilayer; PMF stands for potential of mean force.

##### 2.2.1.1 Polarizable Force Fields

There is a category of intermolecular potentials that explicitly account for the polarization effects in condensed phases. The π–π stacking interactions for carbon rings, for example, are better described by such polarizable FFs^309^ than by the L–J 12–6–1 rigid-charge model described in section 2.2.1. Polarizable and other advanced water models were recently discussed by Ouyang and Bettens^310^ and by Demerdash et al.^311^ Polarizable FFs in general, for a variety of molecular systems, were discussed by Halgren and Damm,^312^ Soloviev et al.,^313^ and Shi et al.^314^ Many different polarizable models have been developed for water, based on different strategies, ranging from moving point charges to atomic multipoles. One of the more established polarizable FFs is the atomic multipole optimized energetic for bimolecular applications (AMOEBA), which has been extended to carbon-based materials^315^ and proteins.^316^

##### 2.2.1.2 Reactive Force Fields

There are classical FFs which allow covalent bonds to form and break by mimicking some principles of QM, such as the reactive FF, called ReaxFF, of van Duin and co-workers^317,318^ that has a well-established position in the simulation community. Differently to classical atomistic force-fields that are intrinsically unable to model changes in atom connectivity, ReaxFF describes reactive events by making use of a bond-order formalism which depends on the interatomic distances. ReaxFF has been continuously generalized and can be used in many applications ranging from nanoparticles and combustion studies to aqueous solutions and biological systems. Its parametrization for each specific system is, however, a major undertaking. The overall interaction energy of the standard ReaxFF is composed of the following nine terms:
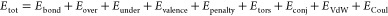
30These are (i) bond energy, (ii) penalty due to overcoordinated atoms, (iii) energy of under-coordinated atoms, (iv) valence angle contribution, (v) penalty due to two double bonds sharing a valence angle atom, (vi) torsional angle energy, (vii) conjugation effect contribution, (viii) van der Waals interactions, and (ix) Coulombic energy. The first term, *E*_bond_, is given as the Morse potential. For the functional form of the remaining terms in eq 30, the reader is referred to the original paper of Van Duin et al.;^317^ an example of how to parametrize ReaxFF for carbon materials can be found in work of Srinivasan et al.^319^ where a novel parametrization has been developed to study the microstructural evolution of large fullerene molecules (C_180_) at high temperature. The starting point of this novel ReaxFF potential is the ReaxFFCHO, which has been previously parametrized against an extensive training set consisting of atomic charges, bond lengths, bond, valence and torsion angle energies, heats of formation, and various hydrocarbon reaction energies. Starting from the ReaxFFCHO functional form, the authors developed a novel ReaxFFC-2013 potential aimed at the description of the dynamics of condensed phases of carbon. The first preliminary results demonstrated that the ReaxFFC-2013 potential accurately predicts the atomization energy of graphite as well as the barrier for transition from graphite to diamond.

Among its applications in the CND field, it is worth mentioning the study performed by Gu et al.^320^ to verify possible atomic configurations of nitrogen and oxygen doped graphene, obtained by solid-phase microwave-assisted (SPMA) pyrolysis of CA and urea. This study is described in greater detail in section 3.2. Although their use in CND simulation is still limited, it is expected to be useful in understanding their structural decomposition and stability.

##### 2.2.1.3 Coarse-Grained Force Fields

The FFs described in the preceding sections represent each atom of the model system as a single classical particle, an approximation that allows for the simulation of relatively large systems, e.g., representing bulk liquids and solutions, biological macromolecules, and membranes. Some problems may require very much larger models, e.g., consisting of millions of atoms, to be simulated for hundreds of nanoseconds to microseconds, often presenting a prohibitive computational load for atomistic (also referred to as “all-atom”, or “AA”) FFs. Additional model simplifications are required in such cases. The united atom (UA)^321,322^ and coarse-grained (CG)^323,324^ FFs mentioned in section 2.2.1 represent selected groups of atoms by single classical particles, reducing the number of particles to be considered. The CG modeling approach, in particular, has become increasingly popular for large-scale computer simulations of diverse systems, including graphene-based NPs in biological environments.^291,306^

While conceptually intuitive, the development of suitable CG interaction potentials is not trivial. In fact, there exists two fundamentally different philosophies for deriving CG potentials: the “top-down” and “bottom-up” methods.^324^ In the former, the CG interactions potentials are chosen in order to reproduce target thermodynamic properties or structural features. The resulting CG FFs often have familiar functional forms, similar to those of AA FFs, consisting of simple potential expressions and a few adjustable parameters; the Martini biomolecular FF is a pertinent example.^325^ The bottom-up methods, on the other hand, essentially use a suitable high-resolution model of the system, e.g., an AA FF or QM description, as basis for the CG potential development effort.^326,327^

CG FFs have been employed rather extensively for computational studies of the interactions of CDs with lipid bilayer membranes, as translocations of CDs across cell membranes are frequently a key step for potential biomedical applications. Such studies also often require very large computational models simulated for tens to hundreds of nanoseconds; this is where CG modeling offers advantages over more conventional FFs. The Martini CG FF was employed by Titov et al.^291^ to study the stability and formation by self-insertion of graphene sheets imbedded inside the hydrophobic interior of a model lipid membrane, so-called “sandwiched super-structures”, including stacked graphene multilayer structures. Wang et al.^305^ also used the Martini FF to perform MD simulations of graphene sheet–lipid bilayer interactions, focusing on the effect of CD particle thickness (i.e., multilayer particles), the degree of graphene oxidation, as well as the precoating with lipid molecules (with the graphene sheet/CD encapsulated by a layer of adsorbed lipid molecules) on the membrane translocation process.

CG models have also been employed within the Dissipative Particle Dynamics framework (section 2.2.2.6) to perform large-scale simulations of CD–lipid bilayer interactions.

##### 2.2.1.4 Machine Learning Force Fields

The use of artificial intelligence (AI) in the construction of FFs has emerged rapidly in the last years. ML, basically a multitude of methods in-between AI and numerical optimization and regression, will have an important impact in the development of the next generation of FFs. ML-based FFs (MLFF) are expected to provide a complex multidimensional energy (and force) landscape at an accuracy of *ab initio* QM calculations but are computationally very much cheaper than first-principles QM simulations, so that they can be applied to large systems.

FFs are still very much faster than MLFFs to use, and they are also transferable, at least within the same types of molecules. MLFFs are highly accurate, offering many new possibilities to study compared to FFs. Unlike FFs, MLFFs do not contain any physical models, except those used in the underlying QM calculations. Therefore, the MLFF should be carefully validated before using them. Nearly all work so far has been about studying bulk systems, and the largest molecular systems where high-end QM MLFFs were applied still consist of only a few dozen of atoms. We will have to wait for MLFFs for complex and large molecular systems such as proteins and other biomolecules until it becomes feasible to compute the large data needed to construct MLFFs. Most likely we will have hybrids between FFs and MLFFs. Coarse-grained MLFF potentials can be the solution as atomistic DoFs in large biomolecular systems are often less important. Besides MLFFs, ML methods will be highly valuable in analyzing the simulation results.

While the use of MLFFs is yet to develop in the field of the carbon nanoparticles, we mention the work of Rowe et al.^328^ to construct an MLFF for graphene and carbon materials which is based on using the Gaussian approximation potential (GAP) from underlying DFT calculations. They evaluate their MLFF with other frequently used empirical and bond-order interaction potentials and compare the results with experimental and *ab initio* data at very different physical conditions. Although computationally more demanding compared to empirical potentials, their MLFF is 4 orders of magnitude faster than AIMD. Rowe et al. later succeeded in making their MLFF for carbon materials transferable, which MLFFs in general are not, and applied it to different phases of carbon as well as to crystal surfaces and defect structures.^329^ It should be applicable to a broad range of applications using various forms of carbon, from bulk to nanostructures, including carbon and graphene dots.

An interesting recent attempt to model the CND structure was carried out by Deringer and Csanyi exploiting new potentials produced with the ML algorithm. Standard model potentials developed for carbon such as Tersoff, Brenner, and EDIP show serious drawbacks such as the underestimated concentration of sp^3^-bonded atoms in tetrahedral amorphous (ta-C) and a poor description of surface properties. In order to improve the accuracy of model potentials for the description of amorphous carbon, the authors developed a novel model potential based on a ML algorithm. The main idea of this method is to map a set of specific atomic configurations onto numerical values for energies and forces. All these quantities are “trained” from a large quantum-mechanical reference database and subsequently interpolated using the ML algorithm.

In detail, the authors^330^ developed an interatomic GAP for carbon, mainly focused on liquid and amorphous phases which is specifically “trained” from a database of reference quantum-mechanical data and is then used to interpolate energies and forces for arbitrary structures. The functional form of the present GAP is broken down into a sum of local contributions, given by a local energy function ε. The present function is generated using a kernel function which measures the similarity to a specific neighbor environment. All the structural data used for the training procedure were obtained using first-principles DFT calculations from melt-quench MD, following protocols that are well established for amorphous carbon. A typical protocol included the generation of 100 independent structures at densities of 1.5–3.5 cm, for a single snapshot from each trajectory, a single-point DFT calculation was performed, and the results were included in the next round of training. See Section 3.1.2. for further details on the assessment of the accuracy of this model potential in Section 3.1.2.

Although we have focused here on machine learning interaction potentials, we want to stress that ML is constantly finding new applications in molecular simulations.^331^ ML can be used, for example, for more effective sampling by reducing the dimensionality, mining and analyzing data from trajectories, prediction of molecular structures, and properties and mechanical characteristics and also for mapping of spectral lines, finding critical interactions, reaction pathways and rare events, deciphering complex processes, and for establishing structure–property relationships,^332^ just to give a few examples.

#### 2.2.2 Molecular Dynamics Method and Simulations

Classical Molecular Dynamics is a simulation method based on the use of molecular mechanical force fields and is widely used to study the dynamical behavior of interacting particles at different physical conditions in varying chemical environments.^333−335^ The method was outlined already in 1957,^336,337^ and the underlying physics dates back to Isaac Newton, since his laws describing the relationship between the motion of an object and the forces acting on it, can, for many purposes, be applied also to microscopic particles. After Stillinger and Rahman in 1974^338^ published the radial distribution functions (RDF) for simulated liquid water in close agreement with those obtained from X-ray diffraction studies, the full power of MD simulations was realized, and the rest is history. Today, classical MD simulations are used to calculate structural, thermodynamic, and dynamical properties of most diverse systems, from liquids and solutions to fractures and defects, from surfaces and interfaces to clusters and nanoparticles, from biological systems to drug delivery, liquid crystals, and mesoscopic soft particles, etc.

##### 2.2.2.1 Physical and Mathematical Outline of MD

In classical MD simulations, the classical equations of motions (EoM) are solved for each particle in a simulation cell. The chaotic path of each molecule, for example, in a liquid, can be followed during the entire simulation. In a simulation cell with *N* particles there is a net force (a vector) **F** acting on each atom created collectively by all the other particles in the surrounding. Force fields, described in section 2.2, allow one to calculate an instantaneous total interaction energy *U*(**r**_1_, **r**_2_, . . ., **r**_*N*_) as a function of the spatial coordinates of the *N* particles. As shown in eq 31, the force **F** at time *t* can be obtained as the negative spatial gradient of *U*, and in Newtonian dynamics, knowing the force acting on a particle and its momentum, we can calculate the acceleration by the well know relationship **F** = *m***a**, where *m* is the mass of the particle (atom or molecule) and **a** is its acceleration. Since all particles move in the simulation cell, their coordinates, energies, and forces all depend on time *t*. The time-dependent force acting on particle *i* is given as
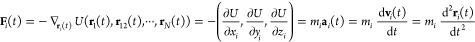
31

Molecular simulations can be considered both as theory and as a computer experiment. In contrast to real experiments, simulations allow us to follow each individual atom. From eq 31, we can see that, knowing the total interaction energy *U*, we can access, besides the acceleration (**a**_*i*_), also the velocity (**v**_*i*_) and position (**r**_*i*_) of each particle *i* as a function of time, by numerically integrating them out from the last two terms. There are many numerical integrators available to solve Newton’s EoM in MD simulations. They are based on truncated Taylor expansion of **r**_*i*_(*t*). Already, Newton was familiar with this type of algorithm.^339^ A commonly used integrator is the original Verlet algorithm,^340^ obtained by expanding the positions in both directions in time and neglecting the fourth and higher order terms. A reformulation of the Verlet algorithm called “Verlet Leapfrog” uses less storage space and increases the accuracy of the velocities. It uses half time-step values for velocities, so that they can be seen pictorially as jumping over (leap-frogging) the positions at full time steps, after which the next position can be calculated. Yet another reformulation, “Velocity Verlet” (eqs 32 and 33), is the most commonly used integrator today, and it can give both positions and velocities at same time points.
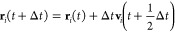
32
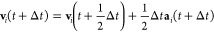
33

Knowing the velocity and position at a given time *t* and the interaction energy between the particles given by the FF, we can calculate the position and velocity a short time period Δ*t* later. In a sense, an MD simulation is a very large collection of snap shots of molecules interacting with each other. Playing the snap shots in a sequence, it becomes a movie of molecular motion, which we can also analyze statistically and calculate a broad and increasing range of properties to compare with experiments. This is the very essence of all MD simulations.

Simulations should, in principle, sample all possible parts of the phase space to give statistically reliable results, so we need to perform long enough simulations to reach this goal. As the length of the entire simulation is the number of time steps (*M*) multiplied with the time step Δ*t*, a bigger time step automatically means a longer simulation with the same amount of used computing power. However, the time step should be small enough that the forces acting on each atom can be considered constant during such time interval; thus, stretching the Δ*t* too much might produce completely unphysical results, as the particles risk penetrating each other without even colliding. The time step should be chosen based on the maximum fluctuation frequency found in the system, which is typically the stretching vibrations of light hydrogen atoms bonded to heavy atoms. This fast vibration, which should also be properly sampled, takes place in the subfemtosecond time scale. Reasonably Δ*t* should be ≪ 2*p*(*m*/*k*)^1/2^, where *m* is the mass of the lightest bonded atom and *k* is the Hooke’s force constant for the bond which we find in the FF we are using. Based on the estimate above, we should choose a time step around 0.1 fs or less. Most all-atom simulations today use 1.0–2.0 fs as Δ*t*, even with the hydrogen atoms included, although even longer time steps up to 5.0 fs are used occasionally with stable integrators. To use such long Δ*t*, special tricks are applied. As fast bond stretching oscillations require short MD time steps, freezing of such fast motions is an efficient way to increase the optimal time step. To use completely rigid molecules without any flexibility is an option for small molecules like water. Indeed, the very first water models (ST2, MCY, SPC, TIP3P, etc.) were used as rigid. For large(r) molecules, using constraints is the only option to freeze fast oscillations and allow slow amplitude motions. The first commonly used method was SHAKE,^341^ referring to the molecule being “shaken” back to its equilibrium geometry. SHAKE is an iterative method and used regularly with the Leapfrog-Verlet integrator. The corresponding iterative algorithm for the velocity-Verlet is called RATTLE.^342^ There is an analytical scheme, SETTLE, to constrain water molecules by Miyamoto and Kollman.^343^ A linear bond constraint solver (LINCS) was developed by Hess et al.,^344^ which was shown to be a factor of 4 faster than SHAKE. Also, as it is not iterative or recursive, it was easy to parallelize.^345^

##### 2.2.2.2 Periodic Boundaries Conditions and Electrostatic Interactions

Molecular computer simulations use simulation boxes typically containing 10^3^–10^5^ particles, and to avoid surface effects and represent bulk systems, periodic boundaries conditions (PBC) are typically applied. The cutoff radius is a distance for interactions in the short-range pair potentials, e.g., Lennard–Jones, after which the interactions are assumed to be zero. For Coulomb pair potentials, the long-ranged interactions do not actually decay to zero within the box dimensions but require a special treatment such as Ewald, reaction field, or a shifted cutoff,^333−335^ but a cutoff is still applied. In the case of Ewald summation, the cutoff applies to the real part of the Ewald summation, after which the calculations are done in the reciprocal space. For more details, see Allen and Tildesley.^333^ Within PBC, the cutoff radius for pairwise interactions cannot exceed half the length of the shortest box side. This restriction is a problem for Coulombic interactions with a range of several box lengths. Coulombic interactions are therefore commonly treated with the 100-year-old Ewald summation method,^346^ invented for calculation of lattice sums in crystals. The artificially crystal-like character of the simulation cells as synchronously dynamic ‘‘unit cells” allow the full application of Ewald summation.

##### 2.2.2.3 Ensembles, Thermostats, and Barostats

Originally all MD simulations were carried out in the microcanonical ensemble where the number of particles (*N*), cell volume (*V*), and energy (*E*) are all constant. In practice, a perfect conservation of *E* cannot be expected in MD simulations as it depends on the chosen physical conditions, the quality of the force field, and the numerical stability of the simulation algorithms, etc. As experiments are typically done at constant temperature and constant pressure, use of the isothermal–isobaric (NPT) ensemble in simulations is required to obtain the same condition as in experiments. The simplest technique to keep the temperature constant in MD simulations is to scale all the individual velocities of all molecules with a factor (*T*_new_/*T*)^1/2^, where *T* is the desired temperature, but this ruins both the energy conservation and the dynamics in the system. Therefore, velocity scaling should be used only in the equilibration phase, i.e., the initial part of the simulations aimed at bringing the system to a state of dynamical equilibrium, typically monitored observing whether the energy of the system and various observables (e.g., density, pressure, structural parameters, etc.) oscillate around a constant values. In general, the equilibration stage is not used for analysis, and only those portions of the MD trajectories where the system can be considered at equilibrium (production phase) are used for extracting information about the structural, dynamic, and thermodynamic parameters of the systems under investigation. In the production phase, velocity scaling should be avoided, and a thermostat providing a proper canonical ensemble should be used. There are many methods to create thermostats to control temperature from stochastic, weak/strong coupling to extended system methods. Examples of these are the thermostats of Andersen,^347^ Berendsen,^348^ and Nose-Hoover,^349,350^ and often the choice of the thermostat and barostat depends on the available options on the used software.

For an overview and details of thermostat/barostat algorithms, the reader is referred to Hünenberger.^351^ As can be seen in Table 4, most of the simulation based on atomistic force field presented in this review make use of molecular dynamics to sample the conformational space of the CDs, either in solvent or in more complex systems comprising large biomolecular systems, as already discussed at the end of section 2.2.1 and throughout section 3.

##### 2.2.2.4 Steered MD Simulations

Nonequilibrium MD simulations can be used to mimic single molecule micro/nanomechanical manipulation experiments. Grubmüller et al.^352^ were first to model atomic force microscopy (AFM) experiments in aqueous solution by pulling the strongly bound biotin ligand out of the streptavidin–biotin complex, following experiments by Lee et al.,;^353^ the simulated pulling was roughly 6 orders of magnitude faster than that in the corresponding AFM experiments. Schulten and co-workers^354,355^ proposed a new technique, Steered MD (S-MD), to better match AFM pulling experiments. S-MD is used either in a mode where a constant force is added to one or several atoms, or in another mode where a harmonic spring is attached to a dummy atom connected to the ligand molecule and pulled at a constant velocity in a specified direction(s) to follow the reaction coordinate for binding/unbinding and to obtain a free energy profile as a potential of mean force (PMF). Umbrella sampling (US) is another popular method to calculate the PMF, but it can quickly become inefficient if the number of windows is large.^356^ S-MD is a better alternative, where the system is steered along the reaction coordinate by applying harmonic potentials with large and stiff force constants. Park and Schulten^357^ later demonstrated that using large force constants in their S-MD simulations for the restraint potential, it is possible to compute the equilibrium PMF using the Jarzynski equality,^358^ no matter how fast the pulling was done. S-MD has been applied in the study of the membrane penetration of CDs, as in refs (63 and 279).

##### 2.2.2.5 Nonadiabatic Molecular Dynamics Simulations

There are simulation techniques we can use to approximately follow chemical reactions, charge transfer, excited state dynamics, and simulate absorption/emission spectra in complex and sizable molecular systems. We discuss here the QM/MM method and thereafter take up the nonadiabatic/surface-hopping dynamics. The QM/MM method was pioneered by Warshel and Levitt^359^ and developed further in Kollman’s and Karplus’ groups.^360,361^ It is a compromise between full QM calculation of a smaller system where the electronic DoF are needed and the surroundings, which can be the rest of a large molecule, solution, surface, or anything that can be built using MM models. In general, there are two main approaches to perform QM/MM simulations as to the embedding strategy, namely, using (i) an additive model or (ii) a subtractive model. In the additive model, the total energy is given as
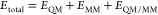
34where *E*_QM_ and *E*_MM_ are the QM energy and the corresponding MM energy, calculated using an MM force field, respectively. *E*_QM/MM_ is the cross interaction between the QM and MM parts, consisting of electrostatic, van der Waals, and the MM bonded interactions if there are chemical bonds in the interface. Other terms are added to make the QM/MM coupling more realistic, for example, for polarization effects.^362^ The hybrid QM/MM method, such as eq 34, was originally presented as an adiabatic method using Born–Oppenheimer dynamics with electronic DoF included at different levels of theory from fast SEMO methods to CC and other high-end *ab initio* schemes once the energy gradients could be calculated. Excited states can also be treated using QM/MM, but since the charge densities can be different in ground and excited states it is important to include polarizability contributions in the coupling term.

**Figure 4:**
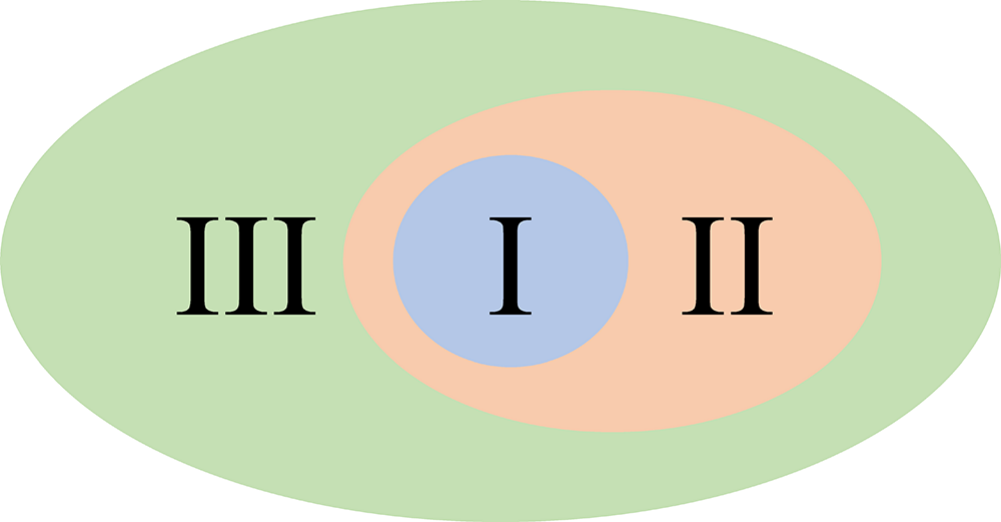
ONIOM partition to (I) QM1 more accurate, (II) QM2 less accurate, and (III) MM part.

In early 90s, Morokuma and co-workers did suggest an alternative hybrid QM/MM scheme^363^ which became later called ONIOM (Our own N-layered Integrated molecular Orbital molecular Mechanics) and was also implemented in the Gaussian98 software package in 1999,^364^ representing the subtractive embedding. As an example, using the ONIOM embedding (with a clear pictorial resemblance to spherical shells around an onion, although they can be in principle of any shape), we can compose the entire studied system from overlapping regions I, II, and III according to Figure 4.

Here, I is the reactive region and is embedded both in II and III. II is also assumed to be described having electronic degrees of freedom but at a lower level of theory, while III can be described as a nonreactive domain treated as a classical simulation box using molecular mechanics. The total energy can be calculated after decoupling the multiply treated regions as
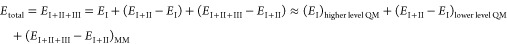
35

The scheme is general concerning the number of shells so it can also be one region embedded in another bigger one, either of the (QM1/QM2) or (QM/MM) type. An excellent review of the ONIOM method and its applications is ref (365). Both the additive and subtractive scheme can be used to describe carbon nanodots as a QM region and surrounding, for example, complexed biological material (protein, DNA etc.) described at the MM level. An example of this using the ONIOM model is found in ref (366) although for quantum dots.

Since both QM and MM parts can be chosen by the user, once they both can communicate with each other, in principle, a nonadiabatic scheme can be used as a QM part. This is what we start to see^367,368^ with, for example, the NewtonX package, already being linked to many QM software packages.^369^

In BO dynamics, the nuclei are fixed in the positions from the previous integration of the equations of motion of the atom masses while the electronic structure is recalculated to give the forces to move the nuclei classically to the next positions. No force field is needed as the potential energy surface is evaluated from the wave functions for the current configuration. In programs like NewtonX,^369^ which is a general-purpose software for excited state dynamics, the nuclear motion can be followed on a multitude of energy states and surfaces. One of the most used techniques today is the Surface Hopping (SH) method allowing an MD trajectory to hop to another level at specified conditions and probabilities. Trajectory surface hopping was suggested as early as 1971 by Tully and Preston^370^ to follow nonadiabatic collisions of a proton in reacting with D_2_. SH has been generalized by Tully and many others since then. The reader is advised to excellent reviews by Tully,^371^ Wang et al.,^372^ Nelson et al.,^373^ and Long et al.^86^ Nonadiabatic MD was used to characterize the chemistry, geometry, and electronic structure of several nanoparticles composited with TiO_2_ to study photoinduced electron and energy transfer on the interfaces following energy relaxation for possible applications for photovoltaic and photocatalytic cells. The type of donor–acceptor interaction was found to be crucial for efficient charge separation. NewtonX, from the group of the software developers,^374^ was used to perform MD simulations where H atoms (radicals) chemisorb to a graphene sheet with defects to form sp^3^ hybridized C–H bonds. Perfect graphene is normally chemically inert. Carbon atoms in the para position show most reactivity toward adsorbed hydrogens.

##### 2.2.2.6 Dissipative Particle Dynamics

Dissipative Particle Dynamics (DPD) is a classical particle-based simulation method for soft matter and mesoscale systems in general. DPD is a relatively new simulation method, introduced by Hoogerbrugge and Koelman in 1992.^375^ The first formulation of DPD by Hoogerbrugge and Koelman did suffer from not having a correct coupling to statistical mechanics. This was presented 3 years later in 1995 by Español and Warren,^376,377^ also connecting it to the fluctuation–dissipation theorem.

**Figure 5:**
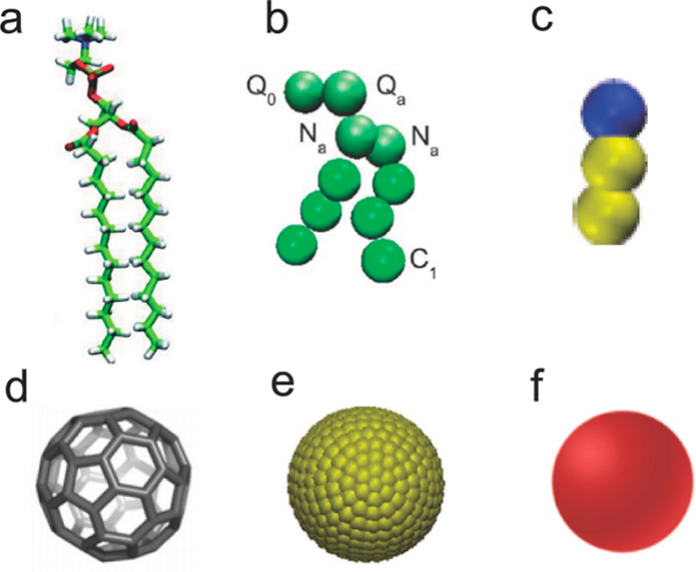
Typical CG models for lipid molecules (b,c) and nanoparticles (e,f) with different levels of coarse graining used in DPD simulations. The structures on the left (a and d) are corresponding all-atom models. Reproduced with permission from ref (378). Copyright 2014 Wiley-VCH.

DPD particles interact though three forces: conservative, dissipative, and random. Intrabead forces for “bonds”, “angles”, and “dihedral angles” can be added in cases where the CG particles are connected to each other. With conservative forces, various soft interbead potentials can be used, whereas dissipative and random terms work together as a thermostat. Commonly, the Flory–Huggins χ_*ij*_ parameter is used for soft repulsion between the CG beads.^379^ Due to the heavy masses of CG beads and soft interaction potentials, DPD allows much longer time steps than all-atom MD, making it an ideal simulation method to cover the length scales needed for those studies which currently are not feasible with all-atom simulations. For more details, see the original papers^376,377^ and recent reviews of DPD perspectives by Groot and Warren^380^ and Español and Warren.^381^ Several highly efficient software packages are available for performing DPD simulations. We mention here GALAMOST,^382^ which has a variety of features such as different force fields, thermostats, barostats, boundaries, etc. and treats electrostatic interactions with linearly scaling Ewald methods; it is also designed and optimized to run on GPU cards. Very often DPD and CG simulations are used as a part of a multiscale modeling study, as DPD per se is not accurate enough to be used in detailed studies of CDs and related structures, and first-principles methods and classical AA MD methods are regularly needed for thorough in silico studies. For an overview of DPD simulations dealing with NPs penetrating lipid membranes, see Shillcock and Lipowsky.^383^ Among applications more relevant to this review are studies of the interactions and translocation of NPs, including various carbon materials, through lipid bilayer membranes.^378^ In building CG models for DPD simulations, there are several choices to be made, notably the assignment of CG beads, as illustrated in Figure 5. In this figure, the atomic-resolution (i.e., non-CG) structures of a lipid molecule and an NP (C_60_) are shown on the left (Figure 5a,d) and selected possible CG models to the right (Figure 5b,c,e,f).

The lipid zwitterionic headgroups together with the joints to the tails need more attention as they are charged, while for the aliphatic tails the CG mapping choice is more straightforward; the optimal assignment is always to choose roughly the same masses for all CG particles.

For example, Li and co-workers,^279^ in their study of the cell membrane piercing of graphene nanosheets, used a 13 bead-model for POPC lipids, of which three describe the headgroup. The graphene monolayer CG model was adapted from Cranford et al.^384,385^ and was calibrated to reproduce the experimental values of Young’s modulus, shear modulus, and bending stiffness of graphene. In this model, each graphene CG unit cell is made of three beads. An alternative coarse-graining of graphene is presented in the DPD study of Mao et al.:^306^ each benzene ring-unit is represented by a single CG bead connected hexagonally by six bonds to the nearest neighbor beads, resulting in a “honeycomb” topology, with an angle potential included between each two neighboring bonds. Experimental values of graphene elasticity were used to parametrize the model. Different degrees of edge and basal plane oxidation of the graphene sheet could be modeled by adjusting the Flory–Huggins parameter for selected graphene beads. The DPPC model of Mao et al.^306^ consists of nine beads, of which three are used to represent the headgroup, corresponding to Figure 5b when N_a_ and N_b_ are merged to one bead. The number of lipid heads per unit area was used to parametrize this model, a standard target property in CG simulations of lipids. Dallavalle et al.^307^ used a similar “honeycomb” CG model to study the effect of size of hexagonal graphene flakes on their interactions with a DOPC lipid bilayer, described using a “12 + 3”-bead DOPC lipid model. All CG models and parameters were adapted from the work of Shillcock and Lipowsky.^383^ Chen et al.^308^ developed a “6 + 3” CG lipid model and graphene sheet model similar to that reported by Mao et al.^306^ to study the transport of such sheets within a lipid bilayer as well as their applicability for enhancing the delivery of membrane-specific drugs (modeled using a single CG bead).

### 2.3 Building CND Model Structures

In view of building a useful model structure of CNDs and selecting the proper computational techniques to deal with a specific property, it is important to understand how different experimental techniques are applied to investigate CND systems. In the following subsections, we will overview them briefly reporting the main experimental results (section 2.3.1). These results are at the same time the starting and the ending points of the molecular modeling investigation, being both the guide to identify the essential features of the CNDs necessary to build a starting structure for the *in silico* investigation (section 2.3.2), to validate the results (section 2.3.3) and to explain them (section 3).

#### 2.3.1 Experimental Features as Targets and Benchmarks for Computational Methods

**Figure 6:**
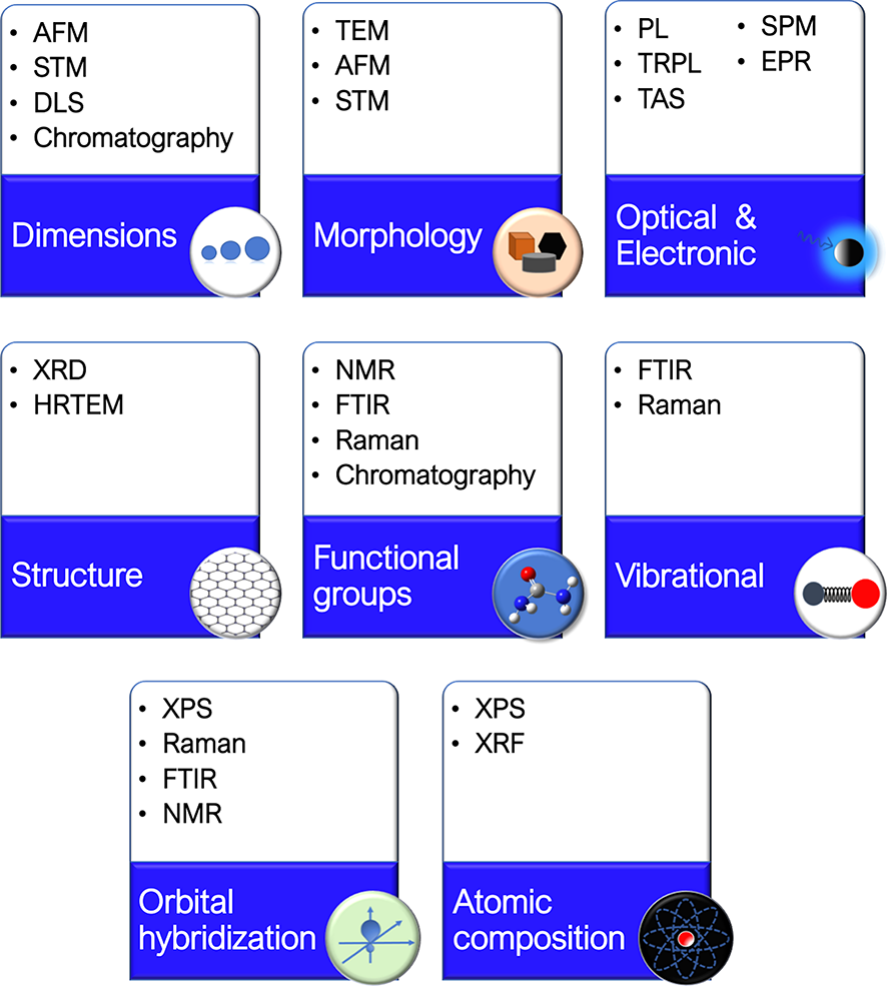
Schematic representation of the experimental techniques used to obtain structural information on CNDs and spectroscopic properties that can be used to verify the computational models. The acronyms are defined in the text.

Comprehensive characterization of CNDs involves the use of several experimental techniques to obtain information about structural, spectroscopic, dynamic, and functional properties, all of which are useful in building molecular models. The general approach to the study of CNDs is mainly based on the wide use of a variety of techniques capable of measuring precise properties, from structural to optical ones (see Figure 6). Among them, optical and vibrational spectroscopies and electron microscopy are probably the most relevant methods.

Despite the multipurpose approach, it is difficult to achieve a detailed description of CNDs and to identify direct correlations between their structural and optical characteristics. To guess reasonable structures capable of explaining the associated physicochemical properties, the modeler has to combine several shreds of experimental evidence related to the size of the particles, the type of functional groups that are present, the hybridization of the carbon atoms, and so on, as detailed in Figure 6. To demonstrate how experimental indications can be exploited to build a computational model, we consider TEM and high-resolution TEM (HRTEM) imaging, that on average indicate rounded nanoparticles of less than 10 nm in diameter with graphitic diffraction planes at 0.2–0.3 nm (when ordered structures are found). Such a large size system generally cannot be simulated with advanced quantum mechanical methods, and one has to use SEMO or force field-based methods, thus keeping the morphology information but mostly losing the details on the fine structure (see the work of Margraf,^79^ Paloncýová,^271^ or Sau^38^). To keep the structural details, the graphitic order is overimposed on the model system, as for refs (73, 95, and 271) and eventually a hybrid approach with both quantum and molecular mechanics adopted to retrieve other crucial information, such as optical features. As reported in Table 4, the larger part of the simulated systems are single or double graphene-like layers, those fixing the acceptable limit to few nanometers on the lateral size and a few tenths of a nanometer in the vertical direction. A second example we can consider are the results of the vibrational spectroscopies, Raman and FTIR. Both can give information about the functional groups and the orbital hybridization, and there are many papers that report the simulation of small PAHs or a single graphene layer with specific terminations. As for the case of doping, the limit in this case is once again the size of the model, the computed concentration of edge and doping defects are in general larger in comparison with the experimental concentration. If the exact concentration, as indicated by elemental analysis or XPS measurements (*vide infra*), cannot be in general achieved, the typical compromise is to consider the reference undoped or unfunctionalized model and to gather information from the comparison of the two systems. Many experimental spectroscopic observables can be predicted from computational studies, as specified in section 3. Thus, even in cases where the experimental observables do not have a unique link to a given structural feature, they are extremely useful to indirectly verify whether the used model correctly represents the system under investigation. The following subsections describe the main information we can gather with the different techniques. We also indicate the main limitations of each technique and present the most relevant examples for CNDs.

##### 2.3.1.1 Optical Spectroscopies

The interpretation of optical absorption and photoluminescence (PL) is a crucial task for a full comprehension of the characteristics of CNDs. In general, high energy absorption bands (200–300 nm) are attributed to strong π → π* transitions, which involve C=C aromatic bonds.^386^ Bands at higher wavelength, less intense, are correlated to *n* → π* transitions of C=O in the carbon structure or C=N lone pairs. Doping with oxygen and nitrogen is believed to promote the absorption at high wavelengths in the UV or blue regions (350–450 nm).^12,387^ Lower energy transitions that trigger the fluorescence in the red or near-infrared regions are still under debate, although the fundamental role of nitrogen is generally recognized.^388^

**Figure 7:**
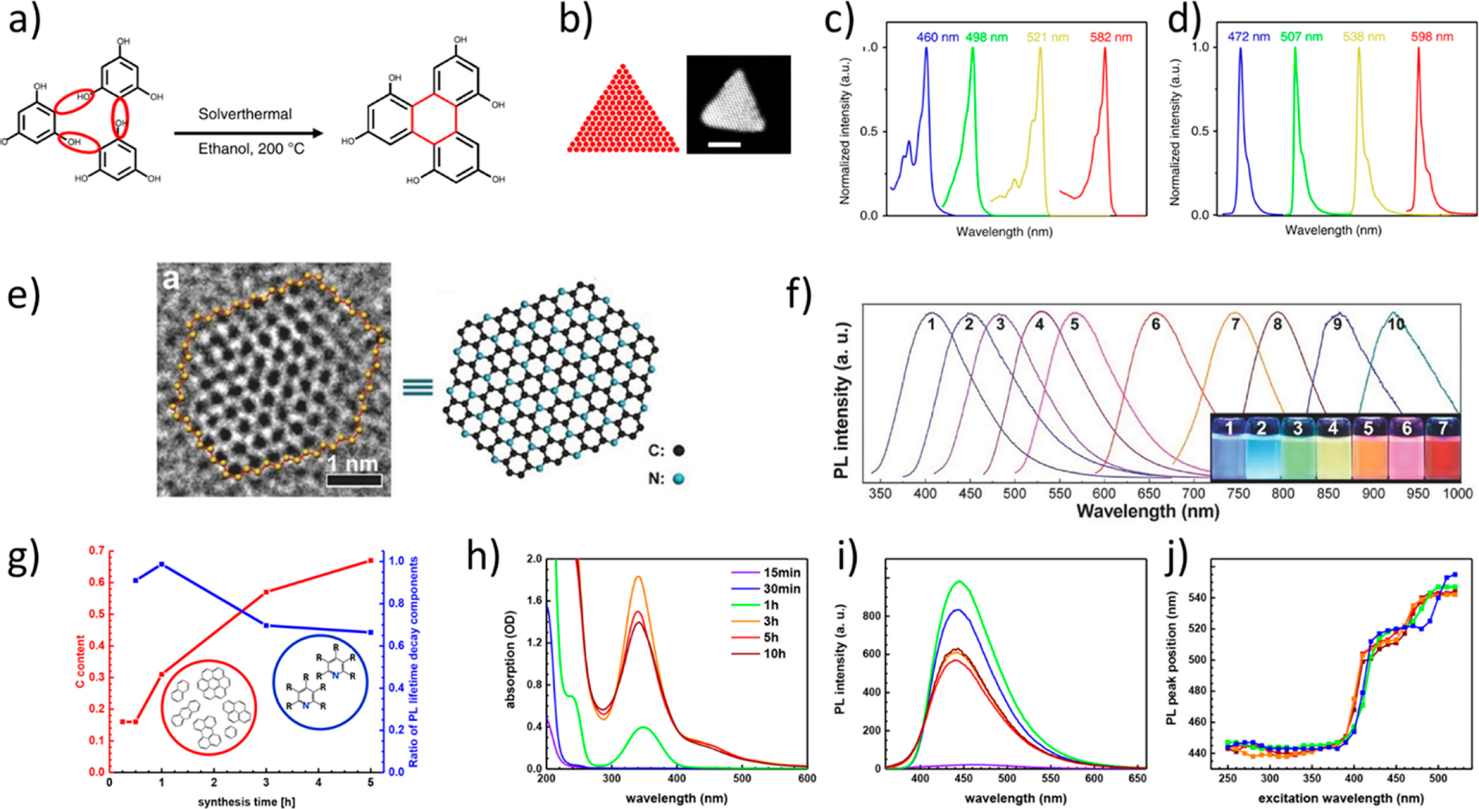
(a) Formation mechanism of phloroglucinol derived triangular carbon dots by the solvothermal method; (b) HRTEM image of triangular graphene carbon dots and corresponding (c) normalized absorption and (d) fluorescence spectra. Readapted with permission from the work of Yuan and co-worker,^44^ Copyright 2019 Springer Nature. (e) HRTEM of C_3_N CNDs and (f) photoluminescence modulation of C_3_N CNDs as a function of the size. Readapted with permission from ref (392). Copyright 2017 Wiley VCH. (g) Carbon content and PL lifetime, (h) absorption spectra, (i) fluorescence spectra, and (j) PL maxima positions of citric acid and ethylenediamine derivate CNDs as a function of reaction time. Adapted with permission from ref (393). Copyright 2017 American Chemical Society.

It is often assumed as a reference model that the optical properties of CNDs originate from a sp^2^-carbon network. In this case, the optical properties are expected to depend on the dimensions of nanoparticles and, correspondingly, the size of sp^2^ domains. Accordingly, the optical properties are tunable by controlling the CNDs size, as for semiconductor QDs by quantum confinement. This was the early interpretation of the most common CNDs.^389^ Although advances in the synthesis and investigation of CNDs have motivated the use of core–shell and fluorophores models, some studies are still supported by this size-related original description.^390,391^ Triangular CDs obtained by Xia and co-workers through solvothermal treatment of 1,3,5-trihydroxybenzene (Figure 7a,b)^44^ exhibit narrow emission strongly related to the dimension of aromatic graphene domains (Figure 7c,d). TDDFT calculations support this interpretation, although the optical features seem to be governed also by the geometry of the graphene cluster. Rational choices of solvent and synthesis time enable one to tune the size and wavelength emission of the system, with Stokes shift between optical absorption and photoluminescence on the order of few tenths of nanometers or less. The same trend was observed in C_3_N clusters (Figure 7e) obtained by polymerization of 2,3-diaminophenazine under hydrothermal conditions,^392^ where a wide range of wavelengths, from UV to near-infrared, can be obtained as a function of C–N network dimensions (Figure 7f). However, this “quantum-like” behavior is less defined in most CNDs, where sp^2^-hybridized domains in a sp^3^ carbonaceous matrix can account for the fluorescence and the large Stokes shift.^393^ Up until now, the most sound models to explain the optical features of CNDs are functional groups attached to the surface of the nanoparticle or the presence of specific fluorescent aromatic molecules, possibly in some aggregated form. The former, that is the formation of surface states on the surface of CNDs due to the presence of various chemical groups, can explain the interaction of the CNDs with the surrounding environment, leading to pH sensitivity, solvatochromic effect, oxidation effect, and surface passivation/functionalization. As suggested by Mintz et al.,^11^ extrinsic surface centers related to surface lattice defects due to adsorbed or bonded chemical species could be the origin of the emission features of CNDs, at least the environment related ones. The latter, that is the formation of specific emitting molecules, can be expected both in the core of the CND or at its surface. Within the molecular framework, the formation of collective excitonic effects was also proposed to explain the optical features of CNDs.^394^ The presence of molecular aggregates was reported, and the molecular exciton theory was successfully applied.^51^ Indeed, small PAHs in an amorphous structure can determine the excitation-dependent emission wavelength. For example, it has been demonstrated that CA and EDA form fluorescent species in the early stages of hydrothermal synthesis and sp^2^ domains arise within minutes (Figure 7g). As shown in Figure 7h, the UV–vis absorption is characterized by two strong bands at 250 and 350 nm due to π → π* and *n* → π* of 2-pyridone molecular moieties.^395,396^ Whether and how the fluorophores interact in the synthesis process of graphitic domains is still unknown since in the process, particularly under critical conditions such as those of solvothermal treatment or pyrolysis, several mechanisms and precursors could be involved.

The presence of molecular fluorophores is indirectly demonstrated by comparing the optical properties (UV–vis and fluorescence) of single molecules in solution with the ones in CNDs. However, it is not clear how the fluorescence of such molecules can survive the quenching mechanism within the carbonaceous structure. In that sense, the case of CZA and HPPT (4-hydroxy-1H-pyrrolo[3,4-*c*]pyridine-1,3,6(2H,5H)-trione) is quite peculiar, being considered responsible for the blue and green fluorescence in CA and urea derived CNDs,^25,397,398^ whose QY exceeds 30%.

An additional issue in understanding optical spectra of CNDs relates to the role of S,N-doping on the efficiency and spectral characteristics .^399^ The combination of CA with sulfur or nitrogen-rich precursors allows for effective doping of the graphitic structure.^400,401^ This drastically affects the optical properties of the produced CNDs by increasing the QY and, in some cases, promoting a strong redshift. Phenylenediamines are an exemplar case. Under the same synthesis route, the three isomers of phenylenediamine (meta-, ortho-, and para-) can produce CNDs with three different emissions, namely, blue, yellow, and red, respectively.^402^ The positions of the amine group and N-doping atoms in the final dot structure unpredictably influence the way the CNDs form. In the same way, the solvothermal treatment of CA and urea in formamide can produce CNDs with nitrogen-doping dependent emission.^42^ According to Holá et al.,^42^ the introduction of nitrogen defects in the sp^2^ carbon framework can be a suitable strategy for the realization of efficient red-emitting CNDs. S,N codoping in carbon dots has also been achieved^400^ through hydrothermal treatment of α-lipoic acid and EDA, leading to a significant enhancement of QY as compared to single doped counterparts. The synergistic effect of codoping was also demonstrated in graphene systems, where the improved catalytic performance was attributed to the marked redistribution of charge densities in the presence of the two heteroatoms, as also highlighted by DFT calculations. In particular, in carbon systems this codoping determines a higher concentration of C=N bonds which seems to be responsible for a greater photoluminescence efficiency in the carbon dots and a better catalytic activity in graphene, respectively.^403^ Overall, these findings indicate that the effect of CND doping on the corresponding properties cannot be predicted unambiguously, and therefore wherever additional experimental structural information is not available, all possible scenarios should be considered when building a doped CND model.

The dynamics of photoluminescence is even more complex. Time-resolved photoluminescence (TRPL) is a suitable technique to address the mechanisms of energy and/or charge transfer among functional groups or interactions among molecules. For small noninteracting molecules, the process of electronic deactivation is generally simple or easily framed in several tested models. On the contrary, the process can be complex in CNDs, with a large variety of CNDs exhibiting an emission wavelength-dependent lifetime. This seems to support the occurrence of complex structures in which inner states strongly interact with surface states. Generally, finding the correlation of the fluorescence decay profile with molecular aggregation, interaction among the functional groups or conformational state is one the most difficult challenges in optical spectroscopy.^404^ The possibility to unveil the mechanisms of charge/energy transfer could help acquire crucial information about the quenching process, origin of spectral components, and structure relaxation.^405^ So far, the effect of common mechanisms of molecular interactions, such as fluorescence quenching in solution or aggregation-induced fluorescence, have been rationalized for small molecules with encouraging results.^406^ Vibrational and rotational motions are also of great relevance in the processes of nonradiative relaxations. The inhibition of those motions favors the radiative relaxation that can be studied by time-resolved techniques. For example, restriction of intramolecular motion can favor the aggregation-induced emission (AIE)^407^ and promote an enhancement of luminescence efficiency. As pointed out by Li et al.^408^ and Peng et al.,^409^ some molecules can display a conical intersection (CI) in solution that is responsible for a weak fluorescence. In the crystal phase or in a more rigid medium, the constraints can inhibit CI and foster the radiative channels. This interpretation of AIE and, in general, of the fluorescence enhancement, could be extended to larger carbon systems and/or small fluorescent molecules adsorbed on them and explain some of the molecular features of carbon dots.

Transient optical absorption spectroscopy (TAS) can provide further data to complement the investigation of optical mechanisms. With this technique, the variation of absorbance as a function of wavelength and time is recorded, and a manifold of information about radiative and nonradiative electronic relaxation are acquired. TAS has been employed to investigate the role on the optical properties of surface sites in CNDs obtained from multiwalled carbon nanotubes (MWCNTs) by the top-down route^410^ and in CA/EDA CNDs^411^ to observe the kinetics of radical formation at the surfaces. Since TAS is sensitive to chemical environment, it has also been applied to sucrose/oleic acid CNDs with the possibility to discriminate among oxygen-containing functional groups in different pH conditions.^412^ The optical spectroscopy techniques are very sensitive, and the most applied to characterize the emission properties of CNDs. A clear limit, when using laser excitation as for TRPL and TAS, is the excitation power, since induced photochanges up to burning of the carbon structures can be potentially caused by high-density power light beams.

##### 2.3.1.2 Vibrational Spectroscopies

Together with optical spectroscopy techniques, vibrational spectroscopies represent key tools to investigate the nature of the functional groups and bonds in CNDs. Fourier transform infrared spectroscopy (FTIR) in the mid-IR range is very sensitive to the stretching and bending vibrational modes related to C–H, C=O, C=C, C–N, and N–H. Therefore, FTIR can be used to gain insights on the formation of the carbonaceous core, on the occurrence of heteroatoms, as well as on the surface functionalities of CNDs.^413,414^ The presence of H-bonds can often be inferred from IR spectra analysis, e.g., the peak associated with COOH involved in H-bonds (1710 cm^–1^), more intense than the peak due to “free” COOH groups (1780 cm^–1^), has been used to reveal the formation of a branched, compact, and static structure made up of nonconjugated polymers through H-bonding and ionic supramolecular interactions, and this type of information can be verified by other techniques such as XPS and NMR.^69^ It should be pointed out that precursors as well as byproducts may also produce signals in the same spectral range.

Raman spectroscopy is extremely efficient to identify and study graphitic structures, mainly through analysis of representative D, D′, and G bands in the 1300–3300 cm^–1^ spectral region.^415^ sp^2^ and sp^3^ hybridization, number of layers, and defects are frequently addressed by this technique.^416,417^ However, since the common excitation wavelengths are in the visible range, where the CNDs fluorescence is triggered, the Raman spectra result is inaccessible in most cases. Overcoming this issue requires the use of less common UV or near-infrared excitation wavelengths.

##### 2.3.1.3 X-ray Diffraction

X-ray powder diffraction (XRD) is used to gain structural information on the carbogenic core of CNDs. In this regard, XRD data may suggest a mainly amorphous/nanocrystalline nature of the core; in the latter case reflections are somehow interpreted based on the graphitic structure.^413,418−420^

XRD patterns can also provide information on the occurrence of additional phases such as those related to precursors or surface modifiers and hence contribute to the investigation of aspects related to the formation and resulting purity of the CNDs.^419,421,422^

##### 2.3.1.4 Microscopy

Microscopy techniques are used to gain insights on the dimension, shape, and crystallinity of CNDs. In particular, AFM^423^ and scanning tunneling microscopy (STM)^424^ are common scanning probe (SPM) techniques that can be used to investigate the dimensions (sometimes in combination with dynamic light scattering) and morphology of carbon particles. The size of CNDs is typically smaller than 10 nm, even if this limit is very often exceeded.

TEM is frequently used to characterize the morphology of CNDs and often associated with selected area electron diffraction (SAED) or HRTEM to combine structural investigation. Although nearly spherical, CNDs with size below 20 nm or their aggregates and poor crystallinity are usually reported, evidence of a wide variety of morphologies and crystallinities have been found depending on the preparation route.^418,421,425−427^ In correspondence to crystalline CNDs, structural features which can be associated with the d_002_ and d_100_ graphitic planes at 0.334 and 0.213 nm, respectively, and lattice constant *a* = 0.246 nm are observed (see for instance, refs (422, 428, and 429)). Despite the potential advantages in terms of combining insights on morphology and structure of nanoparticles, limitations related to possible beam damage and sample preparation should be taken into account.^430,431^ In particular, as for the optical spectroscopy techniques, imaging of carbon structures requires one to control the energy density of the probe to avoid further carbonization of the systems and in addition is limited by the poor contrast offered by carbon-based nanoparticles.

##### 2.3.1.5 Elemental Analysis

CHN analysis has been successfully used to gain information on the elemental composition of CNDs. In particular, CHN data can provide quantitative evidence of the occurrence of N-doping as well as of the effect of oxidation treatments.^432,433^

##### 2.3.1.6 X-ray Photoelectron Spectroscopy

Information on the elemental composition of the CNDs and on the bonding environment of a given atomic species in the topmost nanometers of a sample can be obtained by X-ray photoelectron spectroscopy (XPS). As an example, the deconvolution of the C_1s_ spectral region can be used to assess the ratio of the C–C/C=C, C–O/C–N, and C=O/C=N groups and verify how this varies with some parameters in CND preparation.^34^ It should be pointed out, however, that due to the typical size of CNDs, XPS measurements may provide information due to both the nanoparticle core and surface.^434,435^

##### 2.3.1.7 Nuclear Magnetic Resonance Spectroscopy

Nuclear magnetic resonance (NMR), with particular reference to ^1^H and ^13^C NMR, is a powerful tool to identify the chemical surroundings of selected atoms, thus tracing the molecular structure, including the chemical bonding characteristics, in organic compounds. Recently, the application of NMR to investigate CNDs has increased.

The origin of high fluorescence QY in polymeric CNDs derived from the carbonization of CA and 2-amino-2-(hydroxymethyl)propane-1,3-diol (Tris)^405^ or EDA^69^ has been investigated by NMR. Heteronuclear single quantum coherence (HSQC) and heteronuclear multiple bond correlation (HMBC) suggest that blue fluorescence of CNDs originated from the polymerization by amide bonds. Moreover, it has been pointed out the potential role of CA dehydration into aconitic acid in the strength of radiative recombination.^405^

NMR spectra (^1^H and ^13^C) have been also used to reveal the presence of sp^2^ and sp^3^-hybridized carbon atoms.^436^ In particular, in CNDs synthesized from melamine and dithiosalicylic acid, the aromatic rings signals due to CND cores have been detected at 8.3 ppm, while the carboxyl groups signals occur at 9.99 ppm. ^13^C NMR spectra display sp^3^ carbons in the range 30–45 ppm and sp^2^ carbons from 100 to 185 ppm.^436^ This is also confirmed in *o*-phenylenediamine CNDs, in which an extended aromatic structure has been detected in the region between 100 and 175 ppm (^13^C NMR spectra) and between 6 and 10 ppm (^1^H NMR spectra).^437^ Duan et al.^438^ have investigated CNDs obtained by microwave treatment of CA and EDA by a combination of ^13^C, ^13^C{1H}, ^1^H–^13^C, ^13^C{14N}, and ^15^N solid-state nuclear magnetic resonance (NMR) experiments. They have highlighted the formation of 5-oxo-1,-2,3,5-tetrahydroimidazo[1,2-*a*]pyridine-7-carboxylic acid (IPCA), characterized by the presence of =CH signal at 84 ppm (^13^C NMR) and 5.8 ppm (^1^H NMR), =CN_2_ resonance at 155 ppm (^13^C NMR), and two resonances at 80 and 10 ppm in ^15^N NMR spectra. On the contrary, no significant carbon aromatic structure has been measured, demonstrating the importance of precursors and synthesis routes in promoting graphitization and/or fluorophores formation in CND structures.

##### 2.3.1.8 Column Chromatography

One of the most common strategies to investigate the dimensions of CNDs is column chromatography. This method consists in the separation of chemical species by making the solute flow thorough a solid stationary phase. The efficacy of this technique is based on the different interaction strength of the chemical compounds with the stationary phase resulting in different retention times. Column chromatography is frequently applied to CNDs purification, allowing for a selection in dimensions and typology at the same time. Furthermore, the possibility to extract fluorophores weakly bonded to CNDs surfaces has also been reported.^439^

In general, column chromatography can provide useful information about the nature of functional groups and chemical species within the dots structure. So far, however, a satisfactory fundamental study is still lacking and no established protocol exists for CNDs purification and separation, although some tentative rationalization of this process has been done.^440^ Phloroglucinol-derived CNDs can be separated and purified by a combination of dichloromethane and methanol, which are reported to discriminate among the different CND species as a function of volume ratio.^44^ Column chromatography treatment by water and acetonitrile as eluents of CNDs obtained by hydrothermal reaction of CA and cysteine show the presence of several chemical species such as carbonaceous domains without carboxyl groups, CNDs functionalized with molecular fluorophores and free molecular fluorophores.^439^ Citric acid and urea CNDs are reported to be efficiently purified and selected by water,^441^ methanol, and acetonitrile.^442^ In addition, phenylenediamines CNDs are proved to be efficiently separated into blue, yellow, and red emitters by methylene chloride and methanol.^443^

##### 2.3.1.9 Other Techniques

Physico-chemical characterization of CNDs is based on a multitechnique approach. Some of the most established methods are summarized above, but several other techniques have also been applied to the investigation of CNDs.^431,444^ Just to mention a few, CND dimensions are also investigated by dynamic light scattering (DLS) with the aim to estimate hydrodynamic size,^422^ as well as sedimentation velocity analytical ultracentrifugation (SV-AUC) experiments which measure the sedimentation (s) and diffusion (D) coefficients.^67^ CND surface charge, which in turn can provide valuable information on the occurring functional groups as well as on CND dispersibility in suitable media, have been studied through zeta potential measurements.^433,434^ Additional techniques which may provide information on functional groups include thermogravimetric analysis (TGA) characterization, from which thermal stability can also be investigated.^414,434^ Overall, the experimental studies reported so far contribute to provide insights on the features of this relatively new and broad class of nanoparticles. It appears clear to us that a general multitechnique approach to assess the formation of CNDs, characterizing them, and discussing their features should be improved. This approach could provide evidence from one side of the correlation between chemical and morpho-structural features of CNDs, and from the other side of the correlation between composition, structure, and properties. The approach should include a large panorama of techniques (as many as possible among those shown in Figure 6), ranging from composition analysis, to functional groups identification up to the morphological characterization. Thus, by combining the gathered information, the approach could provide, in turn, robust grounds for modeling, going beyond the limitations of each single technique. For example, some issues relate to the application of given techniques (such as Raman, electron microscopy) to the specific investigation of CNDs. As reported in the previous sections, Raman and TEM are powerful techniques in describing the structures of the nanoparticles and their morphology. However, such techniques also have some limitations, the fingerprinting G and D Raman vibrations being also observable in large and aggregated polyaromatic structures,^445^ and the TEM imaging being always complex with carbon related materials. Additional limits on the information which can be achieved relate to the current control over the monodispersity and purity of the produced CNDs; hence, the potential of separation and purification methods is being investigated.^419,431^

#### 2.3.2 Strategies to Build Model Structures

The model used in the calculations depends on structural information at hand and on the properties we wish to calculate or answers we seek. For optical properties, the model for calculations becomes necessarily highly reduced compared to the actual sizes of real CNDs in the sample. The reduced model is typically built around the part responsible for the optical activities. It can contain important functional groups or a specific fluorophore molecule either physically interacting or chemically bound to the surface of the nanoparticle or embedded inside the core. Tables 1–3 give an indication of typical model simplifications, where polyaromatic compounds or graphene-like layers including either doped or functionalized with relevant edge groups can still give useful information about many optical properties of CNDs.^15,31,66,67,69,72,75,79,33,38,41,46,53,55,56,64^

The use of aromatic molecules as model systems is generally based on the assumption that the optical properties of CNDs are related to the sp^2^ carbon network. These systems do not correspond to real systems which often contain inherently heterogeneous systems in the samples and can contain varying fractions of sp^3^ hybridized atoms.

For instance, a CND can be modeled assuming different relatively small surface PAHs, as proposed by Fu et al.,^75^ combined with the experimental hints of Schneider et al.,^446^ to predict optical properties by means of QM methods. Clearly, when models can reproduce and/or help in interpreting experimental properties or mutual trends, as has been the case in several studies in the recent literature (see section 3), they can be considered good models (until better models are found). Simple models can be very useful and are often used in science to demonstrate principles. However, simple CND models are used partly to reduce the high computational cost, but more importantly, because accurate detailed models often do not exist and even if it would be possible to increase the model sizes with 1 order of magnitude, it would still not be a realistic model in comparison with the real systems. For each given composition and combination of orbital hybridizations of each type of atoms, even when the total number of atoms would be as little as 100, the possible spatial organization and configurational space would be huge. This is clearly an area where machine learning will be of great help in the future. Currently, a systematic chemical intuition-based approach is still used. However, there have been many successful examples as we can see from Tables 1–3.

**Figure 8:**
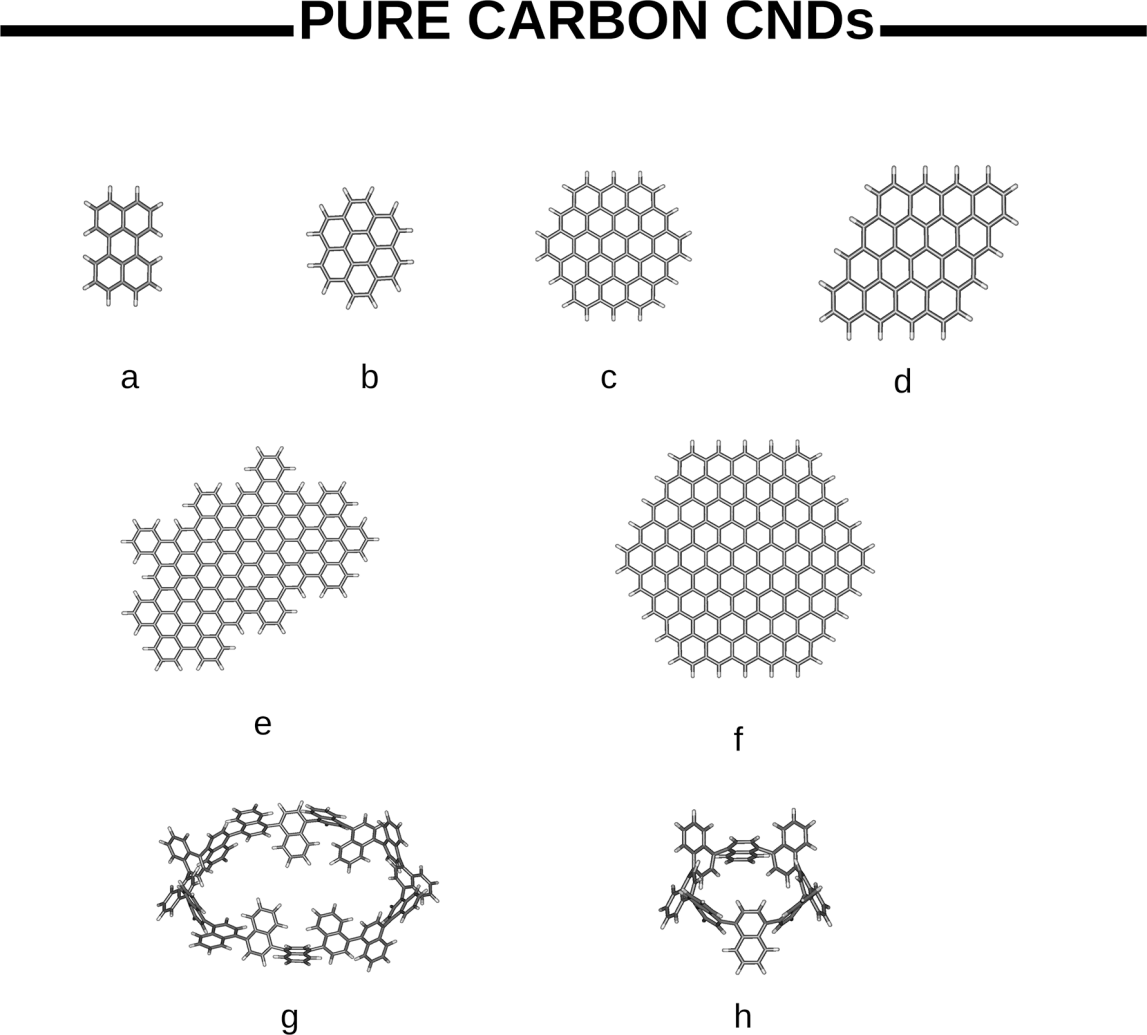
Examples of molecular models used in the recent literature to rationalize the optical properties of pure CNDs. (a) Kundelev et al. 2019,^52^ (b) Holá et al. 2014,^39^ Zhao et al. 2014,^26^ (c,f,g,h) Zhu et al. 2013,^27^ and (d) Sheardy et al. 2020.^77^

**Figure 9:**
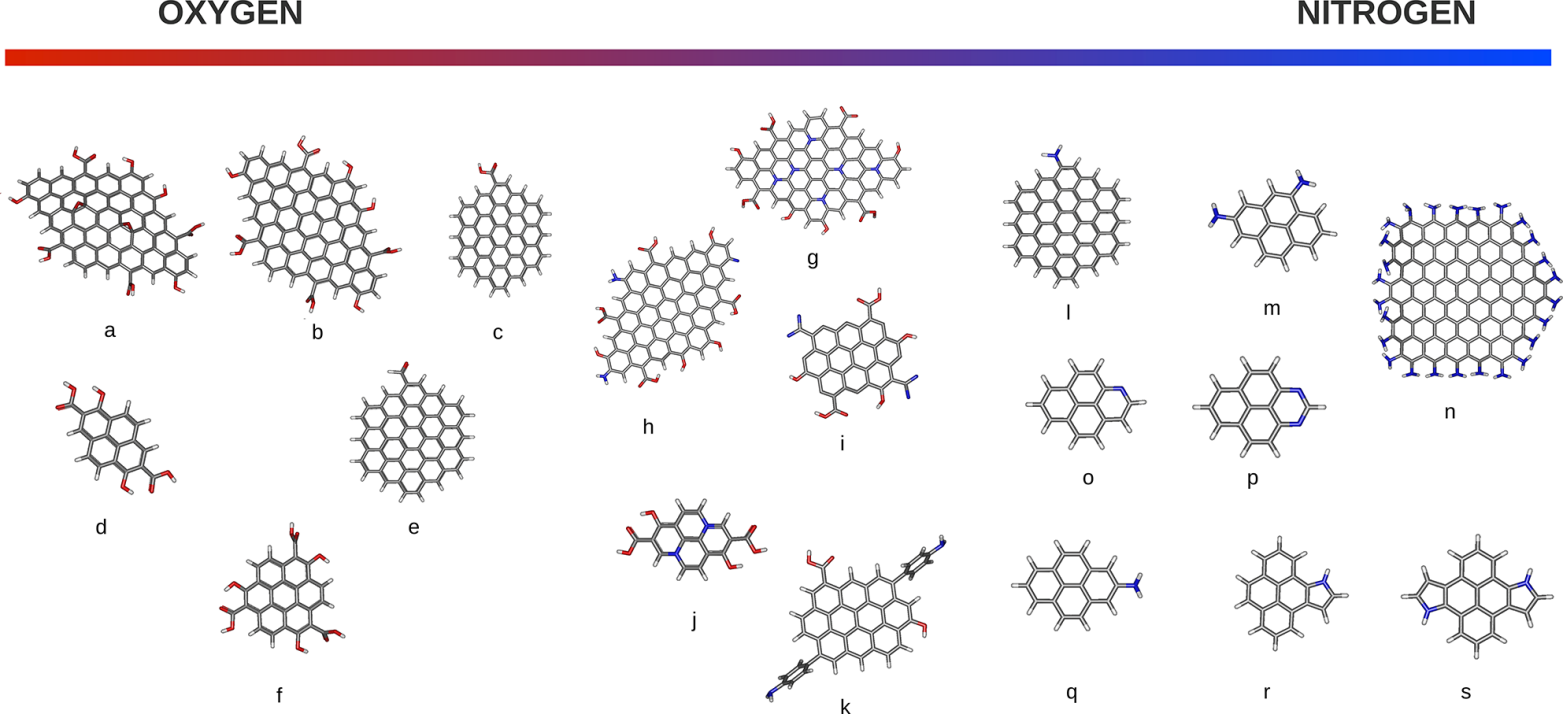
Examples of molecular models used in the recent literature to rationalize the optical properties of CNDs doped/functionalized with oxygen and/or nitrogen atoms. (a,b,g,h) Sheardy et al. 2020,^77^ (c,e,l) Li et al. 2015,^68^ (d,f) Sarkar et al. 2016,^41^ (i,k) Srivastava et al. 2019,^28^ (j) Holá et al. 2017,^42^ (m,o,p,q,r,s) Sudolská et al. 2017,^72^ (n) Wang et al. 2017.^25^

Different types of aromatic molecules such as pyrene, pyrelene, and coronene, often decorated with heteroatoms, have been systematically investigated, either in vacuo or in the presence of the solvent. Examples of molecular systems used as models of CNDs in recent investigations are shown in Figure 8 (undoped systems) and Figure 9 (doped/functionalized). CNDs are represented as planar (Figure 8f) or distorted (Figure 8g,h) PAH networks. Doping or functionalization with O (Figure 9a–f), N (Figure 9l–s), or both O and N atoms (Figure 9g–i) were considered. Such molecules can be used to obtain two-layers (2L) or multilayers (nL) systems constructed by stacking two or more 1L models. The Boltzmann populations of all the stable conformations can be used to mimic the chromophore diversity arising from the synthesis of the CNDs, and a procedure of averaging over the isomer populations and individual isomer absorption/fluorescence spectra can be applied to derive the final ensemble spectra, as done, e.g., by Sudolská et al.^48^ Indeed, conformational disorder, computed through multiple conformations of bilayers sampled with classical MD simulations,^67^ was shown to reproduce emission features better than frequently assumed CNDs’ polydispersity.

Several investigations have been devoted to understanding how the positioning and number of heteroatoms in the chromophore and the number of the functional groups influence the optical properties. In this type of study, the effects of several positions of heteroatoms within or at the edge of the polyaromatic structures are analyzed, considering the different possibilities, their number limited by means of the experimental data on the particle composition, size, vibrational spectra, and so on. Even with a limited number of heteroatoms and knowing from the experimental data the functional groups, the hybridization, particle size, etc., the possible combinations are very large. In the literature there are systematic studies presenting the effect of each functional group and their combinations. We have summarized these studies in section 3.2.

While the sampling of the conformational space of relatively small aromatic compounds can be achieved by systematically modifying few structural variables, the situation is different when several layers are to be included. In this case, it is useful to sample the conformational space using classical molecular dynamics simulations and verify the most probable and most stable configurations and thereafter perform QM calculations on them.^73^

**Figure 10:**
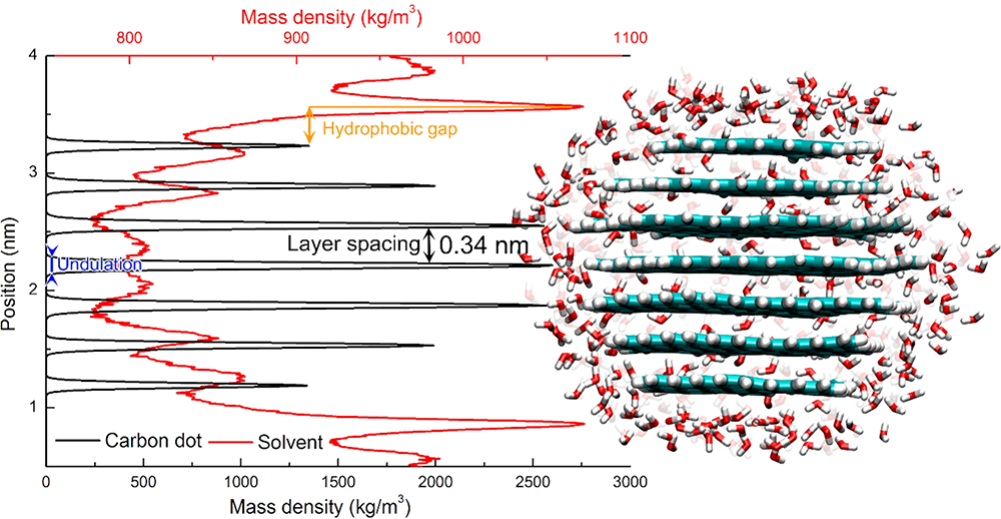
Density plot, interlayer spacing, and molecular representation of CND atomistic model developed by Paloncýová et al. for atomistic simulations with calculation performed using an OPLS-AA force field. Reproduced from ref (271) with permission of American Chemical Society.

Otyepka and co-workers^271^ developed a procedure for performing classical MD simulations of spherical CNDs with various oxygen-containing surface functional groups in solution. The authors presented a builder with a graphical user interface for constructing CND models of variable sizes and degrees of surface functionalization (Figure 10). The method is made available as a plugin for the widely used VMD software,^447^ and the authors describe development of an OPLS-AA based force field,^322^ with partial atomic charges derived from QM calculations on substituted circum-coronene models. MD simulations were performed to assess the stability, structure, internal dynamics (e.g., rotation of individual layers), and aggregation behavior of a variety of CNDs in water and *N*,*N*-dimethylformamide (DMF). Large CNDs with significant negatively charged carboxyl-functionalized surfaces were found to be stable in water, with a high degree of interlayer H-bonds.

In DMF, decreased stability and increased aggregation behavior of pure CDs was found, consistent with previous experimental reports, and also enhanced interlayer H-bonding for surface-functionalized CDs. Overall, the proposed model was found to describe the solution structures and behavioral trends of pure and surface oxygen-functionalized CDs in good agreement with available experimental results.

This model is proving useful not only in sampling the conformational space of the CND in various solvents but also to study the assembling or the embedding of molecular fluorophores in the CNDs^73,95^ or the interactions with molecules of pharmaceutical interest, of importance when the CNDs are used as drug carriers.^448^

CND models comprising sp^3^ carbon moieties or involving amorphous carbon regions have been much less studied.^38,79,82^ Such models are inherently more complex, and there is no standard procedure to generate them. Since the cases are not many so far, it is worth describing the procedure used to build these models, which requires either *ab initio* molecular dynamics to sample the conformational space, or SEMO calculations, or classical MD with reactive force fields (see section 2.2.1.2), or also a chemical intuition driven model building approach.

A useful approach to obtain amorphous CND could be to start from the amorphous carbon and cutting a spherical portion to mimic the carbon particle. McCulloch^84^ used CPMD to generate the spatial atomic configurations at different densities. Starting from cubic disordered structure with different densities (ranging from 2 to 3.2 g/cm^3^), the authors used the strategy to melt the sample by simulating the system at very high temperature (5000 K) and then cooling it down. As different densities naturally cause a different spatial organization, the obtained final structures show different amounts of sp^3^, sp^2^, and sp carbon atoms. An indication of the atomic orbital hybridization is given by the average coordination (which varies from 2 to 4 on going from sp to sp^3^) and the average bond angle (which varies from 180° to 109°). Samples generated at a lower density have a larger sp^2^ fraction, which can generate dimeric and chained structures as well as five-atom rings, while six-atom rings are more frequently found at higher densities. When the density increases from 2 to 3.2 g/cm^3^, the structure of amorphous carbon is more compact and loses the weak layering order present at the lowest density, a residual of a graphite-like structure. Correspondingly, an increase of the sp^3^ fraction can be traced, as confirmed by the growing trend of average coordination and reduction of bond angles. The model structures at the lowest density have non-negligible (15%) fractions of sp (in line with previous IR measurements^449^) and have a microheterogeneous structure, with simultaneous presence of consistent fractions of carbons in all its possible hybridization states, while at densities of 3.2 g/cm^3^, almost all carbons (∼80%) are blocked in sp^3^ hybridization. Further details on the comparison with experimental data are reported in section 3.1.2.

Margraf et al.^76^ built their amorphous CND models by optimizing hundreds of random configurations for a given number of carbons (with or without heteroatoms), keeping the box size constant. First, amorphous bulk carbon structures were approximated as periodic cells of fixed density, each containing 128 nonhydrogen atoms (unit cell size 10 × 10 × 10 Å) and they obtained final geometries by MC sampling in a melt/quench protocol.

CND models were obtained from the periodic structures by cleaving spherical particles, with a diameter ranging between 1 and 2 nm, saturating dangling bonds with hydrogen and then optimizing their structure. The resulting structures showed a different organization of the carbon network of the core compared to the surface, where the larger conformational freedom allows the sp^2^ atoms for the formation of a nearly planar condensed ring. Importantly, the obtained models were found to differ from the core–shell model, which consists of a graphitic core with sp^2^ ordered layers surrounded by a disordered sp^3^ surface shell. As noted also by the authors, different structural organizations are expected depending on the synthesis procedure and by the molecular precursors which can direct the structural organization toward localized preferred geometrical features. This inverted core–shell model, with disordered sp^3^ core and ordered sp^2^ islands on the shell, was further successfully explored. Indeed, as we will see in the following sections (see in particular section 3.1), beside the presence of a graphitic core, which largely depends on the performed synthesis, the presence of sp^2^ islands on the CND surface, as proposed by Tepliakov,^82^ such as PAHs or small aromatic molecules and even their aggregates^53,95^ could be necessary to properly simulate the optical features of CNDs.

**Figure 11:**
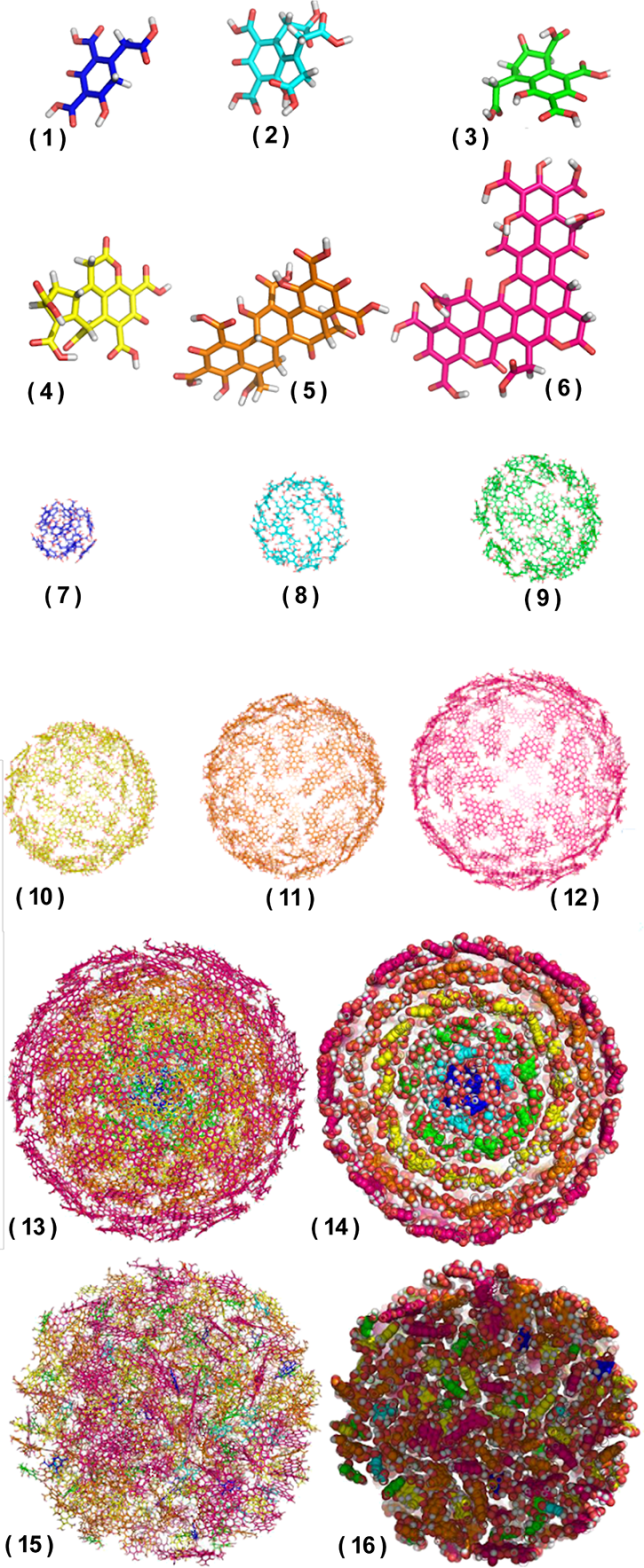
Schematic representation of the pCND model construction by Sau et al.^38^ through several steps. (1–6) Small molecule building blocks; (7–12) spherical shell structures assembled from building blocks, with corresponding color scheme. (13–16) Representations of ordered and disordered final structures obtained by layer-by-layer assembly of structures (7–12). Large models have sizes in the 10–15 nm range. Adapted from ref (38) with permission of the American Chemical Society.

A very elegant model construction as illustrated in Figure 10, arising from chemical intuition, is the one used by Sau et al.^38^ illustrated in Figure 11. They studied the formation of a polymer of CND, pCND, first obtaining the nanoparticles with pyrolysis of CA and ruthenium(III) chloride hydrate, RuCl_3_, and then refluxed with dithiothreitol (DTT) in dimethylformamide (DMF) at 120 °C. To build their pCND models, they did first consider a plausible mechanism for the formation of the “nanodomains” from the building blocks formed by oxidative decarboxylation and the subsequent acid catalyzed condensation reaction of citric acid (Figure 11, 1–6). They successively cross-linked the nanodomains (Figure 11, 7–12) and studied if the moieties, packed up during the synthesis, either in an ordered homogeneous layer-by-layer structure (Figure 11, 13 and 14) or in a disordered heterogeneous one (Figure 11, 15 and 16).

Aromatic molecular models are often used to model either by QM or FF based methods the interactions of CND with inorganic material and metals, small organic or inorganic molecules, biomolecules, polymers, etc. In section 3.6, we present several types of these studies, obtained either at the QM level, when small molecules are involved, or classical atomistic level, or for very large systems at the CG level. The building of the initial structure is even more complex increasing the number of molecules and hence molecular arrangements, and the model construction depends on the particular systems. On the other hand, the time and space scale available with atomistic and CG models generally allows the molecules to sample a large conformational space, and the results of the simulations should be reasonably independent from the starting configurations. This latter point is better evaluated by choosing a different initial arrangement and verifying the convergence of the simulated properties. In some cases, the best interactions mode can be verified evaluating the energies of the possible combinations; in others, several assumptions can be made from the information obtained experimentally concerning the group at the interface between the CND and the interacting molecules. See section 3.6 for several examples.

The choice of a specific strategy to build a model structure depends on many aspects, including the experimental data (synthesis procedures and obtained nanostructure) as well as the trade-off between accuracy and computational cost to describe and predict the chemical-physical properties (see Figure 11). Based on the literature, reviewed in this work, we can divide the proposed strategies in a multiscale ladder of increasing size and complexity. On the rung I of the ladder are the single layer PAHs, either pristine or doped/functionalized, heteroaromatic molecules.^27,39,48,67,236,450^ Model structures at this level are built considering the precursors reactivity, elemental composition, and experimentally observed functional groups.

Going up the ladder, on rung II, we find aggregates of the rung I models.^41,48,51,53,67,72,160^ These aggregates are characterized by a large conformation space, and to identify the most representative structures, the conformational space should be properly sampled and evaluated. This last step is often neglected in the reviewed literature. A semisystematic sampling can be based on chemical intuition, e.g., considering all or some of the possible combinations of H-bond donor and acceptor^51^ or considering stacked planar molecules able to rotate individually around their principal planar axis. This type of sampling can be performed with a geometry optimization at the QM level when the system contains few relatively rigid molecules. After increasing the system size, MD or MC simulations, either first-principles or classical FF-based, (best if followed by a clustering analysis of the most relevant arrangements) represent a good strategy to properly sample the conformational space.^67,95^

On rung III, we include both amorphous and lattice models of the CNDs, generated for example by cleaving a spherical shape from an amorphous or reticular periodic carbon network model (possibly including also doping atoms) optimized at the QM level. The cleft bonds can be terminated with appropriate atoms (depending on the CND composition data) and the resulting structure can be optimized with DFT, SEMO, CPMD, or reactive force field methods.^57,79,451^ The latter methods can also be used to simulate the CND structure obtained with top-down techniques. Concerning this rung, it is still to be evaluated in the framework of CNDs' properties what is already known concerning amorphous structures. Indeed, this knowledge is relevant to evaluate the effects on the optoelectronic properties due to disorder, distortion, and composition at different length scales, considering the size of sp^3^ clusters, the distance between these clusters, and the formation of sp^2^ islands.^82,452,453^

Rung IV contains the aggregates of molecules. Since the conformational space increases rapidly with the number of molecules and/or of the rotatable bonds, classical MD/MC simulations are the most viable option for sampling the conformational space and obtaining reasonable and stable CNDs model structures.^38,95,271^

We expect that in the future, the investigations on CDs interacting with biomolecular systems will use more models of rungs III and IV. Currently, the literature in the field has mainly limited to using models I and II, while the CNDs of rungs III and IV are rarely considered.

Models of rungs I and II often provide sound results for the optical properties, generally providing a good agreement with experimental data. To better describe the effects of the environment on the optical properties, we expect that models of rungs III and IV will become more common in the future. This can be made possible, for example, by using QM/MM methods with the demanding QM calculations made only on the fluorophore and describing the surrounding with MM models. The combination of models of rung I, in the bulk or in the surface of model of rung III of various complexity (containing doping atoms both and functional groups) is little explored and we expect that this venue may provide new useful information on the fascinating properties of CNDs. Another direction of improvement, as also highlighted in section 3.6.9 below, is the use of more realistic CND structures, e.g., those of rungs III and IV, in modeling the interactions of CNDs with other molecules, either small volatile compounds or large biomolecules.

#### 2.3.3 Model Validation

A very important step in molecular modeling is evaluating the reliability of the model employed. This step is even more important when modeling CNDs, since even the structures that are used as starting models described in the previous section are typically a very reduced portion of the real structure. In addition, they are obtained by using a great amount of chemical intuition and not a set of coordinates obtained through some well-established iterative process from experimental structural data, as is often the case for proteins and nucleic acids, for which detailed information are available from X-ray crystal structures or NMR in solution.

Although a fundamental step in computational modeling, no general protocol exists to have a yes/no answer to the question: is my model reliable? Depending on the aim of the research, available experimental data, computational skills, and software, the properties to be considered for model validation will be different.

Considering the great interest in understanding the optical properties of CNDs, it is not surprising that in many cases the models are validated by comparison of computational results with experimental optical spectra. Among the optical properties accessible to computational methods and largely exploited to validate a model, we find the HL gap (see, for example, Tepliakov et al.^82^) and the character of the considered transitions (such as charge transfer or excimer resonance).^114,160^ Also electronic DoS^57^ and fingerprinting vibrational modes^51^ are sometimes exploited to validate the models. However, the lack of knowledge of CND structure, the typical polydispersity of the nanoparticles, and the typically low control of their composition in terms of purity and reproducibility represent a limitation and challenge for the model validation effort. It is known, for example, that N-doping greatly increases the QY of CND emissions, but uncertainty regarding the concentration of nitrogen and its bonding states within the C-network allow for a large set of possible configurations, the optical properties of which can explain different experimentally observed emission features.^59^ When using simplified models to understand a given phenomenon, very often the comparison between the experimental data and the computed spectra should not be expected to give a quantitative match because of some underlying systematic errors in the modeling methods. The matching of the overall trend of the computed and experimental data is in general a better realistic reference to validate the results, see, e.g., refs (66, 73, and 84).

Large-scale computational models, e.g., MD or DPD simulations of CNDs, on the other hand, are often validated against experimental imaging results. For example, the average spacing between stacked graphene layers in the CND models of Elvati et al.^269^ or Otyepka and co-workers^95,271^ may be compared to direct measurements from TEM or AFM images.

Additionally, spectroscopic techniques, e.g., NMR or circular dichroism, have been shown to be useful for validating classical MD simulations of GQDs interacting with biological macromolecules such as proteins.^304^ Computer simulations of the translocation of CNDs or graphene sheets into/across lipid bilayers may be similarly validated (at least in part) by comparison with TEM or CLSM images of cell membranes or vesicles exposed to the NPs of interest.^278,279,295,308^

**Figure 12:**
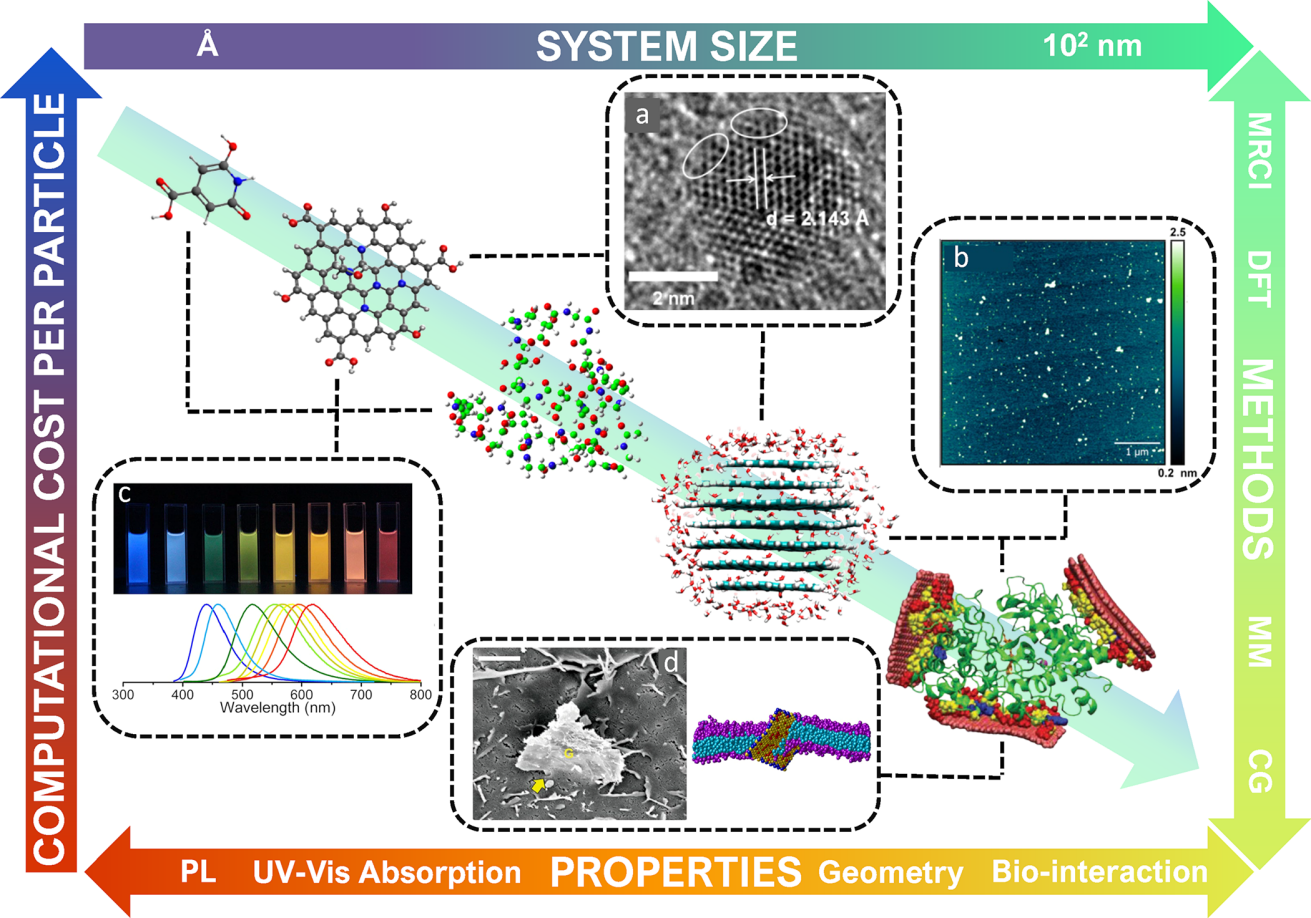
Example of computational models of CNDs of increasing complexity, from single molecule to molecular complex structures (from left to right along the green arrow, reproduced with permission from refs (51, 69, 75, 271, and 276)). In silico studies of CNDs, reviewed by us, can be illustrated and characterized using following practical “parameters”: (1) computational cost, (2) system size, (3) accuracy of the method, (4) theoretical and computational complexity of the property. They can be used to create a “road map” to find a compromised solution to perform computational studies of CNDs as shown by examples in the map. Four experimental features are reported and related to different computational models: structure and dimensions through HRTEM^65^ (a) and AFM^441^ (b), tunable fluorescence^454^ (c), and interaction with biomolecule^279,306^ (d). Inspired by the work of Dans et al., 2016.^455^

Measured thermodynamic properties may also be used to validate computational models: Margraf et al.^76^ compared the experimental and computationally predicted bulk densities to validate the capability of a given theoretical method to reproduce carbon phases, since low-density amorphous carbon phases are known to feature a higher proportion of sp- and sp^2^-carbon atoms than high-density phases. The heats of formation and geometries of diamond and graphite were also used to evaluate the method; satisfactory agreement with the experiment was obtained, although (as expected from the semiempirical method employed) in the modeled graphite, the distances between the layers were overestimated. Figure 12 summarizes the space scale of the systems reviewed in this work, connecting them with the methods and properties that can be used to characterize them.

## 3 Computational Studies of CNDs

Due to the wide range of possible applications of CNDs in different research and technological fields, a broad variety of computational studies have been performed in the past few years. One of the main focus of interest has been the origin of the emission of CNDs and the understanding of its excitation dependence or independence, and still it remains the holy grail in the quest for optimal fluorescent CND based devices. As a rule of thumb, bottom-up synthesized CNDs display excitation dependence of their emission, with the fluorescence peak red-shifting as the excitation wavelength red-shifts. Since one of the most accredited models for emission is the molecular one, this phenomenon is quite peculiar, because the Kasha rule for the molecular emission spectrum requires its independence on the excitation energy. To account for the excitation dependent luminescence, the hypothesis of a combination of different small polycyclic hydrocarbons or the presence of aggregates of molecules took hold (see, for example, refs (237, 393, and 456)). Sections 3.1 to 3.4 are dedicated to the review of the investigations in which molecular modeling helped explain the optical properties of the CNDs and the relationship with their structure. In section 3.5, we summarize the main conclusions on the effects of size, dopants, and functional groups on the optical properties. However, the interest in CNDs is not only due to their optical properties, and their possible applications as sensors make it desirable, if not necessary, to understand, at the molecular level, the interactions of CNDs with a multitude of small molecules. Similarly, the emerging applications in biomedicine require understanding how the CNDs interacts with biomolecules to clarify the potentials and risks associated with their use. Section 3.6 is devoted to reviewing these studies, with a special focus on the model and methods used for each type of study.

### 3.1 Nondoped CNDs

While the doping and functionalization of CNDs with heteronuclei is very common through many synthetic routes and is used to modulate the optical properties, plain nondoped graphene-like models are important as reference systems to understand how the chemical composition affects them. Indeed, several studies have been performed on these systems with different methods, either to rationalize the optical properties or the interactions with other molecules.

#### 3.1.1 Graphene-like CNDs

Many studies involving CNDs derived from different synthetic routes do use graphene as a simplified model of the particles. This is often due to the assumption that the most interesting optical properties of CNDs derive from sp^2^ carbon networks, thus strongly supporting the modeling by graphene layers.

**Figure 13:**
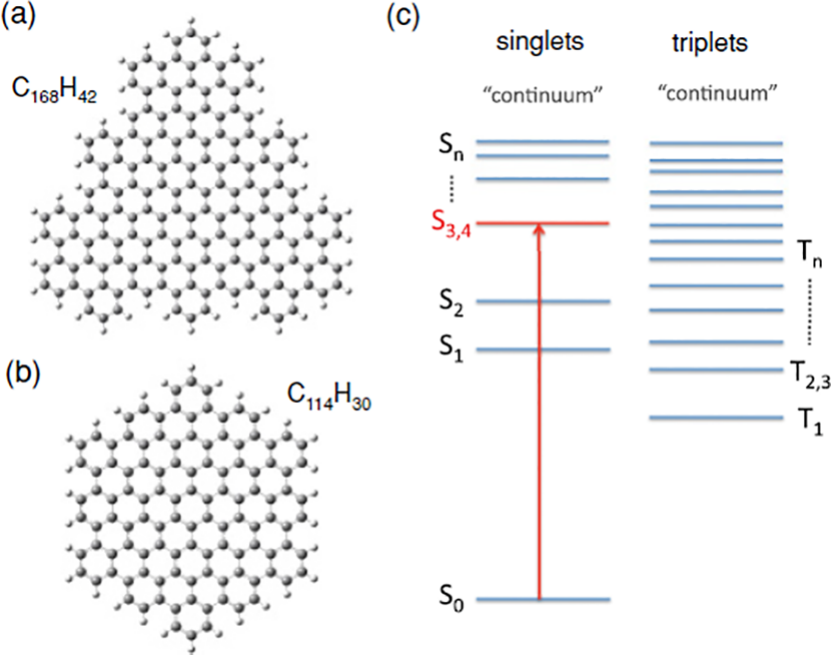
Molecular models of GQDs (left) and corresponding electronic levels (right) calculated at the B3LYP/6-31G(d) level of theory used by Schumacher.^36^ Reproduced with permission from ref (36). Copyright 2011 American Physical Society.

Among earlier DFT studies on GQD is the work of Schumacher,^36^ who studied the vertical absorption transitions in two molecular models, as shown in Figure 13, with a different symmetry and different number of atoms, C_168_ (Figure 13a), experimentally characterized by Yan et al.^238^ and a smaller C_114_ (Figure 13b) hexagonal system. All spectra are calculated using TDDFT and CI in vacuo since, according to the literature,^457^ only negligible corrections are required in nonpolar solvents. After comparing different DFT functionals, the author reports the ordering of the excited states (Figure 13c) to be independent by the specific functional adopted (see benchmarking section 2.1.11) and B3LYP being in better quantitative agreement with the experimental data of C_168_.^238^

According to Schumacher, the following features appear to be general in GQDs: (i) existence of dark singlet states below the first bright singlet transition, (ii) dominance of frontier orbitals (P shells) in singlet transitions, (iii) existence of dark triplet states below the first excited singlet state, and (iv) 2-fold degeneracy of the first bright transition, removed by vibronic coupling.

**Figure 14:**
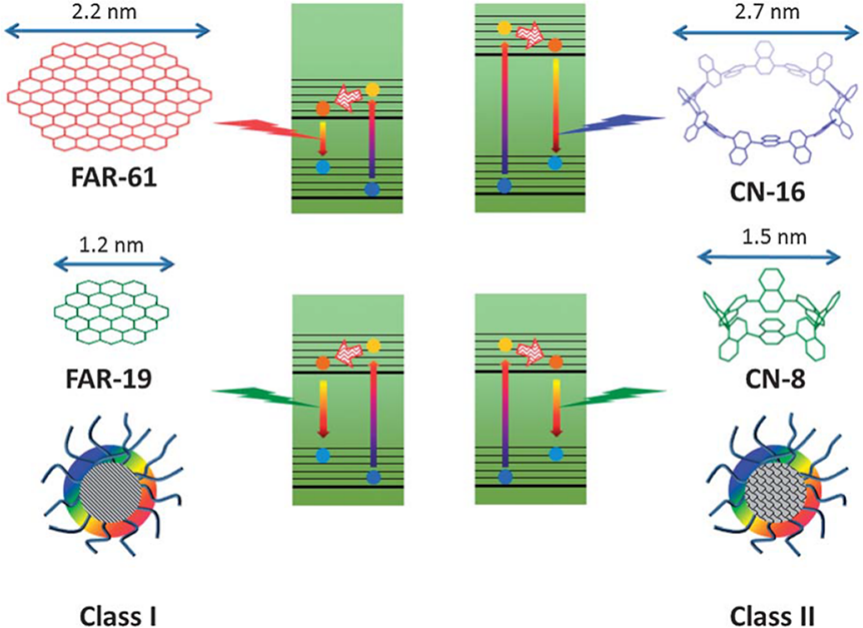
Two different classes of CND consisting either of fused aromatic rings or of cyclo-1,4-naphthylene based and related PL mechanism according to Zhu et al.^27^ Reproduced with permission from ref (27). Copyright 2013 Royal Society of Chemistry.

At the same level of theory as used by Schumacher, Zhu et al.^27^ studied the impact of CNDs microstructure on the PL emission spectra, observed in the UV–vis region and compared the optical properties of GQD (see Figure 14, Class I) to that of rings formed by linked aromatic molecules (see Figure 14, Class II). Exploiting a new three-stage synthesis, involving radical polymerization, cyclization mediated by intramolecular chain collapse, and carbonization, Zhu et al.^27^ successfully produced three rounded CNDs samples with diameters ranging from 4.5 to 2.0 nm. The author discovered an unusual relationship between the emission wavelength and the size: smaller CNDs exhibited larger emission wavelengths, in contrast to the quantum confinement effect and the commonly accepted “particle in a box” model which predicts longer emission wavelengths as size increases.

This unusual feature proved to be robust with respect to surface passivation, with an excitation-dependent PL still present after several hours of UV photobleaching but weaker intensity after surface reduction. Concerning the size effects, different trends are reported in the literature, with positive or negative correlation between size and λ_emission_ for CNDs prepared from cut-graphite layers (Li et al.^458^ and Lu et al.^459^).

Bao et al.^387^ focused on strong surface effects (possibly induced by defects or passivation) to explain the failing of the theoretical models purely based on quantum confinement effect in describing this kind of system. Bao’s idea, however, does not seem to apply to Zhu’s case, since surface treatments do not influence blueshift but proved to be detrimental just for QY (which, from XPS spectral analysis, can be related to partial suppression of carboxyl groups’ optical activity). According to Zhu et al., the explanation is related to the microstructure of the CNDs, which is more irregular compared to the crystalline, semiconductor QDs for which the “particle in a box” model appears to work.^460^

To test this hypothesis, the authors built two computational prototype-models (see Figure 14), a graphene-like fused aromatic rings (FAR, class-I) and a cyclo-1,4-naphthylene based CNDs (CN, class-II) and calculated the corresponding vertical emission energies as their sizes enlarge.

The authors found that while class-I FAR CNDs obey the general model proposed for semiconductor QDs, class-II CNDs display the opposite trend and explained this phenomenon by invoking the higher strain energy of class-II CNDs. In fact, while in class-I CNDs increasing size leads to a larger orbital delocalization but no significant increase of built-in strain energy (since the structure is planar), in class-II (which are mainly produced by carbonization of small organic molecules or polymers) size enlargement produces an increase of the strain energy in the sp^2^ bond structure connecting the predominant amorphous phase. Release of the strain energy can explain the blueshift as the CND’s dimensions shrink.

In Zhao et al.’s work,^26^ the absorption and photoluminescence spectra of PAH of varying size and shape (C_132_, C_168_, and C_170_) are calculated on both pristine and doped forms to verify the effect of hydroxy ether and carboxyl groups. Also, the effect of the solvent inclusion and the performances of various DFT functional are verified (see section 2.1.11).

The ether group is modeled as a part of an anisole molecule (PhOMe) functionalizing the PAH; the COOH group is either directly bound to the PAH or included as a benzoic moiety (PhCOOH) bound to the PAH. The ether and the carboxyl groups induce a very small redshift compared to pristine samples in the same solvent suggesting that the impact of solvation in toluene is clearly more crucial than functionalization via ether or carboxyl groups.

The COOH and OH functional groups induce a redshift of the absorption peak which increases with the number of functional groups. With OH, a very large red shift is observed when all the edge sites are substituted, and there is a large reduction in the HL gap.

Das et al.^32^ investigated the optical and magnetic properties of diamond shaped GQDs (DSGQDs), with the aim to understand the effect of edge geometry and symmetry. DSGQD of various sizes were considered (16, 30, and 48 carbon atoms) to understand the effect of quantum confinement and its role on magnetic and polarization dependent optical properties by DFT calculations. This study revealed that Fermi energy decreases when increasing the size and the C_30_ intermediate structure shows the highest magnetic moment among the three structures. Blue shifting of optical transitions occurs in the smallest DSGQDs calling for quantum confinement, while for the largest DSGQDs multiple broad peaks in the case of parallel polarization are gathered. Finally, Raman spectra simulations showed a G band peak for the biggest system, supporting the bulklike structure.

Among the papers on graphite systems as possible reference for CDs modeling, we want to mention the work of Hjort and Stafström,^81^ dealing with vacancies in a large single graphite layer. The theoretical approach is based on the Hückel method, to model both ordinary and hydrogenated vacancies within a 114 carbon atoms single layer of benzenoid polycyclic aromatic hydrocarbon. As for the obtained results, the ordinary vacancy was simulated by treating the three carbon orbitals around it as pseudo π-orbitals, leading to localized states around the vacancy, in agreement with electron spin resonance measurements. The extra charge was claimed to explain the bright spot observed in scanning tunneling microscopy measurements. Although in the hydrogenated case, the C atoms surrounding the vacancy are in the sp^3^ configuration, a significant overlapping interaction between the C–H bonds and the conjugated systems is required for the description of the hydrogenated carbon layer. The density of states and the optical absorption of the systems was studied as a function of the increasing vacancy concentration, up to 10%, increasing the system size up to 3786 atoms. The authors found little interaction among defects even at high concentration, with an overestimated absorption peak at 6.34 eV (to be compared to the 5 eV experimental peak). When hydrogenated vacancies are accounted for, the DoS is largely modified, casting some doubt on the previously claimed presence of an optical gap in hydrogenated amorphous carbon systems. The main conclusion from the authors is that large graphite clusters do not allow the formation of a band gap, the sp^3^ hybridization being requested for that for carbon systems.

The reactivity of carbon related nanosized materials, such as graphene and CNDs, largely depends on the presence of reactive sites where impinging molecules or radicals can more easily interact and become adsorbed at the surface. Nieman^374^ and coauthors, in a very recent paper, discussed the phenomenon on a graphene layer, assuming that a C–H bond defect is present on the surface of the circumcoronene model (C_54_) and exploring different trajectories of possible interaction with an impinging H radical. The static and dynamic calculations were performed within the DFT framework. Spin density on carbon atoms is the key parameter in selecting the most favorable trajectory to the target, a stronger bond being realized with carbon atoms with the largest spin density, the result being mostly inelastic scattering (48%) or Eley–Rideal (ER) abstraction of the chemisorbed H atom as vibrationally hot H_2_ (40%). With a residual 12% of probability also, new C–H bonds are formed, allowing local movement with low or zero-barrier reaction with the surface.

Single and aggregated PAHs are considered as a possible origin of the peculiar optical absorption and emission features recorded in GQDs and CNDs. Modeling of excited states and excitonic interactions is crucial to understand the optical properties from these graphitic regions, focusing on the role of single and aggregated systems, on the dependence, if any, on the model dimension of the considered PAH and on the effect of doping. There are two relevant and interconnected issues: to show that few paradigmatic PAHs can reproduce the large Stokes shift between absorption and emission bands recorded in carbon nanosystems and to identify the best computational method to achieve this result. The group of Lischka and co-workers dedicated a series of papers to tackle with these two aspects starting with the investigations of the UV–vis absorption spectra of pyrene and coronene as models for graphene quantum dots.^113,114,160,236,240^ In these papers, the authors proposed an analysis of the PAHs using single and multireference methods (SR and MR, respectively). Details for each work are reported in Table 3. Depending on the size of the analyzed models, different levels of approximation were used, as described in section 2.1.11.

With reference to the first study on the UV properties of pyrene and coronene,^236^ those PAHs were considered as prototypes of graphitic regions to study the effect of system size on the PAHs’ electronic excitations. The system size dimension was symmetrically increased by circularly surrounding the starting PAHs with benzene rings, thus exploring a range from 4 benzene rings in pyrene up to 37 benzene rings in the extended coronene system. The computed excitation transitions were directly compared with the electronic spectra of GQDs. When experimental references were not available, the DFT/MRCI method was used as a high-end reference, being proved the best performing method as compared to available experimental data (see section 2.1.11). Although TDDFT SR methods usually do not perform well in ordering the first excited bright and dark states, TDB3LYP performed quite well for the whole set of simulated models (better for the pyrene family, indeed) as well as the MR NEVPT2 method. On the contrary SORCI gave reliable results only for the coronene case, while the worst performing method was the TD-CAM-B3LYP, both in terms of larger errors in the estimated excitation energies and the order of the lowest two states. The SOS-ADC(2) does not perform well as the system size increases but confirmed through natural transition orbital analysis that multireference configurations are required to correctly characterize most of the lower electronic states. Finally, as shown by the extrapolation of the UV spectra to infinite PAH size, where MR methods cannot be applied because of computational cost, both SOS-ADC(2) and TD-CAM-B3LYP give results in a reasonable agreement with the experimental data, confirming that the PAH model can be applied to compute the optical properties of graphene-like nanosized systems.

Considering smaller pyrene and coronene systems, up to 19 benzene rings,^160^ the authors verified the best solution in modeling excited states of PAH dimers, e.g., systems where method properly treating long-range interaction and polarizability are required to describe charge transfer. The results indicate that the multireference benchmark DFT/MRCI should be preferred with respect to the other methods, when computationally affordable. ADC(2) and CAM-B3LYP overestimated the HL gap, the latter by a larger amount and failing in the excited state order, as previously also reported. For all the methods, most excited states showed multiconfigurational character with the absorption energies decreasing as the model size increases. In addition, the distance between the two monomers in the excited states decreases as compared to the ground state for the larger models. Those systems can be reasonably assumed as representative of graphitic regions in CNDs.

One of the fingerprints of CNDs and graphene-like nanosystems, beside the excitation dependent emission, is the large Stokes shift reported between absorption and luminescence features. In a later work,^114^ the role of the formation of excimer states in PAHs systems was considered, applying the same computational scheme as previously proposed to compare the Stokes shift in monomer and dimer structures. Although the emission features of simulated PAH monomers could not describe the observed shift in CNDs, the paper shows that trapping effects, induced by excimer formation, leads to significant Stokes shifts. Thus, excimer interaction in stacked dimers must be considered to account for the experimentally reported large Stokes shift in CNDs. In addition, as the number of benzene rings in the pyrene or coronene related models increases, a larger red shift of the emission feature was calculated. Finally, the authors also showed that the formation of bonding or antibonding orbital on single sheet PAHupon HOMO to LUMO (S_0_ to S_1_) excitation do well correlate with the character of the natural transition orbitals and the structural changes, in terms of C–C distance, within the benzene rings.

The performance of the SR and MR methods was finally tested on a linear model PAH system, tetracene, considering the stacked and T-shaped dimers.^112^ The goal was to analyze the excitonic and charge transfer interactions in those systems, providing a critical test able to describe the excimer survival process. The DFT/MRCI reference method gave absorption results in good agreement with the experimental one, also showing some expected configuration mixing in the excited states. The experimental emission excitation data for tetracene dimers do not typically show excimer characteristics, while the predicted results do show such a character with significantly lower energies, suggesting a possible way to discriminate the formation of exciton from other structural changes. Consistent results were obtained using all the methods, including the parametrized ones, in predicting the lowest energy S_0_ confomer, having monomers parallel and rotated relative to each other.

A displaced-stacked conformer was predicted to give the lowest S_1_ configuration. Spectral shape, state character, and potential energy curves were mutually consistent between all the used methods, despite some state-order inversions, and the effect of charge transfer interactions were in general relevant for the excimer and several higher states. These results indicate that the parametrized LC-TD-DFTB2 method could be potentially exploited to investigate excimer formation and survival processes in larger and computationally demanding PAH systems.

To end the subsection we note that computer simulation studies, including both classical and *ab initio* MD, aimed at understanding the mechanical and thermal properties of various pristine and functionalized graphenes were recently reviewed by Kumar and co-workers.^461^ The review notably also considers the properties of graphene-based polymer nanocomposites (i.e., graphene embedded in polymer matrixes), and we refer the reader interested in mechanical and thermal properties to this work.

#### 3.1.2 Amorphous CNDs

**Figure 15:**
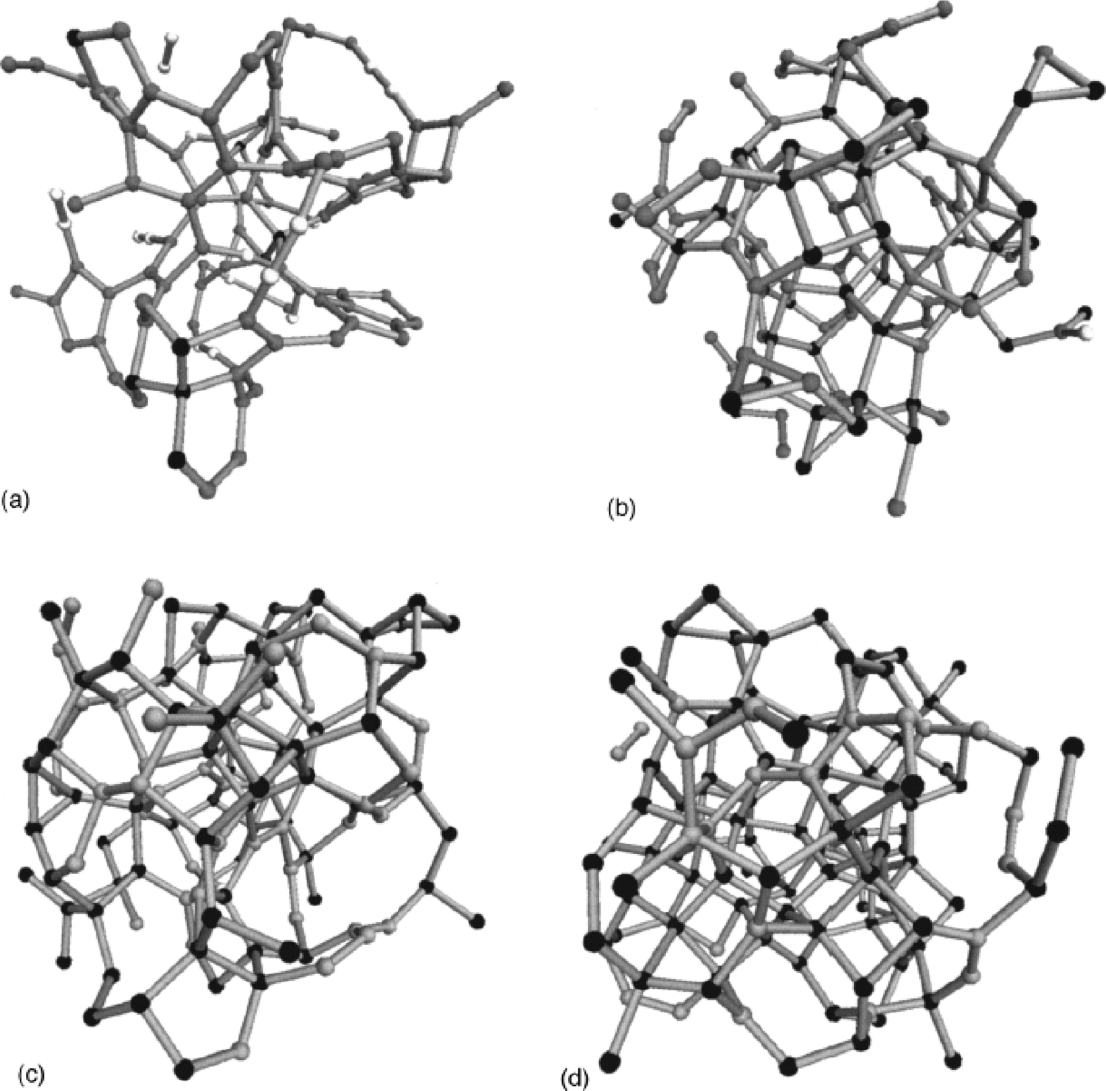
Final samples of amorphous carbon calculated at the B3LYP/PW(40 Ry) level studied by McCulloch et al.^84^ at (a) 2, (b) 2.6, (c) 2.9, and (d) 3.2 g/cm^3^. Black, gray, and white atoms are sp^3^, sp^2^, and sp coordinated, respectively. Reproduced with permission from ref (84). Copyright 2000 American Physical Society.

The influence of the sample density on the ratio of the sp^2^/sp^3^ fractions in amorphous carbon was studied by McCulloch et al. (see Figure 15).^84^ using CPMD as discussed in section 2.3.2. Although the used BLYP functional proved to be insufficient, giving unsatisfactory estimations of optical gaps on the generated structures (the predicted gap for the most dense sample is 0.8 eV, in contrast with experimental 2.5 eV^462^), a linear relation between density and sp + sp^2^ fraction is confirmed which is in sound agreement with experiments^463,464^ and previous simulations.^465^ A noteworthy behavior emerges from analysis of the sp^3^ fraction as a function of the intrinsic built-in stress of the samples: in contrast to the predicted graphite-diamond sp^2^ → sp^3^ transition, triggered by 1.4–1.6 GPa, in this work the authors report a sudden, linear, and dramatic increase of the sp^3^ fraction up to 4 GPa, with a less steep linear growth above this threshold.

**Figure 16:**
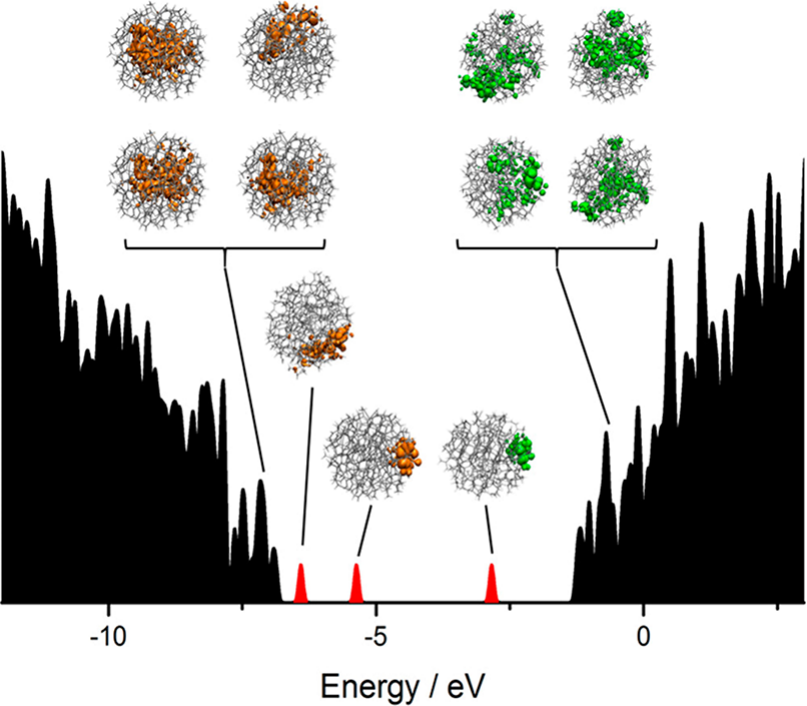
Diagram of the electronic density of states of a 2 nm amorphous CND and electron isodensity (0.01 e^–^ Å^–3^) plots of the orbitals corresponding to different bandlike and surface states. Calculations are performed using the INDO-S semiempirical method. Occupied orbitals are shown in orange, and unoccupied ones are in green. Black states are delocalized states within the structure, and midgap red states are localized on the surface of the dots. Reproduced with permission from ref (79). Copyright 2015 American Chemical Society.

Margraf et al.^79^ performed semiempirical molecular-orbital calculations on models for amorphous CNDs in order to study the factors that affect their electronic properties. The generation of amorphous bulk structure was achieved as discussed in section 2.3.2, and the validation of the used method (AM1) included verifying the heat of formation and geometries of diamond and graphite, which were a well reproduced exception given from the interlayer spacing in graphite, due to the inability of AM1 to reproduce dispersion interactions. In the periodic systems, the elemental composition influences the band gap only marginally. The researchers suggested that the observed electronic and optical properties of CNDs could be caused by the diversity of underlying structures. Overall, the decisive factor appears to be the effective degree of conjugation, which is given by the specific topology of the structure, where structural motifs comprising hexagonal rings similar to aromatic hydrocarbon are observed, although with structural distortions from planarity and not pure sp^2^ character. As the authors note “hexagonal carbon networks seem to emerge in amorphous systems. Conceptually, this establishes graphene fragments as components of CND and thus bridges the gap between graphene and completely amorphous”. Based on their analysis, heteroatoms influence the electronic structure by inducing structural changes. Their study on the CNDs indicates that the band gap decreases with increasing size, as in inorganic QDs. Differently from the latter, additional individual isolated states appear in the band gap: states in the continuous band region are relatively delocalized, while the midgap states are localized on the surface of the dots (see Figure 16). These sub-band gap surfaces states allow optical transitions in the near UV and visible range. This surface states are linked to the different conformation freedom of the atoms at the surface, which allows adopting localized planar geometry, which in turn affect the optical transition, and also the reactivity as evaluated by indices like the local molecular electrostatic potential and the local electronic affinity and ionization potential.

**Figure 17:**
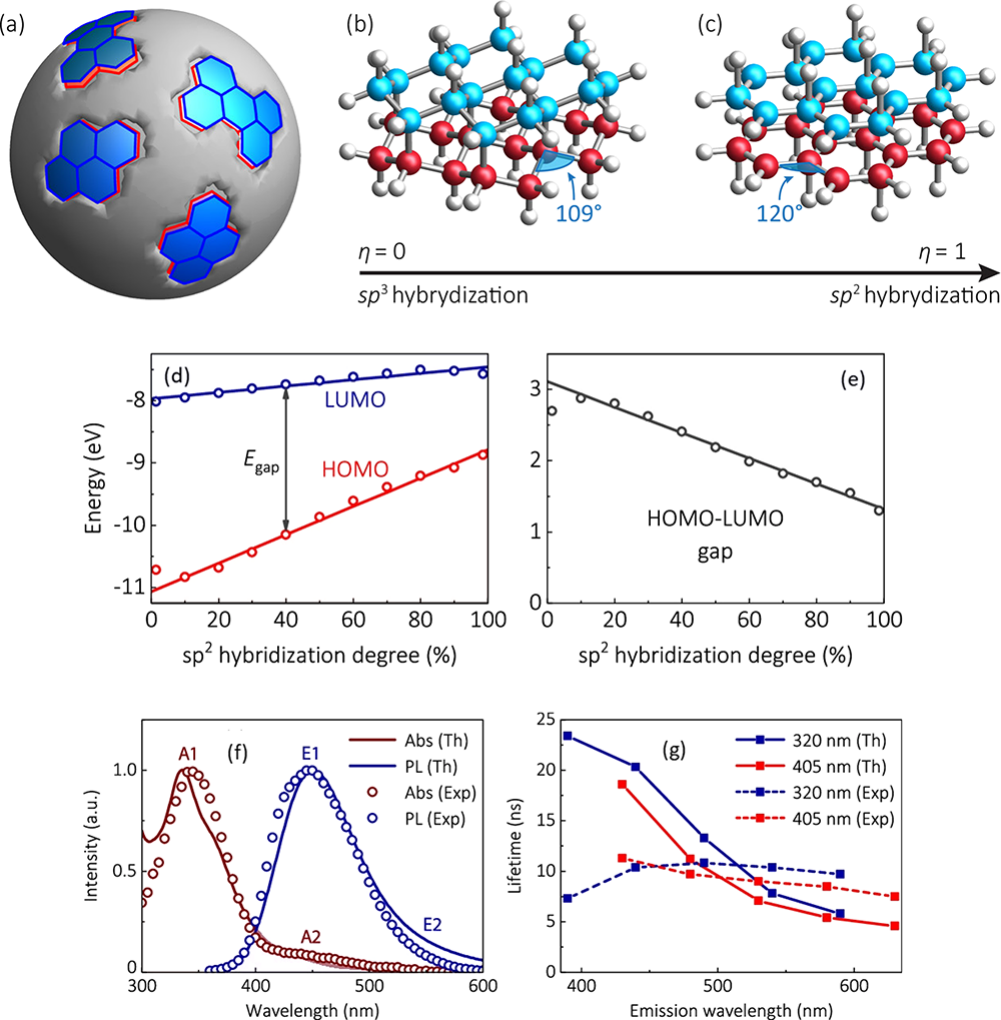
(a) CND schematic model with pyrene and pyrilene molecules at the surface. (b,c) sp^3^ to sp^2^ hybridization of the system. Blue and red spheres are C atoms; white spheres are H atoms. The variation of the HOMO–LUMO gap as a function of hybridization is shown in panels d and e. Absorption and emission simulated spectra (f) and estimated lifetime (g). Properties are calculated using EHM. Adapted with permission from ref (82). Copyright 2019 American Chemical Society.

A different approach was proposed by Tepliakov et al.^82^ that applied a semianalytical model based on the extended Hückel method to describe the optical properties of CNDs. In this approach the core–shell paradigm was inverted, assuming a sp^3^ amorphous carbon core with small domains of sp^2^ C atoms mainly located at the surface (Figure 17a), where the formation energy of these partially hybridized sp^2^ islands is expected to be less onerous. The sp^3^ polymeric core is represented as a mere support for the sp^2^ surface domains, providing the requested ballast to absorb any fluctuation and stress from the surface but not participating in the optical properties of the system. Indeed, the presence of this ballast allows considering double layers of aromatic molecules whose configuration is not usually recovered in dimer aggregates, being their reduced flatness and shorter distance granted by the supporting polymeric core. In this sense the authors talk about partial sp^2^ hybridization, since they assume that the carbon surface structures may form some intermediate configuration where structural parameters, such as, for example, the carbon angle, range from sp^3^ to sp^2^ values (between 109.5° in pure sp^3^ and 120° in pure sp^2^ systems in the case of the carbon angle, see Figure 17b,c). According to this study, the optical properties depend on the defined sp^3^ to sp^2^ hybridization factor (0 ≤ η ≤ 1) and the lateral dimensions of the aromatic domain, with the absorption and emission features red-shifted in larger molecules, as for the case of pyrene and pyrelene ones. Indeed, the authors tested their model on both the molecules, finding better agreement to experimental data for the pyrene case.

The optical properties are evaluated through the dipole approximation, retrieving absorption and emission spectra and emission lifetimes. As reported in Figure 17d,e, the proposed hybridization model predicts that the HL gap of the pyrene system undergoes a red-shift as the content of sp^2^ C atoms increases, linearly depending on the η hybridization factor. The analysis of the electron density on the two states suggested to Tepliakov and co-workers that the large Stokes shift of the emission spectrum (Figure 17f,g) with respect to the absorption one typically observed in CNDs could depend on the displacement of the electron density upon excitation that induces a stress on the structure able to temporarily modify the hybridization of the system. Based on these assumptions, the authors compared the calculated optical features (assuming a Boltzmann distribution of the η factor) to the experimental data. The agreement is very good, and the model also allows one to predict the observed peculiar excitation dependence of the CNDs emission as related to the distribution of the hybridization parameter. The latter also explains the inhomogeneous broadening of absorption and emission spectra.

In the last decades, different interatomic potentials have been developed for carbon, as discussed in the section 3.2. Deringer and Csanyi^330^ assessed the potential they produced with an ML algorithm (see section 2.2.1.4) by comparing different structural parameters of several carbon-based systems. The authors compared the lattice parameter and the elastic properties of diamond with the corresponding experimental values. In both cases, the agreement was very close even if modulus and elastic constants slightly deviate from the DFT reference. The next comparison involved the liquid and amorphous phases of carbon by analyzing the concentration of sp^3^ atoms during melt-quench simulations, the radial distribution function of the liquid phase, and the corresponding angular distribution functions. In all these cases, the potential reproduces these features very well, both the location and the extent of the maxima, thus promoting ML derived potentials for CND structure predictions.

**Figure 18:**
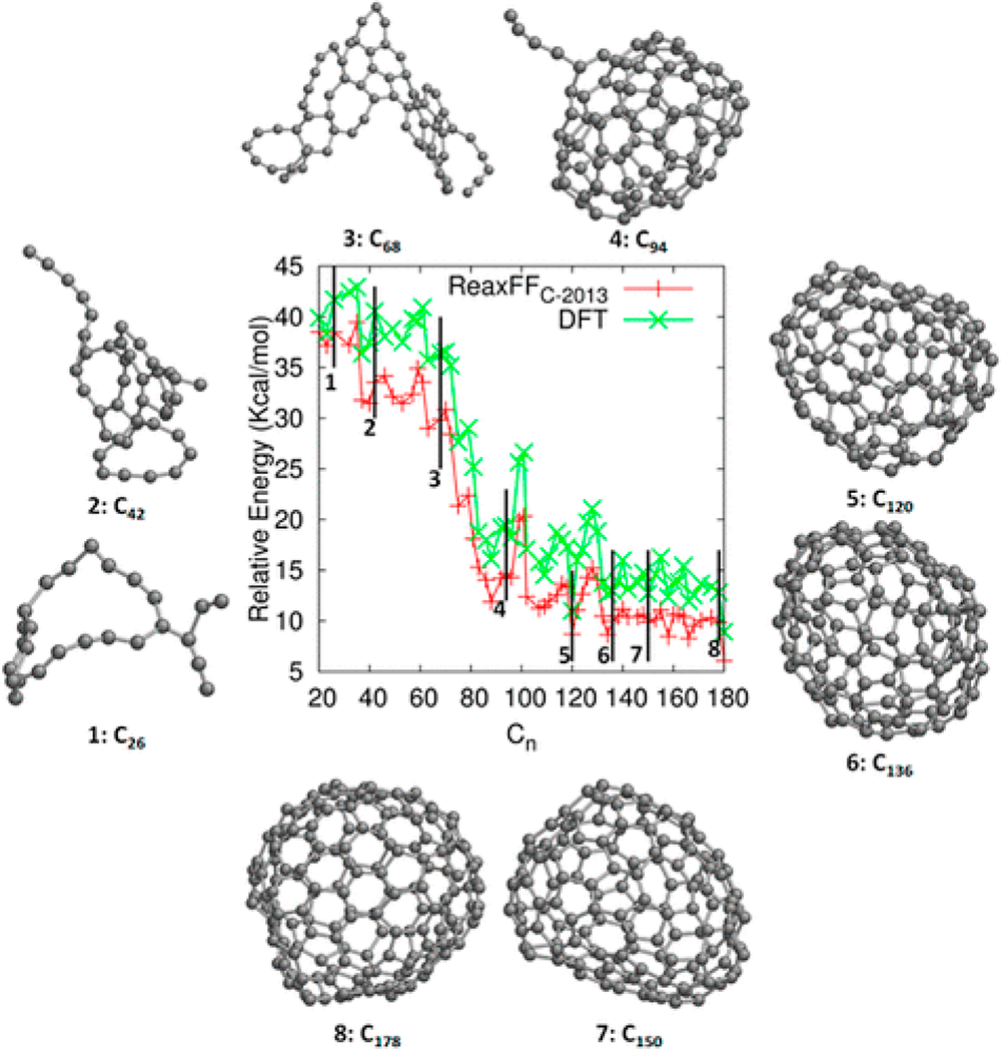
Structural evolution of the thermal decomposition of a C_180_ fullerene, simulated by Srinivasan et al.^319^ with the ReaxFF. Each structure corresponds to the state indicate in the plot. Reproduced with permission from ref (319). Copyright 2015 American Chemical Society.

Srinivasan et al.^319^ developed a new parametrization of a reactive force field (see section 2.2.1.2 for details) to study CND formation from large fullerene molecules (C_180_) at high temperatures. The developed parameter was then used to study the evolution of the fullerene molecule during MD simulations performed at various temperatures ranging between 4100 and 4500 K as illustrated in Figure 18(1–8).

In all cases the simulations resulted in the decomposition of the C_180_ fullerene into smaller fullerenes with many topological defects through loss of small fragments and later into an amorphous phase. In particular, the authors were able to observe that the overall fullerene thermal fragmentation follows an exponential function of time with a corresponding activation energy of 7.66 eV for the loss of carbon atoms from the fullerene.

In a very recent work, Kang et al.^466^ studied at the atomistic level the formation mechanism of GQDs and amorphous carbon materials by means of pulsed laser fragmentation in liquid (PLFL) using a combination of experimental (XPS, TEM, and HRTEM) techniques and MD simulations with a reactive force field potential and DFT calculations. The PLFL technique is considered as a simple top-down, clean, and relatively cheap process and an alternative to the bottom-up route for synthesis of different nanoparticles. The main result of Kang et al.’s investigation is their identification of a specific threshold for the formation of GQDs being strictly dependent on pulse laser energy. The authors were able to monitor the formation mechanism of GQDs from MWCNTs at a different pulse laser energy. Moreover, their atomistic calculations were able to describe the formation of GQDs during the PLFL process. In particular, the MD simulations revealed that the increase of the surface temperature (due to the injected pulsed laser) in MWCNTs promoted bond-breaking and morphology changes, leading to the formation of GQDs. By increasing the applied pulse laser energy beyond a threshold value, the formation of amorphous carbon structures becomes favored.

To sum up the reported work on nondoped CNDs’ models, the calculated optical absorption in graphene-like models is observed to red shift as the size of the layer increases, suggesting a presence of quantum confinement as seen previously for the inorganic QDs.^27,458^ These results are usually achieved with simple single layer PAHs, typically in single reference DFT and TDDFT calculations. For *n*-layers more reliable multireference methods should be used to better evaluate the optical features, for example, to identify the transitions defining the electronic character of the excited states.^112,114,160,236^ However, as the size of the simulated system increases, it becomes necessary to use less sophisticated methods (i.e., semiempirical) as for the disordered CNDs, allowing one to calculate the size effect.^79^ Results from these large disordered systems^27,79^ also indicate that beside the quantum confinement effect, other aspects should be considered to reproduce the optical properties of CNDs, such as geometrical deformation from the ideal flat graphene-like single layer or contribution of the sp^3^ fraction.^27,82^ The overall effect of geometry on optical features is further described in section 3.5.

### 3.2 Functionalized and Doped CNDs

Several studies have shown how doping the CNDs with different elements such as nitrogen, sulfur, and boron affect the fluorescence properties of CDs. Heteronuclei are known to affect the optical properties both when added to the edges/external surface as functional groups or included in the inner region of the CNDs as dopant. An increasing number of investigations have been using molecular modeling either to understand how the functionalization or the doping tune the optoelectronic activity of CNDs^10,12^ or to help rationalizing the best experimental synthetic procedure. Indeed, tailoring the band gap size has a number of technological motivations in the optoelectronics field, beside the fundamental research effort to understand the mechanism promoting such a tailoring.

The effect of functionalization on emission properties is an issue shared by CNDs and GQDs; the boundaries between the two systems become very pale when nanoparticles of less than 10 nm in diameter size are considered, at least from the optical properties point of view (see, for example, Cayuela et al.^467^). One could argue that GQDs are constituted by one or a few layers of graphene with similar planar dimensions, in a disklike configuration, while CNDs are due to the superimposition of various carbon-based layers with ranging diameters to form a spherelike structure. It is not surprising that from the theoretical ground, the two systems, often reduced to the study of one or two single graphene-like layers, share common features, prompting us to include in this section papers concerning the characterization of GQDs, similar to what is done for section 3.1 devoted to nondoped/functionalized CNDs or in section 3.6 dedicated to the interactions with other chemical systems. As in the previous section, technical details on the theory level employed in the review’s studies can be found in section 2.

#### 3.2.1 Oxygen

As a consequence of the method of preparation in solution, even the nondoped CDs are not solely composed of carbon. Their surface usually comprises hydrogen and very often oxygen-containing functional groups, such as the carboxyl, carbonyl, hydroxyl, or epoxy groups. The amount and nature of these groups depends on the synthesis method and can be used to tune not only the PL properties of the CNDs but also other relevant properties as the solubility or biocompatibility. The effect of oxygen on the CNDs properties depends on the nature of the specific functional group and on its amount, which in turn depends on the synthesis procedure. Several studies have been dedicated to understanding these relationships.

##### 3.2.1.1 Carboxylic Acid and Ester, Hydroxyl, and Epoxy Groups

Holá et al.^39^ introduced a simple bottom-up approach to synthesize hydrophobic and hydrophilic CNDs via thermal treatment of three different gallate molecular precursors. They first obtained organo-dispersible CNDs from three gallic acid derivatives characterized by different alkyl lengths which not only regulates the lipophilicity of the resulting particle but also its size. By converting the alkyl chains on the CNDs surface to carboxylate groups via base hydrolysis water-dispersible CNDs could be obtained. The PL measurements indicate that emission due to the core predominates in organo-dispersible CDs.

The carboxyl groups in the water-dispersible CNDs act as emissive surface traps. To provide a basis to understand these phenomena, TDDFT calculations were performed on a simplified model for CND structures (coronene, hexabenzocoronene functionalized with a carboxylic acid or ester group) aimed at reproducing the properties of the carbon sp^2^ parts of the core of the particles and the functional groups at the surface. The calculated emission maxima values show that a red shift is related both to the attachment of carboxyl groups to coronene and the increase in size.

**Figure 19:**
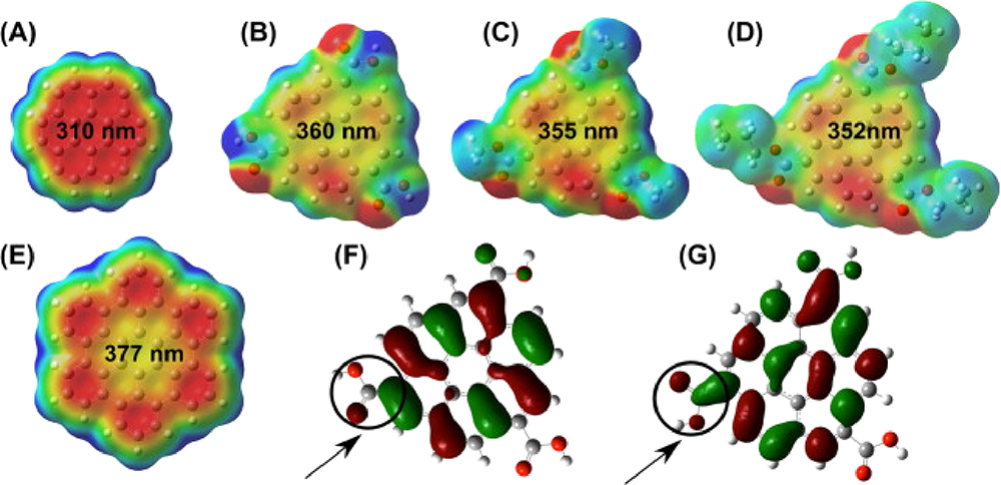
(a–e) Electrostatic maps and calculated emission maxima of different coronene-based models obtained at the B3LYP/6-31G(d,p) level of theory (the red color is the most negative charge, the green color is the neutral charge, the blue color is the positive charge). (f) HOMO and (g) LUMO orbitals of tricarboxycoronene with highlighting of the transfer of the electronic density to the carboxyl group after excitation (the red color is the positive value, the green color is the negative value). Reproduced with permission from ref (39). Copyright 2015 Elsevier.

These effects are correlated to an extension of the system of π-conjugated electrons, as it was observed in the case of tricarboxycoronene, whose highest HOMO (Figure 19f) are mainly positioned on the coronene core while LUMO (Figure 19g) are related to carboxyl groups conjugated with the coronene π-electrons. Moreover, the calculated electrostatic potential surfaces (Figure 19a–e) show how the addition of carboxyl groups shift the charge toward the edge and this can justify a bigger contribution of these functional groups in the PL mechanism.

**Figure 20:**
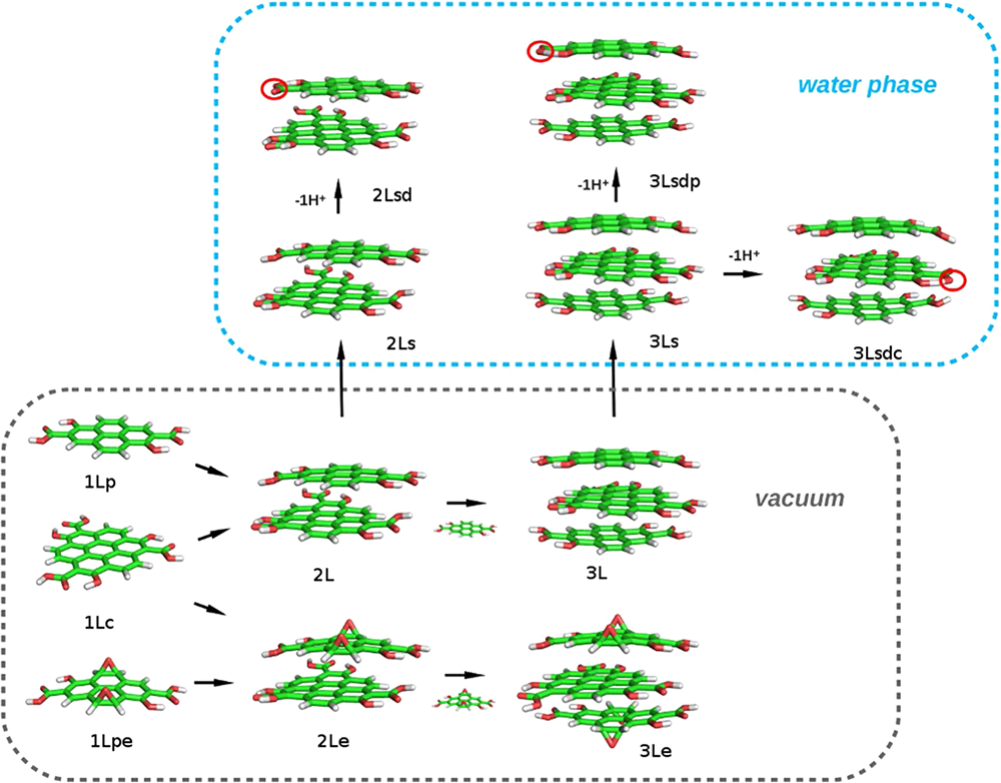
Schematic representation and labeling of the multilayer systems analyzed in Sudolská et al.’s work.^48^ 1L, 2L, and 3L stands for the one-, two-, and three-layer models, respectively. The suffixes -p and -c refer to the type of PAH-based unit adopted: pyrene or coronene. Moreover, the water environment was indicated by adding -s, while -d indicate the deprotonation of the system. Finally, the letters -dc and -dp for 3L models mark the deprotonation of the surface in the first case and of the middle layer in the second one. Structures are optimized at the B3LYP/6-311++G(d,p) level of theory. The green atoms are C, the red atoms are O, and the white atoms are H. Reproduced with permission from ref (48). Copyright 2015 American Chemical Society.

The following investigation by Sudolská et al.^48^ extends the focus to other oxygenated groups, (hydroxyl, carboxyl, and epoxy) studying model CNDs as single (1L) or multilayers (*n*L) of coronene and pyrene functionalized with the oxygenated groups, see Figure 20. A TDDFT study of these models was focused on the overall and specific contributions to the UV–vis spectra of the functional groups, stacking interaction, and solvent effect.

The calculated UV spectrum of similarly functionalized pyrene and coronene are shown to be largely different, with the former having three separate peaks, at 256 nm (strong) and 326 and 420 nm (broad and weak). That of functionalized coronene has two close peaks at 305 and 288 nm.

The bilayer model obtained from the two molecules is clearly stable both in vacuo and in water, and the stacking is associated with charge transfer processes, which impact the UV absorption band peak at 300 nm. The overall spectrum displays clear analogies with that of the corresponding monomer, with the main absorption peak inherited from pyrene red-shifted and broadened at 262 nm. Interlayer interaction, however, is weak, as proved by the fact that stacking distance observed (0.34 nm) is comparable with the graphite’s one (0.334 nm^468^) and by calculation of transition dipole coupling’s energy, which led to Förster coupling energies of 550 and 240 cm^–1^ for the two main peaks.

The UV absorption spectrum calculated on the CND model obtained by adding an extra pyrene to the bilayer reveals important differences with that of the constituent being dominated by an interlayer charge transfer transition at 300 nm and a very intense band at 254 nm (π–π* character). By analyzing the molecular orbitals, the authors reject the traditional assignation of the 300 nm band to an *n*–π* transition,^469−471^ observing, instead, a π–π* character. Thus, the inclusion of *n*-layers is very important to accurately describe spectroscopy data and to define the *n*–π* or π–π* character to transitions. Independently from the number of layers, the spectra are completely unaffected by deprotonation, both in vacuo and in water: the solvent contribution (water in this study) proved to be negligible in all models.

Functionalization via epoxy groups, by contrast, completely disrupts the UV spectra of the mono- and bi-layer systems. In the epoxidated pyrene, an enhancement and a strong blueshift of the peak due to the HOMO–LUMO transition from 420 to 332 nm is observed, while the previously brightest π–π* 256 nm transition, instead, is partially suppressed and red-shifted at 293 nm.

In the bilayer, epoxidation leads the fusion of the 254 and 300 nm bands observed in the absence of this group, into a single broad band, centered at 290 nm, in qualitative agreement with experimentally observed values.^472^ This phenomenon is explained in terms of interaction of the oxygen lone pair with the π delocalized CND’s cloud, which causes the orbitals’ intermixing and a partial disruption of the π conjugation.

##### 3.2.1.2 Ether Groups

The role of the edge ether groups in GQDs and CNDs optical properties was theoretically investigated by Chen et al.,^71^ following experimental evidence that self-trapped excitons play an important role in the GQD and CND optical properties.^473,474^ To this purpose, they employed DFT and TDDFT methods to predict the emission and absorption UV–vis spectra for carbon nanostructures in which ether groups are introduced at the center and the edges of the systems. To tackle the problem, both pristine and doped PAH of varying sizes were studied as reference.^473,474^ In pristine samples, both emission and excitation energies display the well-known decrease as dimensions increase, related to quantum confinement.^475−477^ Stokes shift in this case, however, is very small compared to the experimental data,^237,477,478^ a circumstance which is interpreted assuming that other excited states are involved in absorption and emission^479^ and that no significant structural modifications are related to the e^–^/h^+^ formation, excluding self-trapping in pure graphene monosheets.^480,481^

To model the effect of the ether groups, a circumcoronene model was used, adding the oxygen atom in the more favorable edge or inner position. The binding position strongly influences the simulated UV spectrum; while in center-oxidized samples, the absorption and emission energies, AE and EE, are almost identical to the pristine values, in edge-oxidized sample a clear increase in AE and decrease in EE is observed as oxidation degree progresses, leading to Stokes shift comparable to the experimental values (0.53–1.16 eV).^473,482,483^ This fact is interpreted by authors as the proof that structural changes sufficiently strong to induce exciton self-trapping can happen only if ether are at the edge. Moreover, edge functionalization activates the forbidden S_1_ → S_0_, as others proposed.^484^ Evidence of the localization is given by analysis of the electron–hole distribution: after excitation from S_0_ to S_1_ state, in fact, the electron and the hole densities localize close to one or two oxygen atoms of cyclic ether rings at the edges. Notably, a planarization of the ether rings is observed following this transition, which spatially enlarges the aromatic conjugation, stabilizing the excitons.

##### 3.2.1.3 Carbonyl Group

Compared to other oxygenated or nitrogen containing functional groups, the carbonyl group have a different effect on the tuning of the energies of the frontier orbital. In Li et al.’s work,^68^ the effect of several functional groups is evaluated by a three step procedure involving DFT, the GW method, and the Bethe–Salpeter equation (BSE) on a circumcoronene model (see Figure 9c,e,l).

By analyzing the HOMO and LUMO energies as obtained from DFT calculations, authors noticed that functional groups can be divided into two groups: the ones containing a carbonyl group C=O (CHO, COCH_3_, COOH), leading to a lowering of HOMO and LUMO energies, and the ones without C=O bonds (CH_3_, NH_2_, OH), causing a rise of the HOMO and LUMO energies. In any case, since the energy shifts are different for HOMO and LUMO, a general reduction of gap is observed.

In C=O containing groups, the LUMO shift is significantly more prominent than the HOMO’s one, while the reverse happens if C=O is absent. This is justified by authors by invoking the spatial extension of π conjugation of the system by injection of electrons from C=O orbitals.^485^ In addition, by direct inspection of the molecular orbitals, it was shown that LUMO’s orbitals are less hybridized than HOMO’s orbitals if C=O is absent, while the reverse happens if C=O is present. In particular, the larger the hybridization, the larger the gap reduction.

The inclusion of a GW many-body correction significantly modifies the observed HL gaps. These differences are explained in terms of the induced changes in the electron densities because all R groups examined (with the only exception of CH_3_, used as reference) are electron-acceptors: this implies that large charge transfers take place between the site of functionalization and the carbon backbone of the system, leading to a reduction of electron screening which, in turn, is responsible for an enhancement of electron–particle interactions.^68,486,487^

This is not the case of the CH_3_ group, which donates a very tiny fraction of charge, and is characterized by the lowest quasi-particle correction. This work shows how the hybridization of the atoms contributing to the frontier orbital and charge transfer are clearly in competition in determining the HL gap, with the former leading to its reduction and the latter to its increase. It is also clear that functional groups imply large hybridization but low charge transfer required to close the HL gap. Unfortunately, this is not the case of NH_2_, in which the large charge transfer almost cancels out the effect of hybridization: the CHO group, instead, seems to be more recommendable for this purpose.

To explain an apparently contradictory behavior for exciton binding energies, Li et al.^68^ proposed frontier orbitals hybridization and charge transfer no longer acting in competition but producing a collaboration which enhances optical activity: a large degree of hybridization leads to an activation of low lying states (dark excitons), while charge transfer is responsible in the stabilization of activated states by increasing binding energies.

**Figure 21:**
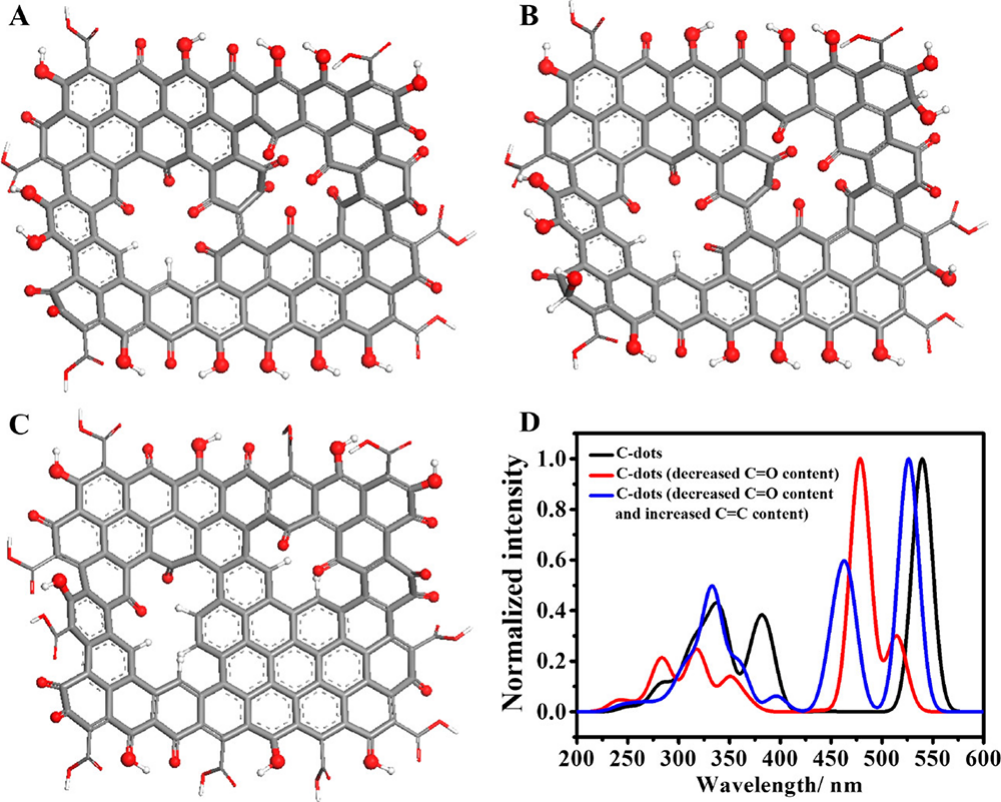
Adopted models by Liu et al.^78^ for oxidized graphene layers before (a) and after NaBH_4_ (b) or NaOH (c) reducing agents treatment. The corresponding optical properties (d) for pristine (black line), NaBH_4_ treated (blue line), and NaOH treated (red line) systems are calculated using PM6/UNO-CIS sempiempirical method and explained in terms of the different C=O and C=C content in parts a–c. Reproduced with permission from ref (78). Copyright 2019 American Chemical Society.

Another recent investigation from Liu et al.^78^ is dedicated to the understanding of how surface carbonyl groups and the π-electron system are coupled and contribute to determine the spectroscopic feature of CNDs. Since the size and degree of surface oxidation, by means different carbon–oxygen systems (C–O, C=O, COO−), affects the CND energy gap but the contribution of each oxidation result is unclear, the authors proceeded through different experimental chemical methods to tune the oxygen functional groups on the surface of 2 nm CNDs. NaBH_4_ and NaOH were chosen as reductants to reduce carbon dots prepared from the oxidation of carbon fiber powder in a nitric acid solution. The reduction process revealed that the emission wavelength of CNDs is strongly related to the extent of delocalization of π-electron system while carbonyl groups largely affect the emission QY, the two mechanism being coupled and producing opposite shifts of the emission peak. To corroborate the experimental results, theoretical calculations were performed modeling the CNDs as a single layer graphene model (2 nm × 2 nm) where the oxidation degree was randomly adjusted according to experimental relative content (accounting for surface C–OH, C=O, and −COOH groups). Some internal C=O groups were also included to balance the experimental oxidation degree gathered after NaBH_4_ reduction reaction. The optical absorption spectra were calculated by means of the configuration interaction with all single excitations. The theoretical calculations supported the coupling effect between the π-electron system and the carbonyl group on the PL of CNDs, as reported in Figure 21. Indeed, the absorption peak at about 540 nm in starting CND (in good agreement with the experimentally detected emission at 534 nm) was blue-shifted by 61 nm in the model system with decreased C=O content, while it is blue-shifted only 14 nm around the experimental value of about 526 nm when the C–C content is further increased.

#### 3.2.2 Nitrogen

Nitrogen-doped CDs are widely used in optoelectronic devices and in catalytic applications. The nitrogen content has been shown to largely affect the photoluminescence properties of CDs, being correlated with the increase of quantum efficiency of CDs and, to some extent, to their emission properties. Consequently, the synthesis and characterization of a wide variety of nitrogen containing carbon nanodots (N-CNDs) has attracted considerable attention, and quite a few investigations were combined with quantum mechanical calculations, aiming at understanding how the doping and functionalization with nitrogen atoms affect the optical properties. Several studies have been devoted to clarifying the role of the possible hybridization and bonding network of the nitrogen atoms. Among these, particular attention has been devoted to the N atom as a dopant embedded in the network of aromatic carbon atoms or being at the edge of the nanoparticle in pyridinic, pyrrolic, and amino groups.

##### 3.2.2.1 Graphitic, Pyridinic, and Pyrrolic Nitrogen

To elucidate the role of each N species, Otyepka and co-workers^41^ performed a systematic TDDFT study of one or two layers of pyrene- and coronene-based models. The attention was paid to the UV–vis optical properties extracted from HOMO and LUMO electronic levels and their electronic distribution. Importantly, it was found that graphitic nitrogen causes a red-shift of the absorption spectrum with respect to the undoped model. The optical transitions involve the highest occupied and lowest unoccupied orbitals (from HOMO – 2 to LUMO + 4), with a negligible excitonic coupling effect between the monolayer units.

**Figure 22:**
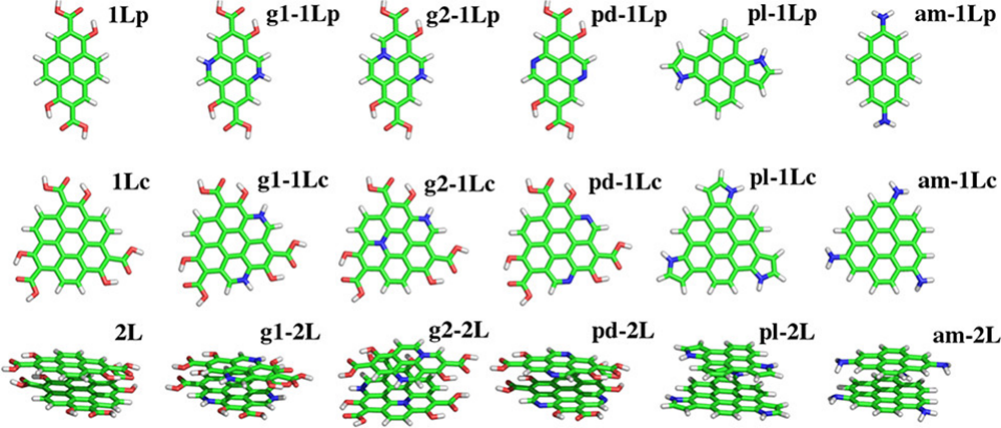
Model systems used to study 1L and 2L layers N-CNDs optimized at the B3LYP/6-31++G(d,p) level of theory by Sarkar et al.^41^ 1L and 2L stand for single- and double layer models, respectively. The suffixes -p and -c are referred to the type of PAH-based unit adopted: pyrene or coronene. Finally the prefixes g1/g2-, pd-, pl-, and am- indicate the presence of graphitic, pyridinic, pyrrolic, and amino units in the N-doped models. Reproduced from ref (41). Copyright 2016 American Chemical Society.

Similarly to what observed by Sudolská et al.,^48^ the two layers model (see Figure 22) produces results in better agreement with the experimental absorption spectra compared to the single layer model, indicating that the mean size of the π-conjugated regions in the experimental and theoretical CNDs is similar. Models with pyridinic, pyrrolic, and amino groups were predicted to have UV–vis absorption spectra like the undoped CNDs but for a slight blue shift in the pyridinic case. Although the focus of the work was on the nitrogen atom effect, the investigation suggested the assignment of the absorption band in the 400–430 nm range to OH/COOH electron donating/withdrawing groups and the absorption above 400 nm to COOH groups. Finally, since the excitonic coupling effect between the subunits is negligible, the absorption properties could be in principle predicted from the combination of the spectra of the single layers, at least for the oxygen containing computed systems.

**Figure 23:**
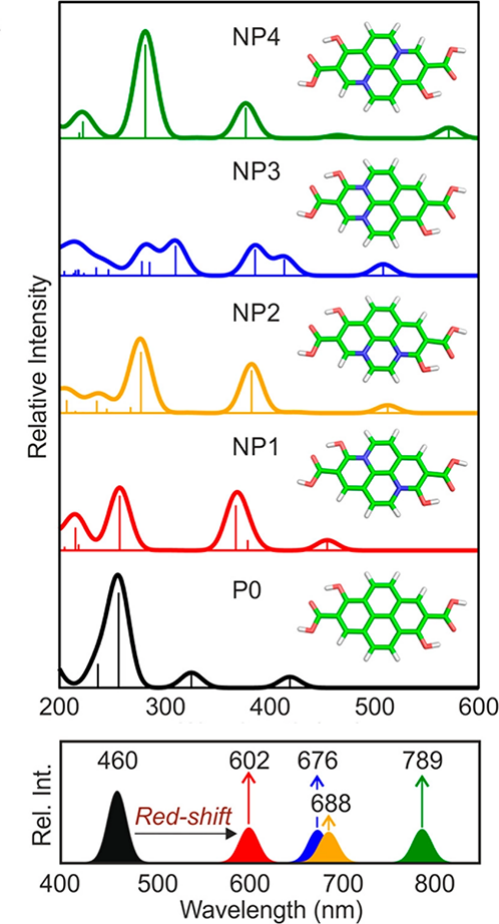
Nitrogen-doped models (NPx) used by Holá et al.^42^ and calculated UV–vis absorption spectra compared to nitrogen-free system (P0) of the same size (top): carbon (green), hydrogen (white), oxygen (red), nitrogen (blue). Model fluorescence spectra for the NPx models (colored) and nitrogen-free system P0 (black) (bottom). All spectra are calculated using the ωB97XD/6-31+G(d) combination of functional and basis sets. Adapted with permission from ref (42). Copyright 2017 American Chemical Society.

In the following investigation, Holá et al.^42^ explained by DFT calculations the role of graphitic nitrogen and how it can be exploited to trigger red fluorescence. As in previous investigations,^41,48^ the authors used a simplified model of the chromophores based on pyrene as illustrated in Figure 23. The calculated long-wavelength absorption of the most stable nitrogen-doped structure suggested that the experimentally observed red-shift was originated from the HOMO–LUMO gap narrowing due to the presence of graphitic nitrogen in the structure. In fact, it creates midgap states within the original HOMO–LUMO gap of the undoped system, which is a consequence of donated excess electrons into the unoccupied π* orbitals of a conjugated system. It must be noted that other chemical forms of nitrogen such as pyrrolic and pyridinic can cause the opposite effect, i.e., blue-shifted emission.

Interestingly, the models of CND chromophores were also useful to explain the trends observed in anion-exchange chromatography. Separation of the mixture of CDs with emission spanning the whole range of visible spectra was based on fractionalization by a negative charge. Surprisingly, this trend was not explainable by an increasing amount of negatively charged carboxyl groups, which were also included in their simplified model. For instance, the most negative fraction did not contain the highest amount of negatively charged carboxyl groups. This also demonstrates the important role of doped nitrogen on the acidity of carboxyl groups. Indeed, the computed electrostatic potential map of a selected N-CND model indicated that a graphitic nitrogen doping resulted in a higher electrostatic potential on carboxyl oxygens, which causes easier deprotonation and hence increased acidity of carboxyl groups with respect to the nitrogen-free model. The balanced charge distribution in the latter model is disturbed by the introduction of graphitic nitrogen atoms bringing two excess electrons into the conjugated system, which are pulled toward the more electronegative oxygen atoms of the carboxyl groups. In turn, the anion-exchange resin binding ability of graphitic nitrogen rich N-CNDs was significantly higher than that of CNDs with lower levels of graphitic nitrogen. This finding explains why the particles with red fluorescence and the highest amount of graphitic nitrogen were eluted by concentrated HCl as the very last fraction.

Shao et al.^240^ considered N-doping in the archetype pyrene molecule used as a reference for PAHs and CNDs in general. Three different types of N-doping, graphitic, graphitic-edge, and pyridinic, with two symmetric N atoms were considered in using the SR and MR methods to compute the changes in the absorption spectrum and compared to those in undoped pyrene. The authors propose a simple classification scheme based on VB theory and the Clar sextet rule in combination with the harmonic oscillator as a measure of the aromaticity (HOMA) index and verify if the scheme is useful to rationalize the electronic properties of different N-doping. In particular, the scheme allows one to explain the largest calculated red shifts of the absorption features as related to the formation of diradical VB structures paired with Clar sextets. Whereas the graphitic and graphitic edge doping, thanks to the increase of the π electrons, allows red shifting, pyridinic doping does not, and the absorption spectrum is rather similar to the undoped pyrene case. Depending on the position of the two doping N atoms, a large red shift is calculated for graphitic doping, up to 2.2 and to 3.2 eV. In the graphitic-edge doping case, a larger variation is observed, the LUMO excitation level being both slightly blue-shifted and largely red-shifted. As previously reported,^236^ the MR methods guarantee a correct estimation of the double excitation character of low-lying excited states, possibly neglected by the SR methods, thus further promoting the use of the DFT/MR level of theory when computationally affordable.

**Figure 24:**
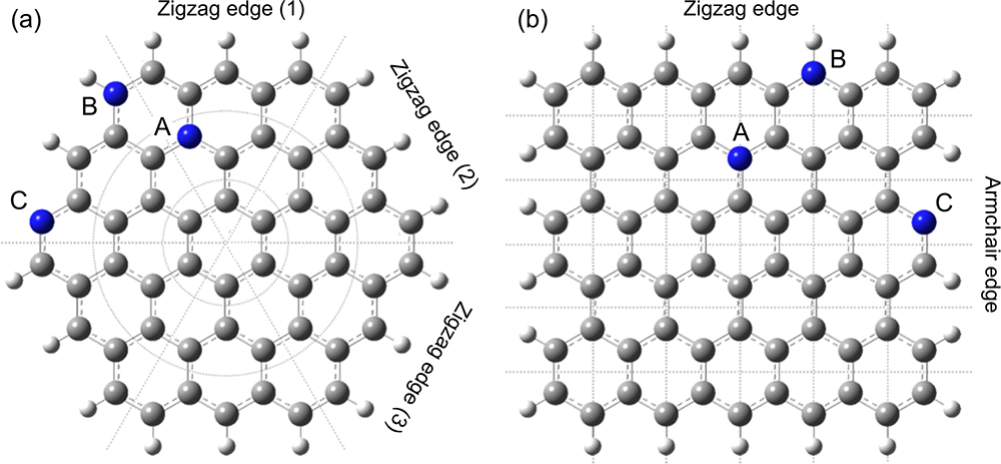
N-doped graphene layers, with both internal (A) and edge (B,C) substitution, optimized using B3LYP/6-31G(d) by Lin:^30^ (a) circumcoronene and (b) a rectangular model. Adapted with permission from ref (30). Copyright 2018 Wiley-VCH.

Lin^30^ compared the effect of N-doping on graphene layers of a hexagonal form (i.e., circum-n-coronene) and a rectangular form of varying sizes. As shown in Figure 24, while the former shape (Figure 24a) has only “zigzag” edges; in the latter (Figure 24b) there are also “armchair” edges, which can result in different preferential edge locations of N dopant atoms.

Three types of N dopants were considered by Lin (Figure 24): (A) graphitic substitution inside the nanographene, (B) graphitic substitution at the edge, i.e., edge N–H group, and (C) edge pyridinic-type. The DFT computed formation energies of a variety of models, including single and double N substituted layers, revealed that substitutions at the edges are favored over internal sites and that zigzag edge substitution is favored over armchair edge locations. Moreover, N–H edge doped layers were found to be generally more stable than those with pyridinic-type edge dopants, apparently contrary to recent experimental findings indicating the latter as the predominant form of N dopants given sufficient growth time,^488^ therefore, requiring further attention. Interestingly, pyridinic-N edge doped layer formation energies were found to be largely independent of the number and positions of substitution. Finally, in the case of multiple doping with N atoms, the models in which N atoms were separated, e.g., located on opposite edges, were more stable.

TDDFT calculations revealed that the UV–vis absorption spectra of pyridinic-type N-doped graphene layers are largely insensitive to the number and position of N atoms, showing a simple size-dependent trend due to the quantum confinement effect. According to this finding, the pyridinic-N dopant positions could likely neither be controlled or exactly determined from absorption spectra. Conversely, the other N-doped models exhibited highly diverse, nearly fingerprint-like spectra.

**Figure 25:**
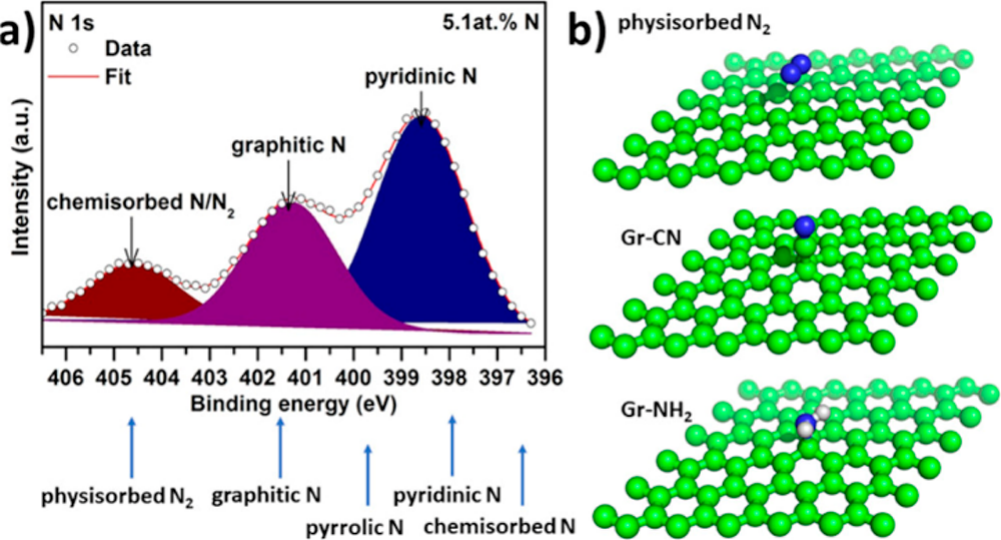
XPS simulated spectra of the CND model with different N-dopings from Lazar et al.^59^ Properties calculated using the PAW–PBE level of theory. Reproduced with permission from ref (59). Copyright 2019 American Chemical Society.

In their recent paper, Lazar et al.^59^ propose a computational study of different N-doping in nanographene (Figure 25b) showing that the best spectroscopic fingerprints, among the calculated IR, Raman, and XPS features (Figure 25a) are offered by the last technique, which allows discriminating among graphitic, pyrrolic, pyridinic, and chemisorbed nitrogen. IR, Raman, and XPS features were computed by exploiting Born effective charges, derivative of polarizability, and total energy differences, respectively. The main distinguishing IR vibration of graphitic N with respect to the other forms is calculated at about 1340 cm^–1^, unfortunately, in the same range as the well-known D band, attributed to structural defects and other disordered structures on the graphitic plane. It was also reported that the single layer considered as a model for graphene was not large enough to produce the 1600 cm^–1^ vibration typically ascribed to skeletal ring vibrations in graphene system doped with graphitic N. In the case of pyrrolic, pyridinic, and chemisorbed N, a peak at about 1610 cm^–1^ was calculated, almost coincident with the D′ band due to intravalley double-resonant scattering process. As for the Raman modes, no distinguishing features can be retrieved, the vibrations being calculated for all the computed systems in the 1500–1600 cm^–1^ range but for the mode at 1339 cm^–1^ calculated for graphitic N doping. Those modes do not allow fingerprinting the doping because of the overlapping of the pure graphene G (due to carbon atoms movement in the graphene plane), D and D′ bands. On the contrary, fingerprinting is obtained with XPS calculations, since different forms of N have different calculated binding energies, at 401.5, 399.7, 397.9, and 396.6 eV, for graphitic, pyrrolic, pyridinic, and chemisorbed N, respectively. In addition, also physisorbed N_2_ can be distinguished by XPS, with a binding energy estimated at 404.7 eV.

##### 3.2.2.2 Amino Groups

**Figure 26:**
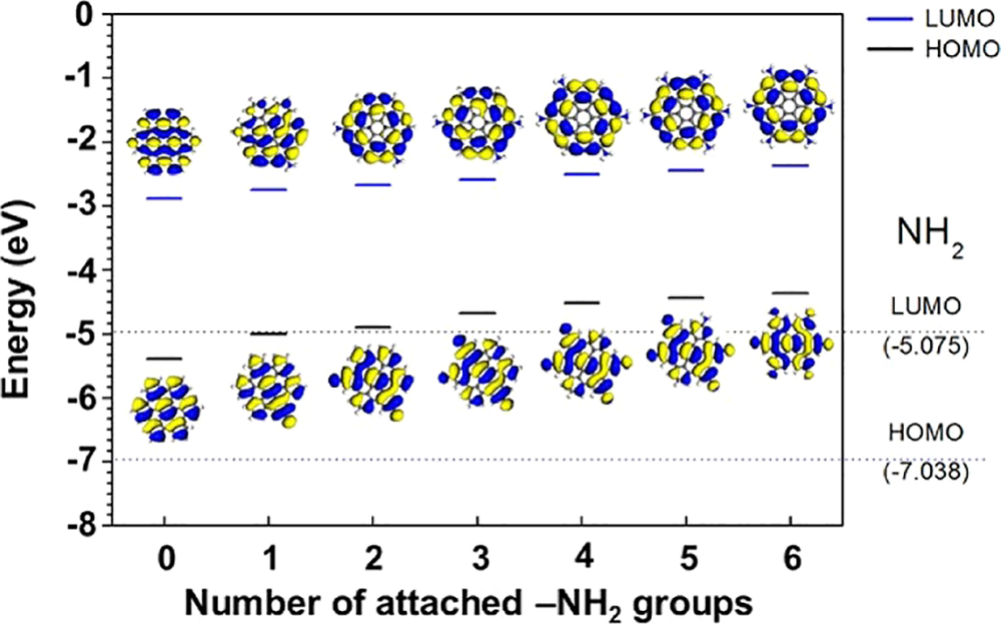
Effect on the HOMO and LUMO energies of the number of amino functional groups attached to the surface calculated using the LDA-PWC/DNP method. Adapted with permission of from ref (65). Copyright 2013 American Chemical Society.

Jin et al.^65^ analyzed the band gap tuning of GQDs by the NH_2_ functional group by combined experimental and theoretical investigations. Layers of graphene (1–3) were fabricated by a two-step cutting process starting from graphene oxides. The realized samples were functionalized with amine groups and compared with unfunctionalized GQDs. Functionalization slightly affected the measured ordered/disordered network ratio, evaluated by means of the above-mentioned G and D Raman bands, leading to a reduced value of *I*_D_/*I*_G_ ratio, a proof of the quality of the samples. Optical spectroscopy characterization on the other hand displayed that amine functional groups cause a red shift of the GQDs emission, associated with the charge transfer from amine to benzene rings. The experimental results were confirmed by DFT simulations carried out on a 13-ring cluster functionalized with amino groups at the cluster edges. The calculated band gap of the simulated systems decreases as the number of functional amino groups increases, as shown in Figure 26. The charge distribution and the HOMO and LUMO molecular orbitals of the optimized geometries agrees with the charge transfer hypothesis.

Also the work of Wang et al.^29^ is aimed at discriminating the effect of surface functionalization by NH_2_ groups. The comparison was carried out on two different graphene layer models, the 41-rings undoped GQD (Figure 9a) and the fully functionalized counterpart SF-GQD (26 NH_2_ groups, Figure 9n). The functionalization promotes a red-shift of the whole absorption spectrum, thus supporting the experimentally reported fluorescence red-shift. The analysis of the charge difference densities at the calculated excitation transition evidenced that the electronic transitions are localized excited states for GQDs, while they are charge-transfer excited states in SF-GQDs. In the latter, a large distortion of the model structure is also observed, leading to the delocalization of the electrons and a larger electron–hole separation responsible for the larger fluorescence efficiency.

**Figure 27:**
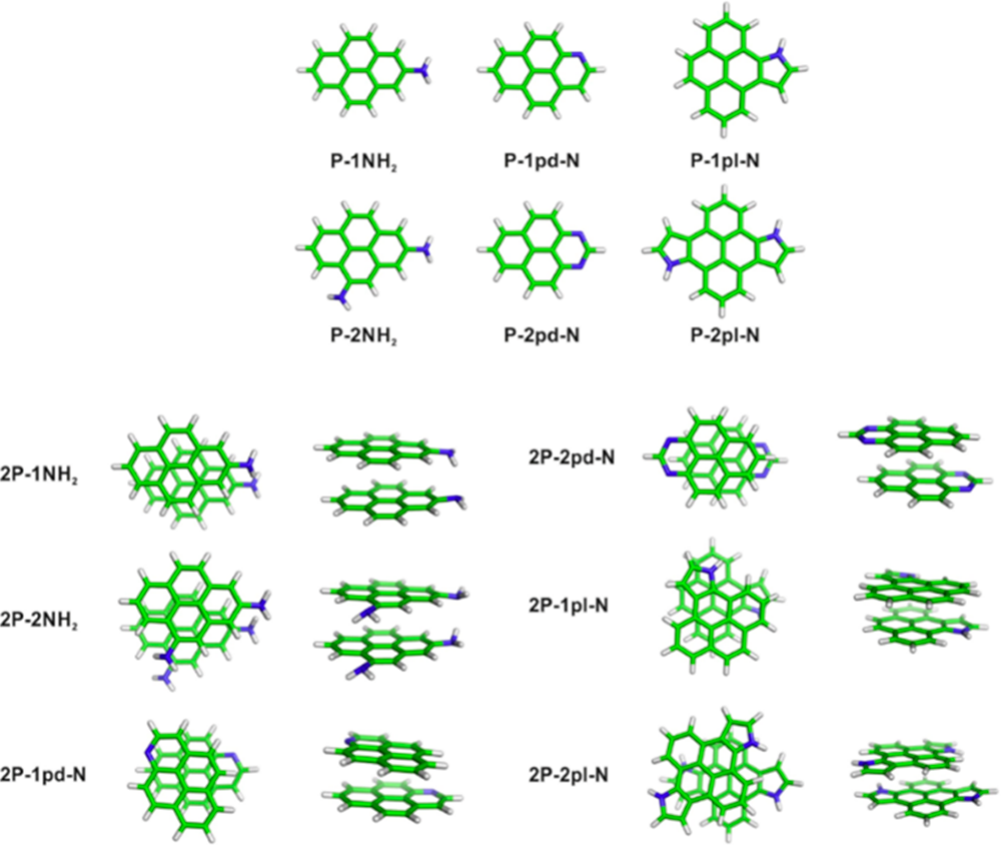
One- and two-layered model NCNDs used by Sudolská et al.^72^ All displayed structures are optimized at the ωB97XD/6-31++G(d,p) level of theory. Adapted with permission from ref (72). Copyright 2017 Elsevier Ltd.

In the following work, Sudolská and Otyepka^72^ present a systematic approach to model how absorption and emission spectra are affected by different chemical forms of nitrogen at the edge of the graphene layers. TDDFT calculations and Boltzmann averaging were applied on one and two layered CD models containing pyridinic, pyrrolic, or amine nitrogen in the pyrene-models representing the aromatic carbon domains within N-CNDs, see Figure 27.

The main advantage of the model system was that it enabled focus solely on the effects of nitrogen doping and systematic exploration of all possible isomers because the small model proportions correspond to a restriction to the uniform pyrene-like sp^2^ core that ruled out the effects of the size, shape, and edge structure of CDs.

The study of emission properties was conducted using the same approach only for the 1L systems, where they assumed that Kasha’s rule holds. The calculations of the 1L systems were performed both in a vacuum and water, modeled with a C-PCM model, while the 2L systems was modeled only in vacuo. The used approach was allowed to reach important conclusions on how the chemical environment surrounding nitrogen affects the optical properties of the N-CND. Independently from the solvent medium (vacuum or C-PCM), the amine pyrene models displayed appreciably red-shifted absorption and fluorescence compared to the pyridinic and pyrrolic models. Furthermore, increasing the nitrogen content leads to an increase in the red-shifting with amine groups and in the blue-shifting with pyridinic and pyrrolic groups. Therefore, a synthetic procedure that can balance properly the different nitrogen chemical form can allow to obtain red emission. The study of the 2L systems allowed verifying that the studied optical properties are only slightly modulated by the exact arrangements/conformations of individual components in multilayered CNDs. This does not mean that a single layer approach would suffice to model the absorption/emission of the CNDs, and indeed the authors highlighted that when higher excited states are involved the stacking might play a role, at least when considering edge N-doping.

**Figure 28:**
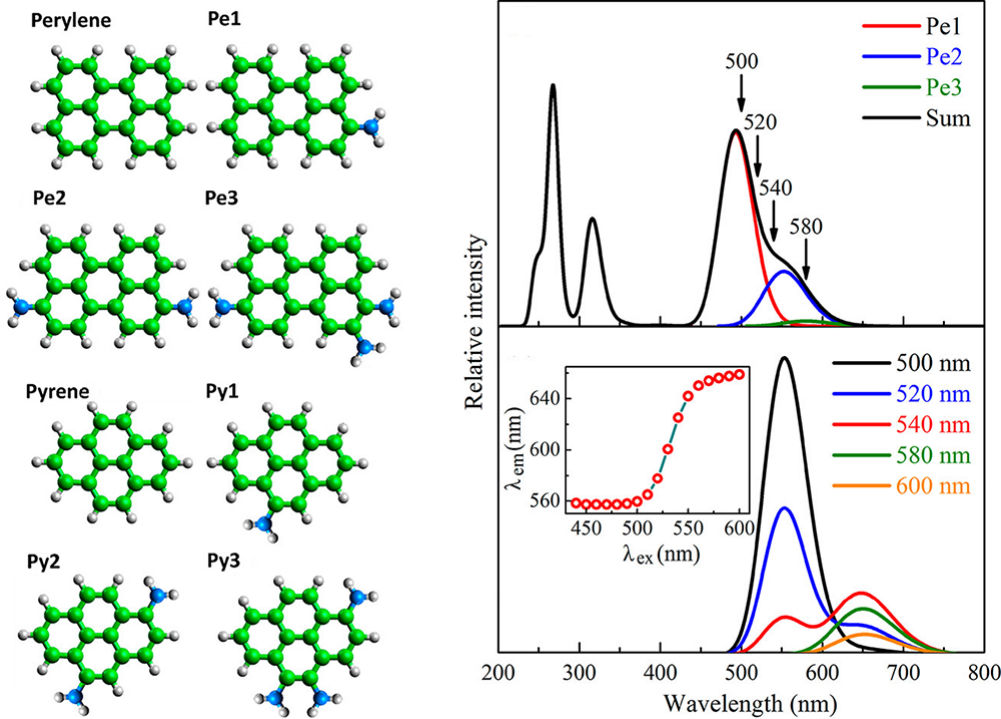
Subunits of polycyclic aromatic hydrocarbons with one, two, or three −NH_2_ groups used by Kundelev et al.^52^ to model the optical response due to the functionalization of the CDs’ surface with amino groups (left) and corresponding absorption spectra and spectral sum of them (right). Structures optimized using the DFT/B3LYP/DZ. Adapted with permission from ref (52). Copyright 2019 American Chemical Society.

The role of the amino groups is also at the focus in the recent work of Kundelev, Rogach, and co-workers^52^ that modeled the N-CNDs chromophores as perylene molecules and functionalized with one, two, or three −NH_2_ groups at the CDs’ surface, in order to explain the excitation energy dependence of the emission spectra. The authors analyze the optical features of CNDs in the long-wavelength region considering the average of the absorption and PL spectra of CNDs over the distribution of the degrees of functionalization that, for a broad variety of functionalized materials, is described well by a purely statistical binomial distribution (Figure 28). Their calculations revealed that a red shift of the absorption and PL spectra occurs when CNDs are functionalized with −NH_2_ groups, and this phenomenon increases with the degree of functionalization due to charge transfer from amino groups to the core of the particle. Moreover, different kinds of amino subunits on the surface lead to tunable PL depending on the excitation wavelength.

Finally, this study asserts that the presence of amino groups not only caused a red shift of the emission but also affects the emission efficiency by preserving the PL oscillator strength unlike other nitrogen forms which also red shift the fluorescence but suppress the oscillator strength of the first radiative transition.^41,42^

#### 3.2.3 Oxygen and Nitrogen

**Figure 29:**
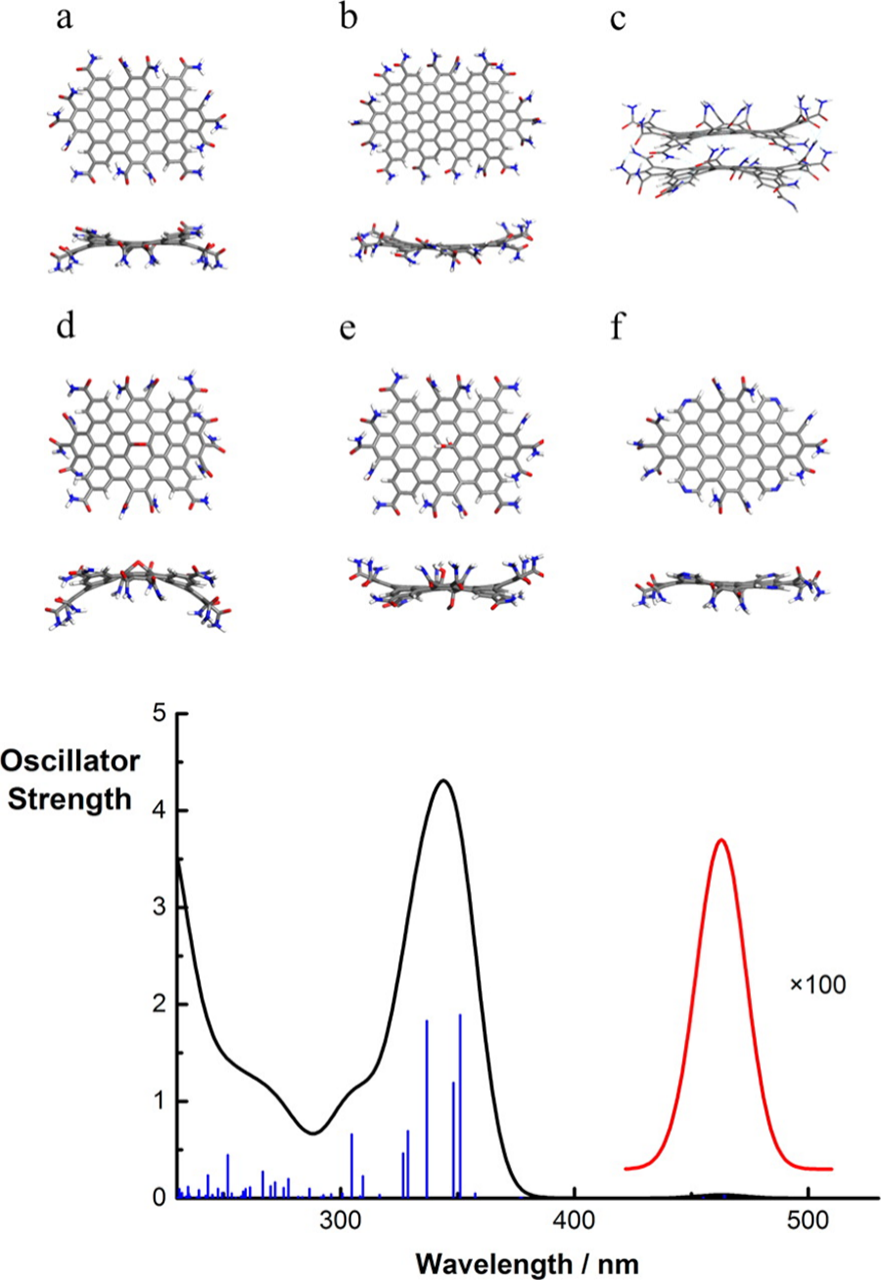
Model systems of CND used by Strauss et al.^67^ (top) and simulated absorption spectra (bottom). Parts a and b are differently sized amide-capped graphene models. From model a, the authors derived (c) dimer, (d) epoxy-, (e) hydroxy, and (f) pyridine-containing models. Structures are optimized at ωB97XD/def2-SVP (B97D in the dimer model), while absorption properties are calculated using the UNO-CI semiempirical method. Adapted with permission from ref (67). Copyright 2014 American Chemical Society.

In a seminal work, Strauss and co-workers^67^ investigated the structural and optical properties of microwave synthesized CNDs prepared starting from standard precursors, CZA and urea, under controlled pressure conditions (atmospheric and 15 bar). Disordered structures with no evidence of crystalline particles were reported irrespective of the synthesis conditions, suggesting the formation of nanosized heavily functionalized sp^2^ systems. On the contrary, the optical properties evidenced large differences, showing the typical excitation dependent blue emission in the case of atmospheric pressure synthesis, and a blue excitation independent molecule-like emission in the case of high-pressure synthesis. The optical spectroscopy analysis leads the authors to ascribe the reported features to trap states and intrinsic centers, with some interaction mechanism between the two. To study possible relationships between structure and optical properties, CNDs were modeled by means of DFT and SEMO methods on single and double layers of amide-capped graphene layers of different size. Different chemical modifications to the sp^2^ network were also considered to account for specific chemical origin of the observed optical properties (such as epoxidation, hydroxylation of a central double bond, pyridinic nitrogen atoms at the edges of the lattice). As reported in Figure 29, all the models were nonplanar, probably because of the large functionalization degree at the sp^2^ network edges by amide groups. The optical properties were calculated within the semiempirical UNI-CIS framework with a PM3 Hamiltonian. Simulated absorption and emission spectra (Figure 29), coupled with radiative transition rates, matched well the experimental results reported in the paper and allows one to infer some important indications. First, larger graphene layers are characterized by red-shifted optical features and increased radiative decay rates. Second, the formation of a bilayer also causes red-shift of the emission, while the absorption is blue-shifted. Epoxidation and hydroxylation have opposite effects, the former causing a blue shift of optical properties coupled with a large distortion of the sp^2^ network, the latter producing red-shift of the absorption and emission spectra. Finally, pyridinic nitrogen atoms at the layer edges causes a large blue shift of the spectral features and an increase of the radiative decay rate.

The luminescence spectrum, computed on multiple conformations of the bilayers as sampled with classical MD simulations, is qualitatively like the one of the experimental samples synthesized at higher pressure, thus assigned to conformational disorder instead of to the polydispersity of the observed large distribution of spectral features.

A close comparison of experimental optical features of pure and N-doped CNDs with different possible model structures was recently reported by Sheardy^77^ where relevant features of the TDDFT optical absorption spectrum were mainly assigned to the deformation induced by the doping of the graphitic structures, rather than to a specific functional groups. Experimental samples were synthesized from sucrose (undoped CNDs) and from a CA and EDA (N-doped CNDs) and fully characterized by AFM, optical spectroscopy, NMR, XRD, and XPS measurements. TDDFT calculations were carried out on several single layer graphitic structures, in which hydroxyl and carboxyl groups, Stone–Wales defects, epoxides, and primary, secondary, and tertiary amines were placed at the edges or within the inner structure. Four examples of the 12 studied structures are reported in Figure 29a–c,g). All the simulated structures show a large contribution at around 150–400 nm, resulting from π → π* transitions. The number and intensity depend on the structural disorder induced by the disruptive functional groups: as the distortion increases, the intensity of individual transition decreases, and new ones are allowed. In addition, the contribution of different amine groups produces different relative contributions to the 200–300 nm band but do not affect the structure planarity. The effect of different doping sites and N concentration requires further investigation but the reported results on amine doping suggest that close packing can be obtained in graphitic N-doped CNDs, as confirmed by XRD. The packing could be related to the larger efficiency reported for those systems. Finally, the excitation dependent trend typically shown by CNDs with low crystalline order is ascribed by the authors to the large density of energy levels near the Fermi level calculated in the simulated structures.

Srivastava et al.^28^ investigated the photophysical properties of hydrothermally synthesized CDs passivated with electron-accepting malononitrile and electron-donating *N*,*N*-dimethylaniline. They studied both bulk-state and single-particles features, considering the bulk-state as the ensemble-averaged measurements which result from the sum of individual contributions. They observed that at the bulk-state level, passivated CNDs show blue-shift emission along with an increase of the emission intensity. Moreover, in a mixture of both types, electron-accepting CNDs dominate the photophysical properties and brought to the formation at least two associated geometries, in the form of ring and rodlike complex structures. Nevertheless, at a single-particle level, observations do not report an “acceptor-dominating” scenario. For a better understanding of the phenomena, they evaluated electronic and emission properties of CNDs performing DFT calculations. They modeled CNDs using ovalene-based models: both bare and passivated CNDs present surface groups such as carboxylic acid and hydroxyl groups based on XPS measurements percentage of carbon and oxygen while *N*,*N*-dimethylaniline and malononitrile were added to the surface of donor and acceptor CNDs, respectively.

Calculations of HOMO–LUMO displayed that in a mixture of bare and passivated CNDs, CND-donor are capable of donating electrons to the other types of CNDs. Instead, in the presence of only bare CND and CND-acceptor, this last one can act as excited-state electron acceptors showing that CNDs can act either as electron acceptors or electron donors.

**Figure 30:**
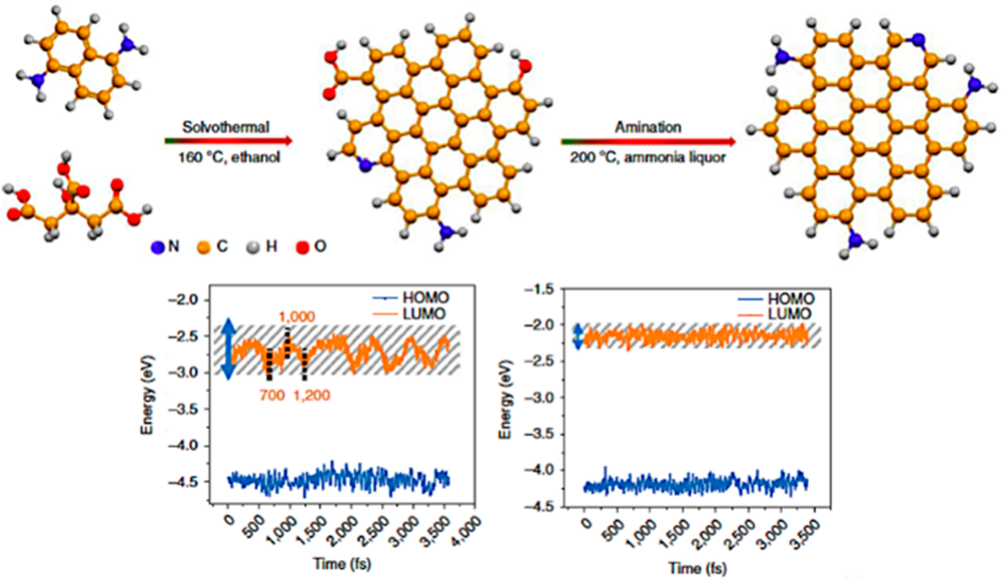
Scheme of the synthetic route to the nitrogen-functionalized graphene layers obtained from Yuan et al.^58^ together with their simplified models (top). Band gap fluctuations for the two models used in the BOMD (bottom). Structures optimized using DFT/PAW-PBE. Adapted with permission from ref (58). Copyright 2020 Springer Nature Limited.

Countertrend compared with the quest of red emitting CNDs, the paper of Yuan et al.^58^ deals with the production of bright high-color purity deep blue CDs for LED applications. The synthetic route to achieve this outstanding results in terms of efficiency and color was planned by means of DFT calculations to understand how the edge amination process could lead to blue-emitting CNDs. The presence of oxygen-containing functional groups on the edge of the carbon dot broadens its emission feature because of the molecular vibrational and the structure distortion. To reduce these vibrational contributions, the oxygen related groups, such as OH and COOH, could be substituted with NH_2_ amine ones. The model system consists of 13 fused benzene rings with different functional groups at the edges and was investigated by means of Born–Oppenheimer molecular dynamics (see Figure 30). By substituting the oxygen-containing functional groups with amine ones, the fluctuations of the excited state are largely reduced, thus suggesting that the oxygen groups are responsible for the broadening of the optical band also thorough the rotational freedom of these groups that affects wave function localization. The calculated results were verified by experimental measurements, showing amino functionalized CNDs having narrow and more efficient blue emission.

**Figure 31:**
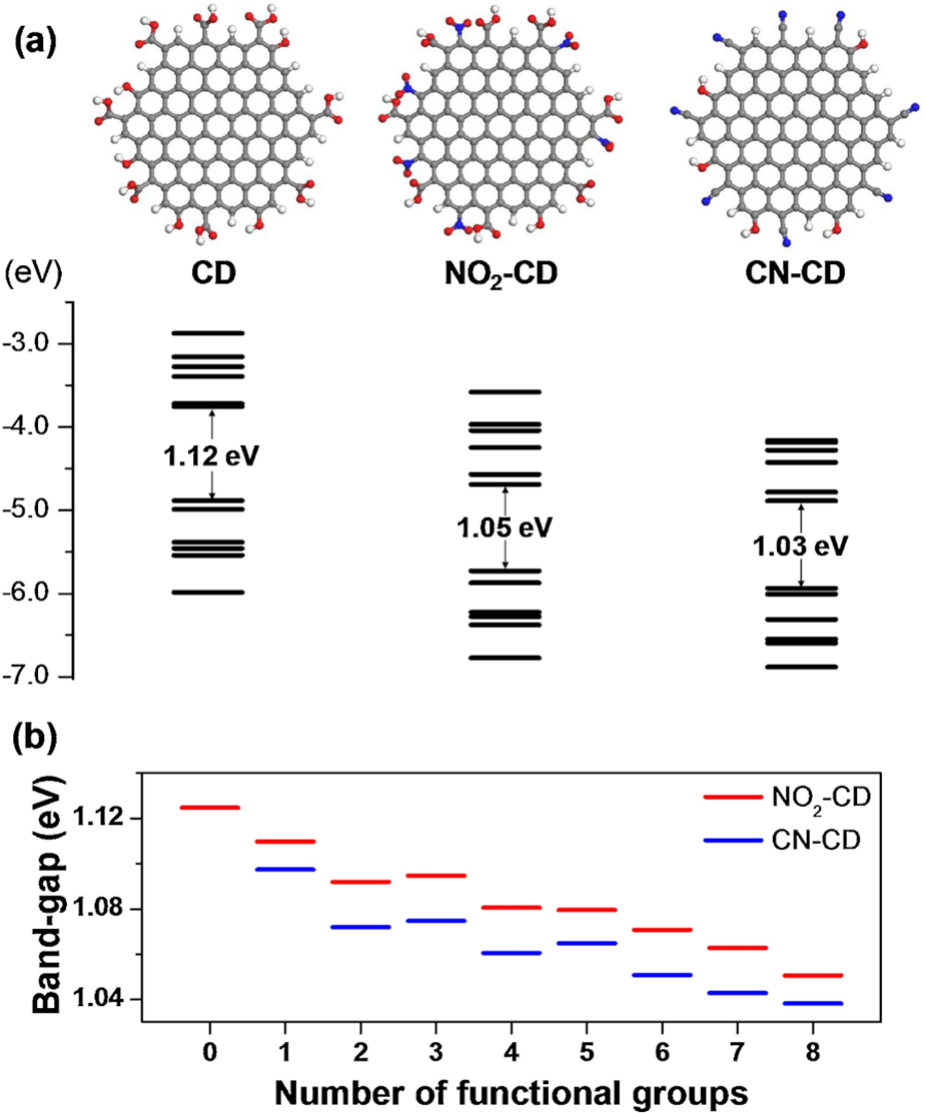
(a) Simple (left), nitrile- (center), and nitro-functionalized (right) molecular models and respective FMO energies used by Choi et al.^64^ to verify the shift induced by functionalization with electron withdrawing groups. (b) Variation of computed band gap as a function of the number of functional groups added to the model. Properties calculated at PWC/DNP level of theory. Reproduced with permission from ref.^64^ Copyright 2018 The Korean Society of Industrial and Engineering Chemistry.

In the work of Choi et al.,^64^ strong electron-withdrawing functional groups, such as −NO_2_ and −CN, were exploited to red-shift the absorption and emission features of CNDs. The synthesis of CNDs was achieved by chemical oxidation of graphite and hydrothermal reaction leading to the surface functionalization of CNDs with the graphene core. Structural, morphological, and optical characterizations revealed disklike systems consisting of 2–3 layers of graphene nanosheets with increasing, red-shifted optical properties from pure CNDs to NO_2_ and CN functionalized ones. A circum-2-coronene model (see Figure 31) was used to rationalize the finding with DFT based investigation, which revealed that the functionalization with increasing electron-withdrawing groups red-shifted the absorption spectrum of the system; the larger the functionalization, the larger the red-shift.

An important subset of CDs is constituted by graphitic carbon nitride (g-C_3_N_4_) QDs that exhibit more intense fluorescence and higher QYs compared to graphene and graphene oxide QDs,^489−491^ yet they possess many of the favorable properties for biomedical applications, e.g., good water solubility and biocompatibility, attributed to the latter QD types.

g-C_3_N_4_ materials are increasingly studied for their promising photocatalytic and photoluminescence properties.^492^ A considerable research effort has been devoted to enhancing the performance of CN-based materials; among these, the incorporation of carbon nanostructures by covalent bonding has shown much promise. However, detailed knowledge of the factors determining the photocatalytic/photoluminescence performance of such hybrid materials is currently lacking.

g-C_3_N_4_ polymorphs can be easily obtained by thermal condensation of nitrogen-rich sources like dicyandiamide, urea, or melamine. Stagi and co-workers^493^ studied by a combination of experiments and computational simulations the evolution of triazine units as a function of temperature, attributing the rise of a blue emission to the formation of heptazine monomers and polyheptazines with a subsequent redshift as the condensation proceeds. Moreover, the gradual fluorescence quenching of high-temperature treated systems was attributed to the marked interaction between contiguous layers, whose mutual distance reduces with the temperature, also confirmed by vibrational calculations.

**Figure 32:**
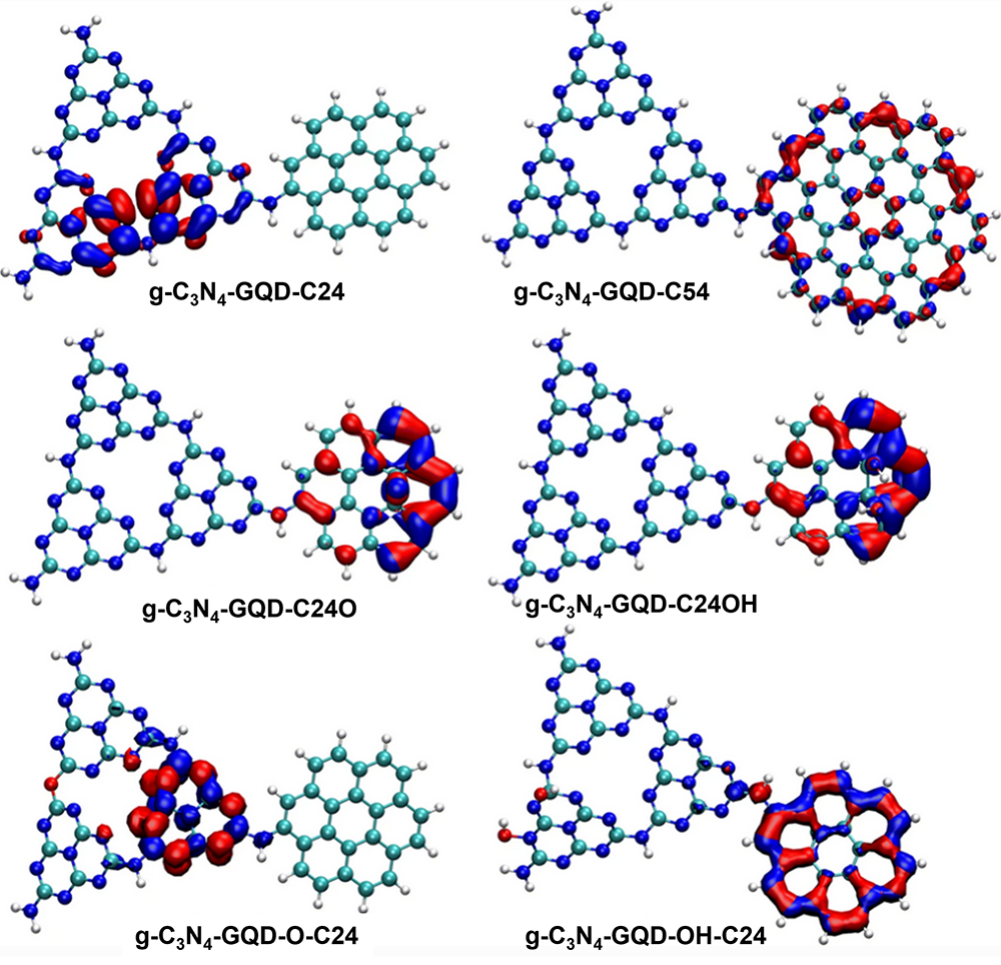
Model of the hybrid g-C_3_N_4_-GQD structures considered by Chen et al.^494^ showing the calculated S_1_ state minimum exciton distribution: red regions show the position of the electron hole, blue regions the photoexcited electron. Adapted with permission from ref (494). Copyright 2020 Springer Nature.

A significant redshift can be also obtained by introducing impurities or functionalizing the CN structure. In this context, Chen et al.^494^ recently reported a TDDFT theoretical study of the light absorption and photoexcited state characteristics of model covalently bonded hybrid structures of g-C_3_N_4_ QDs (CNQDs) and GQDs (Figure 32). The effects of introducing oxygen-containing surface functional groups were also investigated, in view of likely chemical reaction occurring under practical working conditions. CNQD-GQD hybrid structures were found to show GQD size-dependent light absorption red shift compared to that of the CNQD only; the larger the GQD component, the greater the red shift, consistent with the expected quantum confinement effect on optical gaps. The light absorption intensities of the hybrid structures were also found to be higher overall, indicating potentially higher optical efficiencies. Addition of GQDs to g-C_3_N_4_ is thus predicted by the authors to be a practical means of tuning the light absorption properties of these materials. Interestingly, model hybrid structures featuring oxygen-containing functional groups (epoxy, hydroxyl) showed considerable absorption spectrum differences, confirming that chemical reactions expected to occur under practical working conditions may indeed greatly change the optical properties of hybrid CNQD-GQDs in photocatalytic applications.

Chemical modifications to CN-based materials may also significantly influence electron–hole recombination dynamics, which is crucial to both photocatalytic and photoluminescence performances. In CNQD-GQD hybrid structures, the photoexcited electron and hole may localize on either the CNQD or GQD part, with electron–hole separation expected to lead to a low recombination rate which favors photocatalytic applications. Notably, no obvious electron–hole separation was found for the hybrid structures considered in this work. Nevertheless, incorporation of oxygen-containing surface groups was found to affect electron–hole distributions, highlighting the potential importance of unintentional chemical modifications occurring under working conditions in the photocatalytic/photoluminescence performance of these materials.

**Figure 33:**
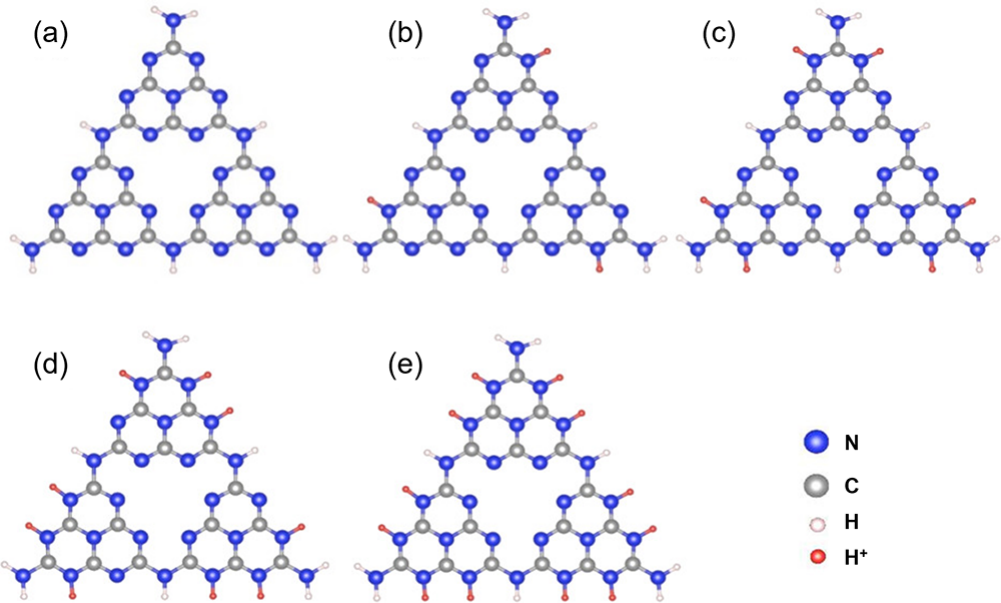
Optimized structures of of (a) neutral (pristine) and protonated g-C_3_N_4_-QDs with 3 (b), 6 (c), 9 (d), and 12 (e) H^+^ at its edges to model pH decreasing conditions. Optimization is performed by using DFT/B3LYP/TZP. Adapted with permission from ref (495). Copyright 2019 Wiley-VCH.

Interestingly, the photoluminescence spectra of g-C_3_N_4_ QDs have been found to be pH-dependent; however, apparently contradictory experimental pH-dependent tendencies have been reported.^478^ Zhou et al.^495^ performed a comprehensive theoretic investigation of the pH-dependent photoluminescence of g-C_3_N_4_ QDs using TDDFT and nonadiabatic MD (NAMD) simulations. Model g-C_3_N_4_ QDs, consisting of tri-s-triazine rings cross-linked by trigonal N atoms, were protonated at selected edge N atom sites in order to account for neutral and weakly and strongly acidic conditions (Figure 33). Enhanced light absorption was found for systems representing both weakly and strongly acidic conditions compared to a charge neutral model. However, the g-C_3_N_4_ protonation state was shown to significantly affect the competition between radiative and nonradiative electron–hole recombination by changing the transition channel orbital composition and frontier molecular orbital overlap. In weakly acidic media, nonradiative recombination is weak, resulting in a strong fluorescent emission; however, under strongly acidic conditions, the high degree of edge N protonation leads to fast recombination with a high nonradiative transition probability with subsequent reduced fluorescent emission. The proposed synergetic mechanism between light absorption and radiative or nonradiative electron–hole recombination was shown to explain the experimental pH-dependent photoluminescence tendencies of g-C_3_N_4_ QDs, and possibly of other 2D QDs containing lone pair electron sites. Finally, in view of the mainly aqueous environments of QDs and the likely inclusion of O-containing functional groups, the authors investigated the effect of additional edge hydroxyl functional groups on the pH-dependence of g-C_3_N_4_ photoluminescence. Interestingly, absorption and emission trends for g-C_3_N_4_ QDs under acidic conditions were found to be unaffected by the inclusion of hydroxyl groups.

Gu et al.^320^ synthesized nitrogen-doped graphene and graphitic carbon nitride quantum dots by means SPMA technique using as precursors CA and urea. The corresponding synthesized quantum dots show a homogeneous particle size and a general circle and/or ellipse shape that minimizes the surface free energy.

The nitrogen/carbon (N/C) ratio at the surface shows values up to an unprecedented value of 1.74. The distance between the layers within the g-C_3_N_4_ crystalline configuration depend on the relative amount of CA and urea used. The observed distances of (0.330 and 0.320 nm) are smaller than that in pure graphite, due to the presence of the N dopants which reduce the interlayer spacing distance.

**Figure 34:**
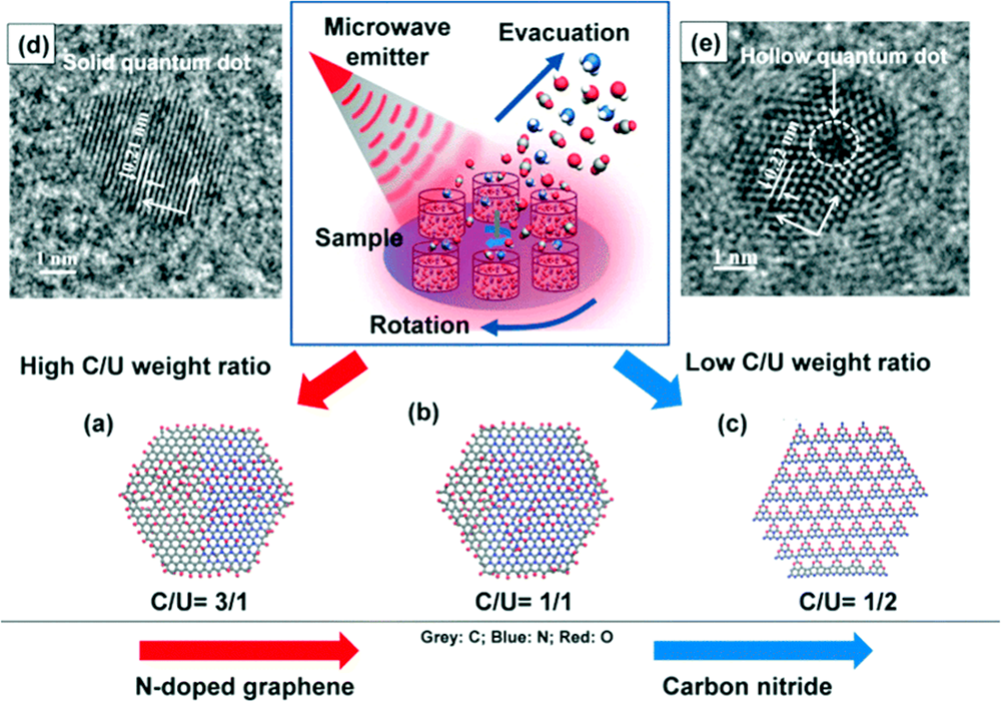
Simulated MD models of CNDs obtained by Gu et al.^320^ via the SPMA technique at different weight ratios of citric acid and urea: (a) 3/1, (b) 1/1, (c) 1/2. Experimental HRTEM micrographs of (d) 3/1 and (f) 1/2 products are also reported. The scale bar is 1 nm long. Reproduced with permission from ref (320). Copyright 2019 Royal Society of Chemistry.

The atomistic details of the N-doped graphene and g-C_3_N_4_ doped quantum dots were studied by means of classical MD simulations using the ReaxFF, on various model systems some of which are reported in Figure 34, in order to verify different possible configurations generated during the SPMA process. Two different atomic structures were considered: (i) heavily N-doped graphene with O adsorption and (ii) heavily O-doped g-C_3_N_4_. The MD simulations indicate that by increasing the urea concentration, it is possible to increase the tendency of in-plane N substitution over that of other amino functionalizations. Finally, MD simulations indicate that N-doped graphene structures preferentially form with a lower N/C ratio, in agreement with the XPS experimental observations.

#### 3.2.4 Sulfur, Boron, Phosphorus and Other Elements as Dopants

Besides the largely exploited N doping, other elements are considered as possible dopants of CNDs to tune their optical, chemical, and physical properties, and the study of their effect is presently very active.

**Figure 35:**
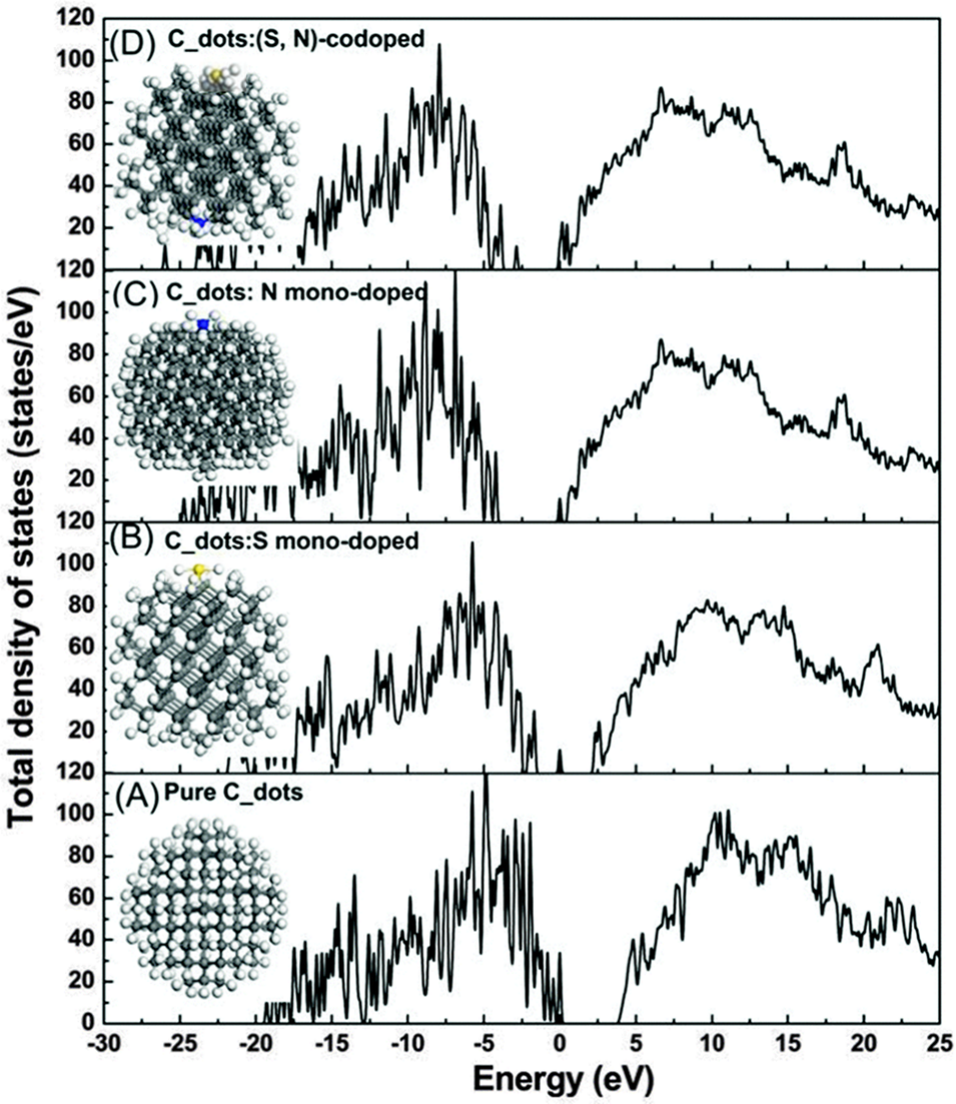
Pure and doped CND models and computed density of states obtained by the HF/OLCAO/LDA method. Adapted with permission from ref (57). Copyright 2015 Royal Society of Chemistry.

Co-doping with sulfur and nitrogen atoms positioned at the edge surface of CND can be exploited in luminescence-based sensing application. Xu et al.^57^ used one-pot hydrothermal synthesis of sulfamide and sodium citrate, a very low amount of sulfamide (ratio 0.1) being required to achieve semicrystal CNDs with very high photoluminescence QY (0.55). S,N codoped CNDs were spherical with an average size of about 7 nm. Beside the semicrystal character, these dots possess an efficient excitation independent emission at 440 nm with an excitation peak at about 350 nm. The emission features are related to surface states whose efficiency is increased by the S and N edge functionalization of CNDs. Within this scenario, the authors performed DFT-calculations of spherical S,N codoped CDs with substitutional single S,N atoms (see Figure 35b–d). The computational results were applied to evaluate the electronic density of states (DoS) and the X-ray absorption near-edge structure (XANES) spectra. The author reported that the pose of the impurity atom incorporation into the CND lattice and single or double doping affects the properties of doped dots. XANES analysis showed that S and (S,N)-doping generates further options of photoinduced electron transitions, contributing to tune the electronic structure of the carbon dot. The latter, compared to the electronic density of states of pure CNDs (see Figure 35A), shows that S,N-related peaks move toward the LUMO in single doped systems, while they move to the HOMO in the codoping case. The presence of S, N and S–N dangling bonds allow the formation of impurity related levels within the energy gap that promote the charge transfer/charge recombination of charge carriers at the surface.

Jana and co-workers^66^ performed an investigation specifically devoted to assess the role of boron doping. A combined experimental and computational investigation was performed making use of a large multitechnique experimental characterization of B-doped CNDs obtained from different B-containing precursors, such as boric acid or sodium borate, and DFT and TDDFT calculations. The computational model system was a PAH with 19 fused rings doped with substitutional boron atoms or boron oxide molecules introduced at the center of the model graphene layer and having the terminal bonds saturated with H atoms and OH groups.

Although the computed spectra did not quantitatively match the experimental findings, the overall observed trend is qualitatively retrieved, showing that B doping causes a red shift of absorption spectrum, the shift increasing with boron concentration. The presence of B atoms produces a huge charge polarization at the carbon surface, thus promoting the formation of surface defects alleged to be responsible for the measured red-shifted optical features. The different effects on the electron distribution and optical properties of doping of GQD with nitrogen, boron, sulfur, and phosphorus is studied with TDDFT methods by Feng et al.^35^

**Figure 36:**
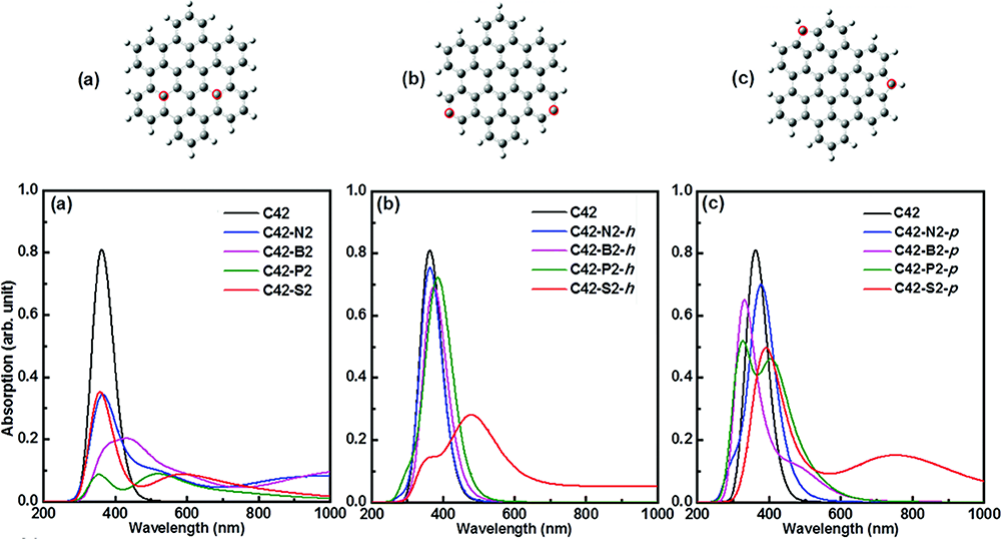
Molecular models (top) analyzed by Feng et al.^35^ to study the doping with nitrogen, boron, phosphorus and sulfur; the positions of insertion of the heteroatoms at the center (a), at the edge (b), in 5 and 6 member rings at once (c) are evidenced by red circles. The calculated absorption spectrum (bottom) is shown under the corresponding structure, with insets indicating the type of dopant inserted, C42 referring to undoped structure (black lines) used as the reference. B3LYP/6-31G(d) is used to optimize structures and to calculate the properties. Doping sites are highlighted by red circles. Adapted with permission from ref (35). Copyright 2018 Royal Society of Chemistry.

Using a PAH with 42 carbon atoms as the starting structure, both inner and edge doping was considered, and in the latter case, both five- and six-membered rings (Figure 36). The calculated absorption spectra show that, compared to the pristine PAH, inner doping leads in all cases to an evident redshift, except for nitrogen and sulfur doped models in which a slight blueshift is detected. Doping in the edge position of six-membered rings induces significant change only in the case of sulfur (strong red-shift). The heteroatoms in the edge position in five-membered rings, do not exhibit an unique behavior, since the redshift is observed in the case of nitrogen and sulfur (especially for the latter), while the blueshift appears for boron and phosphorus.

Geometrical changes induced by the presence of the heteroatoms are particularly relevant only in the case of inner doping. In this case, doping with the larger P and S atoms caused structural deformations that can be clearly assumed as responsible for the gap modifications observed, while in the remaining cases no clear relation can be found. On the other hand, N and B inner doping leads to a suppression of H(π) → L(π*) transitions, while P and S atoms in contrast exhibit spatially delocalized orbitals.

For the edge doping, no structural deformations can be invoked to explain the differences observed. The effect is clarified by the vertical electronic transitions and charge density difference maps: the smaller boron atoms do not cause differences in spectral lines as pronounced as in the case of larger phosphorus atoms, which strongly localize the involved orbitals. However, both boron and phosphorus impact on the charge density differences resulting from transitions, by creating two distinct charged regions. These regions can promote a charge transfer mechanism, enlarging the HL gap.^68^

Concerning fluorine doping,^113^ two model systems were considered, pyrene and circum-pyrene (4 and 14 benzene rings, respectively) with covalent edge-fluorination (F atoms in substitution to H ones at the edges) and fluorine anion doping (F^–^ ion in the same molecular plane as pyrene or above it in different positions). The study was limited to a comparison of high-end DFT/MRCI calculations with the SOS-ADC(2) and CAMB3LYP levels of theory. As already observed, the latter gives a wrong ordering of the lowest excited states. The former, however, can stabilize the structures because of an overestimation of charge transfer among the two species. Concerning covalent doping, the calculated red shift was relatively small because of the electron-withdrawing character of F atoms. The shift increases with the increasing F-content. As for the fluorine anion doping, the effect largely depends on its position with respect to the pyrene molecule, with large variations observed in the relative ground state stability, in the charge transfer character, and in the shifts of the optical features. In-plane doping causes small red shifts and mostly unstable excited structures and out-of-plane doping causes structure distortion and red shift of optical features because of fluorine interacting with the π electrons resulting in an overall increase of the excited states oscillator strength.

The effect on the doping of silicon on the structure of PAH was investigated by Mocci and co-workers^496^ in a computational study on coronene and ovalene molecules and their Si-atoms substituted counterparts. As observed for other “large atoms” as P and S,^35^ insertions are able to significantly deform the PAH structure in the out-of-plane directions, while some peripheral substitutions do not present this effect.

**Figure 37:**
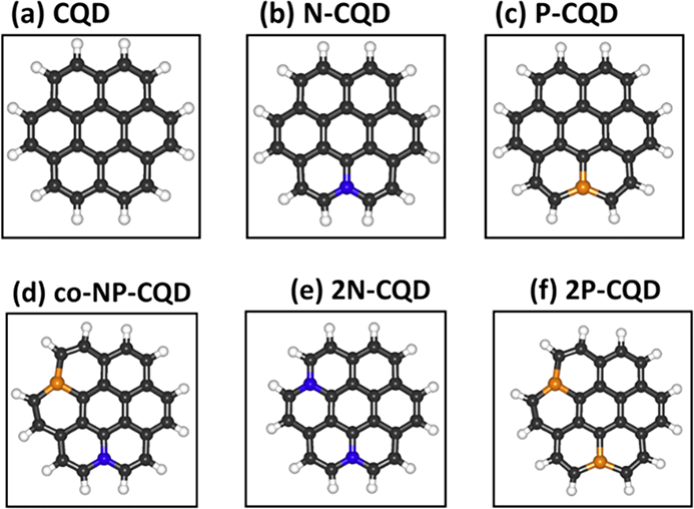
DFT/GGA-PBE/44.1 Ry (a) pure, (b) one unit N-doped, (c) one unit P-doped, (d) N and P codoped, (e) two units N-doped, (f) two units P-doped GQDs considered by Yashwanth et al.^62^ Reproduced with permission from ref (62). Copyright 2020 Elsevier Ltd.

Yashwanth et al.^62^ reported an experimental and computational study focused on the photocatalytic properties of nitrogen and phosphorus codoped carbon quantum dots (“NP-CQDs”, according to the authors’ definition, see Figure 37).

In the following, we will indicate them as CNDs for consistency with the review. N-doped, P-doped, NP-doped, and pure CNDs were prepared using a microwave assisted method were characterized by UV–vis absorption spectroscopy, XRD, HRTEM, XPS, and UPS. All types of doped CNDs showed photocatalytic activity for the degradation of methylene blue under visible light. The absorption main peak of methylene blue decreased with increase in exposure time and completely reduced in the case of NP-CNDs, resulting in the full degradation of the dye. This result is related to the role of oxidizing species, such as O_2_^•–^, which are formed upon transfer of photogenerated electrons to molecular oxygen.^62^ In turn, photogeneration of electrons can be interpreted according to UPS data, which suggest that nitrogen and phosphorus doping promoted a decrease of the samples work function, probably due to N and P generation of extra energy states.^497^ DFT calculations of the work function were performed to theoretically validate the experimental results. Different types of CNDs were modeled as coronene molecules, with edge atoms saturated by hydrogen. From the simulations, the optimized structure, the partial density of states (PDoS), and the electrostatic potential of all models were obtained. The higher value for the work function was predicted for pure CND, while the lower was assigned to NP-CNDs, indicating a synergistic effect in the codoped system and confirming the experimental data.

Looking at the catalytic applications, doping with heteroatoms such as B and N is exploited to confer electronic acceptor or donor character to GQDs and larger reactivity toward the environment, for the adsorption of molecular oxygen. Photocatalytic features are related to carrier relaxation dynamics which are affected by the presence of the heteroatoms. The general computational approach is based on DFT calculations and neglects the thermal nuclear motion and the induced nonadiabatic crossover frequency. To estimate this effect, Cui^498^ used the *ab initio* nonadiabatic molecular dynamics (AINAMD) to compare the carrier relaxation dynamics in pristine GQDs and B or N doped ones. The model system was made of 48 C atoms and 18 H atoms in the pristine case, and five B or N atoms replaced C atoms in the doped systems. DFT was used to compute electronic structure and carrier populations at each step of the nuclear trajectories simulated by means of MD. Thermal fluctuations dominate the nuclear motion, and the Surface Hopping method was applied to describe excited states dynamics (see section 2.2.2.6). B and N doping changed the Fermi level position as compared to the pristine system by introducing additional unoccupied (B) and occupied (N) electronic levels within the gap. Charge carrier relaxation, nuclear dephasing, and recombination processes were considered to evaluate the photocatalytic properties of doped graphene. The analysis showed that in the case of B doping, the carrier relaxation is asymmetric for electrons and holes and it is slowed down, thus promoting BGQDs as a suitable material to catalyze water splitting. On the contrary, NGQDs display slower electron cooling than hole cooling, favoring oxidation instead of reduction activity, as compared to pristine GQDs.

Su et al.^499^ studied Zn-doped CNDs produced with a one-step solvothermal method and capable of emitting in a large spectral range, useful for potential white LED applications. TEM and AFM characterization techniques both indicate the presence of a regular graphitic core structure with an estimated size of ∼4–5 nm. The PL spectra of the CND is dependent on the ratio of the used precursor. A possible way the emission is affected by zinc is due to its capacity to remove C=O and OH groups from the surface.

To elucidate the role of zinc in affecting the emission, DFT calculations were performed on PAH model structures characterized by different combinations of C=O, C–OH, and −NH_2_ groups, and the variation in HOMO/LUMO gaps were analyzed.

Even if the specific role of each group was clearly detected, with pyrrolic nitrogen and C=O leading to a blueshift while the other groups leading to redshift, the synergistic action of these groups was found difficult to be modeled. However, it was found quite plausible that the lowering of the oxidation state induced by zinc, i.e., the removal of red-shifting groups, is compatible with the observed trend in emission spectra.

Chronopoulos et al.^70^ experimentally studied the multifunctionalization of graphene by synthesizing in a one-pot reaction double functionalized graphene from fluorographene (FG) with organometallic nucleophilic reagents containing alkyl or heteroarene ring moieties. The experimental results were rationalized by DFT calculations of nucleophilic strengths and binding energies of nucleophiles on different types of partially functionalized FG substrates showing that the nucleophilicity and electrophilicity of the latter drives the grafting of the units. By comparing natural charges on terminal carbon atoms, butyl (Bu) anion was shown to be a notably stronger nucleophilic agent than the thienyl (Th) anion. The binding energies of the anions follow the electrophilic strength of the substrate, decreasing in the order pFG > G-Bu > G-Th.

**Figure 38:**
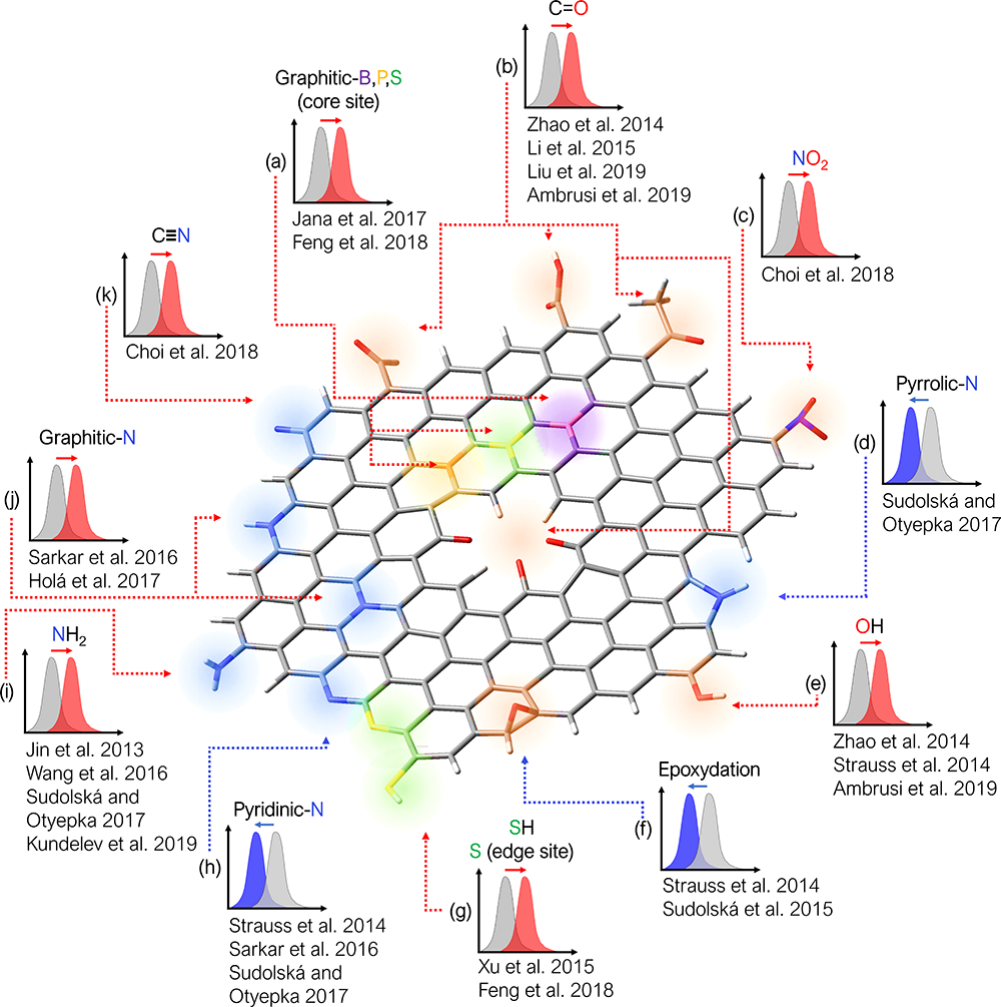
Computed effect on absorption spectrum of different doping/functionalization of CNDs. CNDs is represented as a layer of graphene with doping heteroatoms or functional groups at the edge or within the carbon network: (a) refs (35 and 66), (b) refs (26, 60, 68, and 78), (c) ref (64), (d) ref (72), (e) refs (26, 60, and 67), (f) refs (48 and 67), (g) refs (35 and 57), (h) refs (41, 67, and 72), (i) refs (29, 52, 65, and 72), (j) refs (41 and 42), and (k) ref (64).

**Figure 39:**
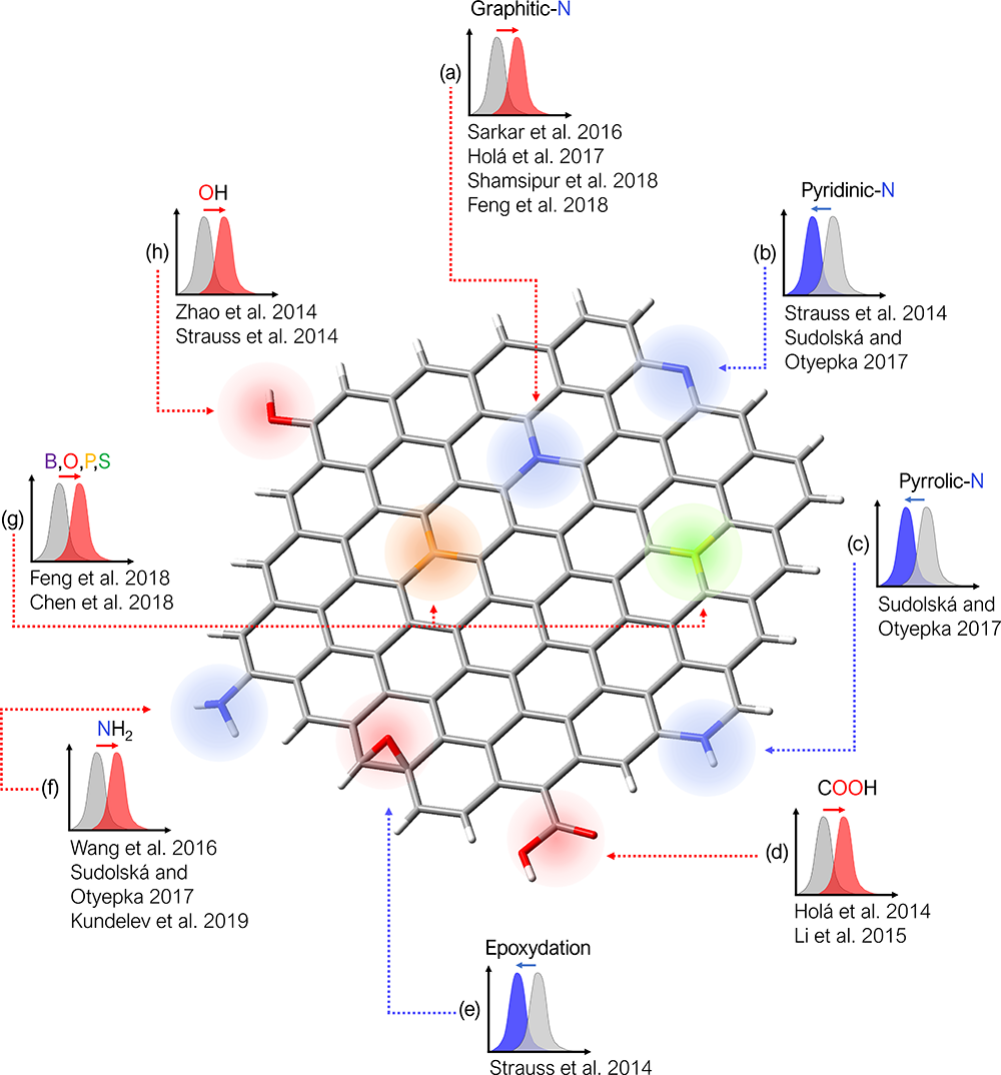
Computed effect on emission spectrum of different doping/functionalization of CNDs. CNDs is represented as a layer of graphene with doping heteroatoms or functional group at the edge or within the carbon network. (a) refs (34, 35, 41, and 42), (b) refs (67 and 72), (c) ref (72), (d) refs (39 and 68), (e) ref (67), (f) refs (29, 52, and 72), (g) refs (35 and 71), and (h) refs (26 and 67).

To sum up the different effects of doping on the optical absorption properties of CNDs (see Figure 38), we note that there is a general agreement on the red shift of the HL gap by graphitic N^41,42^ and by edge amino groups.^29,52,65,72^ On the contrary, pyridinic and pyrrolic are reported to blue shift the absorption features.^41,67,72^ The oxygen related doping is more controversial, the carbonyl^26,60,68,78^ and hydroxyl^26,60,67^ group being associated with a red shift and the epoxy causing a blue shift.^48,67^ The presence of ether groups is reported to increase the Stokes shift between absorption and emission transitions.^71^ The effect on the emission properties are collected in Figure 39. There is a general consensus that graphitic N^34,35,41,42^ and amino groups^29,52,72^ promote a red shift of the emission, while pyridinic^67,72^ and pyrrolic^72^ cause a blue shift. Also epoxidation^67^ induces a blue shift of the emission. On the contrary, carboxyl groups,^39,68^ a hydroxyl group,^26,67^ or substitutional heteroatoms^35,71^ cause a red shift of the emission. Finally, other doping mechanisms, such as nitro or cyano groups, cause a red shift,^64^ and codoping of sulfur and nitrogen are reported to produce impurity levels within the energy gap^57^ or reduce it,^35^ and the B doping may cause both red shifting^66^ or a blue one,^35^ the latter when coupled with P. Beside the shift of the optical properties related to the presence of a specific atom or chemical groups, another important parameter is the geometrical distortion of the C network due to the presence of the dopants, larger when inserted within the network than when positioned at the edge (see, for example, refs (29, 35, 67,71, and 77)). In this respect, the selection of a proper model in terms of size and geometry is mandatory to provide reliable results. The geometry of the model is also relevant to define the proper sequence of excited states and their electronic character, the exploitation of at least two-three layers of graphene like structures being required for these purposes (see the work of Otyepka and co-workers^41^ and Sudolská et al^48^). Indeed, when considering PAH models, both undoped and doped, the interaction among the layers by means of p electrons and the size of the conjugation affect the calculated optical properties. Finally, concerning the methods, when computationally affordable, one should apply the multireference ones to evaluate the excited states, as also reported in the section 2.1.11 and the papers of Lischka (Table 3), since the most common TDDFT-B3LYP combination could fail in predicting the position of bright and dark states.

### 3.3 Fluorescent Molecules in CNDs

As stated before, the main feature of CNDs is their efficient excitation dependent emission, and the majority of reported computational CND research has been devoted to its proper modeling. In the previous sections, we have discussed the efforts in understanding the role of carbon networking (sp^2^ and sp^3^ bonding) and the effect of dopants. Those studies can be framed within the three main models usually adopted to explain the emission properties of CNDs, assuming that the fluorescence is being generated by core states, surface states, or molecular states. Core states account for the size dependence of fluorescence (in general reported in top-down synthesis and C-networking). Formation of surface states is related to the presence of specific atoms or chemical groups at the CND surface and the formation of electronic levels within the energy gap. The molecular states call for the formation of specific fluorescent molecules during the bottom-up synthesis. Lately, a molecular model was largely investigated by computational studies with the aim to ascertain, beside the optical features of selected molecules, the formation of aggregates as a possible explanation of excitation dependent multicenter emission in CNDs. However, the way these emitting molecules and aggregates are organized within the structure of a carbon dot is still an open question.

The synthesis process of CNDs obtained in the bottom-up approach is a complex sequence of polymerization and carbonization steps through which different byproducts can be obtained. These molecular debris can be further involved in the synthesis process as the seed for CNDs enucleation or can be incorporated into the final CNDs product. The presence of intermediates is relevant for the optical properties, since different molecules with emission in the blue-green region of the visible spectrum were identified as the source of the photoluminescence of CNDs and its peculiar excitation dependence, see, for example, the IPCA molecule for the blue range, the HPPT for the green one, and the combination of PAHs for the excitation-dependence feature.^398,450,456^

One of the most exploited reactants to form CNDs is CA, typically combined with N-containing molecules such as urea, ammonia, or various amine compounds.^399^ During the synthesis, CA reacts to form CZA and other molecules, such as the above-mentioned HTTP and IPCA molecules, that received large attention in the context of the fluorescent properties of CNDs, as evidenced by the works reviewed in this section.

As reported by Mura et al.,^47^ “understanding the properties of the intermediate such as CZA is, therefore, a mandatory step for achieving an efficient control of carbon dots synthesis.” Mura et al.^47^ investigated the optical properties of CZA as a function of concentration to show that the absorption and emission features of this molecule and its aggregates largely resemble the ones of CNDs, suggesting that to control the optical properties of CNDs one should take good care of the synthesis procedure that can work as a bias to the final product. The presence of the keto-monomer form and the formation of aggregates which contribute to the red shift of the absorption spectrum, experimentally observed as the concentration of CZA increases, was confirmed by theoretical calculations, carried out at DFT and TDDFT levels (see Table 1). As for the previously cited cases, these results strongly support the attribution of the optical properties detected in CNDs to a molecular model, at least for the CA related CNDs synthesized by the bottom-up approach.

The presence of CZA and its aggregates was also investigated by Nandy et al.^46^ who considered the idea of molecular emission in CNDs from both the experimental and theoretical points of view. First, they showed that room temperature incubation of CZA in dimethylformamide (DMF) can yield nanocarbon particles with excitation dependent emission. Optical and morphological features of these nanoparticles are phenomenologically like the ones of CNDs. Indeed, nanoparticles of about 2.5 nm with graphene-like nanosheets were observed, suggesting that H-bonded molecular clusters of CZA were largely involved in the formation of these carbon nanoparticles. The same relationship could hold also for CNDs prepared by pyrolysis approaches, where the excitation dependence of the emission is related to the precursors applied and is switched off in the case of steric hindrance of aliphatic chain precursors.

To corroborate the aggregation of CZA molecules through H-bonding, quantum chemical calculations were performed within the framework of DFT and TDDFT (see Table 1). The solvent was modeled with a continuum model with integral equation formalism (IEFPCM) in both optimization and vertical excitation energy calculations, the latter being performed on the gas phase optimized geometry of the ground state (S_0_). The agreement between the calculated absorption peak of CZA and the experimental absorption of CNDs grown by hydrothermal synthesis in water is very good, showing that the formation of computed aggregates causes a hypsochromic shift (dimer) or bathochromic shift (tetramer) of the absorption peak that could account for the reported excitation dependence (an overall red shift of 30 nm was calculated between dimer and tetramer spectra). Studying the molecular heterogeneity of CNDs and the possibility to obtain blue, green, and red emissions from the solvothermal synthesis of CA and ammonium thiocyanate in water and DMF, Nandy et al. isolated the excitation dependence feature and ascribed it solely to a blue-emissive fluorophore, such as CZA, formed in the synthesis. In addition, the presence of two other emitting fluorophores with an emission peak in the green and red spectral range, respectively, was experimentally evidenced. The authors exploited DFT/TDDFT simulations to predict the chemical structure of the species obtained during the synthesis. Considering the expected presence of pyridinic and pyrrolic nitrogen atoms and thiophenic sulfur, it was shown that organic N,S-containing fluorescent molecules can cover the whole emission spectrum of the synthesized CNDs. Beside the blue emitting CZA, two other centers were proposed to account for the green (pyridotriazinthione) and red (pyridopentazinthione) emissions.

To evaluate the ability of the molecular model to explain the optical and magnetic properties of CA related CNDs, Mocci et al.^50^ propose a combined experimental and computational approach to study the protonation state of CZA ions in water. While fluorescent CZA and its derivatives were reported as possible sources of the optical features of CA related CNDs, the contribution of CZA on CNDs magnetic properties is debated. In this paper, the formation of different ions of CZA at different pH conditions and the interaction of CZA molecule with specific solvent is investigated by means of DFT and TDDFT calculations (see Table 1). Besides the assessment of the most favored ionic species, the authors calculated the UV–vis absorption spectra, the Raman and IR features, and the NMR chemical shift. The computed results show that CZA molecule is indeed an efficient molecular model for CNDs and can explain the optical and magnetic properties of CNDs synthesized in water from CA at low temperature and with short reaction time.

Wang et al.^25^ proposed CZA and derivatives as a fluorophore in the CND they obtained from CA and urea. By increasing the temperature and time of the synthesis, the QY was found to decrease. A lower temperature and synthesis time (180 °C and 3 h) allowed one to achieve the largest efficiency coupled with a larger photobleaching upon UV exposure. On the contrary, with higher temperature and longer time of synthesis, the produced CNDs have a lower photobleaching response and a lower QY. To model the species responsible of the observed optical features, the authors simulated the amide derived from CZA, and calculated UV–vis optical features by means of the DFT and TDDFT methods. The calculated data fully support the presence of this molecule at the CNDs surface, with a HOMO–LUMO energy gap at about 340 nm as experimentally observed, with the pyridone ring also being responsible for the photobleaching effects on CNDs.

**Figure 40:**
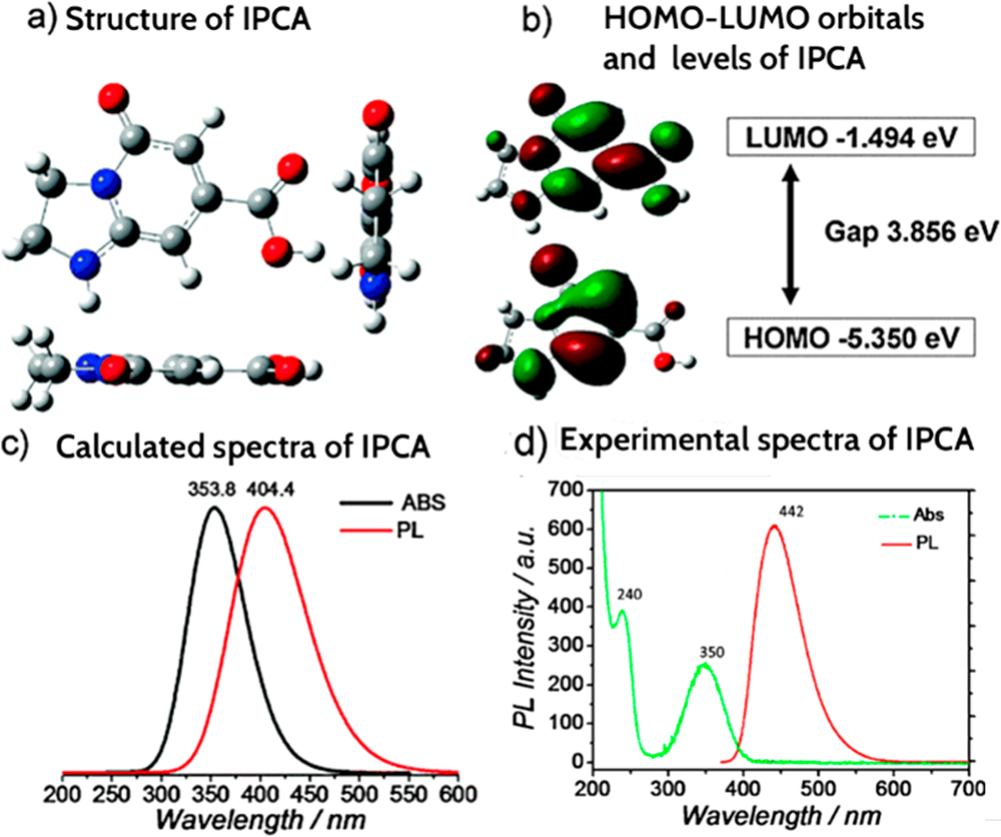
(a) Structure, (b) HOMO and LUMO orbitals and energies, and (c) calculated Abs and PL spectra for the IPCA molecule. Adapted with permission from Song et al.^450^ Copyright 2015 Royal Society of Chemistry^53^^53^

The formation of aggregates was also the target of the work of Kundelev and co-workers^53^ to predict how red emitting CNDs can be produced, assuming that their optical properties can be simulated by means of molecule-like subunits of PAHs attached to the CND surface, as exemplified in Figure 40. Experimental results show that producing red emitting CNDs with high efficiency is a difficult task. As discussed in previous sections, the effect of nitrogen doping through pyridinic, pyrrolic, or amino centers was previously investigated, while coupling of surface emission centers was not considered a possible cause of emission red-shifting and efficiency decrease.

Kundelev et al.^53^ considered perylene based subunits to simulate the optical properties of noninteracting centers (zero coupling, isolated monomers), weakly interacting centers (noncovalently bonded dimers), and tightly interacting centers (covalently bonded dimers, through 2 to 4 carbon linkers). Those surface centers are attached to the CND core by means of long and flexible aliphatic linkers that do not impact the optical properties of the system. No contribution from the core is considered. Absorption and emission features of surface centers were calculated within the TDDFT framework (see Table 1 and Table 2). Excited-state geometry optimization was carried out to compute the energies and oscillator strengths of the electronic transitions in the PL spectra, starting from the geometry of the ground state as a first approximation. No solvent interaction was considered. The computed absorption and oscillator strength show that aggregation causes red-shift of the absorption features and a large decrease (of about 2 orders of magnitude) of the absorption rate. In a similar way the emission brightness decreases and a large Stokes shift with respect to the absorption peak is retrieved, leading to red-emitting centers. The important conclusion the authors draw is that by controlling the interaction among surface emission centers one can control the PL wavelength, the QY, and the oscillator strength of the radiative transitions to enhance the red luminescence of CNDs, as required for photonic and biomedicine applications.

One of the most accredited molecules that are expected to form during the bottom-up synthesis from CA precursor and amines is IPCA. Its formation was inferred by Song et al.^450^ in a combined experimental and theoretical work where “the chemical structure and PL mechanism was uncovered by special synthesis and separation routes.”

**Figure 41:**
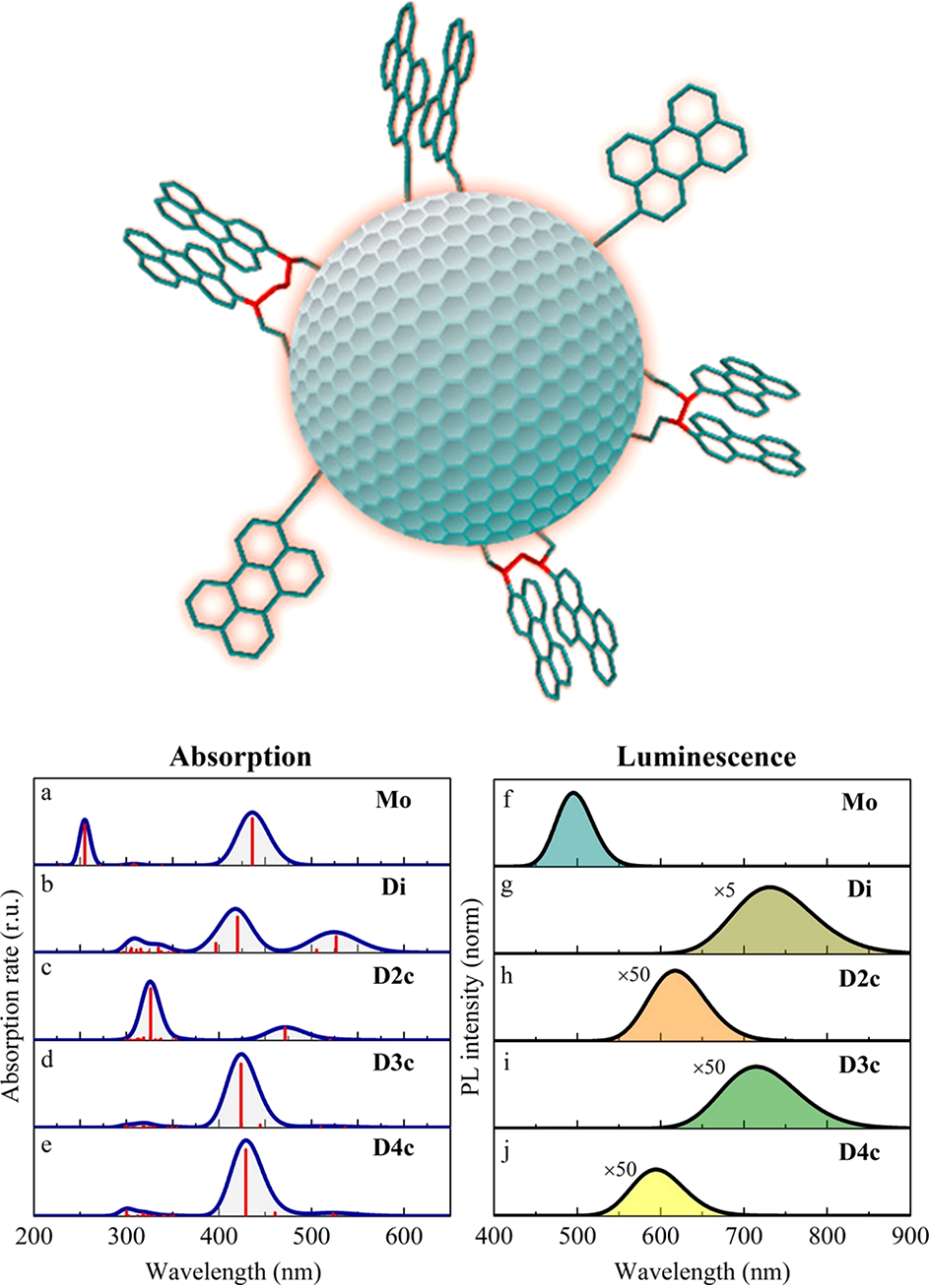
Model system (top) and optical spectra (bottom) of molecular-like subunits of PAH considered responsible for the optical properties of CND in the work of Kundelev et al.^53^ in the case in which a single perylene molecule (Mo), a noncovalent perylene dimer (Di), and a covalent perylene dimer via an n-aliphatic chain (DnC) are attached to the CD surface. All properties are calculated at the B3LYP-D/DZP level of theory. Adapted with permission from ref (53). Copyright 2019 American Chemical Society.^450^

Song et al.^450^ produced CNDs by solvothermal synthesis with CA and EDA, the latter being not only a precursor for IPCA molecule synthesis but also a linker and passivation agent in graphene-like carbon core structures. By changing the molar ratio, pH, and temperature, the optimal synthesis conditions with respect to the optical properties of the obtained CNDs were found to be a neutral pH with a 1:1 molar ratio and a temperature in the range 140–150 °C. After purification, a blue emitting fluorophore with 180 g mol^–1^ molecular mass was isolated, successively identified as the IPCA molecule by mass spectra, NMR, 2D-NMR, and elemental analysis. DFT and TDDFT calculations (see Figure 41 and Table 1 and Table 2) match the experimentally observed optical features and the strong blue PL in aqueous solution at 240 and 350 nm. The presence of the latter peak was confirmed in carbon dots produced at 140 °C and is conclusively related by the authors to the formation of IPCA during the CDs synthesis. In addition, by performing the synthesis at 140 °C with only CA as precursor, blue photoluminescence could also be found with typical signatures linked with the presence of nanosized graphene-like structures, as shorter PL lifetime and multiple exponential decay. The presence of carbon core states was finally confirmed by the possibility to tune the PL emission from green to blue. The temperature was considered as the critical parameter in determining the characteristics of the synthesized CND. Indeed, in the presence of EDA, CA can always form IPCA molecules, which, at higher temperatures, give rise to polymeric structure. A further increase of temperature leads to cross-link carbonization of polymer chains and formation of graphene-like structures that, in turn, can be passivated by the remaining IPCA molecules present in solution.

Shampsipur et al.^34^ proposed an experimental and multivariate decomposition procedure to identify the emission centers responsible for observed UV spectra in CNDs and how each emission center contributes to the overall spectrum. DFT calculations allowed verifying the possible aromatic compounds contributing to the optical properties. After synthesis through pyrolysis of CA and EDA performed at different temperatures (150, 200, 250, and 300 °C), authors observed that temperature has a detrimental effect on the QY, which lowers from 75 to 12% when *T* is increased from 150 to 300 °C. Moreover, the PL appears to depend on the imposed excitation in low-temperature synthesized CDs.

It is worth noting that these differences in emission spectra are in strict relation with the morphology of CDs: in fact, while at 150 °C formation of large polymeric structures can be traced, long-range order seems to be inhibited above 250 °C, when spherical nanoparticles of 4–5 nm size are found in dispersion. Dehydration and aromatization, taking place in the synthesis process, are thought to be the key-factors accounting for cross-linked polymerization and emission features, in good agreement with the literature^8^ and XPS data, suggesting formation of disordered graphitic structures for *T* ≥ 200 °C.

The presence of multiple emitting centers is inferred from a photobleaching experiment. Indeed, also the variation of the absorption spectrum as a function of temperature suggests the presence of different surface groups (like C–OH, C=O, and C=N, as suggested in refs (500 and 501)), molecules like IPCA^450,502^, and finally graphitic aromatic domains.

The origin of fluorescence is very different in each CND specimen: those obtained at 150 °C, in fact, are attributed to the functional groups of unreacted precursors trapped in CD, while those obtained at 200 °C are the luminescent IPCA molecule embedded in a polymer-like structure as well as to electron–hole recombination in carbon core states and polycyclic aromatic hydrocarbons. The latter dominates in the CND obtained at 300 °C. These results are supported by the DFT and TDDFT calculations, with the simulated spectra suggesting the presence of IPCA, aza-polycyclic aromatic molecules, pyracylene, azabenzoanthracene, azoperylene, and azapyrene. Moreover, in the CNDs obtained at 300 °C, strong evidence for the presence of C_60_ and C_70_ is found as well as the presence of its possible precursor, corannulene C_20_H_10_ and the observed redshift explained as an N-doping effect.^41,42,473^

Siddique et al.^55^ very recently performed an extensive computational investigation on the formation of IPCA small oligomers to find a possible relationship between the aggregation trend of this molecule and the optical properties of CNDs. The conformational space of stacked structures of dimers was systematically sampled by DFT calculation (see Table 1), finding several minima with similar energy, indicating a rotational flexibility which was confirmed by atomistic MD simulations (see Table 4). The latter reveal the spontaneous tendency of IPCA to form stable stacked dimers and trimers, where the monomer units participate in the formation of the aggregate through H-bonds, involving the carboxyl, carbonyl, and N–H groups of the molecule. These structures undergo a rotational movement of the units around the axis perpendicular to the stacked planes, and the rotational distribution profile agrees with the most stable structures observed by the DFT calculation. These most stable structures were used to calculate excitation energies with the TDDFT method, and the expected exciton splitting was observed, with the spectral shifting being dependent on the specific dimer configuration. In addition, the π–π* character of the excitations was recognized for both the near and far UV transitions, since each transition is a local excitation realized within the π-orbital space of one monomer only. On the contrary, no n−π* transitions with nonzero oscillator strength were detected, casting some doubts on the previous attribution of the UV absorption spectrum in CNDs, at least for those where IPCA can be invoked. The red-shifted emission spectrum of the dimers was also evaluated, both in a gas and water environment, laying the groundwork for the evaluation of the photokinetics of the dimers, a relevant issue in estimating the QY of CNDs and to engineering larger optical efficiency.

**Figure 42:**
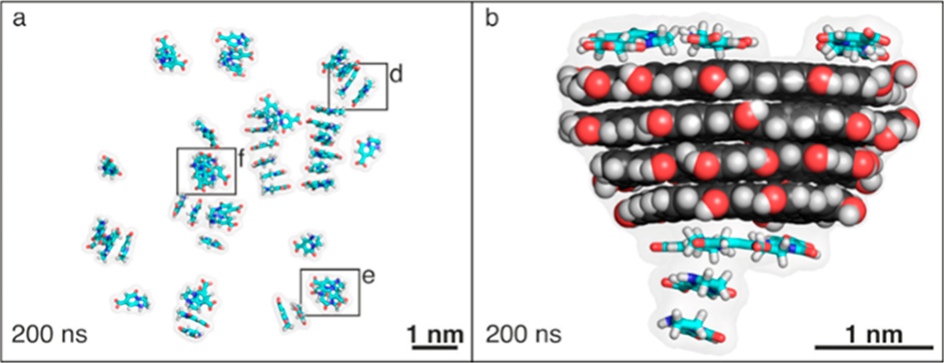
Snapshots from the MD simulation of Langer et al.^95^ showing self-assembly of IPCA molecules (left), stacking of IPCA molecules on the CND surface using GAFF (IPCA) and AMBER (CDs) force fields (right). Adapted with permission from ref (95). Copyright 2020 American Chemical Society.

Langer et al.^95^ made a step forward in the study of fluorophore aggregation starting from their very recent results on IPCA aggregates^55^ and exploring how neutral and anionic forms of IPCA can play as a seed for CNDs nucleation because of their natural tendency to self-assembly in π–π stacked layers. The authors demonstrated, through MD simulations (see Table 4), that IPCA can interact with CNDs, forming additive layers on graphitic CNDs or being inserted within their planar carbon building blocks. The simulations were carried out within the GAFF^274^ and AMBER^273^ FF framework (for the IPCA and CDs, respectively), with the CND structures being provided by a carbon dot builder.^271^ The studied systems included also the formation of CNDs from edge functionalized PAHs. Classical atomistic MD simulations were performed in an explicit water solvent environment both at standard conditions (room temperature, 1 bar pressure) and at a temperature of 473 K and pressure of 15.5 bar to emulate experimental synthetic conditions. The analysis allowed the authors to explore a large panorama of possibilities and to sketch how IPCA molecule and its aggregates can be distributed within the CND structure. The key aspect is the tendency of IPCA to dynamically self-assemble in water into two or three stacked layers (Figure 42) both at room temperature and, to less extent, at high temperature. Those stacked structures, whose lifetime ranges between picosecond and few nanoseconds depending on their size, can be the seeds to the formation of graphitic CDs, the interlayer distance being of about 0.34 nm, in agreement with the interlayer distance of graphene layers in graphite and graphitic layers in CNDs. When fragments of CDs are present in the water solution, IPCA interacts with the carbon layers preferentially forming stacked layers on the solvent exposed surface of the CND fragment. Besides π-stacking interactions, dynamic H-bonding interactions are also observed, with a stability of few nanoseconds which is reduced to tenths of picoseconds when the interaction with a fully spherical CND is considered.

Finally, starting from the aggregation of PAHs in graphitic structures, the authors showed that IPCA can be both added by π-stacking interactions on top of PAH sheets or incorporated into the CND structure, preferably in surface poses to complete the PAH layers. Those results were also confirmed in the high temperature and pressure simulations, providing a fully fledged interpretation of the molecule model of IPCA related emitting centers in CNDs.

Successively, Langer et al.^73^ computed the excitation and emission properties of IPCA at the surface or embedded within the matrix of a CND made of PAH layers. Starting from the structures previously generated with classical MD simulations, the optical properties of the optimized structures were computed with a hybrid QM/MM approach. The ONIOM method^503−505^ was employed to calculate the excitation and emission features of IPCA (QM region), while the CND fragment was surrounded by explicit water molecules (MM region). DFT and TDDFT levels of theory, depending on the monomer or dimer IPCA systems, were considered for the QM region (ωB97X-D/6-31++G(d,p) for the ground state optimization and CAM-B3LYP/def2-TZVP for the excited state properties). The electrostatic embedding (EE) approach was compared with the polarizable embedding (PE) approach in the case of hydrated isolated IPCA molecules, also exploited as benchmark for the level of theory and the solvation model. The comparison indicated that TD-CAM-B3LYP and EE for the QM and MM regions, respectively, allows predicting the PL properties of IPCA in complex environments. Since the structure and the optical features of IPCA can be affected by the interaction with the CND layered structure, depending on the relative position of the IPCA molecule itself, several representative arrangements were considered. For all of the structures, the first bright transitions had a π–π* character for isolated IPCA; and the greatest variation in excitation energies was computed for fully hydrated molecules, suggesting a relevant role of the solvent-shell on the optical features.

**Figure 43:**
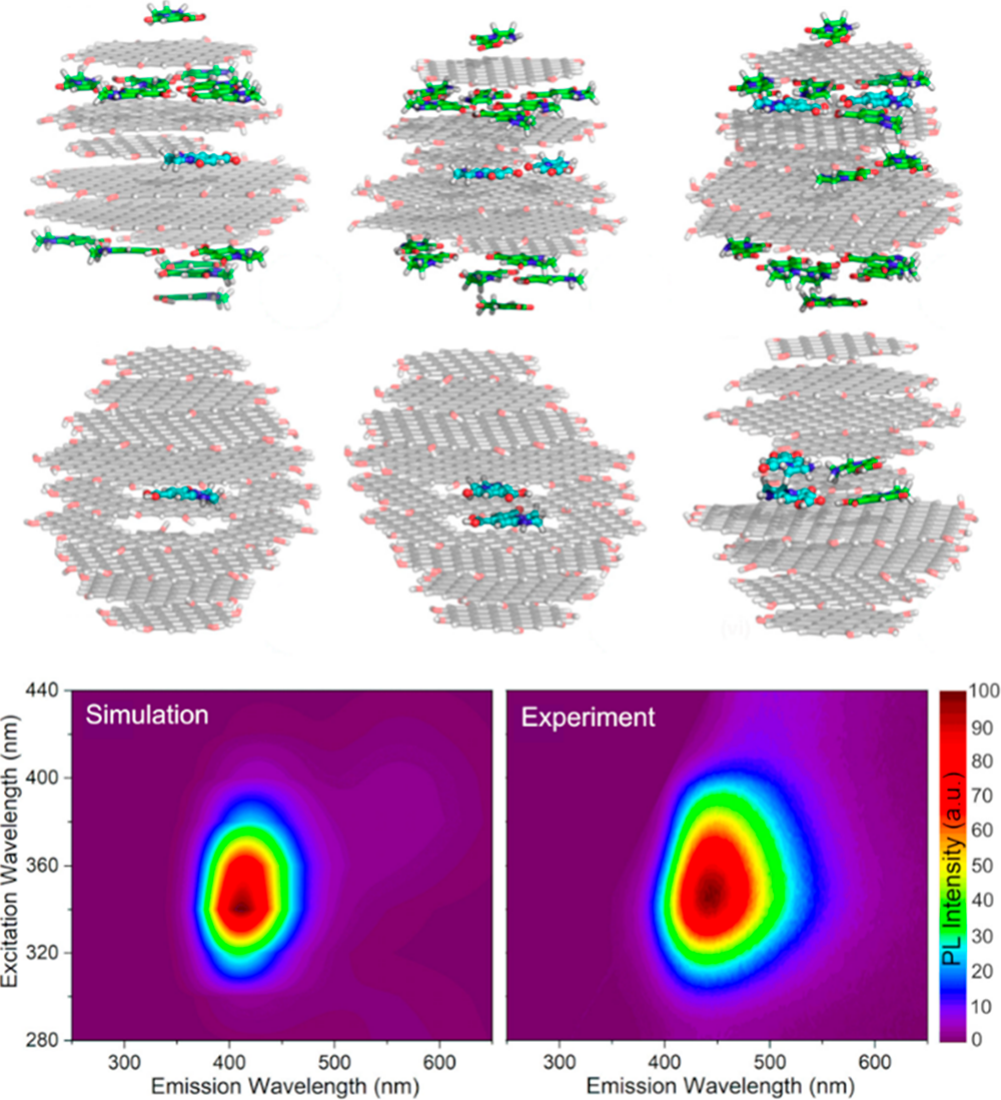
QM/MM models used by Langer et al.^73^ to study the interactions of IPCA molecules with a multilayer GQD in explicit solvent water (not shown for clarity). Excitation–emission map for the IPCA molecule is computed (bottom, left) by averaging the contribution arising from different configurations and compared with the experimental map (bottom, right). Absorption calculated at the CAM/B3LYP/def2TZVP level of theory. Estimated diameter of quasi-spherical structures of about 2–4 nm. Reproduced with permission from Langer et al.^73^ Copyright 2021 American Chemical Society.

The blue or red shift of both excitation and emission transitions, and the efficiency of the latter, depends on the relative position of the molecules with respect to the CND cage and are affected by interactions solvent and by the possible formation of aggregates (dimers, in the present case). The reconstructed excitation–emission map, as reported in Figure 43, agrees quite well with the CNDs prepared by CA and EDA precursors, helping to explain the excitation-independent emission sometimes reported for those CNDs and further supporting the molecular model for the efficient emission properties of CNDs.

A further proof of the molecular model was recently established by our research group^51^ by modeling the aggregation phenomenon of CZA in water. The key aspect was the exploitation of a density functional able to describe noncovalent bonding interactions, such as H-bonds and van der Waals forces, responsible for the aggregation. The formation of dimers, trimers, and tetramers was computed within the DFT framework (see Table 1) with the ωB97XD functional to mimic the interaction among the monomers. The computed structures were tested for optical absorption and vibrational features to model the measured spectroscopic properties in CA-based CNDs. The experimentally reported blue shift of the n−π* transition around 340 nm as a function of CZA concentration was successfully explained by the formation of different aggregates that causes blue and red-shifted excitation transitions with respect to the monomeric one. The computed aggregated structures were further confirmed based on the vibrational Raman features pointing out the aggregation fingerprint around 2900 cm^–1^.

### 3.4 Polymer Carbon Dots

The pyrolysis of carbon sources in the bottom-up approach to the production of CNDs, in particular when CA and amine groups are involved, is expected to produce polymer clusters, the polymerization degree largely depending on specific experimental conditions (such as precursors, solvent, temperature, and combustion procedure). Beside polymerization process, two other mechanisms, condensation and direct carbonization, compete with the formation of the complex structure and morphology of these spectacular emitting nanoparticles (see Song et al.^450^).

CPDs have diverse applications, notably as biosensors, due to their high-water solubility and favorable biocompatibility. However, the precise chemical structures of CPDs and the origin of their unique luminescence properties have not been established, hampering the development of CDs with tunable optical properties.

CPDs prepared from carboxylic acid and amine precursors exhibit unique bright blue fluorescent emission. Vallan et al.^69^ synthesized model polymer CDs, including a novel low-temperature synthesis, performed a chemical structural characterization and studied their optical properties by a combination of spectroscopic techniques and DFT analysis of the ground and excited states of selected CD models. The synthesized CDs were found to consist of a compact network of short polyamide chains, with a size of 1–1.6 nm and were found to have an excitation independent PL spectrum and similar lifetimes but conspicuous differences in the QY, ranging from 64% down to 7%. Observed QY are explained in terms of structures rigidity caused by OH groups, whose amount is dependent on the reactant used and the presence of the coupling agent, as confirmed by varying the pH of the solution containing the carbon dots. A dramatic drop in the emission intensities is found when pH is lower than 5, indicating that protonation and deprotonation can induce a change in the conformation of the polymers present in the CDs.

**Figure 44:**
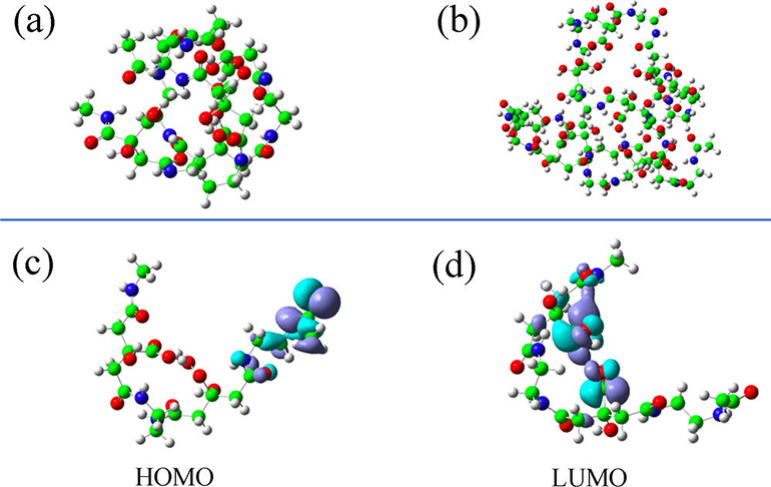
DFT-optimized models of (a) two polyamide dimers and (b) a decamer chain, showing entangled structures due to both intra- and intermolecular H-bonding. Optimization is performed at the ωB97XD/6-31G(d) level of theory while the B3LYP functional is used for HOMO/LUMO calculations. Location of (c) HOMO and (d) LUMO, involved in blue fluorescent emission phenomenon. Reproduced with permission from Vallan et al.^69^ Copyright 2018 American Chemical Society.

A DFT analysis on the structures in Figure 44a,b clearly showed the spatial separation of the HOMO and LUMO located at amide and carboxyl functional groups, as shown in Figure 43c,d. Photoinduced charge transfer (CT) between these sites, enhanced by a rigid supramolecular network structure due to intermolecular H-bonding, was shown to produce the blue fluorescent emission of this class of polymer CD. The metal ion sensing performance of these pCNDs was consequently explained as due to chelation of metal ions by functional groups involved in the CT process.

**Figure 45:**
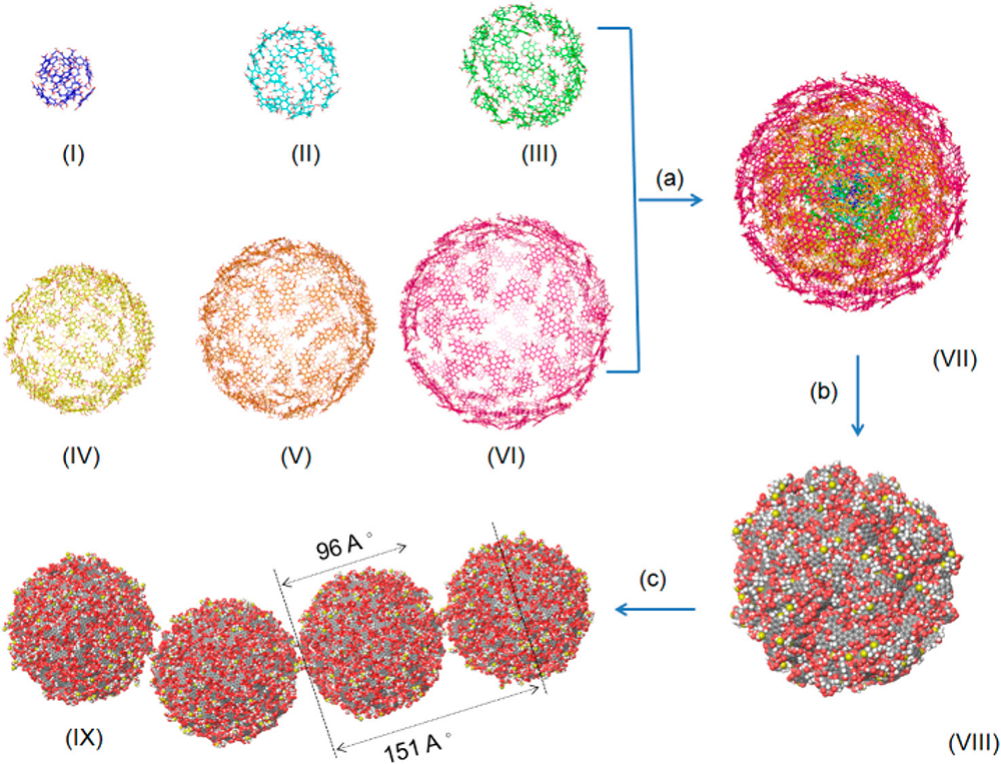
Schematic representation of the pCND model construction as done by Sau et al.^38^ through several steps. (a) Layer by layer assembly of I, II, III, IV, V, and VI. (b) MD simulation of DTT decorated single CND. (c) MD simulation of DTT functionalized pCNDs. Reproduced with permission from ref (38). Copyright 2018 American Chemical Society.

Interestingly, the CNDs can undergo a polymerization process, leading to a new class of fluorescent polymers formed by connected CNDs, called pCNDs. Sau and co-workers^38^ prepared this class of compounds by a polymerization process described in section 2.3.2, leading to a new class of carbon related systems. Indeed, from the experimental point of view, those pCNDs (see Figure 45) show new interesting optical features, such as a double-humped excitation dependent emission due to formation of aggregated fluorophores and energy-transfer states because of the polymerization. The DTT linker is believed to promote the electron density overlap between two blocks of the chain thus causing the formation of these two emitting states. Possible structures resulting from the polymerization process were investigated by MD simulations, confirming that the pCNDs are very stable in water. MD also revealed that π-stacking and intermolecular H-bonding interactions lead to a compaction of the structure.

Beside MD simulations, the authors also investigated, through the TDDFT computational approach, the electron transfer mechanism between CNDs and a model electron acceptor, menadione (MQ), to elucidate the experimentally observed phenomenon. Possible pathways for the orientation of the acceptor model with respect to the polymer surface and consequently for the electron transfer were obtained, the transfer being accomplished between the LUMO levels of the donor and acceptor systems. Interestingly, CNDs were shown to possibly act as both electron donor and electron acceptor depending on the interacting MQ moiety.

### 3.5 Remarks on the Optical Properties of CNDs

To conclude these sections of the review, we want to sum up the main results concerning the effects on the optical features of CNDs due to the size and shape of the nanostructures and the presence of specific functional groups or doping atoms.

**Figure 46:**
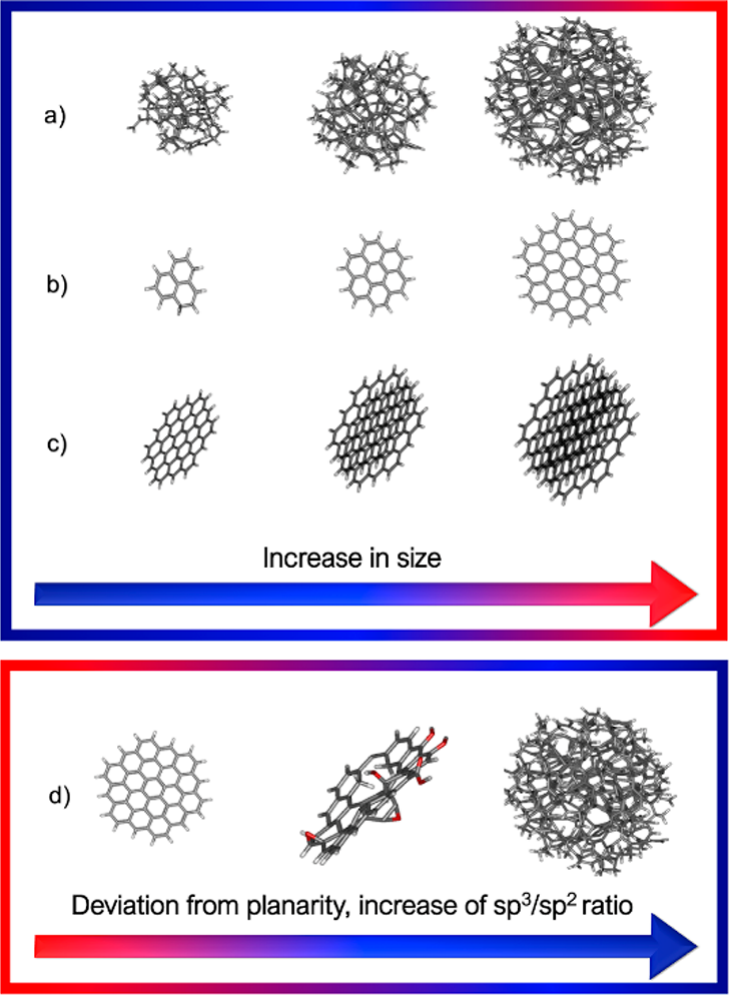
Computed effect on absorption spectrum due to increasing size of (a) amorphous CNDs, (b) GQDs, (c) stacked nanolayers (top); (d) deviation from planarity or increase of sp^3^/sp^2^ ratio (bottom). The arrows indicate the shift toward longer (top) or shorter wavelengths (bottom).

One of the most debated phenomena in CNDs field, both from the theoretical and experimental point of view, is the quantum confinement effect, i.e., the effect of the nanostructure size on the electronic properties. In most cases, the quantum conjugation effect plays a key role by inducing a red shift of the optical features as the conjugation length increases. It should be noted that the relevant size is the extension of the sp^2^ graphene domain rather than the overall size of the nanoparticle. As depicted in Figure 46, the quantum conjugation effect is most relevant for systems with a certain degree of order, like GQDs or CNDs, and it is largely reported in simulations considering graphene layers or PAHs of increasing size.^26,27,78,85,236,30,32,34,36,39,44,46,60^

In the case of disordered systems, such as the amorphous CNDs considered by Margraf,^79^ the same trend is reported but with the red-shift proportional to the diameter of the nanoparticle. The authors assigned a relevant role in determining the observed behavior to the structure of the surface where also the sp^2^ planar island can be formed as a consequence of the relaxing of the geometrical constraints on the surface atoms.

It should be considered, however, that the relationship between electronic properties and geometry of the nanoparticles is not limited to the quantum conjugation effect, because a large variety of structures can be obtained by varying the synthesis conditions. When considering the geometry of the CNDs (Figure 46), an important role is played by the effects induced by changes in planarity,^67^ by the presence of stress,^27^ and by the changes in topology^465^ or the sp^2^/sp^3^ ratio.^68,82^ Indeed, the presence of defects, such as vacancy,^81^ H termination,^374^ specific edge geometries,^32^ or dopants^67,77^ produce a deformation on the planar carbon network whose effect on the electronic levels might counterbalance the red shift of the optical absorption as a function of the increasing system size.

The presence of sp^3^ bonding is, indeed, considered mandatory to open a gap in an otherwise ideal infinite graphene layer^81^ and the increase of density by means of increasing sp^3^ contribution was reported to open the energy gap.^451^

In the work of Zhu et al.,^27^ small nonplanar models of CNDs were affected by a larger strain that releases its energy in the excited states thus producing a decrease of the energy gap (red-shift in wavelength). The geometrical deformation is also related to the sp^2^/sp^3^ ratio,^82^ a crucial parameter in simulating the order–disorder ratio in CNDs. From this point of view, it is important to consider the degrees of freedom offered by the CND surface, where a lot of defects, sp^3^ clusters, and sp^2^ islands in the form of PAHs can be hypothesized and successfully simulated.^53,79,82^

In addition, the hybridization of the atoms, in particular the ones at the edges of the nanostructure as well as the charge transfer among different species also affect the optical absorption, with the former decreasing and the latter increasing the energy gap.^68^ Finally, the formation of aggregates (such as dimers or excimer)^112,114,160^ and interacting hydrocarbon compounds^53^ profoundly affects the electronic levels of the systems resulting, in some cases, in nontrivial modifications of the optical properties. The formation of n-graphitic layers, besides the effect on their electronic character, is reported to slightly red shift the excited states as compared to the single layer case.^48^ On the contrary, Strauss et al.,^67^ while reporting that increasing the size of the model causes a red-shift of both absorption and emission features, calculated that the formation of a bilayer produces from one side the red shift of the emission from the other the blue shift of the absorption. This is indeed in countertrend with respect to expected stacking aggregation effects, where redshift is typically observed, as reported also for the molecular model.^55^

Another important issue is the effect on the optical properties of CNDs of doping by heteroatoms or the one of functionalization by specific chemical groups. We note that red shift of HL gap was attributed to graphitic N^41,42^ (Figure 38) and edge amino groups.^29,52,65,72^ Conversely, pyridinic and pyrrolic induce a blue shift of the absorption properties.^41,67,72^ For what concerns oxygen related doping, carbonyl^26,60,68,78^ and hydroxyl^26,60,67^ groups are associated with a red shift while the epoxy group is reported to cause a blue shift.^48,67^ Focusing on the impact of functionalization of the emission, several interesting conclusions can be drawn as concisely depicted in Figure 39. It was, in fact, found that the passivation of the GQDs with COOH groups^39,68^ as well as doping with boron^35,66^ or phosphor and sulfur^35^ is strictly related with a redshift in emission. In the case of nitrogen, instead, a crucial role is played by the exact position of the atom inside the system. While a clear redshift is found when NH_2_^29,52,72^ and OH^26,67^ groups are added to the system, evidence of a blue-shift are presented in the case of pyridinic or pyrrolic nitrogen.^67,72^

A similar blue shift effect is found in the case of epoxidation,^67^ with C=N groups playing also a significant role in this sense.^28^ Finally, the effect of inclusion of ether groups in a GQD matrix^71^ is accompanied by a red shift.

The above-mentioned results illustrate the case of doping and functionalization of a graphene-like carbon network. Another important model for the optical properties of CNDs is the molecular one, discussed in section 3.3. The molecular model was proved to be very efficient in describing the bottom-up synthesis, and the most exploited models are the IPCA and CZA molecules for the blue range, the HPPT for the green one, and the combination of PAHs.^46,398,450,456^ Those molecules can reproduce both the absorption and emission properties and could be placed at the surface of the nanoparticle or within the inner structure.^53,55,95^ As already discussed about the effect of the shape of the nanostructure, within the context of the molecular model, a phenomenon that is considered to affect the optical features of CNDs is the formation of aggregates, which are recognized as a possible source of the excitation dependent emission typically recorded. In general, the effect of the formation of dimers or oligomers causes a red shift of the absorption spectrum, and the possible combination of monomers, dimers, and oligomers can modulate the optical features within the visible range.^51,55^ However, the formation of different aggregates with both blue and red-shifted features can eventually lead to single molecule like excitation independent emission, such as for the case of IPCA reported by Langer et al.^73^

The last effect we want to mention is the impact of the solvent environment on the optical properties of CDs. There is an almost general consensus in the computationally oriented works with the observation that the solvent effect is usually of secondary importance compared to the concurrent effect due to doping or functionalization. In particular, only a small red shift is traced in the passage from gas phase to solvent,^26,48^ and the amount of this red shift is slightly more evident in a pyrene-based system surrounded by water.^72^ However, the polarity of the solvent seems to play a very marginal role,^26^ and no evidence at all on the possible impact of surrounding environment on the emission appears noticeable.^26^ One should keep in mind that these considerations are almost unanimously drawn in the general theoretical framework of the implicit solvent formalism (SCRF) which is recognized to not be very suitable to model the possible very strong and localized interactions of the carbon dot surface atoms with very polar solvents (i.e., water). Indeed, several experiments, as in Vallan’s work,^69^ do clearly show evidence of a strong dependence of the intensity of fluorescence with the pH. This suggests the need, in perspective, to turn to combined or hybrid methods for a more accurate description of the impact of the solvent on optical properties. This is the case of the combination of QM and MM methods, in the forerunner Siddique’s work^55^ with IPCA systems or the hybrid QM/MM approach in the following works of Langer.^73,95^

### 3.6 Interactions with Bio/organic Molecules, Inorganic Nanocomposites, and Biomedical Applications

One of the most promising fields of application of CDs is within biomedicine, for bioimaging and recognition of materials and biochemical molecules, such as nucleic acids, proteins, lipids, or carbohydrates. The interactions of biomolecules with CDs can have pharmacological implications and applications and can also be exploited for getting rid of the CD in an eco-sustainable fashion through enzymatic biodegradation.

Theoretical studies of complex systems such as CD + biopolymers, which were not amenable to molecular modeling in the past, has developed greatly in recent years and is contributing substantially to the understanding of how CDs interact with biomolecules of varying size and/or how such interactions are affected by doping and functionalizing of the CD.

The modeling of such complex systems typically requires multiple computational techniques, often involving, besides the QM methods, MM-GBSA, classical force field-based methods, such as MD simulations, or even docking.

In addition to interactions with organic/biomolecules, another emerging use of CNDs in the biomedical field involves their photosensitization capability, which can be exploited for the generation of reactive oxygen species.

Considering the complexity of the system studied in this section, a large part of the reviewed papers, even in those cases where the reference experimental counterpart revealed spherical nanoparticles, use GQDs as model for the CD. This is due from one side to the need of reducing computational cost when performing QM calculations, from the other to the lack of precise structures for CDs, and the simplicity of modeling a single or multiple nanographene layers. We included here also selected cases for which also the available experimental data are mostly referred to GQDs, regarding them as a simplified model for all the CD family, in terms of composition of the exposed surface, so that the reported results and/or the methodological approach are relevant for the whole set of CDs.

#### 3.6.1 Interactions with Small Molecules

##### 3.6.1.1 Glucose

**Figure 47:**
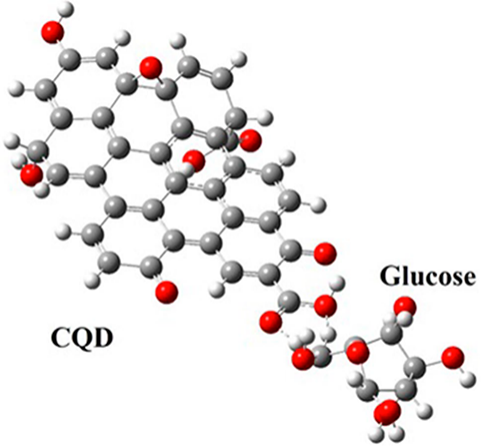
B3LYP/6-31G(p)-optimized structure of CND (referred to as a carbon quantum dot, or “CQD”, by the original authors) interacting with glucose. Reproduced with permission from Sadrolhosseini et al.^33^ Copyright 2019 Springer.

Sadrolhosseini and coauthors^33^ investigated the possibility of glucose detection by CNDs obtained from biochar. The interaction of the CNDs with glucose was studied computationally by DFT; to model the solvent, the integral equation formalism-polarizable continuum model (IEFPMC) method was applied to achieve the minimum energy configuration of both the model CND system and the glucose molecule, separately. The CND was modeled as a system containing 10 fused benzene rings. Based on experimental FTIR spectra, the main CND structure consisted of C–O–C and C=C bonds, with edge C=O and COOH functional groups (Figure 47). The experimental particle size was about 4 nm, while the longest linear dimension of the computational model was about 1–1.5 nm.

From the experimental point of view, the authors reported that the absorption gap of CNDs increased due to the interaction with glucose and that the typical blue emission of the CNDs decreased with increasing glucose concentration. The latter effect is coupled with a change in the emission lifetime. The results were related to the possible interaction between CNDs and glucose by means of hydroxyl and carboxyl groups through H-bonding between the two structures. This hypothesis was supported by DFT calculations, showing a decrease in the total energy of the CND–glucose interacting system.

Although the proposed model is by far a large simplification of the CNDs structure and of its interaction with glucose, it was able to indicate the possible formation of hydrogen bonding between the two compounds. Simulation with more realistic models of CNDs, as those described in the section 2.3.2, belong at least to rung 2 or 3 of our ladder, would certainly help in better understanding this type of interaction.

##### 3.6.1.2 Metal Ions and NPs

**Figure 48:**
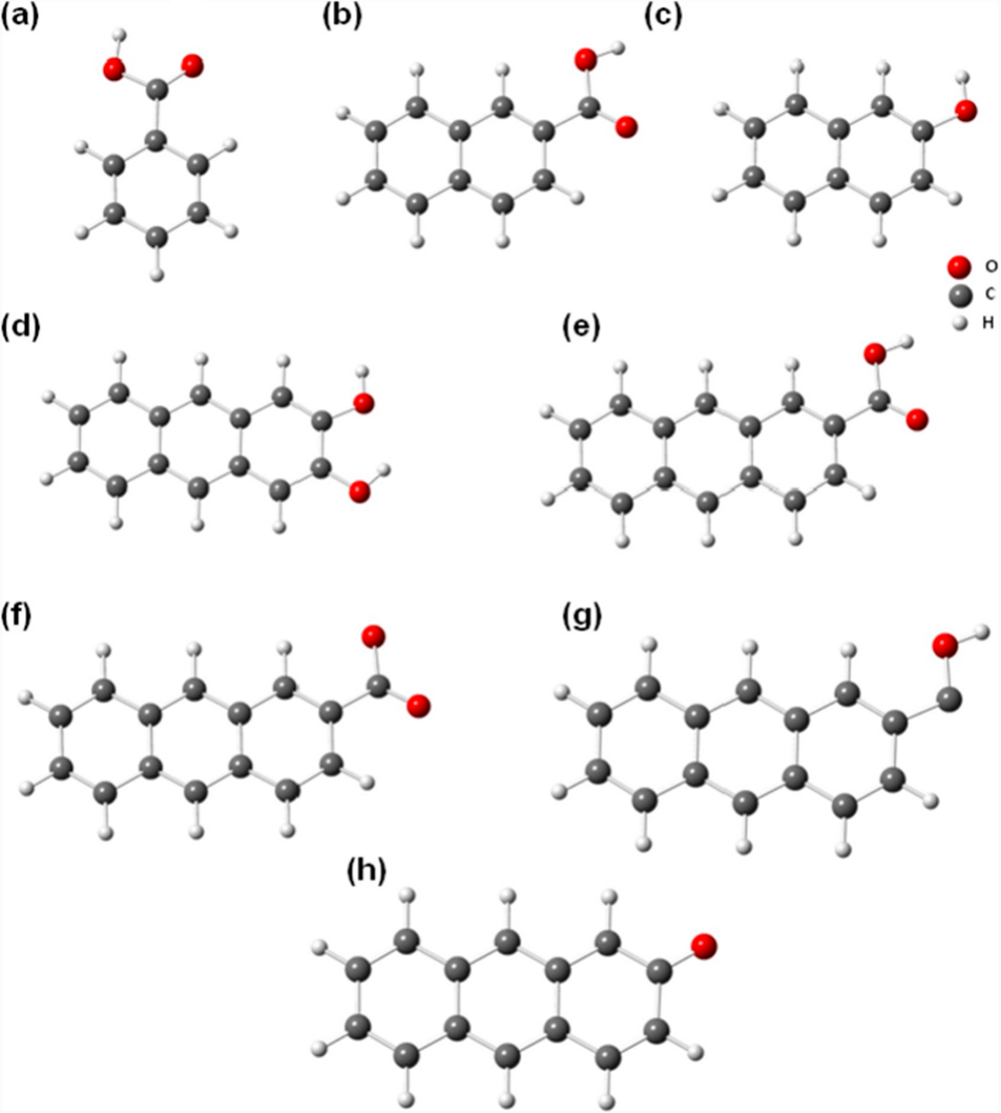
Optimized geometries at the PBE/PW(29.4 Ry) level of theory of model CNDs with oxygen-containing functional groups. In the notation of Ambrusi et al.^60^ 1R, 2R, and 3R indicate structures composed of 1, 2, and 3 fused aromatic rings: the formula following the underscore gives the number of hydroxyl (OH), carbonyl (COO), and carboxyl (COOH) functional groups present in the structure. (a) 1R_1COOH, (b) 2R_1COOH, (c) 2R_1OH, (d) 3R_2OH, (e) 3R_1COOH, (f) 3R_1COO, (g) 3R_1C–OH, (h) 3R_1carbonyl. Reproduced with permission from ref (60). Copyright 2019 Elsevier.

Ambrusi et al.^60^ performed a theoretical study of CND-silver nanoparticle (AgNP) nanocomposite structures, as relevant to the development of detectors for glucose, among other compounds. CNDs were represented by model structures (Figure 48a–h) with hydroxyl (−OH), carboxyl (−COOH), carboxylate (−COO), or carbonyl edge functional groups; the AgNP was modeled as a three-atom Ag cluster (Ag_3_).

DFT binding energy calculation for CND-Ag_3_ structures revealed the strongest adsorption by the −COO functionalized CND (3R_1COO, structure (f) in Figure 45); in fact, the adsorption of this model CND was found to be more favorable by 3.5 eV compared to that of d-glucose (computed separately), leading the authors to conclude that such CNDs should be favored when competing with d-glucose for adsorption sites on AgNPs. Moreover, it seems energetically favorable for CNDs functionalized with −COOH groups to dissociate to −COO anions in order to interact more strongly with Ag_3_, a conclusion perfectly in line with experimental *Z*-potential measurements of a negative charge around CNDs.^506^

The TDDFT-computed UV–vis absorption spectrum of the Ag_3_-3R_1COO CND complex shows, in addition to an intensification of the CND aromatic peak, the disappearance of characteristic Ag_3_ peaks, with the concurrent appearance of a small peak at 335 nm. These observations were attributed to transitions of near-Fermi level O–Ag bond states.

Liang et al.^507^ synthesized blue-emitting nitrogen-doped CDs (N-CDs), which were shown to constitute an “on–off–on” fluorescent sensor for Fe^3+^ ion and glutathione (GSH) detection. Specifically, they found that N-CDs display a strong fluorescence quenching in the presence of Fe^3+^, which is subsequently restored by GSH.

The “turn-off” phenomenon was investigated computationally based on the absorption interaction between N-CDs, modeled as −NH_2_ functionalized coronene structures and a series of metal ions (M^*n*+^ = Fe^3+^, Cr^3+^, Mg^2+^, Ca^2+^, Mn^2+^, Mg^2+^, Co^2+^, Cu^2+^, Pb^2+^, and Zn^2+^) and included an analysis of binding energies (BEs) and frontier molecular orbitals (FMO) of the corresponding complexes. The calculations were performed with Gaussian 09,^145^ using the DFT-D3/B3LYP/6-31+G(d) level of theory for geometry optimization and frequency calculations, with the use of pseudopotentials SDD and Lanl2TZ for N-CDs and metal ions, respectively.

According to geometries, BEs, and FMO results, the N-CDs interact significantly more strongly with Fe^3+^ compared to the other metal ions studied, and the N-CDs-Fe^3+^ complex exhibits the highest stability among the complexes. These results provide a theoretical explanation to the high selectivity and sensitivity of N-CDs toward Fe^3+^ ions.

##### 3.6.1.3 Volatile Organic Compounds

**Figure 49:**
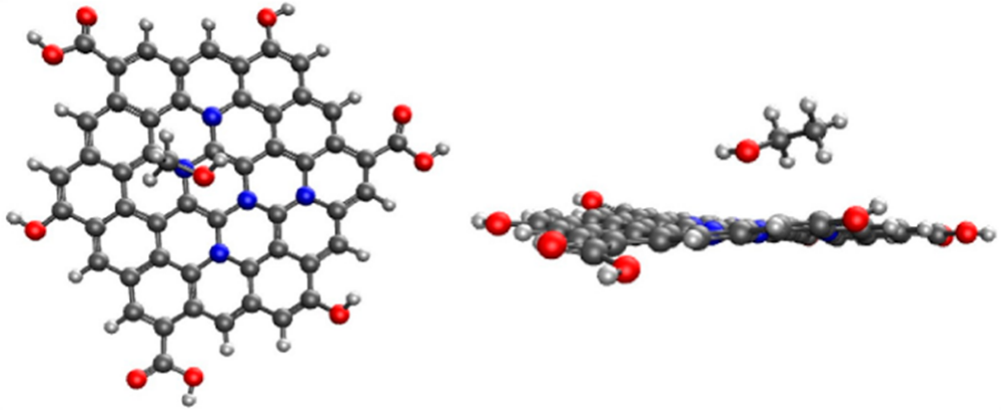
(Left) top- and (right) side-views of the DFT-optimized structure the using PM6/D3H4 semiempirical method of a model CND–acetone complex. Adapted with permission from Thongsai et al.^75^ Copyright 2019 Elsevier.

The optical properties of CNDs can also be exploited for the detection of volatile organic compound (VOC): Thongsai et al.^75^ produced a sensitive optical electronic “nose” to detect acetone vapor at room temperature. TDDFT calculations were carried out on a model CND, a single-layer nitrogen-doped graphitic system (composition C_35_H_16_N_4_O_9_) with hydroxyl and carboxyl group edge functionalization (Figure 49) to assess the interaction between carbon dot and different VOCs, including acetone, hexane, methanol, and ethanol. The absorption spectra of the CND in the presence of different organic solvents were calculated, with solvent effect treated by the polarizable continuum model (PCM). A similar but larger model (C_52_H_18_N_5_O_9_) was used to evaluate CND-VOC dimer complexes, specifically the interaction energies between the CND and the various VOCs; Figure 49 shows the DFT-optimized structure of the CND–acetone complex. Analysis of the interaction energies revealed that the most stable dimer complex was that involving acetone, due to the lower polarity of this molecule as compared to the alcohols and the reduced C–H−π interaction as compared to hexane. The results were taken to indicate that accurate evaluation of the interaction between VOCs and CNDs is of the utmost importance in explaining the sensitivity and selectivity of the electronic nose. Finally, evaluation of the FMOs of the CND in the presence of acetone showed that the CND retains the electron clouds upon excitation, with some transfer to edge functional groups; the authors conclude that this finding confirms the previously reported dominant electron donating- and accepting ability of the CND.^508^ Thongsai et al.^509^ extended this computational approach to study the mechanism by which CNDs (obtained from a different starting material) could be employed to detect and distinguish between methanol and ethanol vapors and those of several other volatile organic molecules.

Singh et al.^54^ developed a CND-based fluorescent sensor for detecting the presence of toxic chlorination disinfection byproducts in water, specifically trihalomethanes (THMs), e.g., chloroform. The fluorescence spectra of the synthesized CNDs were shown to be useful for the detection and quantification of chloroform with high sensitivity (limit of detection 3 ppb). Other THM molecules, including bromoform, bromodichloromethane, and dibromochloromethane were tested for comparison: bromodichloromethane was also found to enhance the CNDs’ photoluminescence, though not as strong as that produced by chloroform; the other two THMs did not show any significant effect. The presence of pyridinic N-oxide groups in the synthesized CNDs was proposed to be responsible for sensitive and selective detection of chloroform.

DFT calculations were performed to deepen the understanding of the role of pyridinic N-oxide for the detection of THMs. CNDs were modeled as small regions of sp^2^-hybridized carbon, made up of 8–10 aromatic rings, with a pyridinic N-oxide functional group at the edge; this group is the expected binding site for THM molecules.

First, the authors calculated the binding energies between the CD pyridinic N-oxide group and the various THM molecules; besides confirming the binding, no relevant differences between the tested species could be identified. Next, FMO analysis revealed that chloroform has no notable wave function overlap with the pyridinic N-oxide group on the CND in any of its low-lying excitations, and consequently it could act as a passivating agent, preventing the nonradiative recombination.^54^ The bromo-form-containing system, on the other hand, displayed a significant wave function overlap, consistent with this THM not enhancing CD photoluminescence. While bromodichloromethane was found to exhibit a behavior between the preceding extremes, consistent with experiment, conflicting results were obtained for dibromochloromethane: this system shows less MO overlap compared to the bromoform case, suggesting somewhat greater CND photoluminescence enhancement, yet experimentally this system exhibits a slight photoluminescence quenching. This discrepancy was proposed to suggest the involvement of some unaccounted-for factor in the interaction between the sensor and analyte, prompting a more advanced computational study.

##### 3.6.1.4 Ammonia

Graphene has been employed in highly sensitive gas sensor applications due to the effect of adsorbed gas molecules on its electrical conductance. Unlike graphene, GQDs have a tunable nonzero band gap which depends on their size and geometry, allowing for their application in electro-optical devices. Seyed-Talebi et al.^510^ studied the adsorption of ammonia onto the surface of pure (C_96_H_24_) as well as boron- and aluminum-doped model hexagonal GQDs using DFT.

**Figure 50:**
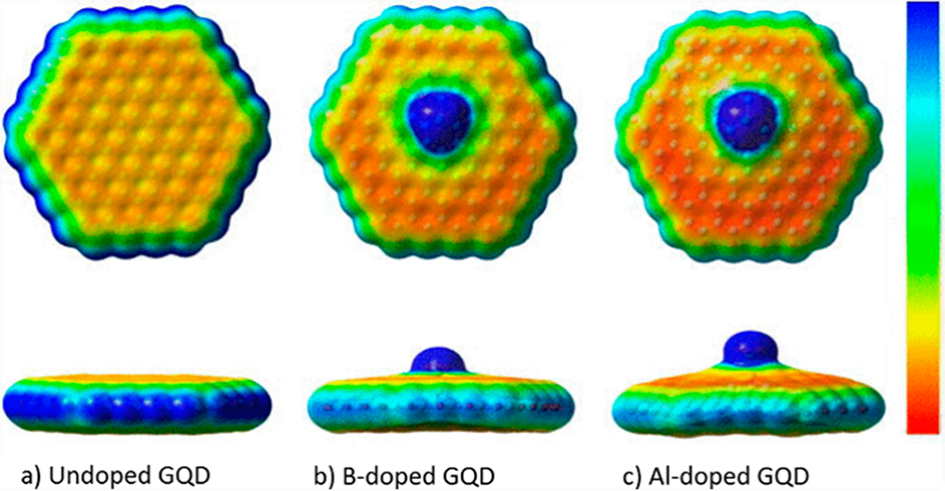
DFT electrostatic potential surface maps of (a) pure, (b) B-doped, and (c) Al-doped GQD models studied by Seyed-Talebi et al.^510^ with a single adsorbed NH_3_ molecule. The 6-31G(d) basis set was used with M06-2X (optimization) and B3PW91 (energies) functionals. The color scale is as follows: red represents an electrostatic potential of −1.5e^–2^ and blue 1.5e^–2^. Note the plate buckling (deviation from planarity) in the B- and Al-doped GQDs. Reproduced with permission from ref (510). Copyright 2013 AIP Publishing LLC.

Model GQDs were prepared with a single dopant atom (B or Al) located in the central GQD hexagon. Pure and B-doped GQDs were found to be planar, while the Al-doped GQD models showed local buckling at the dopant site. A single NH_3_ molecule introduced above the central pure GQD hexagon showed, following DFT geometry optimization, a weak physisorption interaction resulting in an only slight increase of the GQD band gap. Conversely, strong NH_3_ adsorption was found for B- and Al-doped GQDs, inducing local plate buckling (Figure 50a–c). Analysis of the charge distribution revealed a larger charge transfer from NH_3_ to the doped compared to pure GQDs. The authors proceed to characterize the interaction of NH_3_ with the B-doped GQD model as chemisorption with weak charge transfer, while chemical bonding was found in the Al-doped system, which was ascribed to larger local curvature at the Al-dopant site.

##### 3.6.1.5 Borax

Prathumsuwan et al.^76^ synthesized a low-cost fluorescent “label-free” CND probe for borax sensing, a label-free probe being able to generate a signal binding to the target with no need of additional interaction with specific labels. Borax is an important chemical used in numerous industrial and consumer products, but high dosages represent a serious health risk to the human body.^511^ Therefore, the development of a sensitive and cheap sensor is highly desirable for detecting borax residues.^76^ Structural and optical properties of synthesized CNDs were investigated experimentally, followed by tests for borax detection by measuring the quenching of CND fluorescence in the presence of different borax concentrations, also testing for the effect of interference by other compounds. It was demonstrated that the CNDs represent a practical borax sensor with good sensitivity and selectivity. Next, a computational study was performed to deepen the understanding of the fluorescence quenching phenomenon, which is due to a charge transfer process. DFT was used to optimize the geometries for borax (B_4_O_7_H_2_), ascorbic acid (C_6_H_8_O_6_), and CA (C_6_H_8_O_7_) in gas and water phases and to calculate FMOs. Based on experimental FTIR and XPS results, the synthesized CNDs were modeled as a N-doped graphitic layer with oxygen-containing functional groups at the edge (formula C_52_H_18_N_5_O_9_). TDDFT was used to obtain excited states and oscillator strengths of the CND. The HOMO–LUMO level calculations show that electron transfer from the CND to borax is associated with the largest potential drop and consequently the largest driving force for electron transfer after excitation, leading to a more pronounced fluorescence quenching compared to the same phenomenon for ascorbic and citric acids.^512^ This result was consistent with a previous DFT investigation,^513^ indicating fluorescence depletion due to delocalized electrons from a pyreneimine fluorophore onto the boron atom of phenyl boronic acid.^76^

##### 3.6.1.6 Antitumor Drugs

Vatanparast and Shariatinia^63^ studied the role of nitrogen functionalities in drug delivery efficiency of N-doped GQDs (N-GQDs) using classical AA MD simulations and DFT calculations. The interactions of several graphene-based structures (e.g., pristine GQDs and graphitic, pyridinic, etc. N-GQDs) with gemcitabine, a standard drug for the treatment of solid tumors, were studied, with emphasis on the effect of different nitrogen atom doping positions in the structure (e.g., pyridinic at center and pyridinic at edge N-GQDs). From DFT analysis showed that the binding energy of the gemcitabine/N-GQD system was higher when nitrogen was situated at the center of the NP. Nevertheless, even if the values of Gibbs free energy changes indicated that the adsorption process proceeds spontaneously for every model considered, lower values were calculated for adsorption of gemcitabine on the pristine GQDs and edge-doped N-GQDs structures. Moreover, the calculation of quantum molecular descriptors, including the chemical hardness, chemical potential, and electrophilicity index, indicated that the adsorption of gemcitabine enhanced the chemical reactivity. The topological properties were characterized by the quantum theory of atoms in molecules (QTAIM), which revealed that nearly all the interactions of the gemcitabine/N-GQD complexes can be classified as “closed-shell” noncovalent, with notable van der Waals interactions observed with noncovalent interaction index (NCI) analysis. AA MD simulations were performed to explore the drug delivery mechanisms, demonstrating that gemcitabine can be loaded onto the GQDs but favorably released only from the center-doped N-GQDs in acidic cancer tissue environments.

#### 3.6.2 Lipids

Understanding the interactions of CNDs with lipid membranes is of fundamental importance in many areas, especially in nanomedicine and diagnostics. The increasingly studied biomedical applications of CNDs and related graphene-based NPs, e.g., drug delivery, bioimaging, and sensors, require their translocation across physiological cell membranes, for which two essential mechanisms have been identified: endocytosis and direct passive permeation.^285,514^ While the former has been established as the key mechanism, direct permeation is possible for some NPs and may be preferred for some applications, e.g., targeted drug delivery.^285^ Passive permeation may also be affected by application of an external force, e.g., electric field or ultrasound. However, the direct passive permeation mechanism is not yet well-understood nor is its effect on the integrity of the cell membrane itself. Liang et al.^278^ recently reviewed the current state of computational studies of the cytotoxicity of GQDs, notably including various computer simulation studies of GQD–lipid bilayer interactions. Computational studies of “nanotoxicity”, including large-scale computer simulation studies of the interactions between carbon-based nanomaterials and cell membranes (and proteins; see section 3.6.3), were also summarized by Jimenez-Cruz, Kang, and Zhou.^309^

**Figure 51:**
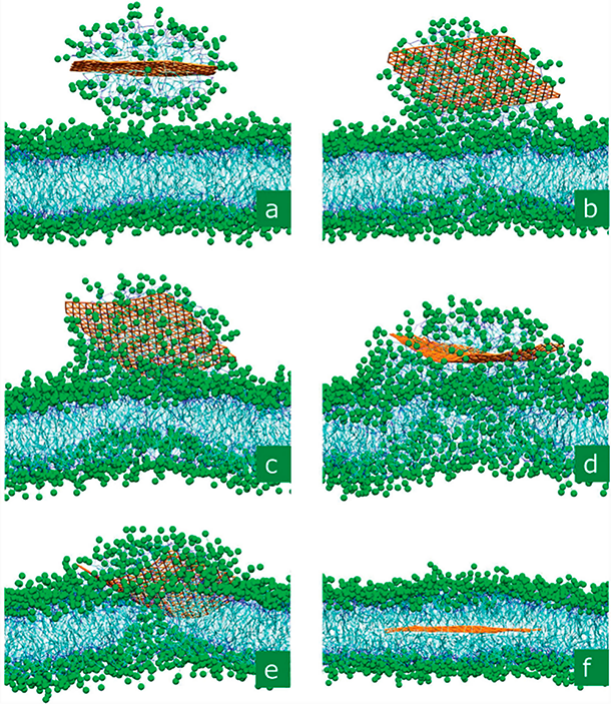
CG MD simulation snapshots showing stages in the self-insertion of a graphene monolayer (brown) inside a POPC bilayer (green lipid heads, blue tails). Simulation times depicted in panels a–f: 2.9, 52.4, 120.0, 299.2, 356.4, and 516.4 ns. Reproduced with permission from Titov et al.^291^ Copyright 2010 American Chemical Society.

Titov, Král, and Pearson^291^ performed CG MD simulations of rectangular graphene sheets imbedded (“sandwiched”) inside the hydrophobic interior of a phosphatidylcholine (POPC) lipid bilayer, so-called “hybrid graphene-membrane super-structures”, as relevant to the development of biosensors and bioelectronic materials. Both the stability and formation of embedded graphene monolayer systems by self-insertion of graphene-containing micelles, i.e., graphene layers covered with phospholipid coating, were studied; see Figure 51. Other sandwiched superstructures, consisting of embedded stacked graphene multilayers, were also evaluated.

CG MD simulations have also been used to study the effect of the degree of GQD oxidation and particle thickness (i.e., multilayer CNDs) on their cell membrane interactions, in particular their ability to penetrate the membrane and the nature of resulting equilibrium structures. Wang et al.^305^ performed CG MD simulations of rectangular graphene nanosheets, both pristine and partially oxidized, interacting with a POPC lipid bilayer using customized graphene parameters; few-layer stacked CNDs and graphene sheet encapsulated by phospholipid coatings of different densities were also considered.

Pristine graphene sheets and few-layer stacked structures were found to spontaneously insert into the bilayer (initiated by nanosheet corner; see below), located in the hydrophobic interior to parallel “sandwiched” structures. Partially edge-oxidized graphene sheets also entered the bilayer, adopting different final (“equilibrium”) orientations depending on the degree of oxidation: 5% edge “carbon” bead oxidation (i.e., substituted for more hydrophilic beads) resulting in the graphene sheet “sandwiched” in the bilayer interior, similar to the pristine analogue, whereas 10% edge-oxidized sheets located perpendicularly across the entire bilayer. Interestingly, it was found that high-density lipid coatings could hinder insertion of graphene sheets into the bilayer.

Li and co-workers^279^ performed a detailed combined experimental-computer simulation study of the spontaneous membrane penetration of graphene (various shapes) and few-layer graphene nanosheets. Their CG DPD simulations revealed that graphene sheets initially penetrate the lipid bilayer through a corner-first approach, i.e., sharp corners or other irregularities initially pierce the membrane. Steered AA MD simulations showed this insertion mode to have a low associated energy barrier, while penetration by long graphene sheet edges (without sharp corners or protrusions) presented a high intrinsic energy barrier. This finding was supported by imaging experiments, and the observed spontaneous penetration of graphene microsheets into cell membranes proposed to result from the fact that these structures rarely exhibit long uniform edges but instead are characterized by nanometer-scale edge irregularities and protrusions.

**Figure 52:**
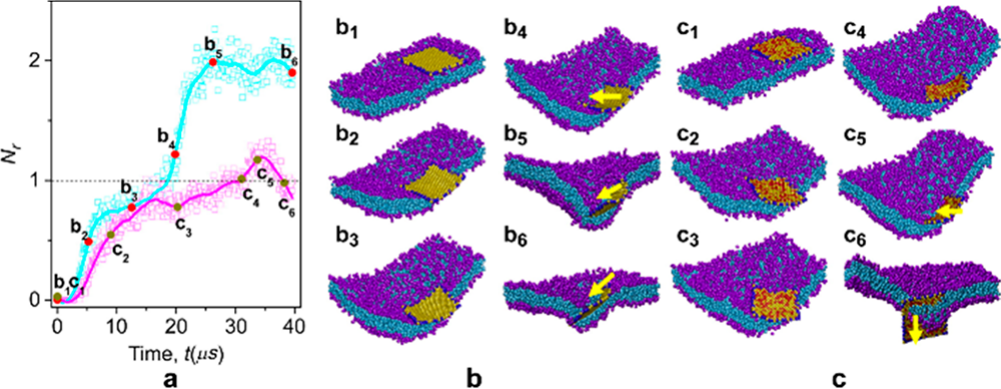
Comparison of lipid bilayer translocation mechanisms of two graphene nanosheets, edge-oxidized (labeled “b”), and densely oxidized (“c”). Panel a shows the time-dependent lipid head coordination number, *N*_r_, to nanosheet beads. Simulation snapshots in panels b and c refer to correspondingly labeled points in panel a. Pristine graphene nanosheet beads are shown in yellow, with edge and basal plane oxidized beads colored blue and red, respectively; lipid heads are shown in purple, tails in cyan. Reproduced with permission from Mao et al.^306^ Copyright 2014 Elsevier Ltd.

Mao, Guo, and Yan^306^ performed mesoscale CG DPD simulations to study the mechanism of interaction and translocation pathways of pristine and partially oxidized (both sheet edge and basal plane) rectangular graphene nanosheets with a model lipid bilayer, also evaluating the role of bilayer perturbations in this process. The simulations made use of specially developed CG bead interaction potentials and associated parameters and allowed for the construction of a “phase diagram” of graphene–membrane interaction states in the space of the degree of nanosheet oxidation and size; higher oxidation degrees were shown to induce greater membrane perturbation, which was exacerbated with increasing the nanosheet edge length. These findings were compared with experiment and the simulations further analyzed to obtain detailed insight into the cellular internalization mechanisms of such graphene nanosheets. Representative simulation snapshots showing the different bilayer translocation mechanisms of nanosheets of different oxidation degree are shown in Figure 52a-c.

**Figure 53:**
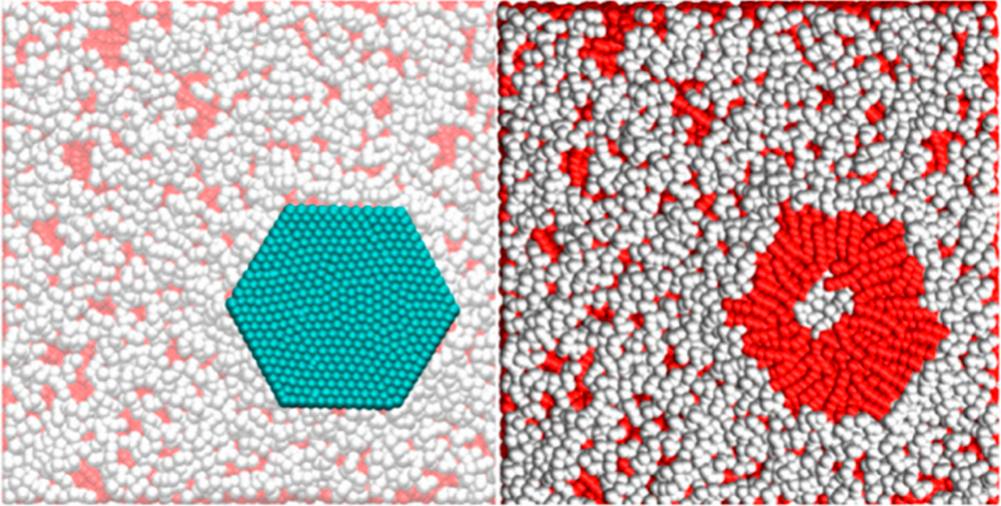
MD configuration showing a large hexagonal GQD (cyan) adhering to the outer surface of a phospholipid bilayer (lipid heads in white, tails in red) (left) and the same lipid bilayer configuration but with the GQD removed so as to show upturned/extracted lipid tails interacting with the GQD (right). Reproduced with permission from Dallavalle et al.^307^ Copyright 2015 American Chemical Society.

The effect of graphene sheet size in determining the mode of interaction with a phospholipid membrane was studied computationally by Dallavalle et al.^307^ Their CG DPD simulations, which also made use of specially developed parameters, revealed interesting changes in the interactions between pristine hexagonal graphene monolayers (nanosheets) of increasing size (0.9–13.3 nm across) with a model phospholipid bilayer: small sheets (<5.2 nm) are able to pierce the bilayer and navigate the hydrophobic interior, whereas those of increasing size (up to 11.2 nm) were found to pierce and then cross the bilayer only in the case of suitable geometric orientations. Graphene sheets larger than 11.2 nm were unable to cross the phospholipid bilayer and adsorbed on the outer surface. This surface-adsorption mode was found to result in considerable local disruption of the native bilayer structure, with phospholipids overturned (or “upturned”, as shown in Figure 53); this observation was noted to be consistent with previous experimental and computational findings of size-dependent cytotoxicity of graphene sheets, specifically the so-called “masking” effect of adsorbed/adhering sheets.

More recently, Chen et al.^308^ reported CG DPD simulation results on the translational motion of graphene oxide (GO) nanosheets sandwiched inside a lipid bilayer; different degrees of graphene oxidation could be modeled by modifying the Flory–Huggins parameter, χ_GT_, describing the interaction strength between the nanosheet beads and those of the lipid tails. While low oxidation degrees (low χ_GT_, corresponding to strong GO-lipid tail interaction) are characterized by Brownian diffusion of sandwiched nanosheets within the bilayer, increasing graphene oxidation (increasing χ_GT_) was found to lead to persistent nanosheet center-of-mass walks interrupted by local jiggling (Lévy dynamics) and, ultimately, directional translation at high oxidation. These motional changes were shown to correlate with the formation of “hemi-pores” of varying stability in the lipid bilayer (i.e., one layer, or “leaflet”, of the bilayer) induced by the sandwiched GO nanosheet: unstable and metastable pores resulted in directional movements of the sandwiched GO nanosheet, reverting to diffusive “jiggling” upon closing of the pores; stable pores formed at high GO oxidation (weak GO-lipid tail interaction) lead to constant directional translation. The authors also present results demonstrating the applicability of bilayer-sandwiched GO nanosheets in enhancing the delivery efficiency of membrane-specific drugs.

**Figure 54:**
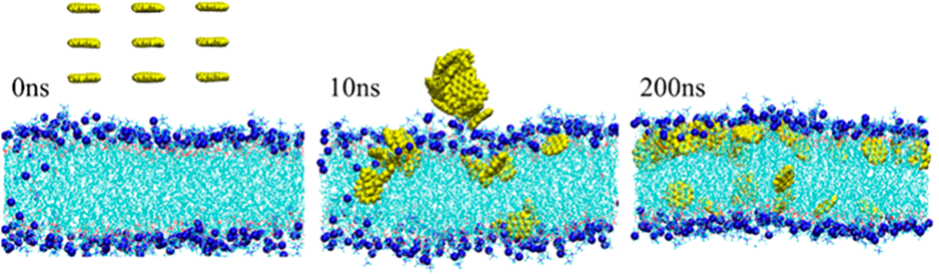
MD simulation configurations of small pristine GQDs interacting with a POPC lipid bilayer, showing GQD aggregation in water and subsequent dispersion within the hydrophobic lipid bilayer interior. Reproduced with permission from Liang et al.^280^ Copyright 2016 American Chemical Society.

Reports of atomic-resolution (AA) MD simulations of CNDs/GQDs interacting with lipid bilayers have increased in recent years, reflecting the ever-increasing computational capacity for dealing with such large model systems. For example, Liang et al.^280^ reported on the size-dependent membrane permeation and cytotoxicity for pristine GQDs, based on their atomistic MD simulations, in which small GQDs were found to spontaneously enter the lipid bilayer without significant mechanical damage to the bilayer structure, suggesting low cytotoxicity. Under high concentrations, such small GQDs were found to aggregate in the aqueous medium but dispersed upon entering the hydrophobic bilayer interior. Interestingly, instead of localizing in the middle of the hydrophobic region formed by the lipid tails (i.e., “sandwiched”), the GQDs were shown to distribute on either side, near the lipid heads (Figure 54). High GQD concentrations were found to affect the bilayer structure and lipid diffusion.

Yao et al.^293^ exploited GQD structural and optical properties to target cancer cell nuclei in vivo and in vitro, taking advantage of the high interstitial fluid pressure (IFP) which allows the penetration of such nanosystems into the tumor cell membrane with minimal uptake by cells in normal tissues. The authors synthesized negatively charged GQDs functionalized with sulfonic and hydroxyl groups, which were shown to be suitable nanomarkers for many applications in cancer therapy and diagnosis, as opposed to previously synthesized toxic amino-GQDs.^515^ AA MD simulations were performed to better understand the targeting mechanism through which these negatively charged GQDs were able to cross the cell membrane and effectively target nuclei, while their positive counterparts showed the opposite behavior. Two oppositely charged amphiphilic GQD models were simulated: the sulfonic-GQDs were modeled as a hydrophobic nanosized graphene monolayer functionalized with hydrophilic SO_3_^–^ and OH groups at the edge sites, whereas the amino-GQDs were represented with the same basic structure but with hydrophilic NHNH_3_^+^ and OH groups covalently bound to it. The tumor cell membrane was modeled as a negatively charged phospholipid bilayer, for which the Berger lipid force field was adopted.^516^ The MD simulations revealed differences in the phospholipid bilayer translocation and detachment of the two types of functionalized GQDs, following similar starting positions in the hydrophobic interior of the bilayer. Upon detaching from the bilayer, the positively charged amino-GQDs extract a coating of lipid molecules adhering to the graphene plane, with their head groups interacting with the edge of the amino groups. Conversely, the negatively charged sulfonic-GQDs do not display this phenomenon because of electrostatic exclusion of the lipid molecules from the surface, a so-called “self-cleaning” bilayer detachment. This repulsive force combined with the ideal amphiphilic structure of the sulfonic-GQDs could represent the key factor to avoid the disturbance of nuclear targeting by absorbed biological molecules and reduce systemic toxicity and immune responses.^293^

Vatanparast and Shariatinia^63^ performed a rather extensive study of the membrane permeation of GQDs and N-GQDs loaded with an antitumor drug, gemcitabine. Their steered AA MD simulations revealed differences in the force required to pull GQD/N-GQD-gemcitabine complexes across a DPPC lipid bilayer at the various stages of the permeation process. Notably, they found that, once the complexes were located within the hydrophobic bilayer interior, a greater force was needed to pull drug-loaded N-GQDs out into the aqueous phase (where the drug would be delivered) compared to drug-loaded GQDs, which represents a potential hurdle in the application of the N-GQD carriers. However, it was ultimately established that center-doped N-GQDs exhibit generally more favorable overall drug delivery characteristics (see section 3.6.1.5).

**Figure 55:**
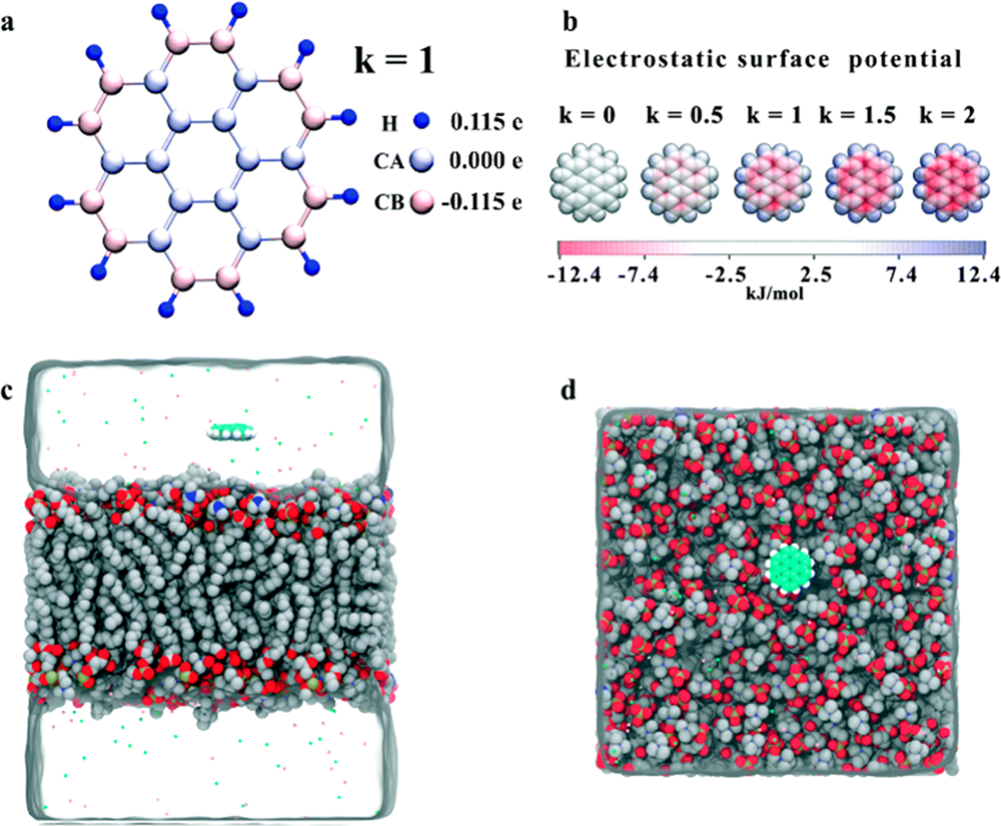
(a) Detailed description of the essential circular GQD model. (b) Electrostatic surface potentials (EP) of GQD models with different partial atomic charge scaling factor (*k*) values. Side (c) and top (d) views of the MD simulation starting configurations: the GQD is located above the POPC bilayer. Reproduced with permission from Tang et al.^281^ Copyright 2020 Royal Society of Chemistry.

The role of doping atoms and consequent electrostatic potential (EP) polarization at GQD edges in their translocation into cell membranes was recently studied by Tang et al.^281^ The authors performed AA MD simulations using a previously described pristine circular GQD model,^280^ based on fused benzene rings with edge sites saturated by hydrogen atoms (Figure 55a); only these terminal hydrogen atoms and their directly bonded edge carbon atoms are assigned partial atomic charges. The surface EP of the GQD model (Figure 55b) was then modified by rescaling of these edge atomic charges by a constant factor 0 ≤ *k* ≤ 2, adjusted in increments of *k* = 0.5, resulting in five types of GQDs with increasing polarization, mimicking the effect of edge functionalization/doping. The dynamics and spatial distribution of these GQDs interacting with a POPC lipid bilayer were analyzed (Figure 55c,d), and the potentials of mean force for the GQD–bilayer interaction and GQD hydration free energies were calculated. In the unpolarized state (*k* = 0) and at low EP polarization, GQDs spontaneously entered the POPC bilayer and localized in the hydrophobic interior; increasing EP polarization leads to a shift in the GQD distribution maximum from the lipid tail region (two peaks, one for each bilayer “leaflet”) toward the hydrophilic head groups. GQDs with highly polarized EP effectively remain associated with the lipid head groups, localized at the water interface (i.e., adsorbed on the outer surface of the bilayer); at *k* = 2 (highest polarization), the GQD was also found to temporarily detach from the bilayer surface, highlighting the importance of GQD–water interactions in the lipid bilayer translocation process.

**Figure 56:**
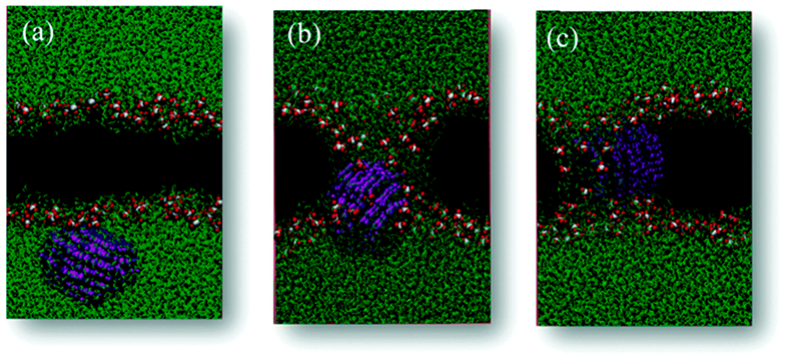
Simulation snapshots of different CND center-of-mass positions perpendicular to the POPC lipid bilayer center: (a) 3.5 nm, (b) 1.44 nm, and (c) 0.00 nm. The CND is shown using blue/purple spheres, water molecules using a green line representation, and lipid headgroups using a space-filling representation (silver and red showing the P and O atoms, respectively); lipid tails are omitted. Reproduced with permission from Erimban and Daschakraborty.^285^ Copyright 2020 Royal Society of Chemistry.

Erimban and Daschakraborty^285^ performed a classical MD simulation study of the direct passive permeation of a model spherical, surface hydroxyl-functionalized CND across a POPC lipid bilayer. The permeability of the CND across the lipid bilayer, calculated using the inhomogeneous solubility diffusion (ISD) model,^517,518^ was found to be negligible at both 300 K and 320 K; an energetic analysis revealed a prohibitive enthalpy increase due to the reduction of CND–water H-bonds as the CND is driven (by application of an external force) to the bilayer interior. The cytotoxicity of CND forced permeation was evaluated by determining the effect of dragging of the CND across the POPC lipid bilayer (Figure 56) on the bilayer structure. Several bilayer structural parameters were evaluated, namely, average area per lipid molecule, electron density profile, bilayer thickness, and lipid acyl chain order parameters.^519^ Only slight changes in these descriptors were observed when the CND adsorbs to the surface of the bilayer, suggesting inherently low cytotoxicity, though forced translocation proved detrimental to the bilayer structure. The extent of water incursion into the lipid bilayer, or water pore formation, upon dragging of the CND across the bilayer was also investigated (Figure 56a–c). It was found that water molecules, H-bonded to the CND, entered the bilayer interior during this process and that lipid head groups could also be extracted from the bilayer by the CND. The results of Erimban and Daschakraborty support endocytosis as the key mechanism of CND translocation across cell membranes, suggesting also low cytotoxicity of hydroxyl-functionalized CNDs adsorbed to the membrane surface. However, forced permeation of such CNDs may prove detrimental to the membrane structure and result in the formation of water pores.^285^

A number of model types have been proposed to describe the passive permeation of NPs into lipid membranes,^520−522^ including studies of the interactions of NPs with lipid head groups, the “head group gating effect”,^523,524^ thought to be one of the main limiting steps. Liu et al.^284^ introduced a theoretical model, based on a combination of classical MD simulation and statistical methods, for predicting the time of entry of simple NPs in lipid membranes (i.e., permeation of the hydrophilic region of the membrane) under physiological conditions. Notably, the model identifies key parameters that describe the permeation process and factorizes them into those contributions dependent on the membrane only (lipid density distribution) and those depending only on the NP (size, shape, solubility).

Several classical AA MD simulations of different carbonaceous NPs in a POPC/cholesterol (10:1) bilayer were performed by the authors to identify membrane permeation trends to be included in the model. Three carbonaceous NPs were selected for their relevance in biomedical applications and a similar size yet different shape and hydrophilicity: (1) a buckminsterfullerene (C_60_), (2) a curved OH-terminated (edge functionalized) graphene quantum dot (GQD), and (3) the same GQD functionalized with two cysteine groups (cys-GQD). The simulations allowed for analysis of membrane lipid low-density area (LDA) distribution and dynamics due to thermal fluctuations; knowledge of these properties is key to describing the probability of a NP encountering an LDA of appropriate size to allow for membrane permeation. The model, which ultimately describes the time of permeation (i.e., the time elapsed from when NP encounters membrane lipid hydrophilic headgroups until it emerges at the hydrophobic lipid tails), was tested against computer simulation and experiment and found to provide very good results. POPC/cholesterol bilayer permeation times for C_60_ and GQDs agreed well with those obtained from MD simulation; the leakage of GQDs from lipid vesicles, monitored experimentally by photoluminescence imaging, was well reproduced by the model permeability. Prospects for adapting the model for the description of more complex systems, e.g., charged NP, which affect the characteristic membrane dynamics, are also described.

#### 3.6.3 Proteins

Several investigations aimed at understanding the interactions between CDs and proteins have been performed in recent years. These studies are fundamental to the computational modeling of nanomaterial-protein systems for future developments in nanoscience and nanotechnology. Atomistic classical computer simulations constitute an important modeling technique of such systems, with progress in this field recently reviewed by Ganazzoli and Raffaini.^525^

CNDs have been studied as promising inhibitors for the formation and development of amyloids,^526^ protein aggregates with fibrillar morphology which are associated with numerous human pathologies, including Alzheimer’s and Parkinson’s disease. Thus, protein fibrillogenesis inhibition represents an important potential medical application of CNDs.

Yang et al.^275^ used a combination of computational methods and experimental measurements to understand how the surface properties of CNDs affect the fibrillation of human insulin (HI). Their study focused on the importance of oxygen-containing functional groups at the surface of the CNDs on the formation of HI aggregates, analyzing the effect of the degree of CND oxidation on the fibrillation process in vitro and with classical MD simulations. The surface composition of the CNDs was analyzed experimentally by XPS, *Z*-potential, and pH measurements. The XPS measurement allowed for the identification of different types of chemical bonds involving carbon and oxygen atoms, such as C=O, OH, C–O–C, and for evaluation of their relative content, which depends on the oxidation state. An increase in oxidation degree is accompanied by an increase in the acidity of the CNDs. The TEM analysis shows that the average size (2.5 nm diameter) of the CND particles is not affected by the oxidation process; similar conclusions were reached with mass spectrometry analysis. Analysis of excitation-dependent emission spectra, in combination with the CND size information, indicate that surface defects formed during oxidation produce a shift in the maximum emission-wavelength-dependent fluorescence to lower wavelengths. The effect of CND oxidation on the HI fibrillation process was analyzed through thioflavin assays and showed oxidized CNDs to be more active than their pristine counterparts in inhibiting protein aggregation; the oxidation degree was also found to have an important effect.

In order to explain why oxidation of the CND surface promotes fibrillation inhibition, the authors used a combination of QM calculations, MD simulations, and MM-GBSA free energy calculations. QM calculations were limited to the parametrization of the CND model. As in many other studies of CNDs, since it is not possible to experimentally characterize the detailed structure of the CND, the authors made a reasonable guess as to the surface structure on the basis of its chemical composition, and the NP surfaces were modeled as smaller molecules.^275^ MM-GBSA calculations were used to obtain the binding free energies of the pristine and oxidized molecules with native and unfolded HI. The analysis of interactions at the molecular level afforded by the MD simulations indicates that the presence of carboxyl groups at CND surfaces increases the inhibition of the fibrillation by increasing the electrostatic interactions with the positively charged histidine on the B-chain of HI, and in turn, this interaction prevents the protein from unfolding.

CDs, and GQDs, are also promising inhibitors of human islet amyloid polypeptide (IAPP), a peptide cosecreted with insulin by pancreatic β-cells. Since amyloid aggregation of peptides and proteins is a fingerprint of neurological disorders and type 2 diabetes, GQDs can be employed to drive the interaction of IAPP with pancreatic cell line, to achieve correct cellular protein expression. Wang et al.^527^ studied the ability of GQDs to mitigate the aggregation and toxicity of IAPP in vivo by a multitechnique approach, where MD simulations were used to help understand the interaction between GQDs and monomers or dimers of IAPP. All-atom discrete MD simulations were carried out on model GQDs of diameter 3.0 nm (C_317_O_81_H_39_), exploiting a Medusa-like FF^528^ to mimic van der Waals interactions, solvation, H-bonding, and electrostatic interaction between GQDs and IAPP. In the presence of GQDs, both intra- and interchain interactions were significantly reduced due to the strong binding between IAPP and GQDs driven by hydrophobic interactions, aromatic stacking, H-bonding, and electrostatic interactions, affecting both the primary and secondary structures of IAPP. The experimental biophysical characterization revealed a significant decrease in IAPP aggregation in the presence of GQDs, due to the coil unstructuring effect and the inhibition of IAPP self-association exerted by GQDs, as shown by MD simulations.

The combined experimental and computational work of the Faridi group^529^ has shown that GQDs have a remarkable capacity to regulate protein expression, reducing aberrant ones through H-bonding and hydrophobic interactions. MD simulations carried out to investigate the interaction between IAPP and GQDs showed that the structured conformation of IAPP monomers (helix and β-sheet) was completely destructed due to IAPP preferential H-bonding with GQDs, leading to adsorption on the GQD surface. Conversely, oligomers of IAPP adopted a structured helical-rich conformation with many internal H-bonds and a low β-sheet content in the presence of GQDs. Thus, GQDs have different effects on the secondary structures of IAPP monomers and oligomers. Finally, a 20-peptide IAPP fibril was simulated to interact with five GQD nanosheets, showing GQD binding capacity to both the elongation and secondary nucleation surfaces of the fibril, thus hindering growth of the fibril itself. The H-bonding of GQDs to the main and side chains of the IAPP fibril was found to be the driving mechanism for a reduced interaction of IAPP fibrils with target cells. Experimentally it was also shown that GQDs mitigated the toxicity of IAPP in vitro, further promoting the exploitation of GQDs to regulate protein expression.

It is worth noting that in view of biomedical applications, while the biodegradability of many other carbon nanomaterials by human peroxidases has been demonstrated, GQDs have not yet received the same attention. To this end, Martín et al.^276^ have performed a combined experimental and MD simulation study of the degradation of model synthesized GQDs by human myeloperoxidase (MPO) and eosinophil peroxidase (EPO). The enzymatic degradation of synthesized GQDs, composed of 1–2 graphene layers with hydroxyl group edge functionalization, by MPO and EPO was monitored by a combination of high-resolution TEM, fluorescence, and Raman spectroscopy. Both enzymes were found to significantly degrade the synthesized GQDs at 37 °C in the presence of hydrogen peroxide within the experimental time frame (40 h).

**Figure 57:**
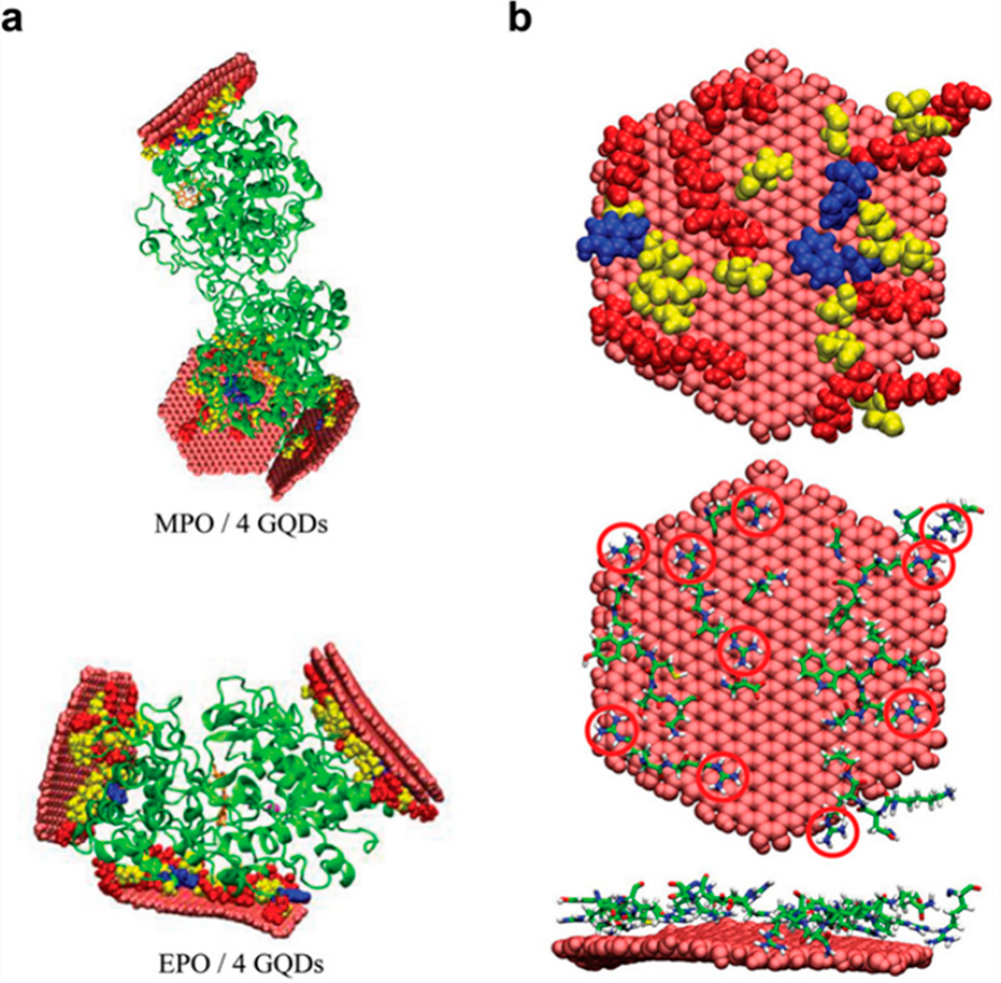
(a) MD simulation configurations of MPO (top) and EPO (bottom) and four model GQDs. (b) EPO residues interacting with EPO; top panel shows positively charged residues in red, aromatic residues in blue, and others in yellow. Middle panel shows guanidine groups of Arg encircled. Reproduced with permission from Martín et al.^276^ Copyright 2019 Wiley-VCH.

MD simulations, using the CD force field parameters developed by Paloncýová et al.,^271^ were performed to help understand the nature of interactions of the GQDs with MPO and EPO. During the MD simulations, the peroxidases were found to directly interact with up to 6 GQDs. Adsorbed GQDs were found to remain at essentially fixed positions on the enzyme surface for the entire 100 ns simulation trajectories and interacted with free GQDs, resulting in the formation of stacked aggregates of up to a few layers around the enzyme (Figure 57a). The authors report that the enzymatic active sites of both MPO and EPO appeared to be inaccessible to the model GQDs. In the case of MPO, surface positions (Figure 57b) consisting of mobile amino acid chains were favored for GQD adsorption; these mobile chains (consisting primarily of charged residues) were found to flatten and fit perfectly to the GQD surface. Finally, an RMSD analysis revealed that enzyme conformations are slightly altered to favor interactions with adsorbed GQDs; however, the GQDs also appear to constrain the enzymes. GQDs adsorbed to enzyme surfaces also exhibited flexibility, structural distortion, and overall curvature.

Another important emerging therapeutic application of GQDs concerns their antibiofilm activity, which can be used to reduce the formation of *Staphylococcus aureus* (SA) biofilms, a major cause of bacterial infections in hospitals and communities. The presence of GQDs dramatically reduced the fibrillation of SA-related peptides (phenol-soluble modulins, PSM), disrupting of the structure and morphology of mature fibers.^297^ It was shown experimentally that GQDs are not intrinsically toxic to the bacteria cells but acted on the protective extracellular matrix via the N-terminal of the PSM.^297^ Classical AA MD simulations supported the proposed GQDs–PSM interactions, showing these to effect changes in the secondary structure of individual peptides, ultimately inhibiting fibrillation.

The interactions between GQDs and ubiquitin were studied by Fang et al.^304^ using a combination of experimental techniques (SPR, CD, NMR) and classical MD simulations. Their simulations, in which the protein ubiquitin was allowed to interact freely with an infinite pristine graphene sheet, revealed two essentially different ubiquitin adsorption types, based on the protein amino acid residues at the ubiquitin–GQD interface: one with and one without involvement of the flexible ubiquitin C-terminal. In both modes, the interface is primarily composed of hydrophobic residues, and the secondary protein structure of adsorbed ubiquitin was found to be preserved. While the identified interfacial ubiquitin residues were shown to be largely consistent with experiment, the simulations did not reproduce the significant reduction in β-sheet content observed experimentally; the authors note that this discrepancy is most likely due to insufficient simulation time.

Kim et al.^530^ studied the interactions between GQDs and α-synuclein (α-syn), the aggregation of which has been correlated to the emergence of Parkinson’s disease, specifically the accumulation and transmission of α-syn aggregates in the midbrain. The authors investigated the activity of GQDs as potential inhibitors of α-syn fibrillization experimentally by thioflavin T (ThT) fluorescence measurements, turbidity assays, and TEM analysis. The presence of GQDs was shown to strongly inhibit α-syn fibrillization by dissociation of α-syn fibrils into short fragments; their average length reduced from 1 μm to 235 nm after 6 h exposure and further to 70 nm after 24 h.

To elucidate the mechanism of GQD action, a 200 ns classical AA MD simulation was performed of an α-syn fibril (experimental solid-state NMR structure, of which only the essential nonamyloid-β component domain considered in the simulation) interacting with GQDs with carboxyl-functionalized edges. The simulation showed that a single GQD was able to bind to the N-terminal cross-β part of α-syn within only 1 ns. After a further 50 ns of simulation, the β-sheet structure of the outer monomer was destroyed, its C-terminal detached and interacted with the opposite plane of the GQDs. An analysis of the protein secondary structure revealed a corresponding decrease in the β-sheet component of the outer monomer from 50 ns onward, indicating a clear structural disruption in the α-syn fibril. These results, along with additional experimental work on the ability of GQDs to cross the blood-brain barrier and their role in preventing neuron death, paved the way for novel strategies based on the use of GQDs in clinical drug development against Parkinson’s disease, as potential inhibitors of α-syn aggregations without severe toxicity.

The potential application of CNDs for protein discrimination was recently studied both experimentally and computationally by Carneiro Cruz et al.^531^ CNDs synthesized by CA thermolysis were chosen as probes, the fluorescence of which could be modulated through interaction with specific metal cations and proteins. The optical and structural properties of the synthesized CNDs were characterized by absorption and emission spectroscopy, FTIR and Raman vibrational spectra, XPS, and AFM measurements. The analysis resulted in typical CA-derived CDs, with excitation dependent blue emission, diameters of nanoparticles in the 1.2–2.5 nm range, and evidence of contemporary ordered and disordered carbon vibrational signatures. The detailed solution structure of the CNDs was investigated by means of MD simulations, the technical details of which were not described by the authors.

**Figure 58:**
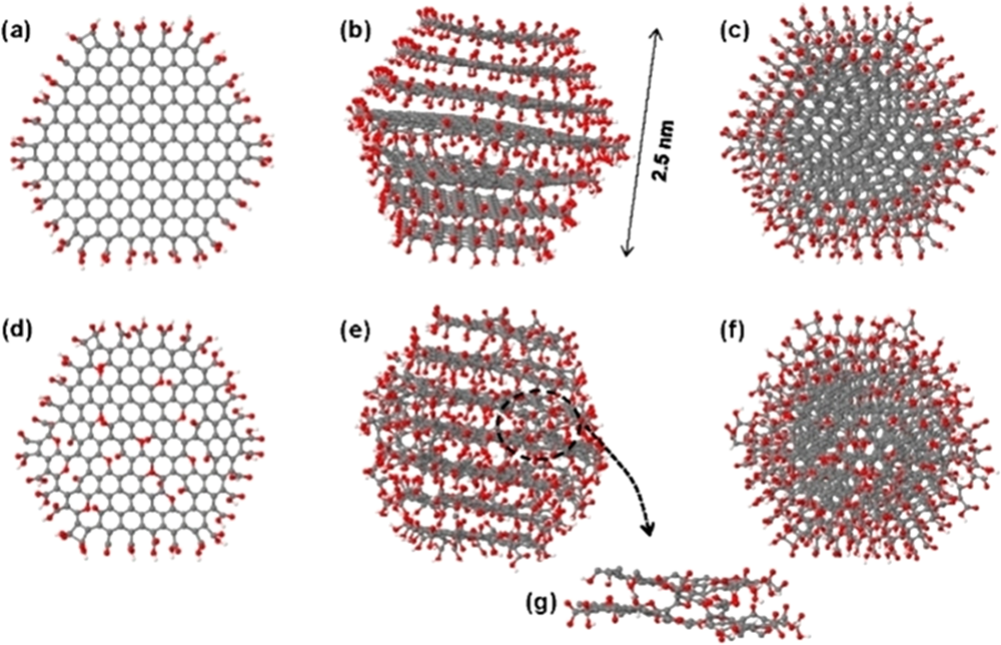
CND models produced using MD by Carneiro Cruz et al.^531^ Graphene layers with edge-only oxygen-containing group functionalization (top). Similar to the top row but with oxygen-containing defects introduced on the graphene layer basal plane (bottom). Reproduced by with permission from ref (531). Copyright 2019 Wiley-VCH.

The CNDs were initially modeled as quasi-spherical NPs of an effective diameter 2.5 nm (as indicated by AFM), consisting of stacked graphene flakes with hydroxyl- and carboxyl-group edge functionalization (Figure 58a–c). Graphene flakes of decreasing diameter were stacked on both sides of the central layer until the NP height effectively matched the central layer diameter (2.5 nm). Relaxation of this starting structure resulted in an average interlayer spacing of ∼0.34 nm, consistent with previous work on stacked graphitic layers, with sp^2^ C atoms in the core region of each layer and sp^3^ C atoms at the surface. The elemental composition of this model structure, however, was found to be inconsistent with that obtained experimentally, underestimating the relative oxygen content. Thus, a second model was considered (Figure 58d–f), in which oxygen-containing defects were introduced within the sp^2^ basal network of each layer. The presence of these defects promoted the formation of interlayer covalent bonds (e.g., Figure 58g), reducing the interlayer distance and increasing the stability of the overall structure but severely affected the graphitic ordered structure. The final nanoparticle is largely amorphous, suggesting an active role of oxygen doping in determining the final structure of such CNDs.

**Figure 59:**
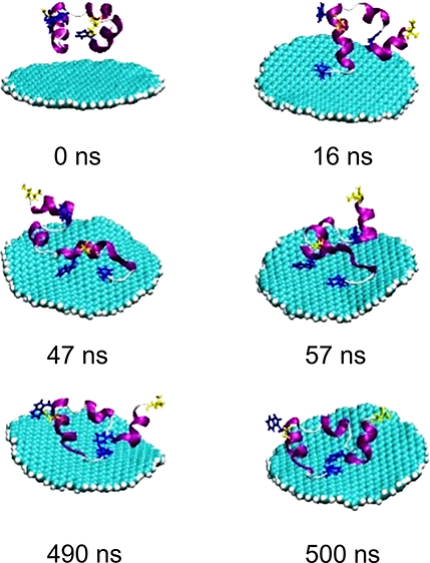
MD simulation configurations showing an HP35 protein interacting with a large pristine GQD, composed of 275 fused benzene rings, as a function of time. Aromatic protein residues are shown in blue, others yellow. Adapted with permission from Zhou et al.^277^ Copyright 2018 Elsevier B.V.

Even though these results are interesting, we cannot assess how reliable they are. Indeed, it is difficult to reach any conclusion on the proposed model without proper information on the simulation details. As already discussed, the interactions of biomolecules with the nanosized-surfaces of GQDs can modify the structure, biochemical properties, and functions of the former. Such modifications may have important consequences, for example, in diseases associated with conformations or aggregation of such biomolecules (e.g., Parkinson’s disease, diabetes). Thus, understanding the interaction of specific proteins with GQDs is crucial to realizing the potential of these materials in biomedicine, also through the evaluation of their cytotoxicity. Zhou and co-workers,^277^ investigated the size effect of GQDs on the adsorption of the HP35 protein, an established model system for studies of protein folding. MD simulations were performed to study the interactions of HP35 with GQDs of different sizes. The GQDs consisted of 7, 19, 61, or 275 fused benzene rings, with the edges of all structures terminating in H atoms (i.e., “pristine” GQDs). The results showed that the π–π stacking interaction between aromatic residues of the protein and the surface of GQDs plays a key role in driving the adsorption of HP35. The larger the GQD, the more protein binding sites interact with it and the greater the GQD-protein binding strength, which has important consequences for the structure of the adsorbed protein (Figure 59). Specifically, it was shown that increasing the GQD size resulted in increasing structural change of the adsorbed HP35, based on protein RMSD and secondary structure analyses. This study contributed toward understanding the GQD size effect on cytotoxicity.

#### 3.6.4 Nucleotides and Nucleic Acids

Wang et al.^301^ conducted a computational study aimed at understanding the molecular interactions of different types of carbon nanoparticles (CNPs) with a double-stranded DNA fragment (dsDNA). MD simulations were performed for various CNPs, including a GQD, graphene oxide quantum dot (GOQD), two fullerenes, C_60_ and C_70_, (8,0) single-walled carbon nanotube (SWNT), and (8,0) double-walled carbon nanotube (DWNT). Optimized geometries and interaction energies were calculated for each CNP–dsDNA complex to analyze the interaction mechanisms.

The optimized geometry for each complex was obtained by a classical annealing simulation with the Forcite Plus code.^300^ The optimized structures show that dsDNA can interact with the CNPs through π-stacking and so-called “T-shaped” structures.^532^ GOQD, C_60_, and DWNT bind at the dsDNA minor groove, while GQD, C_70_, and SWNT interact at the hydrophobic ends. An analysis of interaction energies indicated that van der Waals forces play a major role in the molecular mechanism of complexes with C_60_, C_70_, and SWNT, while electrostatic interactions make a significant contribution in DWNT, GQD, and GOQD-containing complexes. Moreover, full atomistic MD simulations were performed for CNP aggregates in the presence of dsDNA fragments in aqueous media to study the dispersion process. The effect of dsDNA on the dispersion of the CNPs in water was evaluated by computing self-diffusion coefficients: the dispersion of the fullerenes and carbon nanotubes were shown to be enhanced in the presence of dsDNA, while those of GQDs and GOCDs were slightly reduced.

Ghadari^31^ studied the interactions of nitrogen-doped graphene (NG) with different biological molecules, such as nucleobases, nucleotides, and their derived triphosphates (collectively referred to as the “ligands”) by means of a mixed theoretical approach. TDDFT was employed to study the interactions at the atomistic level, including the implicit effect of the surrounding solvent through the PCM model. The explicit interaction with the solvent was accounted for by QM/MM calculations; the atoms in molecules (AIM) approach was used to identify specific interaction. Binding energies of the NG-ligand systems were evaluated by means of the MM Poisson–Boltzmann surface area (MMPBSA) approach. Finally, QM/MM studies were carried out at different theory levels to predict geometries and electronic features of the ligands and to evaluate the effect of NG and explicit solvent molecules on the electronic structure of the ligands.

Large single NG sheet (∼500 C atoms) models were prepared, in which 30 C atoms were replaced by N atoms in graphitic, pyrrolic, and pyridinic doping configurations. The mixing of different theoretical approaches allowed for the evaluation of multiple interesting aspects of the interactions between the NGs and the studied biological molecules. Docking results showed an increasing tendency of ligands to interact with the NGs on going from nucleobases to anionic species. Starting from the most stable NG-ligand configurations found in the docking study, classical AA MD simulations provided binding energies while accounting for explicit solvent interactions. The MD simulations showed lesser affinity of anionic species toward NGs in comparison with the neutral triphosphates, the anionic species being more strongly solvated. The interaction of the studied ligands with both graphene and NGs was found to produce a red-shift of the HOMO-LOMO features compared to those of the corresponding isolated solvated ligands. Binding energy decomposition and AIM analysis suggest that the interactions between the studied ligands and NGs are governed by van der Waals interactions.

**Figure 60:**
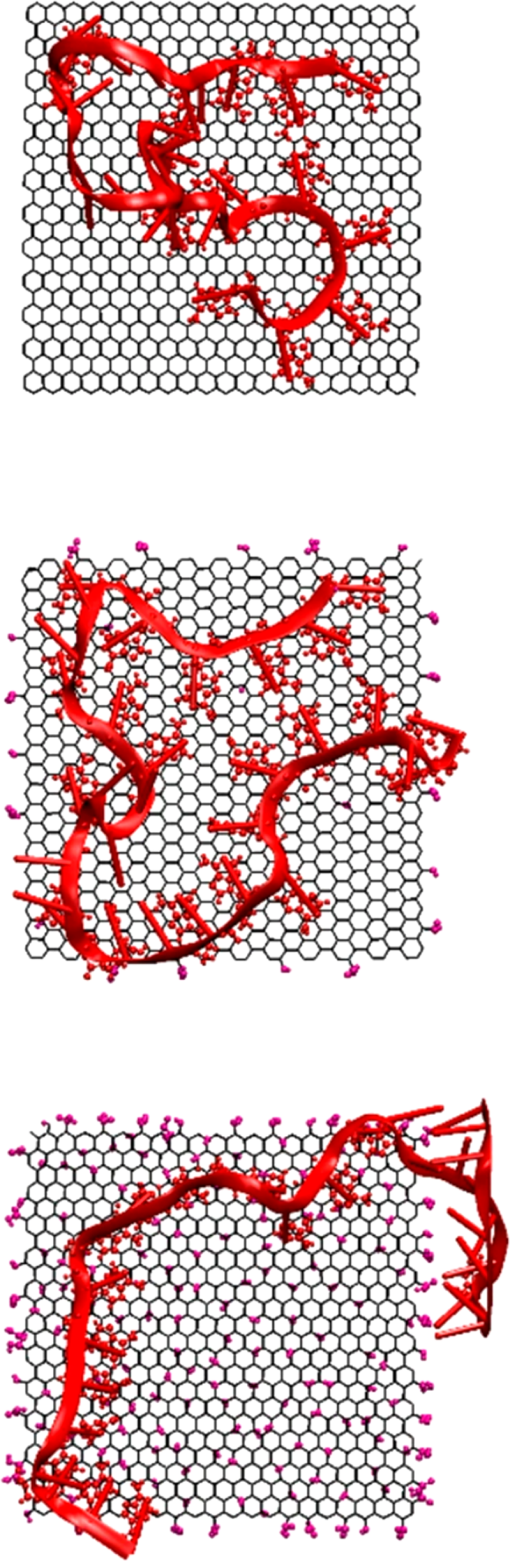
Representative adsorption states (after 100 ns MD) of ssDNA on GQDs of varying oxidation degree: pristine (0% oxidized) (top); middle, 2% oxidized (middle); 17% oxidized (bottom). Adapted with permission from Jeong et al.^298^ Copyright 2020 Springer Nature.

The adsorption of single-strand DNA (ssDNA) on pristine and partially oxidized GQDs was investigated by Jeong et al.^298^ GQDs were modeled as effectively square graphene sheets (area ∼25 nm^2^), based on experimental XPS and AFM characterization, with hydroxyl, carbonyl, and carboxyl groups randomly introduced at both edge and surface sites in order to represent different degrees of GQD oxidation. Specifically, three GQD oxidation levels were considered, computed as the ratio between oxidized and sp^2^ C atoms: 0, 2.28 and 17.36%. The adsorption of a poly adenine (A_30_) ssDNA fragment on the different GQDs was investigated during 100 ns-long classical atomistic MD simulations. Experimentally, ssDNA adsorption, monitored by the quenching of GQD fluorescence, was found to be dependent on the GQD oxidation degree, with increasing oxidation resulting in weaker adsorption. The MD simulations supported this finding and revealed ssDNA adsorption on the GQDs to be primarily driven by van der Waals interactions, with H-bonding contributing marginally to the case of partially oxidized GQDs. The closest, most stable ssDNA-GQD adsorbed structures were found for the pristine GQD, followed by the 2% oxidized GQD; the 17% oxidized GQD only showed transient contacts with the ssDNA fragment after 70 ns simulation time (Figure 60). The effect of ssDNA nucleotide sequence on GQD adsorption was investigated by similar simulations performed for poly cytosine (C_30_) and poly thymine (T_30_) ssDNA, though considering only pristine and 2% oxidized GQDs. While T_30_ showed GQD adsorption dynamics similar to that of A_30_, C_30_ was found to adsorb more weakly. In fact, C_30_ did not show any attractive interactions with the 2% oxidized GQD, consistent with the experimental finding that C_30_ does not quench the fluorescence of low-oxidation GQDs.

Xu et al.^295^ studied the genotoxicity of GQDs through a combined experimental and theoretical approach. The authors exposed rat alveolar macrophages to amine-modified GQDs (referred to as “AG-QDs” by the authors, hereafter “A-GQDs” in the interest of consistency), monitoring their nuclear internalization experimentally by confocal laser scanning microscopy (CLSM). The A-GQDs were structurally characterized by XPS, TEM, and AFM, allowing the authors to propose 10 small model A-GQD structures with a common core structure consisting of five fused benzene rings, all having a single amino-, hydroxyl-, carbonyl-, and carboxyl-group at edge sites yet at different relative positions. The specific interaction of the A-GQDs with DNA was studied by AFM and molecular docking simulations, with DNA starting coordinates taken from a crystal structure. Of the 10 A-GQDs simulated, 6 were found to form various H-bonds with DNA, while π–π stacking interactions with DNA bases were observed for all A-GQDs studied. It was proposed that this H-bonding and π–π stacking with A-GQDs could disrupt the DNA double helix structure, resulting in the experimentally observed DNA cleavage and cross-linking. The authors demonstrated that exposure to A-GQDs could lead to DNA damage, which is highly relevant to the potential biomedical applications of GQDs, which includes bioimaging, drug and gene delivery, and biosensors.

**Figure 61:**
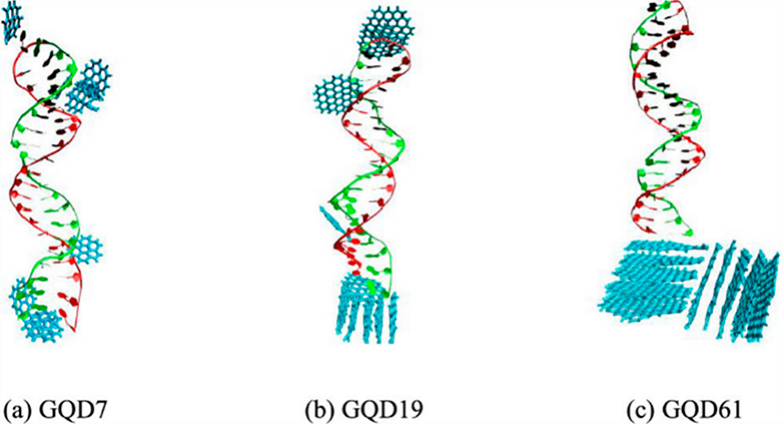
MD simulation configurations showing the adsorption on poly(A-T)_20_ for different size GQDs at simulation time 100 ns: (a) seven-fused benzene rings, GQD7, (b) GQD19, and (c) GQD61. Nucleotides A and T are shown in, respectively, green and red. Reproduced with permission from Kong et al.^302^ Copyright 2020 American Chemical Society.

Kong et al.^302^ recently reported a computational study aimed at identifying the adsorption modes and sites of pristine GQDs on DNA to help understand their role in DNA damage. The authors performed MD simulations of pristine circular GQDs (three different sizes were considered, consisting of 7, 19, and 61 fused benzene rings, see Figure 61a–c), using previously described potential parameters^280,533^ and a DNA fragment, poly(A-T)_20_. The simulations allowed for the identification of three major GQD adsorption sites on the DNA fragment: the DNA interior, i.e., between two strands (site A), the DNA fragment terminal (site B), and on the double helix groove (site C). The smallest GQDs studied (seven fused benzene rings) were found to adsorb at all three sites, with insertion in the DNA interior (parallel to base planes) disrupting H-bonds between A-T base pairs; nevertheless, the terminal site (B) was found to be favored by this GQD, allowing for effective stacking interactions with the exposed base surfaces. With increasing GQD size, terminal adsorption (site B) was increasingly favored, with GQD aggregation (stacking) also becoming more prominent (Figure 61).

The effect of adsorption of a large GQD (61 fused rings, GQD61) on the structure of a different DNA fragment, poly(G-C)_20_, was also considered. In contrast to the case of poly(A-T)_20_, the GQDs were found to adsorb at both the fragment terminal (site B) and the major groove (site C); this latter adsorption mode was shown to result in significant bending of the poly(G-C)_20_ fragment. The stronger interactions between the GQD and C and G bases, as opposed to A and T, was supported by DFT binding energy calculations. While higher GQD concentrations were found to increasingly affect the poly(A-T)_20_ structure, the authors concluded that GQD genotoxicity should be relatively low.

#### 3.6.5 Carbohydrates

A few computational studies aimed at understanding the interactions of graphene layers with carbohydrates for application purposes have been reported. Wu and co-workers^534^ prepared β-cyclodextrin (β-CD) and cellulose silica composites functionalized with GQDs as chiral stationary phases (CSP) for high-performance liquid chromatography (HPLC) to study the effect of GQDs on chiral separation. GQDs present structural features which makes them suitable not only for general chromatographic purposes but also for their application in chiral enhancement materials. To this end, GQD functionalization was first performed on β-CD and cellulose silica composites, and their enantioseparation performance was evaluated in comparison with the corresponding unmodified (i.e., non-GQD-functionalized) CSPs. GQD functionalization was found to enhance the enantioseparation behavior of the chosen CSPs with all tested racemic mixtures, unlike the corresponding CSPs functionalized with the established enantioselectivity enhancer 3,5-dimethylphenyl isocyanate. A molecular docking study was performed to investigate the mechanism by which GQD functionalization enhances chiral separation; β-CD models with and without GQD functionalization were prepared according to previously described methods.^535,536^ The resulting β-CD and GQD/β-CD geometries were optimized by MD simulations and subsequently used in molecular docking calculations. Enantiomers were first docked to β-CD and then to the GQD/β-CD complex. The interaction energies between the studied enantiomers and CSP were greater in the presence of GQDs functionalization, indicating the enhancement of the affinity between the chiral species and the selector system. Furthermore, the presence of GQDs was found to increase the interaction energy difference between enantiomers, improving the discrimination properties of the CSP. The enantioseparation of 10 different species were investigated: benzoin, benzoin methyl ether, benzoin ethyl ether, 6,6′-dibromo-1,1′-bi-2-naphthol, *trans*-stilbene oxide, flavanone, 6-hydroxyflavanone, naphthylethanol, diclofop, and metalaxyl.

**Figure 62:**
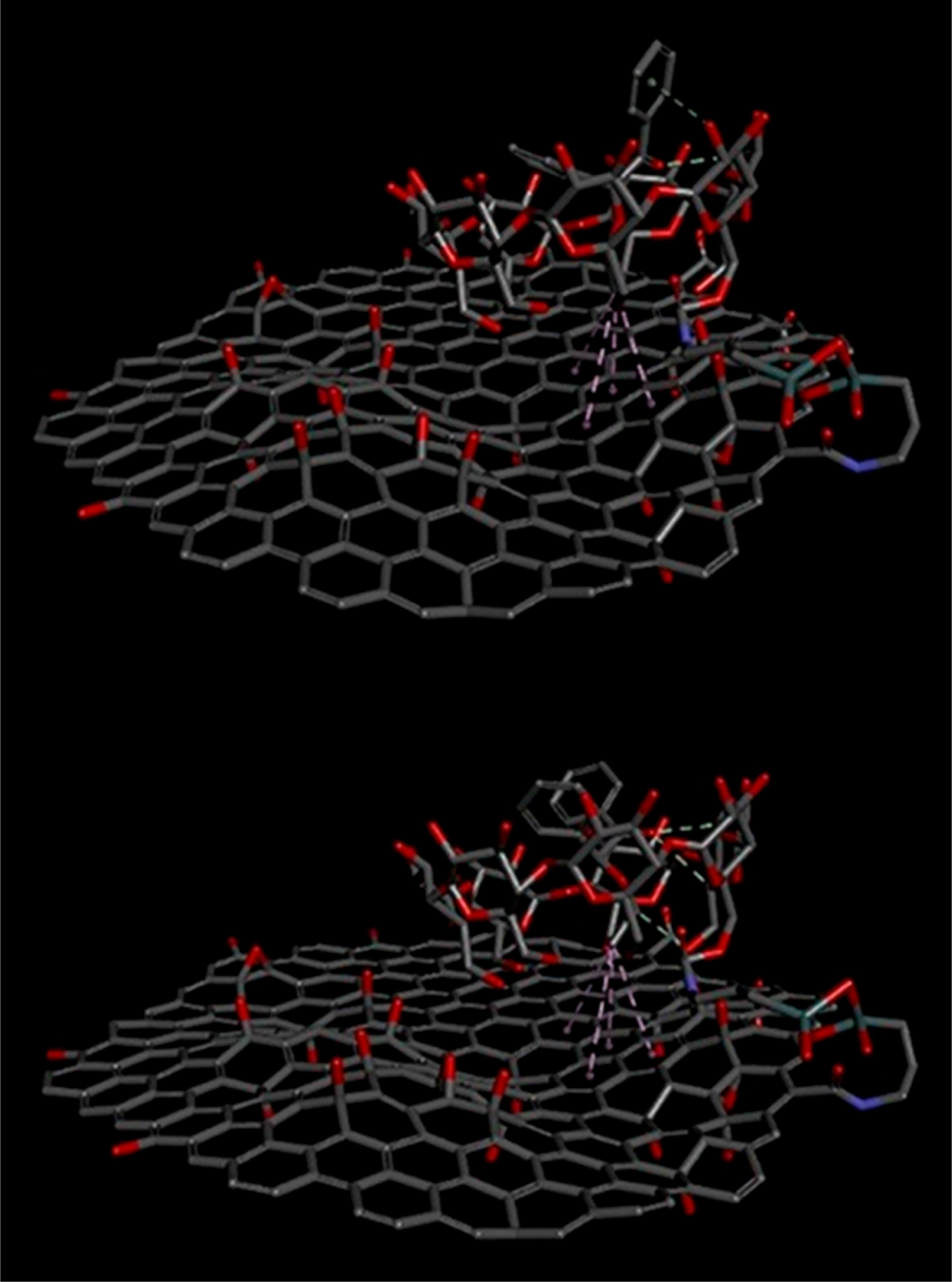
Molecular docking configurations for benzoin ethyl ether enantiomers and a model GQD/β-CD complex: (top) R- and (bottom) S-enantiomer. Prominent interactions are indicated by dashed lines. Adapted with permission from Wu et al.^534^ Copyright 2019 Elsevier B.V.

The molecular docking configurations for benzoin ethyl ether enantiorecognition on both CSPs considered are shown in Figure 62a–d. Based on these simulations, the authors state that “it was demonstrated that the existence of GQDs provides interactions with enantiomers during the inclusion process and changes the types of interactions between enantiomers and β-CD, which helps to discriminate the enantiomers and improves the enantioseparation performance”.^534^

#### 3.6.6 Photosensitization

**Figure 63:**
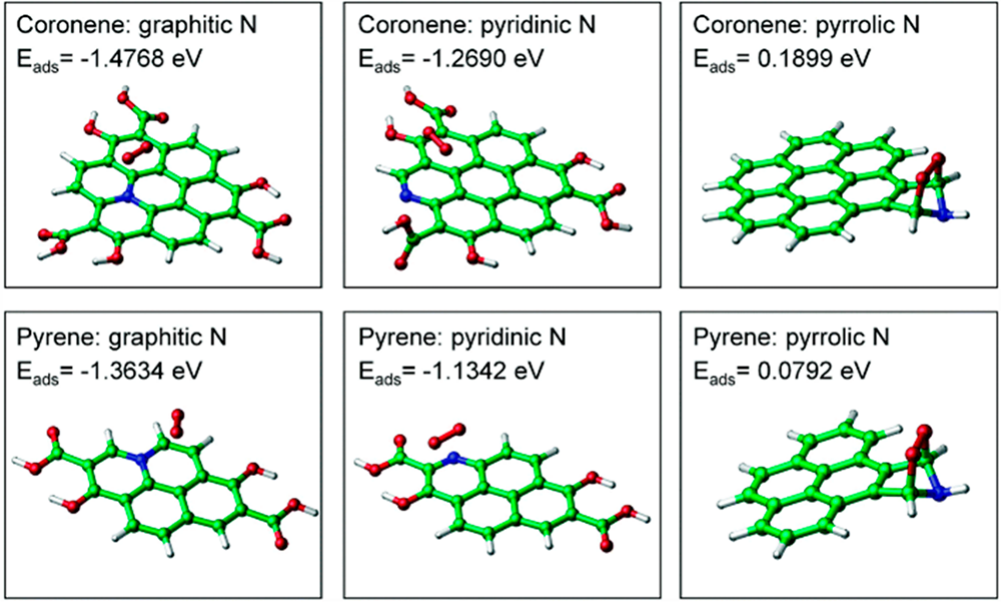
Models used by Wu et al.^43^ to rationalize the photosensitization capability of GQDs. B3LYP/6-311++G(d,p) is used to compute the displayed properties. Reproduced with permission from ref (43). Copyright 2020 Royal Society of Chemistry.

Ge et al.^537^ showed that GQDs can be efficiently used as photodynamic therapy (PDT) agents, with great potential due to their photosensitization properties and cancer imaging capability. Photosensitization allows the conversion of molecular oxygen into highly reactive oxygen species (ROS) that can be used in several photodynamic applications. The main aspects concerning CD-based photosensitizers are triplet state activation and the interaction with oxygen. Wu et al.^43^ investigated the oxygen photosensitization properties of CDs focusing on the role of nitrogen doping. N-doped CDs were hydrothermally synthesized varying the content of nitrogen and structural and optical characterization shows that the surface content of graphitic and pyrrolic nitrogen are related to photosensitization properties. Further computational investigations were performed to calculate oxygen adsorption energies and the values of the energy difference between the excited singlet state and the excited triple state (Δ*E*_ST_) for different nitrogen doping types (Figure 63). Theoretically, small values of Δ*E*_ST_ facilitate the triple state activation because of the boost of intersystem crossing (ISC) effect. DFT/TDDFT simulations were implemented in the B3LYP-6-31++G(d,p) framework using Gaussian 09-D.01.^145^ Moreover DFT-D3 dispersion correction was introduced for oxygen adsorption simulation. Coronene and pyrene-based structures modeled N-CDs as proposed by Rogach, Otyepka, and co-workers^41,42^ representing graphitic nitrogen in the center and pyridinic and pyrrolic nitrogen at the edges. On one hand, oxygen adsorption simulations determined that the nitrogen species whose structure presents the highest adsorption energy of oxygen was the pyrrolic nitrogen, followed by pyridinic and graphitic. On the other hand, the analysis reveals that graphitic nitrogen in N-CDs possessed the smallest Δ*E*_ST_.

#### 3.6.7 Inorganic Nanocomposites

Interaction between CDs and inorganic surfaces may represent an emerging area of investigation within the framework of the preparation of application-oriented CD-based devices. Composites where CDs are dispersed within a solid inorganic substrate could enable the production of functional devices by overcoming issues such as limited thermal and chemical stability, nonradiative relaxation, and self-quenching, which can be associated with CDs dispersed in liquids or to unsupported CDs powders.

Liu et al.^538,539^ presented a successful example of CD-based composites in which crystalline microporous zeolites were used as inorganic matrixes. The authors demonstrate that through solvothermal/hydrothermal routes, composites which benefit from the advantageous features of the solid matrix and from nanoconfinement associated with microporosity are obtained. The modulation of the optical properties of the composite is achieved thanks to the contribution of the zeolite acting as a good oxygen barrier and locking the emissive species by inhibiting intramolecular vibrations and rotations. Other porous inorganic solids which have been studied to host CDs including mesoporous alumina^540^ and silica, which can be prepared with different features and porosities by exploiting the flexibility of sol–gel techniques.^541,542^

Tuning the architecture and surface of the matrix is expected to open the way to the design of CD-based composites with different properties. Layered solids such as magnesium hydroxide^543^ and layered double hydroxides^289^ (LDH) exhibit an interlayer region which can be exploited for controlled introduction and further stabilization of CDs. In addition, charge density of the layer can be adjusted to optimize the functional properties of the resulting composite. Interfacing carbonaceous materials with TiO_2_ represent a strategy to address systematic efficiency issues due to the use of the isolated oxide as photocatalysts for hydrogen fuel production such as the wide band gap and the fast recombination rate. Carbon localization on TiO_2_ influences the mechanism of carrier transport and separation, hindering or boosting the performances according to the purpose of the material.

While the support of molecular modeling to rationalize this property is highly required, the additional complexity of these systems still hampers their widespread use, and the computational studies are still limited to a very limited number of works, which are analyzed below.

**Figure 64:**
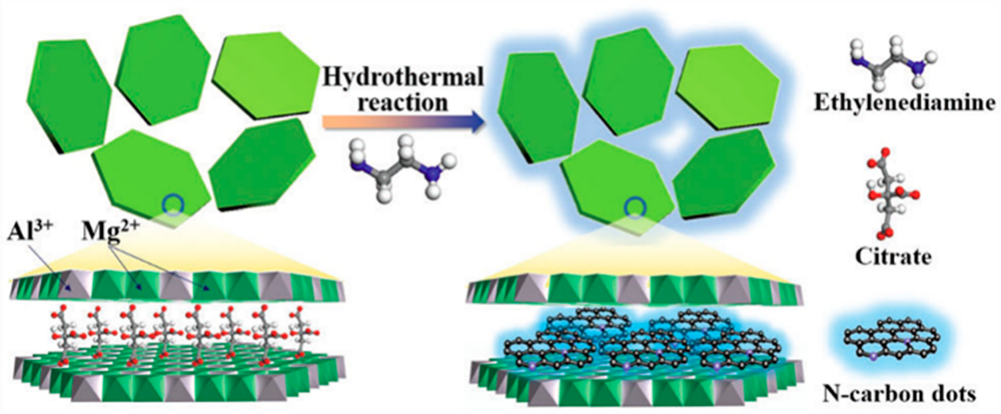
Schematic representation of the synthesis procedure used by Liu et al.^289^ to prepare the N-CNDs/LDH composite material. Reproduced with permission from ref (289). Copyright 2017 Royal Society of Chemistry.

Recently Liu et al.^289^ presented the results of an “in situ” synthesis of solid-state N-CNDs by introducing the precursors EDA and citrate between LDH layers. The aim of this novel synthesis procedure was to achieve a better CNDs dispersion and a better control of nitrogen doping level by varying the LDH layer’s charge and to enhance the overall stability of CNDs. The synthesis takes place in a two-stage process with the insertion of citrate in a LDH structure containing Mg and Al atoms (used to control the charge density) and then (formation of CND by hydrothermal reaction of LDH/citrate structures with EDA (see Figure 64).

The XRD analysis shows the formation of a periodic structure of layers with interlayer cavities with sizes compatible with the dimensions of the citrate molecules. The produced CNDs were characterized by an average diameter of 5.8 nm and were spatially arranged to form a platelet of 0.62 nm thickness, as measured from TEM and AFM images.

SEM and energy dispersive X-ray spectroscopy (EDX) morphological analysis demonstrated the formation of nanoplatelets in which Mg, Al, C, O, and N are uniformly distributed: the bright blue fluorescence (440 nm) observed at the end of the synthesis process was considered proof of formation of CNDs. The fluorescence properties of the obtained CNDs were strongly affected by the synthesis temperature (*T*): emission is excitation independent (λ_ex_ = 360 nm with *T* = 125–150 °C), while in the range 175–200 °C, an excitation-dependent behavior is observed, where fluorescence QY decreases with increasing *T*. The highest QY (26.16%) was observed at 150 °C and further enhanced to 61.63% (among the highest QY level of CND-based solid-state materials) by increasing the Mg/Al ratio from 2 to 5. Classical MD simulations helped to rationalize the enhanced N doping and QY of N-CNDs/LDH. Various model systems, consisting of layers with different Mg^2+^/Al^3+^ ratios (as experimentally determined in the prepared samples) and citrate molecules were modeled using the LDHFF3 force-field^287^ in the isothermal–isobaric ensemble at a temperature 298 K and pressure *P* = 0.1 MPa. The simulations show that an increase in the metal ratio (see above), which is related also to a decrease in the layer charge density, leads to a larger separation between the citrate molecules, thus leaving larger space for the N-containing EDA molecules to diffuse. This is as confirmed by the diffusion coefficient calculated when explicitly including the EDA molecule in the simulation. The highlighted relationship explains the variation in the N-doping amount and the enhanced PL QY of N-CNDs/LDH.

Long et al.^86^ theoretically investigated the interactions of GQDs with other metal oxides, i.e., TiO_2_. The aim of the work was to analyze the interfacial pyrene, coronene, and GQDs with the TiO_2_ electron transfer (ET) for solar energy conversion.

**Figure 65:**
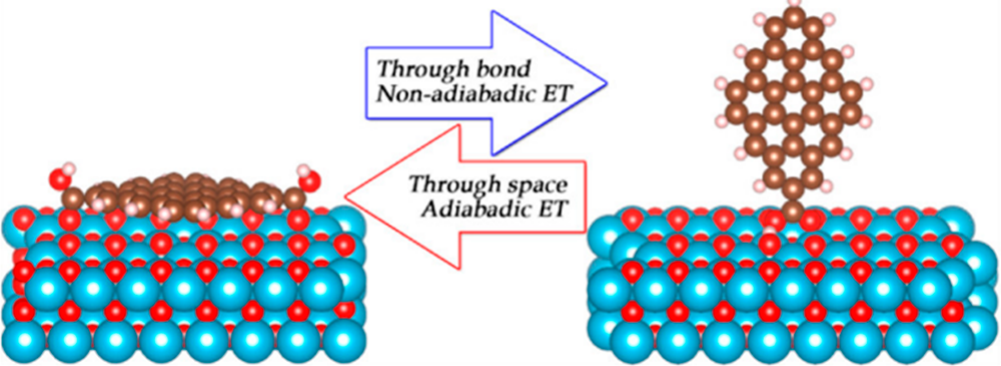
Representation of the two configurations (flat and vertical) used to study the ET and energy transfer with TiO_2_. Reproduced with permission from ref (86). Copyright 2017 American Chemical Society.

Photoinduced ET was computationally analyzed by considering a molecule/GQD model covalently linked to a TiO_2_ surface with carboxylic acid linkers fixed in flat and vertical configurations (see Figure 65). A combined approach of adiabatic and nonadiabatic MD (NAMD) and TDDFT allowed the study of chemical, geometric, and electronic properties of the mentioned composites; the obtained data were compared to the results of pump–probe experiments, showing excellent overall agreement. After relaxing the geometry at 0 K, uniform velocity rescaling was used to bring the temperature of the system up to 300 K. Then, picosecond-length adiabatic MD simulations were carried out accounting for the propagation of the photoexcited electron density. To account for the ET from the molecules and GQD to the TiO_2_ surface, the photoexcited electron density was integrated over the region occupied by the molecules and GQD. The average behavior of the photoinduced ET from pyrene into the TiO_2_ surface was obtained using Fewest switches surface hopping (FSSH)-NAMD simulations by sampling 500 initial conditions from the adiabatic MD trajectory. Concerning the energy relaxation, the vibrational modes involved in the ET process were accounted for by the so-called “influence spectrum”, which is related to the fluctuations in the electronic excitation energy. The reported simulations allowed for the determination of the mechanism and time scale of the photoinduced interfacial electron and energy transfer and energy relaxation demonstrating few differences between the vertical and planar arrangements. In the vertical configurations, the relatively weak donor–acceptor coupling, attributed to the π-electron withdrawing properties of the carboxylic acid group, results in a nonadiabatic ET mechanism. Conversely, in flat structures, the observed strong donor–acceptor coupling causes adiabatic ET. Finally, it was theoretically observed that photoexcitation of the two models has a partial charge transfer character, and the ET and energy transfer are faster than energy relaxation, a critical condition for applications in photocatalysis and photovoltaics.

**Figure 66:**
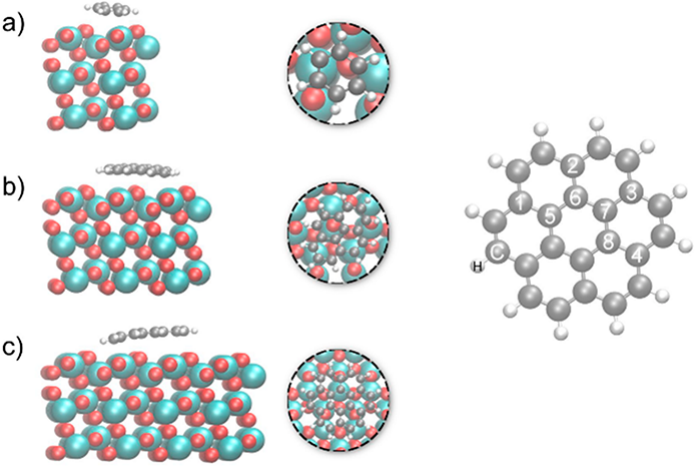
Side (left) and top (middle) views of the optimized geometries of TiO_2_–CDs heterostructures studied by Sen et al.,^544^ with the CND modeled as (a) benzene, (b) pyrene, (c) boron doped coronene. Evaluated B doping sites (right). Reproduced with permission from Otyepka and co-workers.^544^ Copyright 2019 American Chemical Society.

Otyepka and co-workers,^544^ recently studied the effects of pure and B-doped CNDs at the interface with TiO_2_. DFT calculations were performed to analyze the optical absorption range and photocatalytic water-splitting performances of the TiO_2_/CNDs heterostructures. The authors used benzene and two PAHs (pyrene and coronene) as small nondoped CND models, while B-doped CNDs were modeled as a substituted coronene (see Figure 66a–c). The band gap between the valence and the conduction bands of pristine CNDs and TiO_2_ at their interface has a staggered alignment, known as a type II heterojunction, which facilitates spontaneous spatial carrier separation and hydrogen evolution for sensitizer configuration. However, CND band gaps are too large to obtain convenient optical efficiencies. Instead, the band alignment was found to be of the straddling type (type I heterojunction), with the substitutional B-doped CNDs interfaced with TiO_2_ showing an ∼48–57% CD band gap reduction compared to their pristine CD–TiO_2_ counterpart, resulting in an improved full spectrum optical absorption. This heterojunction is suitable for hydrogen evolution but with high chances of recombination loss; nevertheless, internal electric fields at these heterojunctions were found to act as a “charge valve”, countering the recombination phenomenon by selectively permitting electrons but restricting hole migration.

In addition to interactions with metal oxides, the interactions of CNDs with specific metals have been characterized. In fact, CNDs have been proposed as potential environmentally friendly alternatives to traditional corrosion inhibitors, which typically contain heavy metals. Corrosion inhibitors protect metals by exploiting the interactions between some specific functional groups and the charge located at the metal surface.

In particular, the corrosion protectant properties of CNDs can be tuned by doping their surface functional groups with various elements. Recently, Ye et al.^45^ investigated, by means of experiments as well DFT calculations and classical MD simulations, the properties of N-CNDs as new metal corrosion inhibitors.

The N-CNDs were synthesized from CA and l-histidine and the corrosion inhibitive behavior of both the precursors and the final product was studied by considering a system formed by Q235 steel in 0.1 M HCl solution containing CA, l-histidine (l-His), and N-CNDs.

DFT-B3LYP calculations were performed, modeling the N-CND as formed by four fused heteroaromatic rings, or its precursors CA, L-Hi. The lower value of N-CND band gap compared to the other two inhibitors suggested that N-CNDs may present higher chemical reactivity with the steel surface, explaining the experimentally observed more efficient inhibitive power. Atomistic MD simulations allowed for the characterization of the interactions of CA, l-His, and N-CNDs at the interface between steel and the inhibitors. Unfortunately, no details on the force fields used were given by the authors. The atomistic simulations show that the equilibrium adsorption configurations of the N-CNDs on the iron surface are characterized by a stronger binding energy compared to those of the precursors. This further explains its superior corrosion inhibitive capability, since the corrosion inhibition performance is expected to increase with the strength of the binding.^545^

#### 3.6.8 Interactions with Polymers

Salestan et al.^546^ presented a combined MD simulations and experimental study to determine the structure of polyamide (PA) thin-film composite (TFC) membranes modified with GQDs.

PA-TFC membranes are currently widely used in desalination and forward osmosis due to their superior chemical stability as well as their high rejection of salts and other contaminants. Recently, it was demonstrated that the properties of TFC membranes can be tailored by incorporation of different types of nanomaterials. The resulting composite structures are highly complex, often preventing the rationalization of their performance. In general, a nanocomposite membrane is made by including various nanomaterials into the membrane selective layer. In this way, the interfacial regions between the PA chains and the filler affect the transport properties of the resulting membrane; from this point of view, GQDs are promising nanomaterials to be potentially included into PA due to the presence of hydroxyl- and carboxyl-functional groups on GQDs edges or surfaces that could, in principle, improve their compatibility with PA.

**Figure 67:**
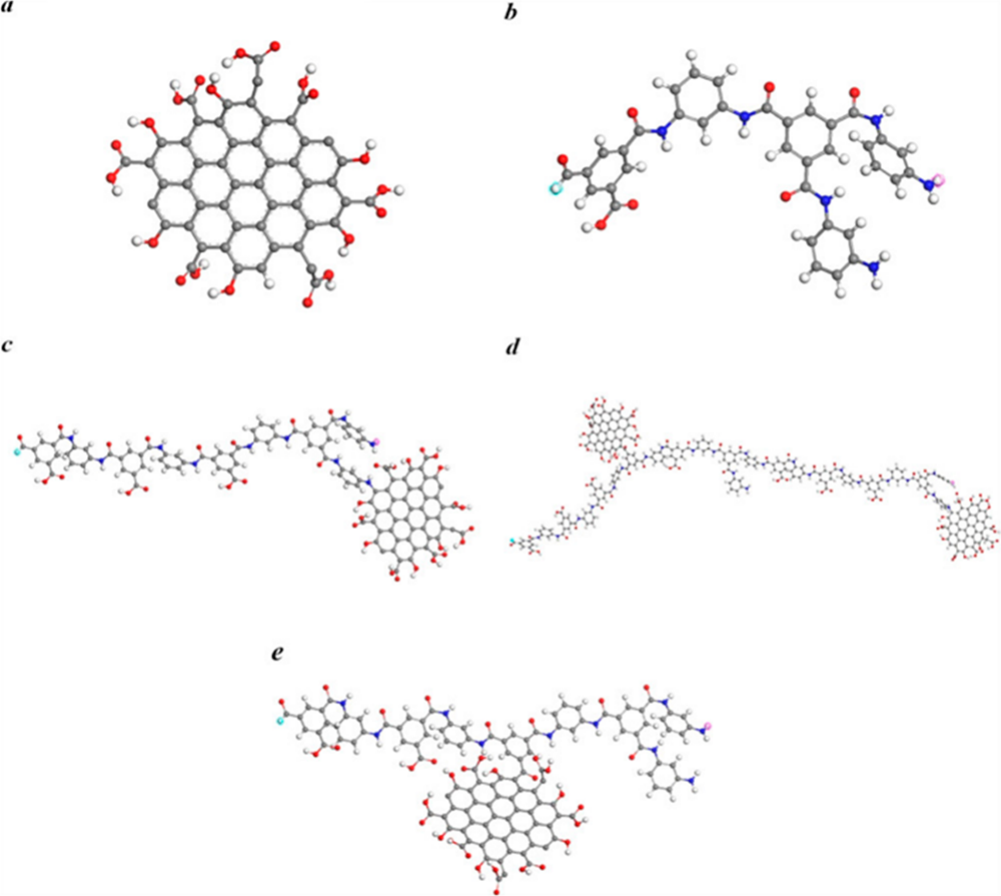
Models of (a) GQD and (b) polyamide P1. Panels c–e: polyamide-GQD systems considered by Salestan et al.^546^ Reproduced with permission from ref (546). Copyright 2020 American Chemical Society.

Classical MD simulations were performed to elucidate how the incorporation of GQDs into a PA matrix affects the molecular-level interactions and eventually analyze, at the atomistic level, the effects on the water flux behavior of the resulting composite membranes. Specifically, GQDs (Figure 67a) interacting with model polyamines (Figure 67b–e) were considered, and the corresponding energies, densities, and water diffusion coefficients means square displacement and fractional free volume were finally predicted from the simulations. A most-likely GQD-PA structure was identified from the simulations, in which the GQDs form covalent bonds with the amine-containing monomers and H-bonds with the PA chains. Moreover, both experiments and MD simulations show that in this structure, the water diffusivity is particularly high compared to the other hypothesized configurations and pristine PA. This specific characteristic has been attributed to the relatively large surface wettability resulting from the presence of GQDs.

#### 3.6.9 Overview

Computational studies of the interactions of CDs with small molecules,^33,37,43,54,60,63,75,76,509,510^ surfaces^45,86,289,544^ and biological macromolecules or assembled structures, including proteins,^275−277,297,304,526,527,529,530^ nucleic acids,^31,295,298,302^ and lipid bilayers,^63,279,307,308,281,284,285,291,293,302,305,306^ generally make use of highly simplified or idealized CD models. The former two fields (interactions with small molecules or surfaces), which often employ computationally expensive electronic structure methods, specifically, DFT/TDDFT routinely models the CDs themselves as small molecules, e.g., pyrene or coronene derivatives. Recent examples reviewed in the preceding subsections include Wu et al.’s study of the oxygen photosensitization of N-doped CDs (GQDs)^43^ and that of Otyepka and co-workers,^544^ investigating the photocatalytic water splitting performance of B-doped CNDs at TiO_2_ surfaces. Studies of CDs in biological environments, on the other hand, require the simulation of large to extremely large models, which are then necessarily described by approximate FFs or even further simplified by using CG models. These studies also consider large CD models, e.g., nanosheets,^279,291,293,298,305,306,308^ larger GQDs,^276,277,280,302,307^ or spherical multilayer CNDs^285,531^ consisting of hundreds of atoms, and attempts are frequently made to introduce experimentally detected surface or basal plane defects or O-containing functional groups. Nevertheless, the CD models considered to date have remained essentially highly simplistic, lacking amorphous disordered regions that have been found to be present in the cores of spherical CNDs (depending on a number of factors, including synthetic procedure).^11,79,451^ Recently, a spherical CND model was described in which O-containing functionalities and disordered sp^3^-hybridized regions are incorporated in the core,^531^ in order to better approximate the experimentally determined CND structure, including the sp^2^/sp^3^ hybridization ratio. Future computational studies of CDs, and spherical CNDs in particular, will likely aim to develop CD models that more accurately incorporate experimentally derived information on atomic composition (including surface and basal plane oxidation and dopant atoms) and disordered sp^3^-hybrized regions.

## 4 Perspectives

Carbon nanodots have emerged as novel carbon-based materials for prospective applications in diverse areas ranging from biomedicine to optoelectronics. Owing to their optical properties, they can be regarded as an alternative to both molecular fluorophores and inorganic quantum dots for their reduced toxicity and easier production routes. To further design and engineer CNDs toward new applications and markets, fundamental aspects such as the emission mechanisms and the CND structures need to be further elucidated, as highlighted in this review and in refs (547 and 548).

Questions still awaiting an answer include

(i) What are the structures and elements responsible for CND properties such as fluorescence and at which step of the synthesis are these formed?
(ii) How do the fluorescent species bind to the graphitic core? Why are they not affected by aggregation quenching phenomena and how do they retain their quantum efficiency, possibly providing a fluorescence tunability?
(iii) How does the surrounding solvent and pH influence CND optical properties?

Intensive experimental work has been devoted to the investigation of CNDs, and advantages and limitations of the main techniques for the investigation of carbon dots have also been discussed here (see section 2.3.1). It appears that the characterization of CNDs requires a multiscale methodology spanning individual methods surveyed in this review, and novel approaches would be desirable to achieve a comprehensive description of CNDs. Among other techniques, it is expected that application of NMR spectroscopy could provide relevant insights into the structure and formation of CNDs, although to date it has not been used much. For this reason, calculations of NMR parameters and spectra are still very limited. Experimental characterization of carbon dots so far does not enable one to fully address the questions raised above and points at some further challenges to be addressed for CND design. One is the polydispersity in size and shape often associated with synthetic protocols. Advances in controlled synthesis, purification, and separation are expected to enable a better characterization of carbon dots. Besides existing purification and separation procedures ranging from dialysis and filtration to electrophoresis and chromatography, further investigation and standardization of these protocols would be needed to enable production of well-defined CNDs.^419,431^

Reported carbon dots include a wide variety of carbon-rich nanostructures, with a variable C/H/O ratio, possible heteroatom doping, and different degrees of crystallinity ranging from amorphous to a nanocrystalline graphite-like structure. On the other hand, a unique, comprehensive, and straightforward classification involving the core composition and structure of the different CNDs which can be produced is not available and would be highly desirable as a framework for the modeling investigation and the elucidation of the synthesis-structure-properties relationship for this class of materials. Recent reviews on structure and material chemistry perspectives on carbon dots^547,548^ propose that their classification should consider ordered/disordered inner structures and the morphology of the nanoparticles, as stressed also in the present review. These are related to two critical parameters: the sp^2^/sp^3^ ratio and the 3D topology of the carbon network. To disentangle the structural pathway during a specific synthesis of CNDs is one of the most important goals. In silico studies can help to pinpoint those pathways and further define the boundaries of each category in the classification. As discussed in this review, computational studies can provide a significant contribution in elucidating the main features and properties of CNDs. In particular, in silico studies are necessary in providing the needed atomistic insight into CNDs to explain and rationalize several complex phenomena like the pH dependence of their emission,^549^ detection of room-temperature phosphorescence, or thermally activated delayed fluorescence in solid state CND hybrids,^550,551^ aggregation of nanoparticles to tune optical properties,^38,552^ or energy/charge transfer processes and nonradiative relaxation mechanisms. All these phenomena are intimately related to the structure and composition of the nanoparticles and can be used to broaden the application areas of CNDs once the structure-to-property connection is firmly established. Future improvements include detailed studies of the reactions between precursors, condensation, and cyclization mechanisms should be studied in detail. This would allow building better CND models and a better interpretation of the experimental results, including formation of fluorophores, graphitization processes, and interaction of fluorophores with possible core structures.

Identification of distinct classes of carbon-based particles may also require more tailored computational protocols for different classes of CDs. An effort to employ more realistic CND models than simple single PAHs or graphene layer is required as well as to include structural features over different scale length^452,453^ that can have an important effect on the experimentally measured electronic properties. Due to current limitations in computing power, models proposed for graphene carbon dots are often adapted to the other classes of carbon dots. However, considering the different and variable degree of crystallinity of CNDs as compared to GQDs, besides sp^2^ based C-structures also sp^3^ networking should receive more attention, including variable sp^2^/sp^3^ ratio. To better explain the properties of CNDs it is mandatory to investigate more complex systems going up and down the ladder identified in the section Strategies to Build Model Structures of this review. Presently very few reviews are available on CDs theoretical modeling, surveying the computational results on CD structure or electronic features. In this review, the role of different in silico approaches is discussed, considering the results achieved so far as well as computational time, model size, and complexity and the support from experimental characterization.

In the future, we expect that mesoscale coarse-grain (CG) models will continue to become more accurate and CG simulations will become more standard, like atomistic simulations currently. This will allow CNDs of large sizes to be studied and, importantly, to include more realistic models in the simulation of CND interactions with large biomolecular systems. To date, computational studies of such large and complex systems rely on extremely simplified CND models such as those based on graphene quantum dots. For carbon allotropes and carbon-based materials, it should be possible to have good quality transferable CG force fields allowing naturally multiscale modeling from first-principles to mesoscale and connecting to many experimental techniques operating on the mesoscale. Also, relatively easy fine-graining can be expected, as the generic structures, topologies, and morphologies of different carbon systems are well-known. This would make it possible to go back and focus on atomistic details whenever needed.

To connect with experiments beyond mesoscale, we need to continue the multiscale ladder by continuum, thermodynamical, and kinetic models. Another important computational perspective for CNDs is offered by machine learning (ML), replacing the role of empirical molecular mechanical force fields except in biomolecule–nanoparticle interactions. Future directions include the use of ML to develop interparticle potential functions from accurate *ab initio* calculations/simulations and will be operational in the analysis of the results from both computations and experiments. It is important to note that ML applications to CND studies extend beyond creating force fields based on accurate QM calculations and optimizing models: ML has many uses for materials scientists. Examples are to predict synthetic pathways or probability for material to crystallize, analyze multidimensional data from different experiments, spectroscopy, or imaging. Among the most important uses is to establish structure–property relationships to find new materials with specific properties. Further application of ML to investigations of CDs is to be expected considering that new applications of ML emerge continuously to support both in silico and experimental work and ML is becoming more available to scientists with easy-to-use tools and accessible molecular databases.^553^

Nonadiabatic MD simulations could also find increasing applications for large atomic ensembles, such as CNDs, and for statistically treated trajectories to obtain reaction coordinates, useful to estimate the mechanism of nanoparticle formation. Clearly, DFT and TDDFT are expected to retain a crucial role as reliable routine tools in evaluating spectroscopic features of CNDs and will receive a new drive from approximate DFT tight binding schemes and their updated parametrizations of different elements. DFTB can also be used as engine in MD simulations to study ground and excited state dynamics.

Thus, photoluminescence mechanisms, surface functionalization, interaction between the core and surface, and quantum confinement effects connected to π-domains in CNDs would be rationalized looking at possible multipurpose applications. A recent excellent review by Otyepka and co-workers^23^ underlines how a proper understanding of the photoluminescence of CNDs will highly benefit from multireference calculations to produce a benchmark for high computing cost systems. A remaining challenge relates to the correlation between experimental and computational description of CNDs. Although some computational models can reproduce, at least qualitatively, certain properties or trends for given types of dots, a detailed support of theoretical predictions is still in its early stages. One of the most important issues is the relatively large size of the carbon dot systems, which would require extensive computational resources. Calculations are also limited by the lack of knowledge of the synthesis processes. Future studies to be implemented are therefore further characterization of the system at the relevant steps of CND synthesis. The reactions between precursors, condensation, and cyclization mechanisms should be investigated in detail, and this would support, with less computational effort, the interpretation of experimental results such as the formation of fluorophores, the graphitization processes, and the interaction of fluorophores with possible core structures. It should be also pointed out that there is not yet a consistent and reliable method of calculating fluorescence spectra for large and complex systems such as CNDs, the estimation of which is still mostly qualitative.^23^

The modeling of the interactions of CNDs with the surrounding media (solvent, solid matrix) as well as other (bio)molecules, is an area with large margins of improvement. Indeed, as discussed in sections 2.1.10 and 3.5, solvent and pH effect on optical features of CNDs is an example of poor correspondence between simulated and experimental studies, suggesting that the role of solvent might be overlooked in current models. In particular, the explicit inclusion of solvent molecules, or of the embedding medium, around the CNDs models is currently feasible, possibly with QM/MM methods, and to be recommended for future studies, at least to test the validity of modeling in the gas phase or with implicit solvents. Finally, the mechanisms of interaction among fluorophores in aggregation processes, responsible for quenching and energy transfer and, at least in part, of the excitation dependent photoluminescence feature, would deserve a special emphasis. Overall, although a great deal of work is already available on carbon dots and the reader is referred to dedicated literature for selected aspects, the present review highlights how in silico methods are providing a valuable contribution to investigate CNDs and that further work is expected to help facing the remaining open issues and to guide the synthesis, providing basic ideas to design CND systems at the molecular level to control different properties.

In addition to the more established potential applications of CNDs as detectors, sensors, drug carriers, or active systems for catalysis, electronics, and photonics, computational studies can support additional applications of carbon dots as diverse as detection of food poisoning with histamine or to trace DNA or fingerprints in forensics. We hope that our review will motivate a closer collaboration between experimentalists and theoreticians to develop combined approaches to explain and predict the structural and optical properties of CNDs, and their interactions with other systems.

## References

[ref1] Taniguchi N. On the Basic Concept of Nano-Technology. In Proceedings of the Inetrnational Conference on Production Engineering, Tokyo, Part II, Japan Society of Precision Engineering, 1974; pp 5–10.

[ref2] Reimann S. M.; Manninen M. Electronic Structure of Quantum Dots. Rev. Mod. Phys. 2002, 74, 1283–1342. 10.1103/RevModPhys.74.1283.

[ref3] Sun Y. P.; Zhou B.; Lin Y.; Wang W.; Fernando K. A. S.; Pathak P.; Meziani M. J.; Harruff B. A.; Wang X.; Wang H.; et al. Quantum-Sized Carbon Dots for Bright and Colorful Photoluminescence. J. Am. Chem. Soc. 2006, 128, 7756–7757. 10.1021/ja062677d. 16771487

[ref4] Ekimov A. I.; Efros A. L.; Onushchenko A. A. Quantum Size Effect in Semiconductor Microcrystals. Solid State Commun. 1985, 56, 921–924. 10.1016/038-1098(85)80025-9.

[ref5] Xu X.; Ray R.; Gu Y.; Ploehn H. J.; Gearheart L.; Raker K.; Scrivens W. A. Electrophoretic Analysis and Purification of Fluorescent Single-Walled Carbon Nanotube Fragments. J. Am. Chem. Soc. 2004, 126, 12736–12737. 10.1021/ja040082h. 15469243

[ref6] Bacon M.; Bradley S. J.; Nann T. Graphene Quantum Dots. Part. Part. Syst. Charact. 2014, 31, 415–428. 10.1002/ppsc.201300252.

[ref7] Facure M. H. M.; Schneider R.; Mercante L. A.; Correa D. S. A Review on Graphene Quantum Dots and Their Nanocomposites: From Laboratory Synthesis towards Agricultural and Environmental Applications. Environ. Sci. Nano 2020, 7, 3710–3734. 10.1039/D0EN00787K.

[ref8] Tao S.; Zhu S.; Feng T.; Xia C.; Song Y.; Yang B. The Polymeric Characteristics and Photoluminescence Mechanism in Polymer Carbon Dots: A Review. Mater. Today Chem. 2017, 6, 13–25. 10.1016/j.mtchem.2017.09.001.

[ref9] Wu C.; Chiu D. T. Highly Fluorescent Semiconducting Polymer Dots for Biology and Medicine. Angew. Chemie - Int. Ed. 2013, 52, 3086–3109. 10.1002/anie.201205133. PMC5616106 23307291

[ref10] Yan F.; Sun Z.; Zhang H.; Sun X.; Jiang Y.; Bai Z. The Fluorescence Mechanism of Carbon Dots, and Methods for Tuning Their Emission Color: A Review. Microchim. Acta 2019, 186, 583 10.1007/s00604-019-3688-y. 31359150

[ref11] Mintz K. J.; Zhou Y.; Leblanc R. M. Recent Development of Carbon Quantum Dots Regarding Their Optical Properties, Photoluminescence Mechanism, and Core Structure. Nanoscale 2019, 11, 4634–4652. 10.1039/C8NR10059D. 30834912 PMC6467229

[ref12] Reckmeier C. J.; Schneider J.; Susha A. S.; Rogach A. L. Luminescent Colloidal Carbon Dots: Optical Properties and Effects of Doping [Invited]. Opt. Express 2016, 24, A312 10.1364/OE.24.00A312. 26832584

[ref13] Zhu S.; Song Y.; Zhao X.; Shao J.; Zhang J.; Yang B. The Photoluminescence Mechanism in Carbon Dots (Graphene Quantum Dots, Carbon Nanodots, and Polymer Dots): Current State and Future Perspective. Nano Res. 2015, 8, 355–381. 10.1007/s12274-014-0644-3.

[ref14] Semeniuk M.; Yi Z.; Poursorkhabi V.; Tjong J.; Jaffer S.; Lu Z.; Sain M. Future Perspectives and Review on Organic Carbon Dots in Electronic Applications. ACS Nano 2019, 13, 6224–6255. 10.1021/acsnano.9b00688. 31145587

[ref15] Yuan T.; Meng T.; He P.; Shi Y.; Li Y.; Li X.; Fan L.; Yang S. Carbon Quantum Dots: An Emerging Material for Optoelectronic Applications. J. Mater. Chem. C 2019, 7, 6820–6835. 10.1039/C9TC01730E.

[ref16] Ghosal K.; Ghosh A. Carbon Dots: The next Generation Platform for Biomedical Applications. Mater. Sci. Eng. C. Mater. Biol. Appl. 2019, 96, 887–903. 10.1016/j.msec.2018.11.060. 30606603

[ref17] Roy P.; Chen P. C.; Periasamy A. P.; Chen Y. N.; Chang H. T. Photoluminescent Carbon Nanodots: Synthesis, Physicochemical Properties and Analytical Applications. Mater. Today 2015, 18, 447–458. 10.1016/j.mattod.2015.04.005.

[ref18] Guo L.; Ge J.; Wang P. Polymer Dots as Effective Phototheranostic Agents. Photochem. Photobiol. 2018, 94, 916–934. 10.1111/php.12956. 29896881

[ref19] Choi Y.; Choi Y.; Kwon O.-H.; Kim B.-S. Carbon Dots: Bottom-Up Syntheses, Properties, and Light-Harvesting Applications. Chem. - An Asian J. 2018, 13, 586–598. 10.1002/asia.201701736. 29316309

[ref20] Yuan F.; Li S.; Fan Z.; Meng X.; Fan L.; Yang S. Shining Carbon Dots: Synthesis and Biomedical and Optoelectronic Applications. Nano Today 2016, 11, 565–586. 10.1016/j.nantod.2016.08.006.

[ref21] Chakraborty P.; Ma T.; Zahiri A. H.; Cao L.; Wang Y. Carbon-Based Materials for Thermoelectrics. Adv. Condens. Matter Phys. 2018, 2018, 3898479 10.1155/2018/3898479.

[ref22] Pykal M.; Jurečka P.; Karlický F.; Otyepka M. Modelling of Graphene Functionalization. Phys. Chem. Chem. Phys. 2016, 18, 6351–6372. 10.1039/C5CP03599F. 26323438

[ref23] Langer M.; Paloncýová M.; Medved M.; Pykal M.; Nachtigallová D.; Shi B.; Aquino A. J. A.; Lischka H.; Otyepka M. Progress and Challenges in Understanding of Photoluminescence Properties of Carbon Dots Based on Theoretical Computations. Appl. Mater. Today 2021, 22, 100924 10.1016/j.apmt.2020.100924.

[ref24] Dirac P. A. M. Quantum Mechanics of Many-Electron Systems. Proc. R. Soc. A 1929, 123, 714–733. 10.1098/rspa.1929.0094.

[ref25] Wang W.; Wang B.; Embrechts H.; Damm C.; Cadranel A.; Strauss V.; Distaso M.; Hinterberger V.; Guldi D. M.; Peukert W. Shedding Light on the Effective Fluorophore Structure of High Fluorescence Quantum Yield Carbon Nanodots. RSC Adv. 2017, 7, 24771–24780. 10.1039/C7RA04421F.

[ref26] Zhao M.; Yang F.; Xue Y.; Xiao D.; Guo Y. A Time-Dependent DFT Study of the Absorption and Fluorescence Properties of Graphene Quantum Dots. ChemPhysChem 2014, 15, 950–957. 10.1002/cphc.201301137. 24590822

[ref27] Zhu B.; Sun S.; Wang Y.; Deng S.; Qian G.; Wang M.; Hu A. Preparation of Carbon Nanodots from Single Chain Polymeric Nanoparticles and Theoretical Investigation of the Photoluminescence Mechanism. J. Mater. Chem. C 2013, 1, 580–586. 10.1039/C2TC00140C.

[ref28] Srivastava I.; Khamo J. S.; Pandit S.; Fathi P.; Huang X.; Cao A.; Haasch R. T.; Nie S.; Zhang K.; Pan D. Influence of Electron Acceptor and Electron Donor on the Photophysical Properties of Carbon Dots: A Comparative Investigation at the Bulk-State and Single-Particle Level. Adv. Funct. Mater. 2019, 29, 1902466 10.1002/adfm.201902466.

[ref29] Wang J.; Cao S.; Ding Y.; Ma F.; Lu W.; Sun M. Theoretical Investigations of Optical Origins of Fluorescent Graphene Quantum Dots. Sci. Rep. 2016, 6, 24850 10.1038/srep24850. 27094439 PMC4837401

[ref30] Lin C. K. Theoretical Study of Nitrogen-Doped Graphene Nanoflakes: Stability and Spectroscopy Depending on Dopant Types and Flake Sizes. J. Comput. Chem. 2018, 39, 1387–1397. 10.1002/jcc.25206. 29504131

[ref31] Ghadari R. Nitrogen Doped Nanographene Structures; Study on the Adsorption of Nucleobases, Nucleotides, and Their Triphosphate Derivatives Using Mixed Docking, MD, and QM/MM Approaches. J. Chem. Phys. 2017, 146, 044105 10.1063/1.4974088. 28147537

[ref32] Das R.; Dhar N.; Bandyopadhyay A.; Jana D. Size Dependent Magnetic and Optical Properties in Diamond Shaped Graphene Quantum Dots: A DFT Study. J. Phys. Chem. Solids 2016, 99, 34–42. 10.1016/j.jpcs.2016.08.004.

[ref33] Sadrolhosseini A. R.; Rashid S. A.; Jamaludin N.; Isloor A. M. Experimental and Molecular Modeling of Interaction of Carbon Quantum Dots with Glucose. Appl. Phys. A Mater. Sci. Process. 2019, 125, 529 10.1007/s00339-019-2753-z.

[ref34] Shamsipur M.; Barati A.; Taherpour A. A.; Jamshidi M. Resolving the Multiple Emission Centers in Carbon Dots: From Fluorophore Molecular States to Aromatic Domain States and Carbon-Core States. J. Phys. Chem. Lett. 2018, 9, 4189–4198. 10.1021/acs.jpclett.8b02043. 29995417

[ref35] Feng J.; Dong H.; Pang B.; Shao F.; Zhang C.; Yu L.; Dong L. Theoretical Study on the Optical and Electronic Properties of Graphene Quantum Dots Doped with Heteroatoms. Phys. Chem. Chem. Phys. 2018, 20, 15244–15252. 10.1039/C8CP01403E. 29789854

[ref36] Schumacher S. Photophysics of Graphene Quantum Dots: Insights from Electronic Structure Calculations. Phys. Rev. B 2011, 83, 081417 10.1103/PhysRevB.83.081417.

[ref37] Liang Y.; Xu L.; Tang K.; Guan Y.; Wang T.; Wang H.; Yu W. W. Nitrogen-Doped Carbon Dots Used as an “ on - off - on ” Fluorescent Sensor for Fe 3+ and Glutathione Detection. Dye. Pigment. 2020, 178, 108358 10.1016/j.dyepig.2020.108358.

[ref38] Sau A.; Bera K.; Pal U.; Maity A.; Mondal P.; Basak S.; Mukherjee A.; Satpati B.; Sen P.; Basu S. Design and Synthesis of Fluorescent Carbon-Dot Polymer and Deciphering Its Electronic Structure. J. Phys. Chem. C 2018, 122, 23799–23807. 10.1021/acs.jpcc.8b08322.

[ref39] Hola K.; Bourlinos A. B.; Kozak O.; Berka K.; Siskova K. M.; Havrdova M.; Tucek J.; Safarova K.; Otyepka M.; Giannelis E. P.; et al. Photoluminescence Effects of Graphitic Core Size and Surface Functional Groups in Carbon Dots: COO– Induced Red-Shift Emission. Carbon N. Y. 2014, 70, 279–286. 10.1016/j.carbon.2014.01.008.

[ref40] Bayoumy A. M.; Refaat A.; Yahia I. S.; Zahran H. Y.; Elhaes H.; Ibrahim M. A.; Shkir M. Functionalization of Graphene Quantum Dots (GQDs) with Chitosan Biopolymer for Biophysical Applications. Opt. Quantum Electron. 2020, 52, 16 10.1007/s11082-019-2134-z.

[ref41] Sarkar S.; Sudolská M.; Dubecký M.; Reckmeier C. J.; Rogach A. L.; Zbořil R.; Otyepka M. Graphitic Nitrogen Doping in Carbon Dots Causes Red-Shifted Absorption. J. Phys. Chem. C 2016, 120, 1303–1308. 10.1021/acs.jpcc.5b10186.

[ref42] Holá K.; Sudolská M.; Kalytchuk S.; Nachtigallová D.; Rogach A. L.; Otyepka M.; Zbořil R. Graphitic Nitrogen Triggers Red Fluorescence in Carbon Dots. ACS Nano 2017, 11, 12402–12410. 10.1021/acsnano.7b06399. 29136460

[ref43] Wu S.; Zhou R.; Chen H.; Zhang J.; Wu P. Highly Efficient Oxygen Photosensitization of Carbon Dots: The Role of Nitrogen Doping. Nanoscale 2020, 12, 5543–5553. 10.1039/C9NR10986B. 32091517

[ref44] Yuan F.; Yuan T.; Sui L.; Wang Z.; Xi Z.; Li Y.; Li X.; Fan L.; Tan Z.; Chen A.; et al. Engineering Triangular Carbon Quantum Dots with Unprecedented Narrow Bandwidth Emission for Multicolored LEDs. Nat. Commun. 2018, 9, 2249 10.1038/s41467-018-04635-5. 29884873 PMC5993800

[ref45] Ye Y.; Zhang D.; Zou Y.; Zhao H.; Chen H. A Feasible Method to Improve the Protection Ability of Metal by Functionalized Carbon Dots as Environment-Friendly Corrosion Inhibitor. J. Clean. Prod. 2020, 264, 121682 10.1016/j.jclepro.2020.121682.

[ref46] Nandy A.; Kumar A.; Dwivedi S.; Pal S. K.; Panda D. Connecting the Dots of Carbon Nanodots: Excitation (In)Dependency and White-Light Emission in One-Step. J. Phys. Chem. C 2019, 123, 20502–20511. 10.1021/acs.jpcc.9b02428.

[ref47] Mura S.; Stagi L.; Malfatti L.; Carbonaro C. M.; Ludmerczki R.; Innocenzi P. Modulating the Optical Properties of Citrazinic Acid through the Monomer-to-Dimer Transformation. J. Phys. Chem. A 2020, 124, 197–203. 10.1021/acs.jpca.9b10884. 31829593

[ref48] Sudolská M.; Dubecký M.; Sarkar S.; Reckmeier C. J.; Zbořil R.; Rogach A. L.; Otyepka M. Nature of Absorption Bands in Oxygen-Functionalized Graphitic Carbon Dots. J. Phys. Chem. C 2015, 119, 13369–13373. 10.1021/acs.jpcc.5b04080.

[ref49] Šapić I. M.; Bistričić L.; Volovšek V.; Dananić V.; Furić K. DFT Study of Molecular Structure and Vibrations of 3-Glycidoxypropyltrimethoxysilane. Spectrochim. Acta Part A Mol. Biomol. Spectrosc. 2009, 72, 833–840. 10.1016/j.saa.2008.11.032. 19144563

[ref50] Mocci F.; Olla C.; Cappai A.; Corpino R.; Ricci P. C.; Chiriu D.; Salis M.; Carbonaro C. M. Formation of Citrazinic Acid Ions and Their Contribution to Optical and Magnetic Features of Carbon Nanodots: A Combined Experimental and Computational Approach. Materials (Basel). 2021, 14, 770 10.3390/ma14040770. 33562081 PMC7914458

[ref51] Cappai A.; Melis C.; Stagi L.; Ricci P. C.; Mocci F.; Carbonaro C. M. Insight into the Molecular Model in Carbon Dots through Experimental and Theoretical Analysis of Citrazinic Acid in Aqueous Solution. J. Phys. Chem. C 2021, 125, 4836–4845. 10.1021/acs.jpcc.0c10916.

[ref52] Kundelev E. V.; Tepliakov N. V.; Leonov M. Y.; Maslov V. G.; Baranov A. V.; Fedorov A. V.; Rukhlenko I. D.; Rogach A. L. Amino Functionalization of Carbon Dots Leads to Red Emission Enhancement. J. Phys. Chem. Lett. 2019, 10, 5111–5116. 10.1021/acs.jpclett.9b01724. 31393732

[ref53] Kundelev E. V.; Tepliakov N. V.; Leonov M. Y.; Maslov V. G.; Baranov A. V.; Fedorov A. V.; Rukhlenko I. D.; Rogach A. L. Toward Bright Red-Emissive Carbon Dots through Controlling Interaction among Surface Emission Centers. J. Phys. Chem. Lett. 2020, 11, 8121–8127. 10.1021/acs.jpclett.0c02373. 32893642

[ref54] Singh A.; Eftekhari E.; Scott J.; Kaur J.; Yambem S.; Leusch F.; Wellings R.; Gould T.; Ostrikov K.; Sonar P.; et al. Carbon Dots Derived from Human Hair for Ppb Level Chloroform Sensing in Water. Sustain. Mater. Technol. 2020, 25, e00159 10.1016/j.susmat.2020.e00159.

[ref55] Siddique F.; Langer M.; Paloncýová M.; Medved’ M.; Otyepka M.; Nachtigallová D.; Lischka H.; Aquino A. J. A. Conformational Behavior and Optical Properties of a Fluorophore Dimer as a Model of Luminescent Centers in Carbon Dots. J. Phys. Chem. C 2020, 124, 14327–14337. 10.1021/acs.jpcc.0c02175.

[ref56] Algarra M.; Moreno V.; Lázaro-Martínez J. M.; Rodríguez-Castellón E.; Soto J.; Morales J.; Benítez A. Insights into the Formation of N Doped 3D-Graphene Quantum Dots. Spectroscopic and Computational Approach. J. Colloid Interface Sci. 2020, 561, 678–686. 10.1016/j.jcis.2019.11.044. 31761465

[ref57] Xu Q.; Liu Y.; Gao C.; Wei J.; Zhou H.; Chen Y.; Dong C.; Sreeprasad T. S.; Li N.; Xia Z. Synthesis, Mechanistic Investigation, and Application of Photoluminescent Sulfur and Nitrogen Co-Doped Carbon Dots. J. Mater. Chem. C 2015, 3, 9885–9893. 10.1039/C5TC01912E.

[ref58] Yuan F.; Wang Y. K.; Sharma G.; Dong Y.; Zheng X.; Li P.; Johnston A.; Bappi G.; Fan J. Z.; Kung H.; et al. Bright High-Colour-Purity Deep-Blue Carbon Dot Light-Emitting Diodes via Efficient Edge Amination. Nat. Photonics 2020, 14, 171–176. 10.1038/s41566-019-0557-5.

[ref59] Lazar P.; Mach R.; Otyepka M. Spectroscopic Fingerprints of Graphitic, Pyrrolic, Pyridinic, and Chemisorbed Nitrogen in N-Doped Graphene. J. Phys. Chem. C 2019, 123, 10695–10702. 10.1021/acs.jpcc.9b02163.

[ref60] Ambrusi R. E.; Arroyave J. M.; Centurión M. E.; Di Nezio M. S.; Pistonesi M. F.; Juan A.; Pronsato M. E. Density Functional Theory Model for Carbon Dot Surfaces and Their Interaction with Silver Nanoparticles. Phys. E Low-Dimensional Syst. Nanostructures 2019, 114, 113640 10.1016/j.physe.2019.113640.

[ref61] Sen D.; Błoński P.; Otyepka M. Band-Edge Engineering at the Carbon Dot – TiO 2 Interface by Substitutional Boron Doping. J. Phys. Chem. C 2019, 123, 5980–5988. 10.1021/acs.jpcc.8b11554.

[ref62] Yashwanth H. J.; Rondiya S. R.; Dzade N. Y.; Dhole S. D.; Phase D. M.; Hareesh K. Enhanced Photocatalytic Activity of N, P, Co-Doped Carbon Quantum Dots: An Insight from Experimental and Computational Approach. Vacuum 2020, 180, 109589 10.1016/j.vacuum.2020.109589.

[ref63] Vatanparast M.; Shariatinia Z. Revealing the Role of Different Nitrogen Functionalities in the Drug Delivery Performance of Graphene Quantum Dots: A Combined Density Functional Theory and Molecular Dynamics Approach. J. Mater. Chem. B 2019, 7, 6156–6171. 10.1039/C9TB00971J. 31559403

[ref64] Choi J.; Kim N.; Oh J. W.; Kim F. S. Bandgap Engineering of Nanosized Carbon Dots through Electron-Accepting Functionalization. J. Ind. Eng. Chem. 2018, 65, 104–111. 10.1016/j.jiec.2018.04.018.

[ref65] Jin S. H.; Kim D. H.; Jun G. H.; Hong S. H.; Jeon S. Tuning the Photoluminescence of Graphene Quantum Dots through the Charge Transfer Effect of Functional Groups. ACS Nano 2013, 7, 1239–1245. 10.1021/nn304675g. 23272894

[ref66] Jana J.; Ganguly M.; Chandrakumar K. R. S.; Mohan Rao G.; Pal T. Boron Precursor-Dependent Evolution of Differently Emitting Carbon Dots. Langmuir 2017, 33, 573–584. 10.1021/acs.langmuir.6b04100. 28024393

[ref67] Strauss V.; Margraf J. T.; Dolle C.; Butz B.; Nacken T. J.; Walter J.; Bauer W.; Peukert W.; Spiecker E.; Clark T.; et al. Carbon Nanodots: Toward a Comprehensive Understanding of Their Photoluminescence. J. Am. Chem. Soc. 2014, 136, 17308–17316. 10.1021/ja510183c. 25372278

[ref68] Li Y.; Shu H.; Niu X.; Wang J. Electronic and Optical Properties of Edge-Functionalized Graphene Quantum Dots and the Underlying Mechanism. J. Phys. Chem. C 2015, 119, 24950–24957. 10.1021/acs.jpcc.5b05935.

[ref69] Vallan L.; Urriolabeitia E. P.; Ruipérez F.; Matxain J. M.; Canton-Vitoria R.; Tagmatarchis N.; Benito A. M.; Maser W. K. Supramolecular-Enhanced Charge Transfer within Entangled Polyamide Chains as the Origin of the Universal Blue Fluorescence of Polymer Carbon Dots. J. Am. Chem. Soc. 2018, 140, 12862–12869. 10.1021/jacs.8b06051. 30211547

[ref70] Chronopoulos D. D.; Medved M.; Potsi G.; Tomanec O.; Scheibe M.; Otyepka M. Tunable One-Step Double Functionalization of Graphene Based on Fluorographene Chemistry. Chem. Commun. 2020, 56, 1936–1939. 10.1039/C9CC09514D. 32002534

[ref71] Chen S.; Ullah N.; Zhang R. Exciton Self-Trapping in Sp2 Carbon Nanostructures Induced by Edge Ether Groups. J. Phys. Chem. Lett. 2018, 9, 4857–4864. 10.1021/acs.jpclett.8b01972. 30085672

[ref72] Sudolská M.; Otyepka M. Exact Roles of Individual Chemical Forms of Nitrogen in the Photoluminescent Properties of Nitrogen-Doped Carbon Dots. Appl. Mater. Today 2017, 7, 190–200. 10.1016/j.apmt.2017.03.004.

[ref73] Langer M.; Hrivnák T.; Medved’ M.; Otyepka M. Contribution of the Molecular Fluorophore IPCA to Excitation-Independent Photoluminescence of Carbon Dots. J. Phys. Chem. C 2021, 125, 12140–12148. 10.1021/acs.jpcc.1c02243.

[ref74] Supchocksoonthorn P.; Thongsai N.; Moonmuang H.; Kladsomboon S.; Jaiyong P.; Paoprasert P. Label-Free Carbon Dots from Black Sesame Seeds for Real-Time Detection of Ammonia Vapor via Optical Electronic Nose and Density Functional Theory Calculation. Colloids Surfaces A Physicochem. Eng. Asp. 2019, 575, 118–128. 10.1016/j.colsurfa.2019.04.087.

[ref75] Thongsai N.; Jaiyong P.; Kladsomboon S.; In I.; Paoprasert P. Utilization of Carbon Dots from Jackfruit for Real-Time Sensing of Acetone Vapor and Understanding the Electronic and Interfacial Interactions Using Density Functional Theory. Appl. Surf. Sci. 2019, 487, 1233–1244. 10.1016/j.apsusc.2019.04.269.

[ref76] Prathumsuwan T.; Jaiyong P.; In I.; Paoprasert P. Label-Free Carbon Dots from Water Hyacinth Leaves as a Highly Fluorescent Probe for Selective and Sensitive Detection of Borax. Sensors Actuators, B Chem. 2019, 299, 126936 10.1016/j.snb.2019.126936.

[ref77] Sheardy A. T.; Arvapalli D. M.; Wei J. Experimental and Time-Dependent Density Functional Theory Modeling Studies on the Optical Properties of Carbon Nanodots. J. Phys. Chem. C 2020, 124, 4684–4692. 10.1021/acs.jpcc.9b10373.

[ref78] Liu C.; Bao L.; Yang M.; Zhang S.; Zhou M.; Tang B.; Wang B.; Liu Y.; Zhang Z.-L. L.; Zhang B.; et al. Surface Sensitive Photoluminescence of Carbon Nanodots: Coupling between the Carbonyl Group and π-Electron System. J. Phys. Chem. Lett. 2019, 10, 3621–3629. 10.1021/acs.jpclett.9b01339. 31199162

[ref79] Margraf J. T.; Strauss V.; Guldi D. M.; Clark T. The Electronic Structure of Amorphous Carbon Nanodots. J. Phys. Chem. B 2015, 119, 7258–7265. 10.1021/jp510620j. 25731776

[ref80] Kwon W.; Do S.; Kim J.-H.; Seok Jeong M.; Rhee S.-W. Control of Photoluminescence of Carbon Nanodots via Surface Functionalization Using Para-Substituted Anilines. Sci. Rep. 2015, 5, 12604 10.1038/srep12604. 26218869 PMC4517466

[ref81] Hjort M.; Stafström S. Modeling Vacancies in Graphite via the Hückel Method. Phys. Rev. B - Condens. Matter Mater. Phys. 2000, 61, 14089–14094. 10.1103/PhysRevB.61.14089.

[ref82] Tepliakov N. V.; Kundelev E. V.; Khavlyuk P. D.; Xiong Y.; Leonov M. Y.; Zhu W.; Baranov A. V.; Fedorov A. V.; Rogach A. L.; Rukhlenko I. D. Sp2-Sp3-Hybridized Atomic Domains Determine Optical Features of Carbon Dots. ACS Nano 2019, 13, 10737–10744. 10.1021/acsnano.9b05444. 31411860

[ref83] Shekaari A.; Abolhassani M. R. Car-Parrinello Molecular Dynamics Study of the Melting Behaviors of n -Atom (N = 6, 10) Graphene Quantum Dots. Chem. Phys. Lett. 2017, 678, 177–185. 10.1016/j.cplett.2017.04.058.

[ref84] McCulloch D. G.; McKenzie D. R.; Goringe C. M. Ab Initio Simulations of Amorphous Carbon. Phys. Rev. B - Condens. Matter Mater. Phys. 2000, 61, 2349–2355. 10.1103/PhysRevB.61.2349.

[ref85] Osella S.; Knippenberg S. Environmental Effects on the Charge Transfer Properties of Graphene Quantum Dot Based Interfaces. Int. J. Quantum. Chem. 2019, 119, e25882 10.1002/qua.25882.

[ref86] Long R.; Casanova D.; Fang W. H.; Prezhdo O. V. Donor-Acceptor Interaction Determines the Mechanism of Photoinduced Electron Injection from Graphene Quantum Dots into TiO2: π-Stacking Supersedes Covalent Bonding. J. Am. Chem. Soc. 2017, 139, 2619–2629. 10.1021/jacs.6b09598. 28125783

[ref87] Wilson E. B. Fifty Years of Quantum Chemistry. Pure Appl. Chem. 1976, 47, 41–47. 10.1351/pac197647010041.

[ref88] Hückel E. Zur Quantentheorie Der Doppelbindung. Zeitschrift für Phys. 1930, 60, 423–456. 10.1007/BF01341254.

[ref89] Kutzelnigg W. What I like about Hückel Theory. J. Comput. Chem. 2007, 28, 25–34. 10.1002/jcc.20470. 17103368

[ref90] Hoffmann R. An Extended Hückel Theory. I. Hydrocarbons. J. Chem. Phys. 1963, 39, 1397–1412. 10.1063/1.1734456.

[ref91] Wolfsberg M. A. X.; Helmholz L. The Spectra and Electronic Structure of the Tetrahedral Ions MnO 4-, CrO4-, and ClO4-. J. Chem. Phys. 1952, 20, 837–843. 10.1063/1.1700580.

[ref92] Hall M. B. Perspective on “The Spectra and Electronic Structure of the Tetrahedral Ions Mn04-, Cr04-, and Cl04-” Wolfsberg M, Helmholz L (1952) J Chem Phys 20:837 ± 843. Theor. Chem. Acc. 2000, 103, 221–224. 10.1007/978-3-662-10421-7_17.

[ref93] Pople J. A.; Beveridge D. L. Approximate Molecular Orbital Theory; McGraw-Hill: New York, 1970.

[ref94] Grabill L. P.; Berger R. F. Calibrating the Extended Hückel Method to Quantitatively Screen the Electronic Properties of Materials. Sci. Rep. 2018, 8, 10530 10.1038/s41598-018-28864-2. 30002480 PMC6043563

[ref95] Langer M.; Paloncyova M.; Medved’ M.; Otyepka M. Molecular Fluorophores Self-Organize into C-Dot Seeds and Incorporate into C-Dot Structures. J. Phys. Chem. Lett. 2020, 11, 8252–8258. 10.1021/acs.jpclett.0c01873. 32805121

[ref96] Brus L. E. A Simple Model for the Ionization Potential, Electron Affinity, and Aqueous Redox Potentials of Small Semiconductor Crystallites. J. Chem. Phys. 1983, 79, 5566–5571. 10.1063/1.445676.

[ref97] Brus L. E. Electron-Electron and Electron-Hole Interactions in Small Semiconductor Crystallites: The Size Dependence of the Lowest Excited Electronic State. J. Chem. Phys. 1984, 80, 4403–4409. 10.1063/1.447218.

[ref98] Nirmal M.; Brus L. Luminescence Photophysics in Semiconductor Nanocrystals. Acc. Chem. Res. 1999, 32, 407–414. 10.1021/ar9700320.

[ref99] Boatman E. M.; Lisensky G. C.; Nordell K. J. A Safer, Easier, Faster Synthesis for CdSe Quantum Dot Nanocrystals. J. Chem. Educ. 2005, 82, 1697–1699. 10.1021/ed082p1697.

[ref100] Rice C. V.; Giffin G. A. Quantum Dots in a Polymer Composite: A Convenient Particle-in-a-Box Laboratory Experiment. J. Chem. Educ. 2008, 85, 842 10.1021/ed085p842.

[ref101] Bauer C. A.; Hamada T. Y.; Kim H.; Johnson M. R.; Voegtle M. J.; Emrick M. S. An Integrated, Multipart Experiment: Synthesis, Characterization, and Application of CdS and CdSe Quantum Dots as Sensitizers in Solar Cells. J. Chem. Educ. 2018, 95, 1179–1186. 10.1021/acs.jchemed.7b00593.

[ref102] Onyia A. I.; Ikeri H. I.; Nwobodo A. N. Theoretical Study of the Quantum Confinement Effects on Quantum Dots Using Particle in a Box Model. J. Ovonic Res. 2018, 14, 49–54.

[ref103] Venitucci B.; Niquet Y. M. Simple Model for Electrical Hole Spin Manipulation in Semiconductor Quantum Dots: Impact of Dot Material and Orientation. Phys. Rev. B 2019, 99, 115317 10.1103/PhysRevB.99.115317.

[ref104] Jolie W.; Craes F.; Petrović M.; Atodiresei N.; Caciuc V.; Blügel S.; Kralj M.; Michely T.; Busse C. Confinement of Dirac Electrons in Graphene Quantum Dots. Phys. Rev. B 2014, 89, 155435 10.1103/PhysRevB.89.155435.

[ref105] Autschbach J. Quantum Theory for Chemical Applications: From Basic Concepts to Advanced Topics; Oxford University Press, 2020.

[ref106] Møller C.; Plesset M. S. Note on an Approximation Treatment for Many-Electron Systems. Phys. Rev. 1934, 46, 618–622. 10.1103/PhysRev.46.618.

[ref107] Cremer D. Møller-Plesset Perturbation Theory: From Small Molecule Methods to Methods for Thousands of Atoms. Wiley Interdiscip. Rev. Comput. Mol. Sci. 2011, 1, 509–530. 10.1002/wcms.58.

[ref108] Lewars E. G. Computational Chemistry Introduction to the Theory and Applications of Molecular and Quantum Mechanics; Springer Netherlands, 2011.

[ref109] Dreuw A.; Wormit M. The Algebraic Diagrammatic Construction Scheme for the Polarization Propagator for the Calculation of Excited States. Wiley Interdiscip. Rev. Comput. Mol. Sci. 2015, 5, 82–95. 10.1002/wcms.1206.

[ref110] Trofimov A. B.; Schirmer J. An Efficient Polarization Propagator Approach to Valence Electron Excitation Spectra. J. Phys. B At. Mol. Opt. Phys. 1995, 28, 2299–2324. 10.1088/0953-4075/28/12/003.

[ref111] Mester D.; Kállay M. Combined Density Functional and Algebraic-Diagrammatic Construction Approach for Accurate Excitation Energies and Transition Moments. J. Chem. Theory Comput. 2019, 15, 4440–4453. 10.1021/acs.jctc.9b00391. 31265275

[ref112] Valente D. C. A.; Do Casal M. T.; Barbatti M.; Niehaus T. A.; Aquino A. J. A.; Lischka H.; Cardozo T. M. Excitonic and Charge Transfer Interactions in Tetracene Stacked and T-Shaped Dimers. J. Chem. Phys. 2021, 154, 044306 10.1063/5.0033272. 33514084

[ref113] Liu B.; Aquino A. J. A.; Nachtigallová D.; Lischka H. Doping Capabilities of Fluorine on the UV Absorption and Emission Spectra of Pyrene-Based Graphene Quantum Dots. J. Phys. Chem. A 2020, 124, 10954–10966. 10.1021/acs.jpca.0c08694. 33325716

[ref114] Shi B.; Nachtigallová D.; Aquino A. J. A.; Machado F. B. C.; Lischka H. Emission Energies and Stokes Shifts for Single Polycyclic Aromatic Hydrocarbon Sheets in Comparison to the Effect of Excimer Formation. J. Phys. Chem. Lett. 2019, 10, 5592–5597. 10.1021/acs.jpclett.9b02214. 31479613

[ref115] Roothaan C. C. J. New Developments in Molecular Orbital Theory. Rev. Mod. Phys. 1951, 23, 69–89. 10.1103/RevModPhys.23.69.

[ref116] Margraf J. T.; Dral P. O. What Is Semiempirical Molecular Orbital Theory Approximating?. J. Mol. Model. 2019, 25, 119 10.1007/s00894-019-4005-8. 30993459

[ref117] Dewar M. J. S.; Thiel W. Ground States of Molecules. 38. The MNDO Method. Approximations and Parameters. J. Am. Chem. Soc. 1977, 99, 4899–4907. 10.1021/ja00457a004.

[ref118] Dewar M. J. S.; Zoebisch E. G.; Healy E. F.; Stewart J. J. P. Development and Use of Quantum Mechanical Molecular Models. 76. AM1: A New General Purpose Quantum Mechanical Molecular Model. J. Am. Chem. Soc. 1985, 107, 3902–3909. 10.1021/ja00299a024.

[ref119] Stewart J. J. P. Optimization of Parameters for Semiempirical Methods I. Method. J. Comput. Chem. 1989, 10, 209–220. 10.1002/jcc.540100208.

[ref120] Stewart J. J. P. Optimization of Parameters for Semiempirical Methods V: Modification of NDDO Approximations and Application to 70 Elements. J. Mol. Model. 2007, 13, 1173–1213. 10.1007/s00894-007-0233-4. 17828561 PMC2039871

[ref121] Dral P. O.; Wu X.; Spörkel L.; Koslowski A.; Weber W.; Steiger R.; Scholten M.; Thiel W. Semiempirical Quantum-Chemical Orthogonalization-Corrected Methods: Theory, Implementation, and Parameters. J. Chem. Theory Comput. 2016, 12, 1082–1096. 10.1021/acs.jctc.5b01046. 26771204 PMC4785507

[ref122] Rimola A.; Ferrero S.; Germain A.; Corno M.; Ugliengo P. Computational Surface Modelling of ICES and Minerals of Interstellar Interest—Insights and Perspectives. Minerals 2021, 11, 26 10.3390/min11010026.

[ref123] Zhang Q.; Khetan A.; Er S. Comparison of Computational Chemistry Methods for the Discovery of Quinone-Based Electroactive Compounds for Energy Storage. Sci. Rep. 2020, 10, 22149 10.1038/s41598-020-79153-w. 33335155 PMC7746720

[ref124] Mlýnský V.; Banáš P.; Šponer J.; van der Kamp M. W.; Mulholland A. J.; Otyepka M. Comparison of Ab Initio, DFT, and Semiempirical QM/MM Approaches for Description of Catalytic Mechanism of Hairpin Ribozyme. J. Chem. Theory Comput. 2014, 10, 1608–1622. 10.1021/ct401015e. 26580373

[ref125] Gavezzotti A. Crystal Formation and Stability: Physical Principles and Molecular Simulation. Cryst. Res. Technol. 2013, 48, 793–810. 10.1002/crat.201200706.

[ref126] Ercanli T.; Boyd D. B. Evaluation of Computational Chemistry Methods: Crystallographic and Cheminformatics Analysis of Aminothiazole Methoximes. J. Chem. Inf. Model. 2005, 45, 591–601. 10.1021/ci049671x. 15921449

[ref127] Rezáč J.; Fanfrlík J.; Salahub D.; Hobza P. Semiempirical Quantum Chemical PM6Method Augmented by Dispersion and H-Bonding Correction Terms Reliably Describes Various Types of Noncovalent Complexes. J. Chem. Theory Comput. 2009, 5, 1749–1760. 10.1021/ct9000922. 26610000

[ref128] Rezáč J.; Hobza P. Advanced Corrections of Hydrogen Bonding and Dispersion for Semiempirical Quantum Mechanical Methods. J. Chem. Theory Comput. 2012, 8, 141–151. 10.1021/ct200751e. 26592877

[ref129] Tu Y.; Jacobsson S. P.; Laaksonen A. Re-Examination of the NDDO Approximation and Introduction of a New Model beyond It. Mol. Phys. 2003, 101, 3009 10.1080/00268970310001619935.

[ref130] Voityuk A. A. Intermediate Neglect of Differential Overlap for Spectroscopy. Wiley Interdiscip. Rev. Comput. Mol. Sci. 2013, 3, 515–527. 10.1002/wcms.1141.

[ref131] Ridley J.; Zerner M. An Intermediate Neglect of Differential Overlap Technique for Spectroscopy: Pyrrole and the Azines. Theor. Chim. Acta 1973, 32, 111–134. 10.1007/BF00528484.

[ref132] Dral P. O.; Clark T. Semiempirical UNO-CAS and UNO-CI: Method and Applications in Nanoelectronics. J. Phys. Chem. A 2011, 115, 11303–11312. 10.1021/jp204939x. 21848343

[ref133] Sun H. COMPASS: An Ab Initio Force-Field Optimized for Condensed-Phase Applications - Overview with Details on Alkane and Benzene Compounds. J. Phys. Chem. B 1998, 102, 7338–7364. 10.1021/jp980939v.

[ref134] Rupp M. Machine Learning for Quantum Mechanics in a Nutshell. Int. J. Quantum Chem. 2015, 115, 1058 10.1002/qua.24954.

[ref135] Kranz J. J.; Kubillus M.; Ramakrishnan R.; Von Lilienfeld O. A.; Elstner M. Generalized Density-Functional Tight-Binding Repulsive Potentials from Unsupervised Machine Learning. J. Chem. Theory Comput. 2018, 14, 2341 10.1021/acs.jctc.7b00933. 29579387

[ref136] Hohenberg P.; Kohn W. Inhomogeneous Electron Gas. Phys. Rev. 1964, 136, B864–B871. 10.1103/PhysRev.136.B864.

[ref137] Kohn W.; Sham L. J. Self-Consistent Equations Including Exchange and Correlation Effects. Phys. Rev. 1965, 140, A1133 10.1103/PhysRev.140.A1133.

[ref138] Becke A. D. Density Functional Calculations of Molecular Bond Energies. J. Chem. Phys. 1986, 84, 4524–4529. 10.1063/1.450025.

[ref139] Perdew J. P.; Burke K.; Ernzerhof M. Generalized Gradient Approximation Made Simple. Phys. Rev. Lett. 1996, 77, 3865–3868. 10.1103/PhysRevLett.77.3865. 10062328

[ref140] Perdew J. P.; Burke K.; Ernzerhof M. Erratum: Generalized Gradient Approximation Made Simple (Physical Review Letters (1996) 77 (3865)). Phys. Rev. Lett. 1997, 78, 1396 10.1103/PhysRevLett.78.1396. 10062328

[ref141] Stephens P. J.; Devlin F. J.; Chabalowski C. F.; Frisch M. J. Ab Initio Calculation of Vibrational Absorption and Circular Dichroism Spectra Using Density Functional Force Fields. J. Phys. Chem. 1994, 98, 11623–11627. 10.1021/j100096a001.

[ref142] Becke A. D. Density-functional Thermochemistry. III. The Role of Exact Exchange. J. Chem. Phys. 1993, 98, 5648–5652. 10.1063/1.464913.

[ref143] Perdew J. P.; Wang Y. Accurate and Simple Analytic Representation of the Electron-Gas Correlation Energy. Phys. Rev. B 1992, 45, 13244 10.1103/PhysRevB.45.13244. 10001404

[ref144] Frisch M. J.; Trucks G. W.; Schlegel H. B.; Scuseria G. E.; Robb M. a.; Cheeseman J. R.; Scalmani G.; Barone V.; Petersson G. a.; Nakatsuji H.; Gaussian 16, Revision C01; Gaussian, Inc.: Wallingford, CT, 2016.

[ref145] Frisch M. J.; Trucks G. W.; Schlegel H. B.; Scuseria G. E.; Robb M. A.; Cheeseman J. R.; Scalmani G.; Barone V.; Mennucci B.; Petersson G. A.; . Gaussian 09, Revision B.01; Gaussian, Inc.: Wallingford, CT, 2009.

[ref146] Valiev M.; Bylaska E. J.; Govind N.; Kowalski K.; Straatsma T. P.; Van Dam H. J. J.; Wang D.; Nieplocha J.; Apra E.; Windus T. L.; et al. NWChem: A Comprehensive and Scalable Open-Source Solution for Large Scale Molecular Simulations. Comput. Phys. Commun. 2010, 181, 1477–1489. 10.1016/j.cpc.2010.04.018.

[ref147] Burke K. Perspective on Density Functional Theory. J. Chem. Phys. 2012, 136, 150901 10.1063/1.4704546. 22519306

[ref148] Toulouse J.; Colonna F.; Savin A. Long-Range-Short-Range Separation of the Electron-Electron Interaction in Density-Functional Theory. Phys. Rev. A 2004, 70, 062505 10.1103/PhysRevA.70.062505.

[ref149] Gerber I. C.; Ángyán J. G. Hybrid Functional with Separated Range. Chem. Phys. Lett. 2005, 415, 100–105. 10.1016/j.cplett.2005.08.060.

[ref150] Grimme S.; Waletzke M. A Combination of Kohn-Sham Density Functional Theory and Multi-Reference Configuration Interaction Methods. J. Chem. Phys. 1999, 111, 5645–5655. 10.1063/1.479866.

[ref151] Neville S. P.; Schuurman M. S. Removing the Deadwood from DFT/MRCI Wave Functions: The p-DFT/MRCI Method. J. Chem. Theory Comput. 2021, 17, 7657–7665. 10.1021/acs.jctc.1c00959. 34861111

[ref152] Marian C. M.; Heil A.; Kleinschmidt M. The DFT/MRCI Method. WIREs Comput. Mol. Sci. 2019, 9, e1394 10.1002/wcms.1394.

[ref153] Cohen A. J.; Mori-Sánchez P.; Yang W. Challenges for Density Functional Theory. Chem. Rev. 2012, 112, 289–320. 10.1021/cr200107z. 22191548

[ref154] Kristyán S.; Pulay P. Can (Semi)Local Density Functional Theory Account for the London Dispersion Forces?. Chem. Phys. Lett. 1994, 229, 175–180. 10.1016/0009-2614(94)01027-7.

[ref155] Grimme S.; Antony J.; Ehrlich S.; Krieg H. A Consistent and Accurate Ab Initio Parametrization of Density Functional Dispersion Correction (DFT-D) for the 94 Elements H-Pu. J. Chem. Phys. 2010, 132, 154104 10.1063/1.3382344. 20423165

[ref156] Bao J. L.; Gagliardi L.; Truhlar D. G. Self-Interaction Error in Density Functional Theory: An Appraisal. J. Phys. Chem. Lett. 2018, 9, 2353–2358. 10.1021/acs.jpclett.8b00242. 29624392

[ref157] Pederson M. R.; Ruzsinszky A.; Perdew J. P. Communication: Self-Interaction Correction with Unitary Invariance in Density Functional Theory. J. Chem. Phys. 2014, 140, 121103 10.1063/1.4869581. 24697415

[ref158] Li C.; Zheng X.; Su N. Q.; Yang W. Localized Orbital Scaling Correction for Systematic Elimination of Delocalization Error in Density Functional Approximations. Natl. Sci. Rev. 2018, 5, 203–215. 10.1093/nsr/nwx111.

[ref159] Gagliardi L.; Truhlar D. G.; Li Manni G.; Carlson R. K.; Hoyer C. E.; Bao J. L. Multiconfiguration Pair-Density Functional Theory: A New Way to Treat Strongly Correlated Systems. Acc. Chem. Res. 2017, 50, 66–73. 10.1021/acs.accounts.6b00471. 28001359

[ref160] Shi B.; Nachtigallová D.; Aquino A. J. A.; Machado F. B. C.; Lischka H. Excited States and Excitonic Interactions in Prototypic Polycyclic Aromatic Hydrocarbon Dimers as Models for Graphitic Interactions in Carbon Dots. Phys. Chem. Chem. Phys. 2019, 21, 9077–9088. 10.1039/C9CP00635D. 30869712

[ref161] Sutton A. P.; Finnis M. W.; Pettifor D. G.; Ohta Y. The Tight-Binding Bond Model. J. Phys. C. Solid State Phys. 1988, 21, 35–66. 10.1088/0022-3719/21/1/007.

[ref162] Sankey O. F.; Niklewski D. J. Ab Initio Multicenter Tight-Binding Model for Molecular-Dynamics Simulations and Other Applications in Covalent Systems. Phys. Rev. B 1989, 40, 3979 10.1103/PhysRevB.40.3979. 9992372

[ref163] Harris J. Simplified Method for Calculating the Energy of Weakly Interacting Fragments. Phys. Rev. B 1985, 31, 1770 10.1103/PhysRevB.31.1770. 9935980

[ref164] Foulkes W. M. C.; Haydock R. Tight-Binding Models and Density-Functional Theory. Phys. Rev. B 1989, 39, 12520 10.1103/PhysRevB.39.12520. 9948117

[ref165] Lewis J. P.; Glaesemann K. R.; Voth G. A.; Fritsch J.; Demkov A. A.; Ortega J.; Sankey O. F. Further Developments in the Local-Orbital Density-Functional-Theory Tight-Binding Method. Phys. Rev. B - Condens. Matter Mater. Phys. 2001, 64, 195103 10.1103/PhysRevB.64.195103.

[ref166] Tu Y.; Jacobsson S. P.; Laaksonen A. Efficient Ab Initio Tight-Binding-like Method for Electronic Structure Calculations. Phys. Rev. B - Condens. Matter Mater. Phys. 2006, 74, 205104 10.1103/PhysRevB.74.205104.

[ref167] Niehaus T. A. Approximate Time-Dependent Density Functional Theory. J. Mol. Struct. THEOCHEM 2009, 914, 38 10.1016/j.theochem.2009.04.034.

[ref168] Koskinen P.; Mäkinen V. Density-Functional Tight-Binding for Beginners. Comput. Mater. Sci. 2009, 47, 237 10.1016/j.commatsci.2009.07.013.

[ref169] Seifert G.; Joswig J. O. Density-Functional Tight Binding-an Approximate Density-Functional Theory Method. Wiley Interdiscip. Rev. Comput. Mol. Sci. 2012, 2, 456–465. 10.1002/wcms.1094.

[ref170] Christensen A. S.; Kubař T.; Cui Q.; Elstner M. Semiempirical Quantum Mechanical Methods for Noncovalent Interactions for Chemical and Biochemical Applications. Chem. Rev. 2016, 116, 5301–5337. 10.1021/acs.chemrev.5b00584. 27074247 PMC4867870

[ref171] Hourahine B.; Aradi B.; Blum V.; Bonafé F.; Buccheri A.; Camacho C.; Cevallos C.; Deshaye M. Y.; Dumitrica T.; Dominguez A. DFTB+, a Software Package for Efficient Approximate Density Functional Theory Based Atomistic Simulations. J. Chem. Phys. 2020, 152, 124101 10.1063/1.5143190. 32241125

[ref172] Cardias R.; Barreteau C.; Thibaudeau P.; Fu C. C. Spin Dynamics from a Constrained Magnetic Tight-Binding Model. Phys. Rev. B 2021, 103, 235436 10.1103/PhysRevB.103.235436.

[ref173] Bannwarth C.; Caldeweyher E.; Ehlert S.; Hansen A.; Pracht P.; Seibert J.; Spicher S.; Grimme S. Extended Tight-Binding Quantum Chemistry Methods. WIREs Comput. Mol. Sci. 2021, 11, e1493 10.1002/wcms.1493.

[ref174] Ozfidan I.; Korkusinski M.; Hawrylak P. Electronic Properties and Electron-Electron Interactions in Graphene Quantum Dots. Phys. Status Solidi - Rapid Res. Lett. 2016, 10, 13–23. 10.1002/pssr.201510251.

[ref175] Zeng Z.; Zhang W.; Arvapalli D. M.; Bloom B.; Sheardy A.; Mabe T.; Liu Y.; Ji Z.; Chevva H.; Waldeck D. H.; et al. A Fluorescence-Electrochemical Study of Carbon Nanodots (CNDs) in Bio- and Photoelectronic Applications and Energy Gap Investigation. Phys. Chem. Chem. Phys. 2017, 19, 20101–20109. 10.1039/C7CP02875J. 28726895 PMC5714648

[ref176] Marques M. A. L.; Gross E. K. U. Time-Dependent Density Functional Theory. Annu. Rev. Phys. Chem. 2004, 55, 427–455. 10.1146/annurev.physchem.55.091602.094449. 15117259

[ref177] Ullrich C. A.; Yang Z. A Brief Compendium of Time-Dependent Density Functional Theory. Brazilian J. Phys. 2014, 44, 154–188. 10.1007/s13538-013-0141-2.

[ref178] Casida M. E. Time-Dependent Density-Functional Theory for Molecules and Molecular Solids. J. Mol. Struct. THEOCHEM 2009, 914, 3–18. 10.1016/j.theochem.2009.08.018.

[ref179] Runge E.; Gross E. K. U. Density-Functional Theory for Time-Dependent Systems. Phys. Rev. Lett. 1984, 52, 997–1000. 10.1103/PhysRevLett.52.997.

[ref180] Liebman J. F. A Review of “Time-Dependent Density-Functional Theory: Concepts and Applications.. Mol. Cryst. Liq. Cryst. 2012, 569, 165–166. 10.1080/15421406.2012.711647.

[ref181] Bauernschmitt R.; Ahlrichs R. Treatment of Electronic Excitations within the Adiabatic Approximation of Time Dependent Density Functional Theory. Chem. Phys. Lett. 1996, 256, 454–464. 10.1016/0009-2614(96)00440-X.

[ref182] Franco de Carvalho F.; Tavernelli I. Nonadiabatic Dynamics with Intersystem Crossings: A Time-Dependent Density Functional Theory Implementation. J. Chem. Phys. 2015, 143, 224105 10.1063/1.4936864. 26671356

[ref183] Thiele M.; Gross E. K. U.; Kümmel S. Adiabatic Approximation in Nonperturbative Time-Dependent Density-Functional Theory. Phys. Rev. Lett. 2008, 100, 153004 10.1103/PhysRevLett.100.153004. 18518104

[ref184] Perdew J. P.; Zunger A. Self-Interaction Correction to Density-Functional Approximations for Many-Electron Systems. Phys. Rev. B 1981, 23, 5048–5079. 10.1103/PhysRevB.23.5048.

[ref185] Becke A. D. Density-Functional Exchange-Energy Approximation with Correct Asymptotic Behavior. Phys. Rev. A 1988, 38, 3098–3100. 10.1103/PhysRevA.38.3098. 9900728

[ref186] Castro A.; Appel H.; Oliveira M.; Rozzi C. A.; Andrade X.; Lorenzen F.; Marques M. A. L.; Gross E. K. U.; Rubio A. Octopus: A Tool for the Application of Time-Dependent Density Functional Theory. Phys. Status Solidi Basic Res. 2006, 243, 2465–2488. 10.1002/pssb.200642067.

[ref187] Furche F.; Ahlrichs R.; Hättig C.; Klopper W.; Sierka M.; Weigend F. Turbomole. Wiley Interdiscip. Rev. Comput. Mol. Sci. 2014, 4, 91–100. 10.1002/wcms.1162.

[ref188] Casida M. E.; Gutierrez F.; Guan J.; Gadea F.-X.; Salahub D.; Daudey J.-P. Charge-Transfer Correction for Improved Time-Dependent Local Density Approximation Excited-State Potential Energy Curves: Analysis within the Two-Level Model with Illustration for H2 and LiH. J. Chem. Phys. 2000, 113, 7062–7071. 10.1063/1.1313558.

[ref189] Sternheimer R. On Nuclear Quadrupole Moments. Phys. Rev. 1951, 84, 244–253. 10.1103/PhysRev.84.244.

[ref190] Laurent A. D.; Jacquemin D. TD-DFT Benchmarks: A Review. Int. J. Quantum Chem. 2013, 113, 2019–2039. 10.1002/qua.24438.

[ref191] Zuehlsdorff T. J.; Hine N. D. M.; Spencer J. S.; Harrison N. M.; Riley D. J.; Haynes P. D. Linear-Scaling Time-Dependent Density-Functional Theory in the Linear Response Formalism. J. Chem. Phys. 2013, 139, 064104 10.1063/1.4817330. 23947840

[ref192] Rohrdanz M. A.; Martins K. M.; Herbert J. M. A Long-Range-Corrected Density Functional That Performs Well for Both Ground-State Properties and Time-Dependent Density Functional Theory Excitation Energies, Including Charge-Transfer Excited States. J. Chem. Phys. 2009, 130, 054112 10.1063/1.3073302. 19206963

[ref193] Shao Y.; Mei Y.; Sundholm D.; Kaila V. R. I. Benchmarking the Performance of Time-Dependent Density Functional Theory Methods on Biochromophores. J. Chem. Theory Comput. 2020, 16, 587–600. 10.1021/acs.jctc.9b00823. 31815476 PMC7391796

[ref194] Jacquemin D.; Wathelet V.; Perpète E. A.; Adamo C. Extensive TD-DFT Benchmark: Singlet-Excited States of Organic Molecules. J. Chem. Theory Comput. 2009, 5, 2420–2435. 10.1021/ct900298e. 26616623

[ref195] Brémond E.; Savarese M.; Adamo C.; Jacquemin D. Accuracy of TD-DFT Geometries: A Fresh Look. J. Chem. Theory Comput. 2018, 14, 3715–3727. 10.1021/acs.jctc.8b00311. 29883546

[ref196] Jacquemin D.; Perpète E. A.; Scuseria G. E.; Ciofini I.; Adamo C. TD-DFT Performance for the Visible Absorption Spectra of Organic Dyes: Conventional versus Long-Range Hybrids. J. Chem. Theory Comput. 2008, 4, 123–135. 10.1021/ct700187z. 26619986

[ref197] Guido C. A.; Jacquemin D.; Adamo C.; Mennucci B. On the TD-DFT Accuracy in Determining Single and Double Bonds in Excited-State Structures of Organic Molecules. J. Phys. Chem. A 2010, 114, 13402–13410. 10.1021/jp109218z. 21126028

[ref198] Car R.; Parrinello M. Unified Approach for Molecular Dynamics and Density-Functional Theory. Phys. Rev. Lett. 1985, 55, 2471–2474. 10.1103/PhysRevLett.55.2471. 10032153

[ref199] Rothlisberger U. 15 Years of Car-Parrinello Simulations in Physics, Chemistry and Biology. Comput. Chem.: Rev. Curr. Trends 2001, 6, 33–68. 10.1142/9789812799937_0002.

[ref200] Hutter J. Car-Parrinello Molecular Dynamics. Wiley Interdiscip. Rev. Comput. Mol. Sci. 2012, 2, 604–612. 10.1002/wcms.90.

[ref201] Murphy R. B.; Philipp D. M.; Friesner R. A. A Mixed Quantum Mechanics/Molecular Mechanics (QM/MM) Method for Large-Scale Modeling of Chemistry in Protein Environments. J. Comput. Chem. 2000, 21, 1442–1457. 10.1002/1096-987X(200012)21:16<1442::AID-JCC3>3.0.CO;2-O.

[ref202] Jensen F. Atomic Orbital Basis Sets. Wiley Interdiscip. Rev. Comput. Mol. Sci. 2013, 3, 273–295. 10.1002/wcms.1123.

[ref203] Payne M. C.; Teter M. P.; Allan D. C.; Arias T. A.; Joannopoulos J. D. Iterative Minimization Techniques for *Ab Initio* Total-Energy Calculations: Molecular Dynamics and Conjugate Gradients. Rev. Mod. Phys. 1992, 64, 1045 10.1103/RevModPhys.64.1045.

[ref204] Boese A. D.; Martin J. M. L.; Handy N. C. The Role of the Basis Set: Assessing Density Functional Theory. J. Chem. Phys. 2003, 119, 3005 10.1063/1.1589004.

[ref205] Marx D.; Hutter J. Ab Initio Molecular Dynamics; Cambridge University Press: Cambridge, U.K., 2009.

[ref206] Burden F. R.; Wilson R. M. Optimum Atomic Orbitals for Molecular Calculations A Review. Adv. Phys. 1972, 21, 825–915. 10.1080/00018737200101388.

[ref207] Dupuis M.; Rys J.; King H. F. Evaluation of Molecular Integrals over Gaussian Basis Functions. J. Chem. Phys. 1976, 65, 111 10.1063/1.432807.

[ref208] Slater J. C. Atomic Shielding Constants. Phys. Rev. 1930, 36, 57 10.1103/PhysRev.36.57.

[ref209] Hill J. G. Gaussian Basis Sets for Molecular Applications. Int. J. Quantum Chem. 2013, 113, 21–34. 10.1002/qua.24355.

[ref210] Ulian G.; Tosoni S.; Valdrè G. Comparison between Gaussian-Type Orbitals and Plane Wave Ab Initio Density Functional Theory Modeling of Layer Silicates: Talc [Mg3Si4O10(OH)2] as Model System. J. Chem. Phys. 2013, 139, 204101 10.1063/1.4830405. 24289338

[ref211] Hehre W. J.; Stewart R. F.; Pople J. A. Self-Consistent Molecular-Orbital Methods. I. Use of Gaussian Expansions of Slater-Type Atomic Orbitals. J. Chem. Phys. 1969, 51, 2657–2664. 10.1063/1.1672392.

[ref212] Stewart R. F. Small Gaussian Expansions of Slater-Type Orbitals. J. Chem. Phys. 1970, 52, 431 10.1063/1.1672702.

[ref213] Clark T.; Chandrasekhar J.; Spitznagel G. W.; Schleyer P. V. R. Efficient Diffuse Function-Augmented Basis Sets for Anion Calculations. III. The 3-21+G Basis Set for First-Row Elements, Li-F. J. Comput. Chem. 1983, 4, 294–301. 10.1002/jcc.540040303.

[ref214] Frisch M. J.; Pople J. A.; Binkley J. S. Self-consistent Molecular Orbital Methods 25. Supplementary Functions for Gaussian Basis Sets. J. Chem. Phys. 1984, 80, 3265–3269. 10.1063/1.447079.

[ref215] Seel M.; Del Re G. AccurateSCF Computations on Hydrogen Bonds: Role of Polarization Functions on the Bridge Hydrogen Atom. Int. J. Quantum Chem. 1986, 30, 563–566. 10.1002/qua.560300408.

[ref216] Jaffe R. L.; Smith G. D. A Quantum Chemistry Study of Benzene Dimer. J. Chem. Phys. 1996, 105, 2780–2788. 10.1063/1.472140.

[ref217] Tsuzuki S.; Uchimaru T.; Mikami M.; Tanabe K. Basis Set Effects on the Calculated Bonding Energies of Neutral Benzene Dimers: Importance of Diffuse Polarization Functions. Chem. Phys. Lett. 1996, 252, 206–210. 10.1016/0009-2614(96)00173-X.

[ref218] Papajak E.; Zheng J.; Xu X.; Leverentz H. R.; Truhlar D. G. Perspectives on Basis Sets Beautiful: Seasonal Plantings of Diffuse Basis Functions. J. Chem. Theory Comput. 2011, 7, 3027–3034. 10.1021/ct200106a. 26598144

[ref219] Wiberg K. B.; Hadad C. M.; Foresman J. B.; Chupka W. A. Electronically Excited States of Ethylene. J. Phys. Chem. 1992, 96, 10756–10768. 10.1021/j100205a032.

[ref220] Morgan W. J.; Fortenberry R. C. Additional Diffuse Functions in Basis Sets for Dipole-Bound Excited States of Anions. Theor. Chem. Acc. 2015, 134, 47 10.1007/s00214-015-1647-1.

[ref221] Jacquemin D.; Perpète E. A.; Adamo C. Modelling the UV/Visible Spectrum of Tetrakis(Phenylethynyl)Benzene. J. Mol. Struct. THEOCHEM 2008, 863, 123–127. 10.1016/j.theochem.2008.05.026.

[ref222] Fülscher M. P.; Roos B. O. The Excited States of Pyrazine: A Basis Set Study. Theor. Chim. Acta 1994, 87, 403–413. 10.1007/BF01113393.

[ref223] Jacquemin D.; Adamo C. Basis Set and Functional Effects on Excited-State Properties: Three Bicyclic Chromogens as Working Examples. Int. J. Quantum Chem. 2012, 112, 2135–2141. 10.1002/qua.23208.

[ref224] Dong S. S.; Gagliardi L.; Truhlar D. G. Nature of the 11 Bu and 21 Ag Excited States of Butadiene and the Goldilocks Principle of Basis Set Diffuseness. J. Chem. Theory Comput. 2019, 15, 4591–4601. 10.1021/acs.jctc.9b00549. 31306007

[ref225] Ditchfield R.; Hehre W. J.; Pople J. A. Self-Consistent Molecular-Orbital Methods. IX. An Extended Gaussian-Type Basis for Molecular-Orbital Studies of Organic Molecules. J. Chem. Phys. 1971, 54, 724–728. 10.1063/1.1674902.

[ref226] Martin R. M. Electronic Structure: Basic Theory and Practical Methods; Cambridge University Press, 2004; 10.1017/CBO9780511805769

[ref227] Paizs B.; Suhai S. Comparative Study of BSSE Correction Methods at DFT and MP2 Levels of Theory. J. Comput. Chem. 1998, 19, 575 10.1002/(SICI)1096-987X(19980430)19:6<575::AID-JCC1>3.0.CO;2-O.

[ref228] Aidas K.; Ågren H.; Kongsted J.; Laaksonen A.; Mocci F. A Quantum Mechanics/Molecular Dynamics Study of Electric Field Gradient Fluctuations in the Liquid Phase. The Case of Na + in Aqueous Solution. Phys. Chem. Chem. Phys. 2013, 15, 1621–1631. 10.1039/C2CP41993A. 23247548

[ref229] Tapia O.; Goscinski O. Self-Consistent Reaction Field Theory of Solvent Effects. Mol. Phys. 1975, 29, 1653–1661. 10.1080/00268977500101461.

[ref230] Tomasi J.; Mennucci B.; Cammi R. Quantum Mechanical Continuum Solvation Models. Chem. Rev. 2005, 105, 2999–3093. 10.1021/cr9904009. 16092826

[ref231] Klamt A. The COSMO and COSMO-RS Solvation Models. Wiley Interdiscip. Rev. Comput. Mol. Sci. 2011, 1, 699–709. 10.1002/wcms.56.

[ref232] Marenich A. V.; Cramer C. J.; Truhlar D. G. Universal Solvation Model Based on Solute Electron Density and on a Continuum Model of the Solvent Defined by the Bulk Dielectric Constant and Atomic Surface Tensions. J. Phys. Chem. B 2009, 113, 6378–6396. 10.1021/jp810292n. 19366259

[ref233] Herbert J. M. Dielectric Continuum Methods for Quantum Chemistry. WIREs Comput. Mol. Sci. 2021, 11, e1519 10.1002/wcms.1519.

[ref234] Ehlert S.; Stahn M.; Spicher S.; Grimme S. Robust and Efficient Implicit Solvation Model for Fast Semiempirical Methods. J. Chem. Theory Comput. 2021, 17, 4250–4261. 10.1021/acs.jctc.1c00471. 34185531

[ref235] Tomasi J. Thirty Years of Continuum Solvation Chemistry: A Review, and Prospects for the near Future. Theor. Chem. Acc. 2004, 112, 184–203. 10.1007/s00214-004-0582-3.

[ref236] Shi B.; Nachtigallová D.; Aquino A. J. A.; Machado F. B. C.; Lischka H. High-Level Theoretical Benchmark Investigations of the UV-Vis Absorption Spectra of Paradigmatic Polycyclic Aromatic Hydrocarbons as Models for Graphene Quantum Dots. J. Chem. Phys. 2019, 150, 124302 10.1063/1.5086760. 30927896

[ref237] Fu M.; Ehrat F.; Wang Y.; Milowska K. Z.; Reckmeier C.; Rogach A. L.; Stolarczyk J. K.; Urban A. S.; Feldmann J. Carbon Dots: A Unique Fluorescent Cocktail of Polycyclic Aromatic Hydrocarbons. Nano Lett. 2015, 15, 6030–6035. 10.1021/acs.nanolett.5b02215. 26269962

[ref238] Yan X.; Cui X.; Li B.; Li L. S. Large, Solution-Processable Graphene Quantum Dots as Light Absorbers for Photovoltaics. Nano Lett. 2010, 10, 1869 10.1021/nl101060h. 20377198

[ref239] Yan X.; Li B.; Cui X.; Wei Q.; Tajima K.; Li L. S. Independent Tuning of the Band Gap and Redox Potential of Graphene Quantum Dots. J. Phys. Chem. Lett. 2011, 2, 1119–1124. 10.1021/jz200450r. 26295312

[ref240] Shao X.; Aquino A. J. A.; Otyepka M.; Nachtigallová D.; Lischka H. Tuning the UV Spectrum of PAHs by Means of Different N-Doping Types Taking Pyrene as Paradigmatic Example: Categorization: Via Valence Bond Theory and High-Level Computational Approaches. Phys. Chem. Chem. Phys. 2020, 22, 22003–22015. 10.1039/D0CP02688C. 32975249

[ref241] Angeli C.; Cimiraglia R.; Evangelisti S.; Leininger T.; Malrieu J.-P. Introduction of N-Electron Valence States for Multireference Perturbation Theory. J. Chem. Phys. 2001, 114, 10252–10264. 10.1063/1.1361246.

[ref242] Angeli C.; Cimiraglia R.; Malrieu J.-P. N-Electron Valence State Perturbation Theory: A Fast Implementation of the Strongly Contracted Variant. Chem. Phys. Lett. 2001, 350, 297–305. 10.1016/S0009-2614(01)01303-3.

[ref243] Neese F. A Spectroscopy Oriented Configuration Interaction Procedure. J. Chem. Phys. 2003, 119, 9428–9443. 10.1063/1.1615956.

[ref244] Kranz J. J.; Elstner M.; Aradi B.; Frauenheim T.; Lutsker V.; Garcia A. D.; Niehaus T. A. Time-Dependent Extension of the Long-Range Corrected Density Functional Based Tight-Binding Method. J. Chem. Theory Comput. 2017, 13, 1737–1747. 10.1021/acs.jctc.6b01243. 28272887

[ref245] Humeniuk A.; Mitrić R. Long-Range Correction for Tight-Binding TD-DFT. J. Chem. Phys. 2015, 143, 134120 10.1063/1.4931179. 26450305

[ref246] Jones J. E. On the Determination of Molecular Fields. —II. From the Equation of State of a Gas. Proc. R. Soc. Lond. A 1924, 106, 463–477. 10.1098/rspa.1924.0082.

[ref247] Kumari R.; Kumar R.; Lynn A. G_mmpbsa —A GROMACS Tool for High-Throughput MM-PBSA Calculations. J. Chem. Inf. Model. 2014, 54, 1951–1962. 10.1021/ci500020m. 24850022

[ref248] Schutz C. N.; Warshel A. What Are the Dielectric ?Constants? Of Proteins and How to Validate Electrostatic Models?. Proteins Struct. Funct. Genet. 2001, 44, 400–417. 10.1002/prot.1106. 11484218

[ref249] Gasteiger J.; Marsili M. A New Model for Calculating Atomic Charges in Molecules. Tetrahedron Lett. 1978, 19, 3181–3184. 10.1016/S0040-4039(01)94977-9.

[ref250] Mulliken R. S. Electronic Population Analysis on LCAO-MO Molecular Wave Functions. I. J. Chem. Phys. 1955, 23, 1833–1840. 10.1063/1.1740588.

[ref251] Momany F. A. Determination of Partial Atomic Charges from Ab Initio Molecular Electrostatic Potentials. Application to Formamide, Methanol, and Formic Acid. J. Phys. Chem. 1978, 82, 592–601. 10.1021/j100494a019.

[ref252] Cornell W. D.; Cieplak P.; Bayly C. I.; Kollman P. A. Application of RESP Charges To Calculate Conformational Energies, Hydrogen Bond Energies, and Free Energies of Solvation. J. Am. Chem. Soc. 1993, 115, 9620–9631. 10.1021/ja00074a030.

[ref253] Bayly C. I.; Cieplak P.; Cornell W. D.; Kollman P. A. A Well-Behaved Electrostatic Potential Based Method Using Charge Restraints for Deriving Atomic Charges: The RESP Model. J. Phys. Chem. 1993, 97, 10269–10280. 10.1021/j100142a004.

[ref254] Kontogeorgis G. M.; Economou I. G. Equations of State: From the Ideas of van Der Waals to Association Theories. J. Supercrit. Fluids 2010, 55, 421–437. 10.1016/j.supflu.2010.10.023.

[ref255] Goodwin A. R. H.; Sandler S. I. Chapter 5. Mixing and Combining Rules. In Applied Thermodynamics of Fluids; Goodwin A. R. H., Sengers J. V., Peters C. J., Eds.; Royal Society of Chemistry, 2010; pp 84–134.

[ref256] Delhommelle J.; Millié P. Inadequacy of the Lorentz-Berthelot Combining Rules for Accurate Predictions of Equilibrium Properties by Molecular Simulation. Mol. Phys. 2001, 99, 619–625. 10.1080/00268970010020041.

[ref257] Finnis M. W. Bond-Order Potentials through the Ages. Prog. Mater. Sci. 2007, 52, 133–153. 10.1016/j.pmatsci.2006.10.003.

[ref258] Zhou X. W.; Ward D. K.; Foster M. E. An Analytical Bond-Order Potential for Carbon. J. Comput. Chem. 2015, 36, 1719–1735. 10.1002/jcc.23949. 26018402

[ref259] Bixon M.; Lifson S. Potential Functions and Conformations in Cycloalkanes. Tetrahedron 1967, 23, 769–784. 10.1016/0040-4020(67)85023-3.

[ref260] Allinger N. L. Calculation of Molecular Structure and Energy by Force-Field Methods. Adv. Phys. Org. Chem. 1976, 13, 1–82. 10.1016/S0065-3160(08)60212-9.

[ref261] Lifson S. Potential Energy Functions for Structural Molecular Biology. In Structural Molecular Biology; NATO Advanced Study Institutes Series, Vol. 45; Davies D. B., Saenger W., Danyluk S. S., Eds.; Springer: Boston, MA, 1982; pp 359–385.

[ref262] Lifson S.; Warshel A. Consistent Force Field for Calculations of Conformations, Vibrational Spectra, and Enthalpies of Cycloalkane and n-Alkane Molecules. J. Chem. Phys. 1968, 49, 5116–5129. 10.1063/1.1670007.

[ref263] Lin F. Y.; MacKerell A. D. Force Fields for Small Molecules. Methods Mol. Biol. 2019, 2022, 21–54. 10.1007/978-1-4939-9608-7_2. 31396898 PMC6733265

[ref264] Brooks B. R.; Bruccoleri R. E.; Olafson B. D.; States D. J.; Swaminathan S.; Karplus M. CHARMM: A Program for Macromolecular Energy, Minimization, and Dynamics Calculations. J. Comput. Chem. 1983, 4, 187–217. 10.1002/jcc.540040211.

[ref265] MacKerell A. D.; Bashford D.; Bellott M.; Dunbrack R. L.; Evanseck J. D.; Field M. J.; Fischer S.; Gao J.; Guo H.; Ha S.; et al. All-Atom Empirical Potential for Molecular Modeling and Dynamics Studies of Proteins †. J. Phys. Chem. B 1998, 102, 3586–3616. 10.1021/jp973084f. 24889800

[ref266] Jorgensen W. L.; Maxwell D. S.; Tirado-Rives J. Development and Testing of the OPLS All-Atom Force Field on Conformational Energetics and Properties of Organic Liquids. J. Am. Chem. Soc. 1996, 118, 11225–11236. 10.1021/ja9621760.

[ref267] Cornell W. D.; Cieplak P.; Bayly C. I.; Gould I. R.; Merz K. M.; Ferguson D. M.; Spellmeyer D. C.; Fox T.; Caldwell J. W.; Kollman P. A. A Second Generation Force Field for the Simulation of Proteins, Nucleic Acids, and Organic Molecules. J. Am. Chem. Soc. 1995, 117, 5179–5197. 10.1021/ja00124a002.

[ref268] Case D. A.; Cheatham T. E.; Darden T.; Gohlke H.; Luo R.; Merz K. M.; Onufriev A.; Simmerling C.; Wang B.; Woods R. J. The Amber Biomolecular Simulation Programs. J. Comput. Chem. 2005, 26, 1668–1688. 10.1002/jcc.20290. 16200636 PMC1989667

[ref269] Elvati P.; Baumeister E.; Violi A. Graphene Quantum Dots: Effect of Size, Composition and Curvature on Their Assembly. RSC Adv. 2017, 7, 17704–17710. 10.1039/C7RA01029J.

[ref270] Vanommeslaeghe K.; Hatcher E.; Acharya C.; Kundu S.; Zhong S.; Shim J.; Darian E.; Guvench O.; Lopes P.; Vorobyov I.; et al. CHARMM General Force Field: A Force Field for Drug-like Molecules Compatible with the CHARMM All-Atom Additive Biological Force Fields. J. Comput. Chem. 2010, 31, 671–690. 10.1002/jcc.21367. 19575467 PMC2888302

[ref271] Paloncýová M.; Langer M.; Otyepka M. Structural Dynamics of Carbon Dots in Water and N, N -Dimethylformamide Probed by All-Atom Molecular Dynamics Simulations. J. Chem. Theory Comput. 2018, 14, 2076–2083. 10.1021/acs.jctc.7b01149. 29499118 PMC5905991

[ref272] Cheng A.; Steele W. A. Computer Simulation of Ammonia on Graphite. I. Low Temperature Structure of Monolayer and Bilayer Films. J. Chem. Phys. 1990, 92, 3858–3866. 10.1063/1.458562.

[ref273] Wang J.; Cieplak P.; Kollman P. A. How Well Does a Restrained Electrostatic Potential (RESP) Model Perform in Calculating Conformational Energies of Organic and Biological Molecules?. J. Comput. Chem. 2000, 21, 1049–1074. 10.1002/1096-987X(200009)21:12<1049::AID-JCC3>3.0.CO;2-F.

[ref274] Wang J.; Wolf R. M.; Caldwell J. W.; Kollman P. A.; Case D. A. Development and Testing of a General Amber Force Field. J. Comput. Chem. 2004, 25, 1157–1174. 10.1002/jcc.20035. 15116359

[ref275] Yang Q. Q.; He H.; Li C. Q.; Luo L. B.; Li S. L.; Xu Z. Q.; Jin J. C.; Jiang F. L.; Liu Y.; Yang M. Molecular Mechanisms of the Ultra-Strong Inhibition Effect of Oxidized Carbon Dots on Human Insulin Fibrillation. ACS Appl. Bio Mater. 2020, 3, 217–226. 10.1021/acsabm.9b00725. 35019438

[ref276] Martín C.; Jun G.; Schurhammer R.; Reina G.; Chen P.; Bianco A.; Ménard-Moyon C. Enzymatic Degradation of Graphene Quantum Dots by Human Peroxidases. Small 2019, 15, 1905405 10.1002/smll.201905405. 31769611

[ref277] Zhou M.; Shen Q.; Shen J. W.; Jin L.; Zhang L.; Sun Q.; Hu Q.; Liang L. Understanding the Size Effect of Graphene Quantum Dots on Protein Adsorption. Colloids Surfaces B Biointerfaces 2019, 174, 575–581. 10.1016/j.colsurfb.2018.11.059. 30502669

[ref278] Liang L.; Peng X.; Sun F.; Kong Z.; Shen J.-W. A Review on the Cytotoxicity of Graphene Quantum Dots: From Experiment to Simulation. Nanoscale Adv. 2021, 3, 904–917. 10.1039/D0NA00904K. PMC9419276 36133293

[ref279] Li Y.; Yuan H.; von dem Bussche A.; Creighton M.; Hurt R. H.; Kane A. B.; Gao H. Graphene Microsheets Enter Cells through Spontaneous Membrane Penetration at Edge Asperities and Corner Sites. Proc. Natl. Acad. Sci. U. S. A. 2013, 110, 12295–12300. 10.1073/pnas.1222276110. 23840061 PMC3725082

[ref280] Liang L.; Kong Z.; Kang Z.; Wang H.; Zhang L.; Shen J.-W. Theoretical Evaluation on Potential Cytotoxicity of Graphene Quantum Dots. ACS Biomater. Sci. Eng. 2016, 2, 1983–1991. 10.1021/acsbiomaterials.6b00390. 33440534

[ref281] Tang X.; Zhang S.; Zhou H.; Zhou B.; Liu S.; Yang Z. The Role of Electrostatic Potential Polarization in the Translocation of Graphene Quantum Dots across Membranes. Nanoscale2 2020, 12, 2732–2739. 10.1039/C9NR09258G. 31951244

[ref282] Cohen-Tanugi D.; Grossman J. C. Water Desalination across Nanoporous Graphene. Nano Lett. 2012, 12, 3602–3608. 10.1021/nl3012853. 22668008

[ref283] Huang J.; MacKerell A. D. CHARMM36 All-Atom Additive Protein Force Field: Validation Based on Comparison to NMR Data. J. Comput. Chem. 2013, 34, 2135–2145. 10.1002/jcc.23354. 23832629 PMC3800559

[ref284] Liu C.; Elvati P.; Majumder S.; Wang Y.; Liu A. P.; Violi A. Predicting the Time of Entry of Nanoparticles in Lipid Membranes. ACS Nano 2019, 13, 10221–10232. 10.1021/acsnano.9b03434. 31401835

[ref285] Erimban S.; Daschakraborty S. Translocation of a Hydroxyl Functionalized Carbon Dot across a Lipid Bilayer: An All-Atom Molecular Dynamics Simulation Study. Phys. Chem. Chem. Phys. 2020, 22, 6335–6350. 10.1039/C9CP05999G. 32134073

[ref286] van Gunsteren W. F.; Billeter S. R.; Eising A. A.; Hunenberger P. H.; Kruger P.; Mark A. E.; Scott W. R. P.; Tironi I. G. Biomolecular Simulation: The GROMOS96 Manual and User Guide; Vdf Hochschulverlag AG an der ETH Zurich: Zurich, Switzerland, 1996.

[ref287] Zhang S.-T.; Yan H.; Wei M.; Evans D. G.; Duan X. Valence Force Field for Layered Double Hydroxide Materials Based on the Parameterization of Octahedrally Coordinated Metal Cations. J. Phys. Chem. C 2012, 116, 3421–3431. 10.1021/jp211194w.

[ref288] Sun H.; Mumby S. J.; Maple J. R.; Hagler A. T. An Ab Initio CFF93 All-Atom Force Field for Polycarbonates. J. Am. Chem. Soc. 1994, 116, 2978–2987. 10.1021/ja00086a030.

[ref289] Liu W.; Xu S.; Liang R.; Wei M.; Evans D. G.; Duan X. In Situ Synthesis of Nitrogen-Doped Carbon Dots in the Interlayer Region of a Layered Double Hydroxide with Tunable Quantum Yield. J. Mater. Chem. C 2017, 5, 3536–3541. 10.1039/C6TC05463C.

[ref290] Maity A.; Pal U.; Chakraborty B.; Sengupta C.; Sau A.; Chakraborty S.; Basu S. Preferential Photochemical Interaction of Ru (III) Doped Carbon Nano Dots with Bovine Serum Albumin over Human Serum Albumin. Int. J. Biol. Macromol. 2019, 137, 483–494. 10.1016/j.ijbiomac.2019.06.126. 31265848

[ref291] Titov A. V.; Král P.; Pearson R. Sandwiched Graphene-Membrane Superstructures. ACS Nano 2010, 4, 229–234. 10.1021/nn9015778. 20025267

[ref292] Berger O.; Edholm O.; Jähnig F. Molecular Dynamics Simulations of a Fluid Bilayer of Dipalmitoylphosphatidylcholine at Full Hydration, Constant Pressure, and Constant Temperature. Biophys. J. 1997, 72, 2002–2013. 10.1016/S0006-3495(97)78845-3. 9129804 PMC1184396

[ref293] Yao C.; Tu Y.; Ding L.; Li C.; Wang J.; Fang H.; Huang Y.; Zhang K.; Lu Q.; Wu M.; et al. Tumor Cell-Specific Nuclear Targeting of Functionalized Graphene Quantum Dots In Vivo. Bioconjugate Chem. 2017, 28, 2608–2619. 10.1021/acs.bioconjchem.7b00466. 28903003

[ref294] Wu G.; Robertson D. H.; Brooks C. L.; Vieth M. Detailed Analysis of Grid-Based Molecular Docking: A Case Study of CDOCKER—A CHARMm-Based MD Docking Algorithm. J. Comput. Chem. 2003, 24, 1549 10.1002/jcc.10306. 12925999

[ref295] Xu L.; Dai Y.; Wang Z.; Zhao J.; Li F.; White J. C.; Xing B. Graphene Quantum Dots in Alveolar Macrophage: Uptake-Exocytosis, Accumulation in Nuclei, Nuclear Responses and DNA Cleavage. Part. Fibre Toxicol. 2018, 15, 45 10.1186/s12989-018-0279-8. 30424790 PMC6234698

[ref296] Xue Z.; Sun Q.; Zhang L.; Kang Z.; Liang L.; Wang Q.; Shen J. W. Graphene Quantum Dot Assisted Translocation of Drugs into a Cell Membrane. Nanoscale 2019, 11, 4503–4514. 10.1039/C8NR10091H. 30806416

[ref297] Wang Y.; Kadiyala U.; Qu Z.; Elvati P.; Altheim C.; Kotov N. A.; Violi A.; VanEpps J. S. Anti-Biofilm Activity of Graphene Quantum Dots via Self-Assembly with Bacterial Amyloid Proteins. ACS Nano 2019, 13, 4278–4289. 10.1021/acsnano.8b09403. 30912922 PMC6528478

[ref298] Jeong S.; Pinals R. L.; Dharmadhikari B.; Song H.; Kalluri A.; Debnath D.; Wu Q.; Ham M. H.; Patra P.; Landry M. P. Graphene Quantum Dot Oxidation Governs Noncovalent Biopolymer Adsorption. Sci. Rep. 2020, 10, 7074 10.1038/s41598-020-63769-z. 32341425 PMC7184744

[ref299] Dalosto S. D.; Tinte S. Fluctuation Effects of the Electric Field Induced by Water on a Graphene Dot Band Gap. J. Phys. Chem. C 2011, 115, 4381–4386. 10.1021/jp109297p.

[ref300] Santos S. G.; Santana J. V.; Maia F. F. J.; Lemos V.; Freire V. N.; Caetano E. W. S.; Cavada B. S.; Albuquerque E. L. Adsorption of Ascorbic Acid on the C60 Fullerene. J. Phys. Chem. B 2008, 112, 14267–14272. 10.1021/jp8048263. 18939786

[ref301] Wang Z.; Fang H.; Wang S.; Zhang F.; Wang D. Simulating Molecular Interactions of Carbon Nanoparticles with a Double-Stranded DNA Fragment. J. Chem. 2015, 2015, 531610 10.1155/2015/531610.

[ref302] Kong Z.; Hu W.; Jiao F.; Zhang P.; Shen J.; Cui B.; Wang H.; Liang L. Theoretical Evaluation of DNA Genotoxicity of Graphene Quantum Dots: A Combination of Density Functional Theory and Molecular Dynamics Simulations. J. Phys. Chem. B 2020, 124, 9335–9342. 10.1021/acs.jpcb.0c05882. 32870004

[ref303] Tu Y.; Lv M.; Xiu P.; Huynh T.; Zhang M.; Castelli M.; Liu Z.; Huang Q.; Fan C.; Fang H.; et al. Destructive Extraction of Phospholipids from Escherichia Coli Membranes by Graphene Nanosheets. Nat. Nanotechnol. 2013, 8, 594–601. 10.1038/nnano.2013.125. 23832191

[ref304] Fang G.; Luan B.; Ge C.; Chong Y.; Dong X.; Guo J.; Tang C.; Zhou R. Understanding the Graphene Quantum Dots-Ubiquitin Interaction by Identifying the Interaction Sites. Carbon N. Y. 2017, 121, 285–291. 10.1016/j.carbon.2017.05.096.

[ref305] Wang J.; Wei Y.; Shi X.; Gao H. Cellular Entry of Graphene Nanosheets: The Role of Thickness, Oxidation and Surface Adsorption. RSC Adv. 2013, 3, 15776–15782. 10.1039/c3ra40392k.

[ref306] Mao J.; Guo R.; Yan L.-T. Simulation and Analysis of Cellular Internalization Pathways and Membrane Perturbation for Graphene Nanosheets. Biomaterials 2014, 35, 6069–6077. 10.1016/j.biomaterials.2014.03.087. 24780168

[ref307] Dallavalle M.; Calvaresi M.; Bottoni A.; Melle-Franco M.; Zerbetto F. Graphene Can Wreak Havoc with Cell Membranes. ACS Appl. Mater. Interfaces 2015, 7, 4406–4414. 10.1021/am508938u. 25648559

[ref308] Chen P.; Yue H.; Zhai X.; Huang Z.; Ma G.-H.; Wei W.; Yan L.-T. Transport of a Graphene Nanosheet Sandwiched inside Cell Membranes. Sci. Adv. 2019, 5, eaaw3192 10.1126/sciadv.aaw3192. 31187061 PMC6555626

[ref309] Jimenez-Cruz C. A.; Kang S. gu; Zhou R. Large Scale Molecular Simulations of Nanotoxicity. Wiley Interdiscip. Rev. Syst. Biol. Med. 2014, 6, 329–343. 10.1002/wsbm.1271. 24894909

[ref310] Ouyang J. F.; Bettens R. P. A. Modelling Water: A Lifetime Enigma. Chimia (Aarau). 2015, 69, 104–111. 10.2533/chimia.2015.104. 26507212

[ref311] Demerdash O.; Wang L. P.; Head-Gordon T. Advanced Models for Water Simulations. WIREs Comput. Mol. Sci. 2018, 8, e1355 10.1002/wcms.1355.

[ref312] Halgren T. A.; Damm W. Polarizable Force Fields. Curr. Opin. Struct. Biol. 2001, 11, 236–242. 10.1016/S0959-440X(00)00196-2. 11297934

[ref313] Soloviev A. N.; Gruzdev R. U.; Jenny Lee C.-Y.; Tin H.-W.; C.-C Y. Chapter 38, Polarizable Models in Molecular Dynamics for Identification of Effective Properties. In Advanced Materials, Springer Proceedings in Physics 207; Parinov I. A., Gupta V. K., Chang S.-H., Eds.; Springer International Publishing AG, Part of Springer Nature, 2018; pp 487–493.

[ref314] Shi Y.; Ren P.; Schnieders M.; Piquemal J. P. Polarizable Force Fields for Biomolecular Modeling. Reviews in Computational Chemistry 2015, 28 (John Wiley & Sons, Inc.: Hoboken, NJ), 51–86. 10.1002/9781118889886.ch2.

[ref315] De Miranda Tomásio S.; Walsh T. R. Atomistic Modelling of the Interaction between Peptides and Carbon Nanotubes. Mol. Phys. 2007, 105, 221–229. 10.1080/00268970701197445.

[ref316] Shi Y.; Xia Z.; Zhang J.; Best R.; Wu C.; Ponder J. W.; Ren P. Polarizable Atomic Multipole-Based AMOEBA Force Field for Proteins. J. Chem. Theory Comput. 2013, 9, 4046–4063. 10.1021/ct4003702. 24163642 PMC3806652

[ref317] Van Duin A. C. T.; Dasgupta S.; Lorant F.; Goddard W. A. ReaxFF: A Reactive Force Field for Hydrocarbons. J. Phys. Chem. A 2001, 105, 9396–9409. 10.1021/jp004368u.

[ref318] Senftle T. P.; Hong S.; Islam M. M.; Kylasa S. B.; Zheng Y.; Shin Y. K.; Junkermeier C.; Engel-Herbert R.; Janik M. J.; Aktulga H. M. The ReaxFF Reactive Force-Field: Development, Applications and Future Directions. npj Comput. Mater. 2016, 2, 15011 10.1038/npjcompumats.2015.11.

[ref319] Srinivasan S. G.; van Duin A. C. T.; Ganesh P. Development of a ReaxFF Potential for Carbon Condensed Phases and Its Application to the Thermal Fragmentation of a Large Fullerene. J. Phys. Chem. A 2015, 119, 571–580. 10.1021/jp510274e. 25562718

[ref320] Gu S.; Hsieh C. Te; Ashraf Gandomi Y.; Chang J. K.; Li J.; Li J.; Zhang H.; Guo Q.; Lau K. C.; Pandey R. Microwave Growth and Tunable Photoluminescence of Nitrogen-Doped Graphene and Carbon Nitride Quantum Dots. J. Mater. Chem. C 2019, 7, 5468–5476. 10.1039/C9TC00233B.

[ref321] Weiner S. J.; Kollman P. A.; Case D. A.; Singh U. C.; Ghio C.; Alagona G.; Profeta S.; Weiner P. A New Force Field for Molecular Mechanical Simulation of Nucleic Acids and Proteins. J. Am. Chem. Soc. 1984, 106, 765–784. 10.1021/ja00315a051.

[ref322] Jorgensen W. L.; Tirado-Rives J. The OPLS Potential Functions for Proteins. Energy Minimizations for Crystals of Cyclic Peptides and Crambin. J. Am. Chem. Soc. 1988, 110, 1657–1666. 10.1021/ja00214a001. 27557051

[ref323] Marrink S. J.; Tieleman D. P. Perspective on the Martini Model. Chem. Soc. Rev. 2013, 42, 6801–6822. 10.1039/c3cs60093a. 23708257

[ref324] Noid W. G. Perspective: Coarse-Grained Models for Biomolecular Systems. J. Chem. Phys. 2013, 139, 090901 10.1063/1.4818908. 24028092

[ref325] Marrink S. J.; Risselada H. J.; Yefimov S.; Tieleman D. P.; de Vries A. H. The MARTINI Force Field: Coarse Grained Model for Biomolecular Simulations. J. Phys. Chem. B 2007, 111, 7812–7824. 10.1021/jp071097f. 17569554

[ref326] Lyubartsev A. P.; Laaksonen A. Calculation of Effective Interaction Potentials from Radial Distribution Functions: A Reverse Monte Carlo Approach. Phys. Rev. E 1995, 52, 3730–3737. 10.1103/PhysRevE.52.3730. 9963851

[ref327] Noid W. G.; Chu J.-W.; Ayton G. S.; Krishna V.; Izvekov S.; Voth G. A.; Das A.; Andersen H. C. The Multiscale Coarse-Graining Method. I. A Rigorous Bridge between Atomistic and Coarse-Grained Models. J. Chem. Phys. 2008, 128, 244114 10.1063/1.2938860. 18601324 PMC2671183

[ref328] Rowe P.; Csányi G.; Alfè D.; Michaelides A. Development of a Machine Learning Potential for Graphene. Phys. Rev. B 2018, 97, 054303 10.1103/PhysRevB.97.054303.

[ref329] Rowe P.; Deringer V. L.; Gasparotto P.; Csányi G.; Michaelides A. An Accurate and Transferable Machine Learning Potential for Carbon. J. Chem. Phys. 2020, 153, 034702 10.1063/5.0005084. 32716159

[ref330] Deringer V. L.; Csányi G. Machine Learning Based Interatomic Potential for Amorphous Carbon. Phys. Rev. B 2017, 95, 094203 10.1103/PhysRevB.95.094203.

[ref331] Noé F.; Tkatchenko A.; Müller K.-R.; Clementi C. Machine Learning for Molecular Simulation. Annu. Rev. Phys. Chem. 2020, 71, 361–390. 10.1146/annurev-physchem-042018-052331. 32092281

[ref332] Yu J.; Yong X.; Tang Z.; Yang B.; Lu S. Theoretical Understanding of Structure-Property Relationships in Luminescence of Carbon Dots. J. Phys. Chem. Lett. 2021, 12, 7671–7687. 10.1021/acs.jpclett.1c01856. 34351771

[ref333] Allen M. P.; Tildesley D. J. Computer Simulation of Liquids; Oxford University Press, 1989.

[ref334] Rapaport D. C. The Art of Molecular Dynamics Simulation, 2nd ed.; Cambridge University Press, 2011.

[ref335] Frenkel D.; Smit B. Understanding Molecular Simulation : From Algorithms to Applications, 2nd ed.; Academic Press: San Diego, CA, 2001.

[ref336] Alder B. J.; Wainwright T. E. Phase Transition for a Hard Sphere System. J. Chem. Phys. 1957, 27, 1208–1209. 10.1063/1.1743957.

[ref337] Alder B. J.; Wainwright T. E. Method. J. Chem. Phys. 1959, 31, 459–466. 10.1063/1.1730376.

[ref338] Stillinger F. H.; Rahman A. Improved Simulation of Liquid Water by Molecular Dynamics. J. Chem. Phys. 1974, 60, 1545–1557. 10.1063/1.1681229.

[ref339] Toxvaerd S. Newton’s Discrete Dynamics. arXiv 2020, 2003.02702.

[ref340] Verlet L. Computer “Experiments” on Classical Fluids. I. Thermodynamical Properties of Lennard-Jones Molecules. Phys. Rev. 1967, 159, 98–103. 10.1103/PhysRev.159.98.

[ref341] Ryckaert J.-P.; Ciccotti G.; Berendsen H. J. C. Numerical Integration of the Cartesian Equations of Motion of a System with Constraints: Molecular Dynamics of n-Alkanes. J. Comput. Phys. 1977, 23, 327–341. 10.1016/0021-9991(77)90098-5.

[ref342] Andersen H. C. Rattle: A “Velocity” Version of the Shake Algorithm for Molecular Dynamics Calculations. J. Comput. Phys. 1983, 52, 24–34. 10.1016/0021-9991(83)90014-1.

[ref343] Miyamoto S.; Kollman P. A. SETTLE: An Analytical Version of the SHAKE and RATTLE Algorithm for Rigid Water Models. J. Comput. Chem. 1992, 13, 952–962. 10.1002/jcc.540130805.

[ref344] Hess B.; Bekker H.; Berendsen H. J. C.; Fraaije J. G. E. M. LINCS: A Linear Constraint Solver for Molecular Simulations. J. Comput. Chem. 1997, 18, 1463–1472. 10.1002/(SICI)1096-987X(199709)18:12<1463::AID-JCC4>3.0.CO;2-H.

[ref345] Hess B. P-LINCS: A Parallel Linear Constraint Solver for Molecular Simulation. J. Chem. Theory Comput. 2008, 4, 116–122. 10.1021/ct700200b. 26619985

[ref346] Ewald P. P. Die Berechmmg Optischer Und Elektrostatischer Giltterpotentiale. Ann. Phys. 1921, 369, 253–287. 10.1002/andp.19213690304.

[ref347] Andersen H. C. Molecular Dynamics Simulations at Constant Pressure and/or Temperature. J. Chem. Phys. 1980, 72, 2384–2393. 10.1063/1.439486.

[ref348] Berendsen H. J. C.; Postma J. P. M.; van Gunsteren W. F.; Dinola A.; Haak J. R. Molecular Dynamics with Coupling to an External Bath. J. Chem. Phys. 1984, 81, 3684–3690. 10.1063/1.448118.

[ref349] Nose S. A Unified Formulation of the Constant Temperature Molecular Dynamics Methods. J. Chem. Phys. 1984, 81, 511–519. 10.1063/1.447334.

[ref350] Hoover W. G. Canonical Dynamics: Equilibrium Phase-Space Distributions. Phys. Rev. A 1985, 31, 1695–1697. 10.1103/PhysRevA.31.1695. 9895674

[ref351] Hünenberger P. H. Thermostat Algorithms for Molecular Dynamics Simulations. Adv. Polym. Sci. 2005, 173, 105–149. 10.1007/b99427.

[ref352] Grubmüller H.; Heymann B.; Tavan P. Ligand Binding: Molecular Mechanics Calculation of the Streptavidin-Biotin Rupture Force. Science (80-.). 1996, 271, 997–999. 10.1126/science.271.5251.997. 8584939

[ref353] Lee G. U.; Kidwell D. A.; Colton R. J. Sensing Discrete Streptavidin-Biotin Interactions with Atomic Force Microscopy. Langmuir 1994, 10, 354–357. 10.1021/la00014a003.

[ref354] Izrailev S.; Stepaniants S.; Balsera M.; Oono Y.; Schulten K. Molecular Dynamics Study of Unbinding of the Avidin-Biotin Complex. Biophys. J. 1997, 72, 1568–1581. 10.1016/S0006-3495(97)78804-0. 9083662 PMC1184352

[ref355] Isralewitz B.; Gao M.; Schulten K. Steered Molecular Dynamics and Mechanical Functions of Proteins. Curr. Opin. Struct. Biol. 2001, 11, 224–230. 10.1016/S0959-440X(00)00194-9. 11297932

[ref356] Torrie G. M.; Valleau J. P. Nonphysical Sampling Distributions in Monte Carlo Free-Energy Estimation: Umbrella Sampling. J. Comput. Phys. 1977, 23, 187–199. 10.1016/0021-9991(77)90121-8.

[ref357] Park S.; Schulten K. Calculating Potentials of Mean Force from Steered Molecular Dynamics Simulations. J. Chem. Phys. 2004, 120, 5946–5961. 10.1063/1.1651473. 15267476

[ref358] Jarzynski C. Nonequilibrium Equality for Free Energy Differences. Phys. Rev. Lett. 1997, 78, 2690–2693. 10.1103/PhysRevLett.78.2690.

[ref359] Warshel A.; Levitt M. Theoretical Studies of Enzymic Reactions: Dielectric, Electrostatic and Steric Stabilization of the Carbonium Ion in the Reaction of Lysozyme. J. Mol. Biol. 1976, 103, 227–249. 10.1016/0022-2836(76)90311-9. 985660

[ref360] Singh U. C.; Kollman P. A. A Combined Ab Initio Quantum Mechanical and Molecular Mechanical Method for Carrying out Simulations on Complex Molecular Systems: Applications to the CH3Cl + Cl– Exchange Reaction and Gas Phase Protonation of Polyethers. J. Comput. Chem. 1986, 7, 718–730. 10.1002/jcc.540070604.

[ref361] Field M. J.; Bash P. A.; Karplus M. A Combined Quantum Mechanical and Molecular Mechanical Potential for Molecular Dynamics Simulations. J. Comput. Chem. 1990, 11, 700–733. 10.1002/jcc.540110605.

[ref362] Tu Y.; Laaksonen A. Implementing Quantum Mechanics into Molecular Mechanics—Combined QM/MM Modeling Methods. Adv. Quantum Chem. 2010, 59, 1–15. 10.1016/S0065-3276(10)59001-4.

[ref363] Kawamura-Kuribayashi H.; Koga N.; Morokuma K. An Ab Initio MO and MM Study of Homogeneous Olefin Polymerization with Silylene-Bridged Zirconocene Catalyst and Its Regio- and Stereoselectivity. J. Am. Chem. Soc. 1992, 114, 8687–8694. 10.1021/ja00048a049.

[ref364] Dapprich S.; Komáromi I.; Byun K. S.; Morokuma K.; Frisch M. J. A New ONIOM Implementation in Gaussian98. Part I. The Calculation of Energies, Gradients, Vibrational Frequencies and Electric Field Derivatives. J. Mol. Struct. THEOCHEM 1999, 461–462, 1–21. 10.1016/S0166-1280(98)00475-8.

[ref365] Chung L. W.; Sameera W. M. C.; Ramozzi R.; Page A. J.; Hatanaka M.; Petrova G. P.; Harris T. V.; Li X.; Ke Z.; Liu F.; et al. The ONIOM Method and Its Applications. Chem. Rev. 2015, 115, 5678–5796. 10.1021/cr5004419. 25853797

[ref366] Kathiresan R.; Gopalakrishnan S.; Kolandaivel P. Interaction and Bioconjugation of CdSe/ZnS Core/Shell Quantum Dots with Maltose-Binding Protein. Comput. Theor. Chem. 2017, 1101, 96–101. 10.1016/j.comptc.2016.12.038.

[ref367] Groenhof G.; Bouxin-Cademartory M.; Hess B.; de Visser S. P.; Berendsen H. J. C.; Olivucci M.; Mark A. E.; Robb M. A. Photoactivation of the Photoactive Yellow Protein: Why Photon Absorption Triggers a Trans-to-Cis Isomerization of the Chromophore in the Protein. J. Am. Chem. Soc. 2004, 126, 4228–4233. 10.1021/ja039557f. 15053611

[ref368] Ruckenbauer M.; Barbatti M.; Müller T.; Lischka H. Nonadiabatic Excited-State Dynamics with Hybrid Ab Initio Quantum-Mechanical/Molecular-Mechanical Methods: Solvation of the Pentadieniminium Cation in Apolar Media. J. Phys. Chem. A 2010, 114, 6757–6765. 10.1021/jp103101t. 20518515

[ref369] Barbatti M.; Ruckenbauer M.; Plasser F.; Pittner J.; Granucci G.; Persico M.; Lischka H. Newton-X: A Surface-Hopping Program for Nonadiabatic Molecular Dynamics. Wiley Interdiscip. Rev. Comput. Mol. Sci. 2014, 4, 26–33. 10.1002/wcms.1158.

[ref370] Tully J. C.; Preston R. K. Trajectory Surface Hopping Approach to Nonadiabatic Molecular Collisions: The Reaction of H+ with D2. J. Chem. Phys. 2003, 55, 562 10.1063/1.1675788.

[ref371] Tully J. C. Perspective: Nonadiabatic Dynamics Theory. J. Chem. Phys. 2012, 137, 22A301 10.1063/1.4757762. 23249037

[ref372] Wang L.; Akimov A.; Prezhdo O. V. Recent Progress in Surface Hopping: 2011–2015. J. Phys. Chem. Lett. 2016, 7, 2100–2112. 10.1021/acs.jpclett.6b00710. 27171314

[ref373] TR N.; AJ W.; JA B.; AE S.; Y Z.; B N.; S F.-A.; D M.; AE R.; S T. Non-Adiabatic Excited-State Molecular Dynamics: Theory and Applications for Modeling Photophysics in Extended Molecular Materials. Chem. Rev. 2020, 120, 2215–2287. 10.1021/acs.chemrev.9b00447. 32040312

[ref374] Nieman R.; Aquino A. J. A.; Lischka H. Exploration of Graphene Defect Reactivity toward a Hydrogen Radical Utilizing a Preactivated Circumcoronene Model. J. Phys. Chem. A 2021, 125, 1152–1165. 10.1021/acs.jpca.0c09255. 33507752

[ref375] Hoogerbrugge P. J.; Koelman J. M. V. A. Simulating Microscopic Hydrodynamic Phenomena with Dissipative Particle Dynamics. EPL (Europhysics Lett. 1992, 19, 155 10.1209/0295-5075/19/3/001.

[ref376] Español P.; Warren P. Statistical Mechanics of Dissipative Particle Dynamics. EPL (Europhysics Lett. 1995, 30, 191 10.1209/0295-5075/30/4/001.

[ref377] Español P. Hydrodynamics from Dissipative Particle Dynamics. Phys. Rev. E 1995, 52, 1734 10.1103/PhysRevE.52.1734. 9963592

[ref378] Ding H.; Ma Y. Theoretical and Computational Investigations of Nanoparticle-Biomembrane Interactions in Cellular Delivery. Small 2015, 11, 1055–1071. 10.1002/smll.201401943. 25387905

[ref379] Hill T. Statistical Mechanics. Principles and Selected Applications; Dover Publications: New York, 1956.

[ref380] Groot R. D.; Warren P. B. Dissipative Particle Dynamics: Bridging the Gap between Atomistic and Mesoscopic Simulation. J. Chem. Phys. 1997, 107, 4423 10.1063/1.474784.

[ref381] Español P.; Warren P. B. Perspective: Dissipative Particle Dynamics. J. Chem. Phys. 2017, 146, 150901 10.1063/1.4979514. 28433024

[ref382] Zhu Y.-L.; Liu H.; Li Z.-W.; Qian H.-J.; Milano G.; Lu Z.-Y. GALAMOST: GPU-Accelerated Large-Scale Molecular Simulation Toolkit. J. Comput. Chem. 2013, 34, 2197–2211. 10.1002/jcc.23365. 24137668

[ref383] Shillcock J.; Lipowsky R. Visualizing Soft Matter: Mesoscopic Simulations of Membranes, Vesicles and Nanoparticles. Biophys. Rev. Lett. 2007, 02, 33–55. 10.1142/S1793048007000428.

[ref384] Cranford S.; Sen D.; Buehler M. J. Meso-Origami: Folding Multilayer Graphene Sheets. Appl. Phys. Lett. 2009, 95, 123121 10.1063/1.3223783.

[ref385] Cranford S.; Buehler M. J. Twisted and Coiled Ultralong Multilayer Graphene Ribbons. Model. Simul. Mater. Sci. Eng. 2011, 19, 054003 10.1088/0965-0393/19/5/054003.

[ref386] Liu M. Optical Properties of Carbon Dots: A Review. Nanoarchitectonics 2020, 1, 1–12. 10.37256/nat.112020124.1-12.

[ref387] Bao L.; Zhang Z.-L.; Tian Z.-Q.; Zhang L.; Liu C.; Lin Y.; Qi B.; Pang D.-W. Electrochemical Tuning of Luminescent Carbon Nanodots: From Preparation to Luminescence Mechanism. Adv. Mater. 2011, 23, 5801–5806. 10.1002/adma.201102866. 22144369

[ref388] Carbonaro C. M.; Corpino R.; Salis M.; Mocci F.; Thakkar S. V.; Olla C.; Ricci P. C. On the Emission Properties of Carbon Dots: Reviewing Data and Discussing Models. C 2019, 5, 60 10.3390/c5040060.

[ref389] Kwon W.; Rhee S.-W. Facile Synthesis of Graphitic Carbon Quantum Dots with Size Tunability and Uniformity Using Reverse Micelles. Chem. Commun. 2012, 48, 5256–5258. 10.1039/c2cc31687k. 22510781

[ref390] Noun F.; Manioudakis J.; Naccache R. Toward Uniform Optical Properties of Carbon Dots. Part. Part. Syst. Charact. 2020, 37, 2000119 10.1002/ppsc.202000119.

[ref391] Xia C.; Zhu S.; Feng T.; Yang M.; Yang B. Evolution and Synthesis of Carbon Dots: From Carbon Dots to Carbonized Polymer Dots. Adv. Sci. 2019, 6, 1901316 10.1002/advs.201901316. PMC6891914 31832313

[ref392] Yang S.; Li W.; Ye C.; Wang G.; Tian H.; Zhu C.; He P.; Ding G.; Xie X.; Liu Y.; et al. C3N-A 2D Crystalline, Hole-Free, Tunable-Narrow-Bandgap Semiconductor with Ferromagnetic Properties. Adv. Mater. 2017, 29, 1605625 10.1002/adma.201605625. 28240434

[ref393] Ehrat F.; Bhattacharyya S.; Schneider J.; Löf A.; Wyrwich R.; Rogach A. L.; Stolarczyk J. K.; Urban A. S.; Feldmann J. Tracking the Source of Carbon Dot Photoluminescence: Aromatic Domains versus Molecular Fluorophores. Nano Lett. 2017, 17, 7710–7716. 10.1021/acs.nanolett.7b03863. 29188711

[ref394] Demchenko A. P. Excitons in Carbonic Nanostructures. C 2019, 5, 71 10.3390/c5040071.

[ref395] Mura S.; Stagi L.; Ludmerczki R.; Malfatti L.; Innocenzi P. Reversible Aggregation of Molecular-Like Fluorophores Driven by Extreme PH in Carbon Dots. Materials (Basel). 2020, 13, 3654 10.3390/ma13163654. PMC7476021 32824799

[ref396] Stagi L.; Mura S.; Malfatti L.; Carbonaro C. M.; Ricci P. C.; Porcu S.; Secci F.; Innocenzi P. Anomalous Optical Properties of Citrazinic Acid under Extreme PH Conditions. ACS Omega 2020, 5, 10958–10964. 10.1021/acsomega.0c00775. 32455216 PMC7241015

[ref397] Mura S.; Ludmerczki R.; Stagi L.; Garroni S.; Carbonaro C. M.; Ricci P. C.; Casula M. F.; Malfatti L.; Innocenzi P. Integrating Sol-Gel and Carbon Dots Chemistry for the Fabrication of Fluorescent Hybrid Organic-Inorganic Films. Sci. Rep. 2020, 10, 4770 10.1038/s41598-020-61517-x. 32179839 PMC7075866

[ref398] Kasprzyk W.; Świergosz T.; Bednarz S.; Walas K.; Bashmakova N. V.; Bogdał D. Luminescence Phenomena of Carbon Dots Derived from Citric Acid and Urea - a Molecular Insight. Nanoscale 2018, 10, 13889–13894. 10.1039/C8NR03602K. 29999091

[ref399] Qu D.; Sun Z. The Formation Mechanism and Fluorophores of Carbon Dots Synthesized via a Bottom-up Route. Mater. Chem. Front. 2020, 4, 400–420. 10.1039/C9QM00552H.

[ref400] Ding H.; Wei J. S.; Xiong H. M. Nitrogen and Sulfur Co-Doped Carbon Dots with Strong Blue Luminescence. Nanoscale 2014, 6, 13817–13823. 10.1039/C4NR04267K. 25297983

[ref401] Deng X.; Sun J.; Yang S.; Shen H.; Zhou W.; Lu J.; Ding G.; Wang Z. The Emission Wavelength Dependent Photoluminescence Lifetime of the N-Doped Graphene Quantum Dots. Appl. Phys. Lett. 2015, 107, 241905 10.1063/1.4937923.

[ref402] Jiang K.; Sun S.; Zhang L.; Lu Y.; Wu A.; Cai C.; Lin H. Red, Green, and Blue Luminescence by Carbon Dots: Full-Color Emission Tuning and Multicolor Cellular Imaging. Angew. Chemie Int. Ed. 2015, 54, 5360–5363. 10.1002/anie.201501193. 25832292

[ref403] Liang J.; Jiao Y.; Jaroniec M.; Qiao S. Z. Sulfur and Nitrogen Dual-Doped Mesoporous Graphene Electrocatalyst for Oxygen Reduction with Synergistically Enhanced Performance. Angew. Chemie Int. Ed. 2012, 51, 11496–11500. 10.1002/anie.201206720. 23055257

[ref404] Lakowicz J. R. Principles of Fluorescence Spectroscopy; Lakowicz J. R., Ed.; Springer US: Boston, MA, 2006.

[ref405] Ludmerczki R.; Malfatti L.; Stagi L.; Meloni M.; Carbonaro C. M.; Casula M. F.; Bogdán D.; Mura S.; Mándity I. M.; Innocenzi P. Polymerization-Driven Photoluminescence in Alkanolamine-Based C-Dots. Chem. Eur. J. 2021, 27, 2543–2550. 10.1002/chem.202004465. 33196126

[ref406] Tran T.; Prlj A.; Lin K. H.; Hollas D.; Corminboeuf C. Mechanisms of Fluorescence Quenching in Prototypical Aggregation-Induced Emission Systems: Excited State Dynamics with TD-DFTB. Phys. Chem. Chem. Phys. 2019, 21, 9026–9035. 10.1039/C9CP00691E. 30869714

[ref407] Hong Y.; Lam J. W. Y.; Tang B. Z. Aggregation-Induced Emission: Phenomenon, Mechanism and Applications. Chem. Commun. 2009, (29), 4332–4353. 10.1039/b904665h. 19597589

[ref408] Li Q.; Blancafort L. A Conical Intersection Model to Explain Aggregation Induced Emission in Diphenyl Dibenzofulvene. Chem. Commun. 2013, 49, 5966 10.1039/c3cc41730a. 23715286

[ref409] Peng X.-L.; Ruiz-Barragan S.; Li Z.-S.; Li Q.-S.; Blancafort L. Restricted Access to a Conical Intersection to Explain Aggregation Induced Emission in Dimethyl Tetraphenylsilole. J. Mater. Chem. C 2016, 4, 2802–2810. 10.1039/C5TC03322E.

[ref410] Sciortino A.; Gazzetto M.; Soriano M. L.; Cannas M.; Cárdenas S.; Cannizzo A.; Messina F. Ultrafast Spectroscopic Investigation on Fluorescent Carbon Nanodots: The Role of Passivation. Phys. Chem. Chem. Phys. 2019, 21, 16459–16467. 10.1039/C9CP03063H. 31313777

[ref411] Mondal S.; Yucknovsky A.; Akulov K.; Ghorai N.; Schwartz T.; Ghosh H. N.; Amdursky N. Efficient Photosensitizing Capabilities and Ultrafast Carrier Dynamics of Doped Carbon Dots. J. Am. Chem. Soc. 2019, 141, 15413–15422. 10.1021/jacs.9b08071. 31453686

[ref412] Sui L.; Jin W.; Li S.; Liu D.; Jiang Y.; Chen A.; Liu H.; Shi Y.; Ding D.; Jin M. Ultrafast Carrier Dynamics of Carbon Nanodots in Different PH Environments. Phys. Chem. Chem. Phys. 2016, 18, 3838–3845. 10.1039/C5CP07558K. 26763126

[ref413] Ludmerczki R.; Mura S.; Carbonaro C. M.; Mandity I. M.; Carraro M.; Senes N.; Garroni S.; Granozzi G.; Calvillo L.; Marras S.; et al. Carbon Dots from Citric Acid and Its Intermediates Formed by Thermal Decomposition. Chem. - A Eur. J. 2019, 25, 11963–11974. 10.1002/chem.201902497. 31254368

[ref414] Wang W. J.; Hai X.; Mao Q. X.; Chen M. L.; Wang J. H. Polyhedral Oligomeric Silsesquioxane Functionalized Carbon Dots for Cell Imaging. ACS Appl. Mater. Interfaces 2015, 7, 16609–16616. 10.1021/acsami.5b04172. 26171887

[ref415] Malard L. M.; Pimenta M. A.; Dresselhaus G.; Dresselhaus M. S. Raman Spectroscopy in Graphene. Phys. Rep. 2009, 473, 51–87. 10.1016/j.physrep.2009.02.003.

[ref416] Ferrari A. C.; Basko D. M. Raman Spectroscopy as a Versatile Tool for Studying the Properties of Graphene. Nat. Nanotechnol. 2013, 8, 235–246. 10.1038/nnano.2013.46. 23552117

[ref417] Loche D.; Malfatti L.; Carboni D.; Alzari V.; Mariani A.; Casula M. F. Incorporation of Graphene into Silica-Based Aerogels and Application for Water Remediation. RSC Adv. 2016, 6, 66516–66523. 10.1039/C6RA09618B.

[ref418] Yarur Villanueva F.; Manioudakis J.; Naccache R.; Majewski M. B. Carbon Dot-Sensitized Photoanodes for Visible Light-Driven Organic Transformations. ACS Appl. Nano Mater. 2020, 3, 2756–2765. 10.1021/acsanm.0c00094.

[ref419] Stachowska J. D.; Murphy A.; Mellor C.; Fernandes D.; Gibbons E. N.; Krysmann M. J.; Kelarakis A.; Burgaz E.; Moore J.; Yeates S. G. A Rich Gallery of Carbon Dots Based Photoluminescent Suspensions and Powders Derived by Citric Acid/Urea. Sci. Rep. 2021, 11, 10554 10.1038/s41598-021-89984-w. 34006934 PMC8131706

[ref420] Pal A.; Sk M. P.; Chattopadhyay A. Recent Advances in Crystalline Carbon Dots for Superior Application Potential. Mater. Adv. 2020, 1, 525–553. 10.1039/D0MA00108B.

[ref421] Basoglu A.; Ocak Ü.; Gümrükçüoglu A. Synthesis of Microwave-Assisted Fluorescence Carbon Quantum Dots Using Roasted-Chickpeas and Its Applications for Sensitive and Selective Detection of Fe 3+ Ions. J. Fluoresc. 2020, 30, 515 10.1007/s10895-019-02428-7. 32152829

[ref422] Wei X. M.; Xu Y.; Li Y. H.; Yin X. B.; He X. W. Ultrafast Synthesis of Nitrogen-Doped Carbon Dots via Neutralization Heat for Bioimaging and Sensing Applications. RSC Adv. 2014, 4, 44504–44508. 10.1039/C4RA08523J.

[ref423] Wang W.; Damm C.; Walter J.; Nacken T. J.; Peukert W. Photobleaching and Stabilization of Carbon Nanodots Produced by Solvothermal Synthesis. Phys. Chem. Chem. Phys. 2016, 18, 466–475. 10.1039/C5CP04942C. 26616577

[ref424] Morgenstern M.; Freitag N.; Nent A.; Nemes-Incze P.; Liebmann M. Graphene Quantum Dots Probed by Scanning Tunneling Microscopy. Ann. Phys. 2017, 529, 1700018 10.1002/andp.201700018.

[ref425] Zhai X.; Zhang P.; Liu C.; Bai T.; Li W.; Dai L.; Liu W. Highly Luminescent Carbon Nanodots by Microwave-Assisted Pyrolysis. Chem. Commun. 2012, 48, 7955–7957. 10.1039/c2cc33869f. 22763501

[ref426] Varisco M.; Zufferey D.; Ruggi A.; Zhang Y.; Erni R.; Mamula O. Synthesis of Hydrophilic and Hydrophobic Carbon Quantum Dots from Waste of Wine Fermentation. R. Soc. Open Sci. 2017, 4, 170900 10.1098/rsos.170900. 29308232 PMC5749999

[ref427] Yuan B.; Xie Z.; Chen P.; Zhou S. Highly Efficient Carbon Dots and Their Nanohybrids for Trichromatic White LEDs. J. Mater. Chem. C 2018, 6, 5957–5963. 10.1039/C8TC01659C.

[ref428] Dager A.; Baliyan A.; Kurosu S.; Maekawa T.; Tachibana M. Ultrafast Synthesis of Carbon Quantum Dots from Fenugreek Seeds Using Microwave Plasma Enhanced Decomposition: Application of C-QDs to Grow Fluorescent Protein Crystals. Sci. Rep. 2020, 10, 12333 10.1038/s41598-020-69264-9. 32704038 PMC7378176

[ref429] Dutta Chowdhury A.; Doong R. A. Highly Sensitive and Selective Detection of Nanomolar Ferric Ions Using Dopamine Functionalized Graphene Quantum Dots. ACS Appl. Mater. Interfaces 2016, 8, 21002–21010. 10.1021/acsami.6b06266. 27472083

[ref430] Papaioannou N.; Marinovic A.; Yoshizawa N.; Goode A. E.; Fay M.; Khlobystov A.; Titirici M. M.; Sapelkin A. Structure and Solvents Effects on the Optical Properties of Sugar-Derived Carbon Nanodots. Sci. Rep. 2018, 8, 6559 10.1038/s41598-018-25012-8. 29700398 PMC5920085

[ref431] Hu Q.; Gong X.; Liu L.; Choi M. M. F. Characterization and Analytical Separation of Fluorescent Carbon Nanodots. J. Nanomater. 2017, 2017, 1–23. 10.1155/2017/1804178.

[ref432] Liu R.; Wu D.; Liu S.; Koynov K.; Knoll W.; Li Q. An Aqueous Route to Multicolor Photoluminescent Carbon Dots Using Silica Spheres as Carriers. Angew. Chemie - Int. Ed. 2009, 48, 4598–4601. 10.1002/anie.200900652. 19388019

[ref433] Patir K.; Gogoi S. K. Nitrogen-Doped Carbon Dots as Fluorescence ON-OFF-ON Sensor for Parallel Detection of Copper(Ii) and Mercury(Ii) Ions in Solutions as Well as in Filter Paper-Based Microfluidic Device. Nanoscale Adv. 2019, 1, 592–601. 10.1039/C8NA00080H. PMC9473229 36132272

[ref434] Mintz K. J.; Bartoli M.; Rovere M.; Zhou Y.; Hettiarachchi S. D.; Paudyal S.; Chen J.; Domena J. B.; Liyanage P. Y.; Sampson R.; et al. A Deep Investigation into the Structure of Carbon Dots. Carbon N. Y. 2021, 173, 433–447. 10.1016/j.carbon.2020.11.017.

[ref435] Isaacs M. A.; Davies-Jones J.; Davies P. R.; Guan S.; Lee R.; Morgan D. J.; Palgrave R. Advanced XPS Characterization: XPS-Based Multi-Technique Analyses for Comprehensive Understanding of Functional Materials. Mater. Chem. Front. 2021, 5, 7931–7963. 10.1039/D1QM00969A.

[ref436] Yang H.; Liu Y.; Guo Z.; Lei B.; Zhuang J.; Zhang X.; Liu Z.; Hu C. Hydrophobic Carbon Dots with Blue Dispersed Emission and Red Aggregation-Induced Emission. Nat. Commun. 2019, 10, 1789 10.1038/s41467-019-09830-6. 30996272 PMC6470214

[ref437] Wang L.; Li W.; Yin L.; Liu Y.; Guo H.; Lai J.; Han Y.; Li G.; Li M.; Zhang J. Full-Color Fluorescent Carbon Quantum Dots. Sci. Adv. 2020, 6, eabb6772 10.1126/sciadv.abb6772. 33008913 PMC7852397

[ref438] Duan P.; Zhi B.; Coburn L.; Haynes C. L.; Schmidt-Rohr K. A Molecular Fluorophore in Citric Acid/Ethylenediamine Carbon Dots Identified and Quantified by Multinuclear Solid-State Nuclear Magnetic Resonance. Magn. Reson. Chem. 2020, 58, 1130 10.1002/mrc.4985. 31880813

[ref439] Hinterberger V.; Damm C.; Haines P.; Guldi D. M.; Peukert W. Purification and Structural Elucidation of Carbon Dots by Column Chromatography. Nanoscale 2019, 11, 8464 10.1039/C9NR01029G. 30990494

[ref440] Dorđevic L.; Arcudi F.; Prato M. Preparation, Functionalization and Characterization of Engineered Carbon Nanodots. Nat. Protoc. 2019, 14, 2931–2953. 10.1038/s41596-019-0207-x. 31534230

[ref441] Mauro N.; Utzeri M. A.; Buscarino G.; Sciortino A.; Messina F.; Cavallaro G.; Giammona G. Pressure-Dependent Tuning of Photoluminescence and Size Distribution of Carbon Nanodots for Theranostic Anticancer Applications. Materials 2020, 13, 4899 10.3390/ma13214899. PMC7662895 33142826

[ref442] Liu L.; Xu Z. Study of Chromatographic Fractions from Carbon Dots Isolated by Column Chromatography and a Binary Gradient Elution: Via RP-HPLC. Anal. Methods 2019, 11, 760 10.1039/C8AY02660B.

[ref443] Jiang K.; Feng X.; Gao X.; Wang Y.; Cai C.; Li Z.; Lin H. Preparation of Multicolor Photoluminescent Carbon Dots by Tuning Surface States. Nanomaterials 2019, 9, 529 10.3390/nano9040529. PMC6523770 30987120

[ref444] Baker S. N.; Baker G. A. Luminescent Carbon Nanodots: Emergent Nanolights. Angew. Chemie - Int. Ed. 2010, 49, 6726–6744. 10.1002/anie.200906623. 20687055

[ref445] Castiglioni C.; Mapelli C.; Negri F.; Zerbi G. Origin of the D Line in the Raman Spectrum of Graphite: A Study Based on Raman Frequencies and Intensities of Polycyclic Aromatic Hydrocarbon Molecules. J. Chem. Phys. 2001, 114, 963–974. 10.1063/1.1329670.

[ref446] Schneider J.; Reckmeier C. J.; Xiong Y.; von Seckendorff M.; Susha A. S.; Kasák P.; Rogach A. L. Molecular Fluorescence in Citric Acid-Based Carbon Dots. J. Phys. Chem. C 2017, 121, 2014–2022. 10.1021/acs.jpcc.6b12519.

[ref447] Humphrey W.; Dalke A.; Schulten K. VMD: Visual Molecular Dynamics. J. Mol. Graph. 1996, 14, 33–38. 10.1016/0263-7855(96)00018-5. 8744570

[ref448] Wolski P. Molecular Dynamics Simulations of the PH-Dependent Adsorption of Doxorubicin on Carbon Quantum Dots. Mol. Pharmaceutics 2021, 18, 257–266. 10.1021/acs.molpharmaceut.0c00895. 33325232

[ref449] Merchant A. R.; McCulloch D. G.; McKenzie D. R.; Yin Y.; Hall L.; Gerstner E. G. Structural Investigation of Two Carbon Nitride Solids Produced by Cathodic Arc Deposition and Nitrogen Implantation. J. Appl. Phys. 1996, 79, 6914–6919. 10.1063/1.361515.

[ref450] Song Y.; Zhu S.; Zhang S.; Fu Y.; Wang L.; Zhao X.; Yang B. Investigation from Chemical Structure to Photoluminescent Mechanism: A Type of Carbon Dots from the Pyrolysis of Citric Acid and an Amine. J. Mater. Chem. C 2015, 3, 5976–5984. 10.1039/C5TC00813A.

[ref451] McCulloch D. G.; McKenzie D. R.; Goringe C. M. Ab Initio Simulations of the Structure of Amorphous Carbon. Phys. Rev. B 2000, 61, 2349–2355. 10.1103/PhysRevB.61.2349.

[ref452] Fanchini G.; Tagliaferro A. Localisation and Density of States in Amorphous Carbon-Based Alloys. Diam. Relat. Mater. 2001, 10, 191–199. 10.1016/S0925-9635(00)00467-2.

[ref453] Fanchini G.; Ray S. C.; Tagliaferro A. Density of Electronic States in Amorphous Carbons. Diam. Relat. Mater. 2003, 12, 891–899. 10.1016/S0925-9635(02)00376-X.

[ref454] Ding H.; Yu S. B.; Wei J. S.; Xiong H. M. Full-Color Light-Emitting Carbon Dots with a Surface-State-Controlled Luminescence Mechanism. ACS Nano 2016, 10, 484–491. 10.1021/acsnano.5b05406. 26646584

[ref455] Dans P. D.; Walther J.; Gómez H.; Orozco M. Multiscale Simulation of DNA. Curr. Opin. Struct. Biol. 2016, 37, 29–45. 10.1016/j.sbi.2015.11.011. 26708341

[ref456] Reckmeier C. J.; Schneider J.; Xiong Y.; Häusler J.; Kasák P.; Schnick W.; Rogach A. L. Aggregated Molecular Fluorophores in the Ammonothermal Synthesis of Carbon Dots. Chem. Mater. 2017, 29, 10352–10361. 10.1021/acs.chemmater.7b03344.

[ref457] Montgomery N. A.; Denis J. C.; Schumacher S.; Ruseckas A.; Skabara P. J.; Kanibolotsky A.; Paterson M. J.; Galbraith I.; Turnbull G. A.; Samuel I. D. W. Optical Excitations in Star-Shaped Fluorene Molecules. J. Phys. Chem. A 2011, 115, 2913 10.1021/jp1109042. 21428384

[ref458] Li H.; He X.; Kang Z.; Huang H.; Liu Y.; Liu J.; Lian S.; Tsang C. H. A.; Yang X.; Lee S. T. Water-Soluble Fluorescent Carbon Quantum Dots and Photocatalyst Design. Angew. Chem. Int. Ed. 2010, 49, 4430 10.1002/anie.200906154. 20461744

[ref459] Lu J.; Yang J. X.; Wang J.; Lim A.; Wang S.; Loh K. P. One-Pot Synthesis of Fluorescent Carbon Nanoribbons, Nanoparticles, and Graphene by the Exfoliation of Graphite in Ionic Liquids. ACS Nano 2009, 3, 2367 10.1021/nn900546b. 19702326

[ref460] Gaponenko S. V. Optical Properties of Semiconductor Nanocrystals; Cambridge University Press, 1998.

[ref461] Kumar A.; Sharma K.; Dixit A. R. A Review on the Mechanical and Thermal Properties of Graphene and Graphene-Based Polymer Nanocomposites: Understanding of Modelling and MD Simulation. Mol. Simul. 2020, 46, 136–154. 10.1080/08927022.2019.1680844.

[ref462] Swift P. D. Ph.D. Thesis, Spectroscopic Investigations of the Cathode Spot by Fizeau Interferometry, University of Sydney, Sydney, Australia, 1989.

[ref463] Fallon P. J.; Veerasamy V. S.; Davis C. A.; Robertson J.; Amaratunga G. A. J.; Milne W. I.; Koskinen J. Properties of Filtered-Ion-Beam-Deposited Diamondlike Carbon as a Function of Ion Energy. Phys. Rev. B 1993, 48, 4777–4782. 10.1103/PhysRevB.48.4777. 10008965

[ref464] Schwan J.; Ulrich S.; Theel T.; Roth H.; Ehrhardt H.; Becker P.; Silva S. R. P. Stress-Induced Formation of High-Density Amorphous Carbon Thin Films. J. Appl. Phys. 1997, 82, 6024–6030. 10.1063/1.366469.

[ref465] Stephan U.; Frauenheim T.; Blaudeck P.; Jungnickel G. π Bonding versus Electronic-Defect Generation: An Examination of Band-Gap Properties in Amorphous Carbon. Phys. Rev. B 1994, 50, 1489–1501. 10.1103/PhysRevB.50.1489. 9976331

[ref466] Kang S.; Jung K. H.; Mhin S.; Son Y.; Lee K.; Kim W. R.; Choi H.; Ryu J. H.; Han H.; Kim K. M. Fundamental Understanding of the Formation Mechanism for Graphene Quantum Dots Fabricated by Pulsed Laser Fragmentation in Liquid: Experimental and Theoretical Insight. Small 2020, 16, 2003538 10.1002/smll.202003538. 32830432

[ref467] Cayuela A.; Soriano M. L.; Carrillo-Carrión C.; Valcárcel M. Semiconductor and Carbon-Based Fluorescent Nanodots: The Need for Consistency. Chem. Commun. 2016, 52, 1311–1326. 10.1039/C5CC07754K. 26671042

[ref468] Venugopal G.; Krishnamoorthy K.; Mohan R.; Kim S. J. An Investigation of the Electrical Transport Properties of Graphene-Oxide Thin Films. Mater. Chem. Phys. 2012, 132, 29–33. 10.1016/j.matchemphys.2011.10.040.

[ref469] Li L.; Wu G.; Yang G.; Peng J.; Zhao J.; Zhu J. J. Focusing on Luminescent Graphene Quantum Dots: Current Status and Future Perspectives. Nanoscale 2013, 5, 4015–4039. 10.1039/C3NR33849E. 23579482

[ref470] Luo Z.; Lu Y.; Somers L. A.; Johnson A. T. C. High Yield Preparation of Macroscopic Graphene Oxide Membranes. J. Am. Chem. Soc. 2009, 131, 898–899. 10.1021/ja807934n. 19128004

[ref471] Kozawa D.; Miyauchi Y.; Mouri S.; Matsuda K. Exploring the Origin of Blue and Ultraviolet Fluorescence in Graphene Oxide. J. Phys. Chem. Lett. 2013, 4, 2035–2040. 10.1021/jz400930f. 26283249

[ref472] Wang S.; Chen Z. G.; Cole I.; Li Q. Structural Evolution of Graphene Quantum Dots during Thermal Decomposition of Citric Acid and the Corresponding Photoluminescence. Carbon N. Y. 2015, 82, 304–313. 10.1016/j.carbon.2014.10.075.

[ref473] Bhattacharyya S.; Ehrat F.; Urban P.; Teves R.; Wyrwich R.; Döblinger M.; Feldmann J.; Urban A. S.; Stolarczyk J. K. Effect of Nitrogen Atom Positioning on the Trade-off between Emissive and Photocatalytic Properties of Carbon Dots. Nat. Commun. 2017, 8, 1401 10.1038/s41467-017-01463-x. 29123091 PMC5680170

[ref474] Xiao L.; Wang Y.; Huang Y.; Wong T.; Sun H. Self-Trapped Exciton Emission from Carbon Dots Investigated by Polarization Anisotropy of Photoluminescence and Photoexcitation. Nanoscale 2017, 9, 12637–12646. 10.1039/C7NR03913A. 28825435

[ref475] Zhang R. Q.; Bertran E.; Lee S. T. Size Dependence of Energy Gaps in Small Carbon Clusters: The Origin of Broadband Luminescence. Diam. Relat. Mater. 1998, 7, 1663–1668. 10.1016/S0925-9635(98)00240-4.

[ref476] Scholes G. D.; Rumbles G. Excitons in Nanoscale Systems. Nat. Mater. 2006, 5, 683–696. 10.1038/nmat1710. 16946728

[ref477] Li X.; Rui M.; Song J.; Shen Z.; Zeng H. Carbon and Graphene Quantum Dots for Optoelectronic and Energy Devices: A Review. Adv. Funct. Mater. 2015, 25, 4929–4947. 10.1002/adfm.201501250.

[ref478] Pan D.; Zhang J.; Li Z.; Wu M. Hydrothermal Route for Cutting Graphene Sheets into Blue-Luminescent Graphene Quantum Dots. Adv. Mater. 2010, 22, 734–738. 10.1002/adma.200902825. 20217780

[ref479] Sharma A.; Gadly T.; Gupta A.; Ballal A.; Ghosh S. K.; Kumbhakar M. Origin of Excitation Dependent Fluorescence in Carbon Nanodots. J. Phys. Chem. Lett. 2016, 7, 3695–3702. 10.1021/acs.jpclett.6b01791. 27588560

[ref480] Chen S.; Ullah N.; Wang T.; Zhang R. Tuning the Optical Properties of Graphene Quantum Dots by Selective Oxidation: A Theoretical Perspective. J. Mater. Chem. C 2018, 6, 6875–6883. 10.1039/C8TC02083C. 32254380

[ref481] Song K. S.; Williams R. T. Investigation of Self-Trapped Excitons from a Defect Perspective; Springer: Berlin, Heidelberg, Germany, 1996; pp 32–65.

[ref482] Li L.-L.; Ji J.; Fei R.; Wang C.-Z.; Lu Q.; Zhang J.-R.; Jiang L.-P.; Zhu J.-J. A Facile Microwave Avenue to Electrochemiluminescent Two-Color Graphene Quantum Dots. Adv. Funct. Mater. 2012, 22, 2971–2979. 10.1002/adfm.201200166.

[ref483] Kilina S.; Kilin D.; Tretiak S. Light-Driven and Phonon-Assisted Dynamics in Organic and Semiconductor Nanostructures. Chem. Rev. 2015, 115, 5929–5978. 10.1021/acs.chemrev.5b00012. 25993511

[ref484] Kang B.; Choi Y.; Kim B. S.; Youn I. S.; Lee G. Orbital Hybridization Mechanism for the Enhanced Photoluminescence in Edge-Functionalized Sp2 Carbon Clusters. Carbon N. Y. 2016, 109, 418–427. 10.1016/j.carbon.2016.06.007.

[ref485] Cocchi C.; Prezzi D.; Ruini A.; Caldas M. J.; Molinari E. Electronics and Optics of Graphene Nanoflakes: Edge Functionalization and Structural Distortions. J. Phys. Chem. C 2012, 116, 17328–17335. 10.1021/jp300657k.

[ref486] Mak K. F.; Da Jornada F. H.; He K.; Deslippe J.; Petrone N.; Hone J.; Shan J.; Louie S. G.; Heinz T. F. Tuning Many-Body Interactions in Graphene: The Effects of Doping on Excitons and Carrier Lifetimes. Phys. Rev. Lett. 2014, 112, 207401 10.1103/PhysRevLett.112.207401.

[ref487] Yang L. Excitonic Effects on Optical Absorption Spectra of Doped Graphene. Nano Lett. 2011, 11, 3844–3847. 10.1021/nl201928g. 21861511

[ref488] Matsoso B. J.; Ranganathan K.; Mutuma B. K.; Lerotholi T.; Jones G.; Coville N. J. Time-Dependent Evolution of the Nitrogen Configurations in N-Doped Graphene Films. RSC Adv. 2016, 6, 106914–106920. 10.1039/C6RA24094A.

[ref489] Zhou Z.; Shen Y.; Li Y.; Liu A.; Liu S.; Zhang Y. Chemical Cleavage of Layered Carbon Nitride with Enhanced Photoluminescent Performances and Photoconduction. ACS Nano 2015, 9, 12480–12487. 10.1021/acsnano.5b05924. 26502265

[ref490] Li Y.; Zhang H.; Liu P.; Wang D.; Li Y.; Zhao H. Cross-Linked g-C3N4/RGO Nanocomposites with Tunable Band Structure and Enhanced Visible Light Photocatalytic Activity. Small 2013, 9, 3336–3344. 10.1002/smll.201203135. 23630157

[ref491] Tong Z.; Yang D.; Shi J.; Nan Y.; Sun Y.; Jiang Z. Three-Dimensional Porous Aerogel Constructed by g-C3N4 and Graphene Oxide Nanosheets with Excellent Visible-Light Photocatalytic Performance. ACS Appl. Mater. Interfaces 2015, 7, 25693–25701. 10.1021/acsami.5b09503. 26545166

[ref492] Ong W. J.; Tan L. L.; Ng Y. H.; Yong S. T.; Chai S. P. Graphitic Carbon Nitride (g-C3N4)-Based Photocatalysts for Artificial Photosynthesis and Environmental Remediation: Are We a Step Closer to Achieving Sustainability?. Chem. Rev. 2016, 116, 7159–7329. 10.1021/acs.chemrev.6b00075. 27199146

[ref493] Stagi L.; Chiriu D.; Carbonaro C. M.; Corpino R.; Ricci P. C. Structural and Optical Properties of Carbon Nitride Polymorphs. Diam. Relat. Mater. 2016, 68, 84–92. 10.1016/j.diamond.2016.06.009.

[ref494] Chen S.; Ullah N.; Zhang R. Engineering the Excited State of Graphitic Carbon Nitride Nanostructures by Covalently Bonding with Graphene Quantum Dots. Theor. Chem. Acc. 2020, 139, 20 10.1007/s00214-019-2525-z.

[ref495] Zhou Z.; Niu X.; Ma L.; Wang J. Revealing the PH-Dependent Photoluminescence Mechanism of Graphitic C3N4 Quantum Dots. Adv. Theory Simulations 2019, 2, 1900074 10.1002/adts.201900074.

[ref496] Mocci P.; Cardia R.; Cappellini G. Inclusions of Si-Atoms in Graphene Nanostructures: A Computational Study on the Ground-State Electronic Properties of Coronene and Ovalene. J. Phys. Conf. Ser. 2018, 956, 012020 10.1088/1742-6596/956/1/012020.

[ref497] Cruz-Silva E.; Lopez-Urias F.; Munoz-Sandoval E.; Sumpter B. G.; Terrones H.; Charlier J.-C.; Meunier V.; Terrones M. Electronic Transport and Mechanical Properties of Phosphorus- and Phosphorus-Nitrogen-Doped Carbon Nanotubes. ACS Nano 2009, 3, 1913–1921. 10.1021/nn900286h. 19572616

[ref498] Cui P. Effect of Boron and Nitrogen Doping on Carrier Relaxation Dynamics of Graphene Quantum Dots. Mater. Res. Express 2018, 5, 065034 10.1088/2053-1591/aacadb.

[ref499] Su R.; Guan Q.; Cai W.; Yang W.; Xu Q.; Guo Y.; Zhang L.; Fei L.; Xu M. Multi-Color Carbon Dots for White Light-Emitting Diodes. RSC Adv. 2019, 9, 9700–9708. 10.1039/C8RA09868A. 35520699 PMC9062395

[ref500] Li M.; Cushing S. K.; Zhou X.; Guo S.; Wu N. Fingerprinting Photoluminescence of Functional Groups in Graphene Oxide. J. Mater. Chem. 2012, 22, 23374–23379. 10.1039/c2jm35417a.

[ref501] Yeh T. F.; Huang W. L.; Chung C. J.; Chiang I. T.; Chen L. C.; Chang H. Y.; Su W. C.; Cheng C.; Chen S. J.; Teng H. Elucidating Quantum Confinement in Graphene Oxide Dots Based on Excitation-Wavelength-Independent Photoluminescence. J. Phys. Chem. Lett. 2016, 7, 2087–2092. 10.1021/acs.jpclett.6b00752. 27192445

[ref502] Zhu S.; Zhao X.; Song Y.; Lu S.; Yang B. Beyond Bottom-up Carbon Nanodots: Citric-Acid Derived Organic Molecules. Nano Today 2016, 11, 128–132. 10.1016/j.nantod.2015.09.002.

[ref503] Maseras F.; Morokuma K. IMOMM: A New Integrated Ab Initio + Molecular Mechanics Geometry Optimization Scheme of Equilibrium Structures and Transition States. J. Comput. Chem. 1995, 16, 1170–1179. 10.1002/jcc.540160911.

[ref504] Svensson M.; Humbel S.; Froese R. D. J.; Matsubara T.; Sieber S.; Morokuma K. ONIOM: A Multilayered Integrated MO+ MM Method for Geometry Optimizations and Single Point Energy Predictions. A Test for Diels- Alder Reactions and Pt (P (t-Bu) 3) 2+ H2 Oxidative Addition. J. Phys. Chem. 1996, 100, 19357–19363. 10.1021/jp962071j.

[ref505] Morokuma K. ONIOM and Its Applications to Material Chemistry and Catalyses. Bull. Korean Chem. Soc. 2003, 24, 797–801. 10.5012/bkcs.2003.24.6.797.

[ref506] Pal T.; Mohiyuddin S.; Packirisamy G. Facile and Green Synthesis of Multicolor Fluorescence Carbon Dots from Curcumin: In Vitro and in Vivo Bioimaging and Other Applications. ACS Omega 2018, 3, 831–843. 10.1021/acsomega.7b01323. 30023790 PMC6044881

[ref507] Liang Y.; Xu L.; Tang K.; Guan Y.; Wang T.; Wang H.; Yu W. W. Nitrogen-Doped Carbon Dots Used as an “on-off-on” Fluorescent Sensor for Fe3+ and Glutathione Detection. Dye. Pigment. 2020, 178, 108358 10.1016/j.dyepig.2020.108358.

[ref508] Kwon W.; Do S.; Won D. C.; Rhee S.-W. Carbon Quantum Dot-Based Field-Effect Transistors and Their Ligand Length-Dependent Carrier Mobility. ACS Appl. Mater. Interfaces 2013, 5, 822–827. 10.1021/am3023898. 23323938

[ref509] Thongsai N.; Tanawannapong N.; Praneerad J.; Kladsomboon S.; Jaiyong P.; Paoprasert P. Real-Time Detection of Alcohol Vapors and Volatile Organic Compounds via Optical Electronic Nose Using Carbon Dots Prepared from Rice Husk and Density Functional Theory Calculation. Colloids Surfaces A Physicochem. Eng. Asp. 2019, 560, 278–287. 10.1016/j.colsurfa.2018.09.077.

[ref510] Seyed-Talebi S. M.; Beheshtian J.; Neek-Amal M. Doping Effect on the Adsorption of NH3Molecule onto Graphene Quantum Dot: From the Physisorption to the Chemisorption. J. Appl. Phys. 2013, 114, 124307 10.1063/1.4822165.

[ref511] Pongsavee M. Effect of Borax on Immune Cell Proliferation and Sister Chromatid Exchange in Human Chromosomes. J. Occup. Med. Toxicol. 2009, 4, 27 10.1186/1745-6673-4-27. 19878537 PMC2776007

[ref512] Park H.; Kim H. Il; Moon G. H.; Choi W. Photoinduced Charge Transfer Processes in Solar Photocatalysis Based on Modified TiO2. Energy Environ. Sci. 2016, 9, 411–433. 10.1039/C5EE02575C.

[ref513] Tharmaraj V.; Pitchumani K. D-Glucose Sensing by (E)-(4-((Pyren-1-Ylmethylene)Amino)Phenyl) Boronic Acid via a Photoinduced Electron Transfer (PET) Mechanism. RSC Adv. 2013, 3, 11566–11570. 10.1039/c3ra40544c.

[ref514] Beddoes C. M.; Case C. P.; Briscoe W. H. Understanding Nanoparticle Cellular Entry: A Physicochemical Perspective. Adv. Colloid Interface Sci. 2015, 218, 48–68. 10.1016/j.cis.2015.01.007. 25708746

[ref515] Wang L.; Wang Y.; Xu T.; Liao H.; Yao C.; Liu Y.; Li Z.; Chen Z.; Pan D.; Sun L. Gram-Scale Synthesis of Single-Crystalline Graphene Quantum Dots with Superior Optical Properties. Nat. Commun. 2014, 5, 5357 10.1038/ncomms6357. 25348348

[ref516] Benz R. W.; Castro-Román F.; Tobias D. J.; White S. H. Experimental Validation of Molecular Dynamics Simulations of Lipid Bilayers: A New Approach. Biophys. J. 2005, 88, 805–817. 10.1529/biophysj.104.046821. 15533925 PMC1305157

[ref517] Marrink S. J.; Berendsen H. J. C. Permeation Process of Small Molecules across Lipid Membranes Studied by Molecular Dynamics Simulations. J. Phys. Chem. 1996, 100, 16729–16738. 10.1021/jp952956f.

[ref518] Tieleman D. P.; Marrink S. J.; Berendsen H. J. C. A Computer Perspective of Membranes: Molecular Dynamics Studies of Lipid Bilayer Systems. Biochim. Biophys. Acta - Rev. Biomembr. 1997, 1331, 235–270. 10.1016/S0304-4157(97)00008-7. 9512654

[ref519] Erimban S.; Daschakraborty S. Compatibility of Advanced Water Models with a United Atom Model of Lipid in Lipid Bilayer Simulation. J. Chem. Phys. 2019, 151, 065104 10.1063/1.5108830.

[ref520] Nagle J. F.; Mathai J. C.; Zeidel M. L.; Tristram-Nagle S. Theory of Passive Permeation through Lipid Bilayers. J. Gen. Physiol. 2008, 131, 77–85. 10.1085/jgp.200709849. 18166627 PMC2174158

[ref521] Song B.; Yuan H.; Jameson C. J.; Murad S. Role of Surface Ligands in Nanoparticle Permeation through a Model Membrane: A Coarse-Grained Molecular Dynamics Simulations Study. Mol. Phys. 2012, 110, 2181–2195. 10.1080/00268976.2012.668964.

[ref522] Parisio G.; Stocchero M.; Ferrarini A. Passive Membrane Permeability: Beyond the Standard Solubility-Diffusion Model. J. Chem. Theory Comput. 2013, 9, 5236–5246. 10.1021/ct400690t. 26592263

[ref523] Xiang T.-X.; Anderson B. D. Permeability of Acetic Acid Across Gel and Liquid-Crystalline Lipid Bilayers Conforms to Free-Surface-Area Theory. Biophys. J. 1997, 72, 223–237. 10.1016/S0006-3495(97)78661-2. 8994607 PMC1184311

[ref524] Xiang T.-X.; Anderson B. D. Influence of Chain Ordering on the Selectivity of Dipalmitoylphosphatidylcholine Bilayer Membranes for Permeant Size and Shape. Biophys. J. 1998, 75, 2658–2671. 10.1016/S0006-3495(98)77711-2. 9826590 PMC1299941

[ref525] Ganazzoli F.; Raffaini G. Classical Atomistic Simulations of Protein Adsorption on Carbon Nanomaterials. Curr. Opin. Colloid Interface Sci. 2019, 41, 11–26. 10.1016/j.cocis.2018.11.008.

[ref526] Li C. Q.; Liu X. Y.; Li S. L.; Jiang P.; Jiang F. L.; Liu Y. High-Oxygen-Content Carbon Dots as a High-Efficiency Inhibitor of Human Insulin Aggregation. ACS Appl. Bio Mater. 2019, 2, 4067–4076. 10.1021/acsabm.9b00583. 35021340

[ref527] Wang M.; Sun Y.; Cao X.; Peng G.; Javed I.; Kakinen A.; Davis T. P.; Lin S.; Liu J.; Ding F.; et al. Graphene Quantum Dots against Human IAPP Aggregation and Toxicity: In Vivo. Nanoscale 2018, 10, 19995–20006. 10.1039/C8NR07180B. 30350837 PMC6212334

[ref528] Yin S.; Biedermannova L.; Vondrasek J.; Dokholyan N. V. MedusaScore: An Accurate Force Field-Based Scoring Function for Virtual Drug Screening. J. Chem. Inf. Model. 2008, 48, 1656–1662. 10.1021/ci8001167. 18672869 PMC2665000

[ref529] Faridi A.; Sun Y.; Mortimer M.; Aranha R. R.; Nandakumar A.; Li Y.; Javed I.; Kakinen A.; Fan Q.; Purcell A. W.; et al. Graphene Quantum Dots Rescue Protein Dysregulation of Pancreatic β-Cells Exposed to Human Islet Amyloid Polypeptide. Nano Res. 2019, 12, 2827–2834. 10.1007/s12274-019-2520-7. 31695851 PMC6834229

[ref530] Kim D.; Yoo J. M.; Hwang H.; Lee J.; Lee S. H.; Yun S. P.; Park M. J.; Lee M. J.; Choi S.; Kwon S. H.; et al. Graphene Quantum Dots Prevent α-Synucleinopathy in Parkinson’s Disease. Nat. Nanotechnol. 2018, 13, 812–818. 10.1038/s41565-018-0179-y. 29988049 PMC6351226

[ref531] Carneiro Cruz A. A.; Freire R. M.; Froelich D. B.; Alves de Lima A. C.; Muniz A. R.; Ferreira O. P.; Fechine P. B. A. Fluorescence Based Platform to Discriminate Protein Using Carbon Quantum Dots. ChemistrySelect 2019, 4, 5619–5627. 10.1002/slct.201901014.

[ref532] McGaughey G. B.; Gagné M.; Rappé A. K. π-Stacking Interactions. Alive and Well in Proteins. J. Biol. Chem. 1998, 273, 15458–15463. 10.1074/jbc.273.25.15458. 9624131

[ref533] Cohen-Tanugi D.; Lin L.-C.; Grossman J. C. Multilayer Nanoporous Graphene Membranes for Water Desalination. Nano Lett. 2016, 16, 1027–1033. 10.1021/acs.nanolett.5b04089. 26806020

[ref534] Wu Q.; Gao J.; Chen L.; Dong S.; Li H.; Qiu H.; Zhao L. Graphene Quantum Dots Functionalized β-Cyclodextrin and Cellulose Chiral Stationary Phases with Enhanced Enantioseparation Performance. J. Chromatogr. A 2019, 1600, 209–218. 10.1016/j.chroma.2019.04.053. 31047665

[ref535] Li X.; Yao X.; Xiao Y.; Wang Y. Enantioseparation of Single Layer Native Cyclodextrin Chiral Stationary Phases: Effect of Cyclodextrin Orientation and a Modeling Study. Anal. Chim. Acta 2017, 990, 174–184. 10.1016/j.aca.2017.07.033. 29029741

[ref536] Liu Z.; Du Y.; Feng Z. Enantioseparation of Drugs by Capillary Electrochromatography Using a Stationary Phase Covalently Modified with Graphene Oxide. Microchim. Acta 2017, 184, 583–593. 10.1007/s00604-016-2014-1.

[ref537] Ge J.; Lan M.; Zhou B.; Liu W.; Guo L.; Wang H.; Jia Q.; Niu G.; Huang X.; Zhou H.; et al. A Graphene Quantum Dot Photodynamic Therapy Agent with High Singlet Oxygen Generation. Nat. Commun. 2014, 5, 4596 10.1038/ncomms5596. 25105845 PMC4143951

[ref538] Liu J.; Wang N.; Yu Y.; Yan Y.; Zhang H.; Li J.; Yu J. Carbon Dots in Zeolites: A New Class of Thermally Activated Delayed Fluorescence Materials with Ultralong Lifetimes. Sci. Adv. 2017, 3, e1603171 10.1126/sciadv.1603171. 28560347 PMC5446214

[ref539] Liu J.; Zhang H.; Wang N.; Yu Y.; Cui Y.; Li J.; Yu J. Template-Modulated Afterglow of Carbon Dots in Zeolites: Room-Temperature Phosphorescence and Thermally Activated Delayed Fluorescence. ACS Mater. Lett. 2019, 1, 58–63. 10.1021/acsmaterialslett.9b00073.

[ref540] He Y.; He J.; Wang L.; Yu Z.; Zhang H.; Liu Y.; Lei B. Synthesis of Double Carbon Dots Co-Doped Mesoporous Al2O3 for Ratiometric Fluorescent Determination of Oxygen. Sensors Actuators B Chem. 2017, 251, 918–926. 10.1016/j.snb.2017.05.104.

[ref541] Joseph J.; Anappara A. A. Cool White, Persistent Room-Temperature Phosphorescence in Carbon Dots Embedded in a Silica Gel Matrix. Phys. Chem. Chem. Phys. 2017, 19, 15137–15144. 10.1039/C7CP02731A. 28561114

[ref542] Carbonaro C. M.; Thakkar S. V.; Ludmerczki R.; Olla C.; Pinna A.; Loche D.; Malfatti L.; Cesare Marincola F.; Casula M. F. How Porosity Affects the Emission of Fluorescent Carbon Dot-Silica Porous Composites. Microporous Mesoporous Mater. 2020, 305, 110302 10.1016/j.micromeso.2020.110302.

[ref543] Xie Y.; Geng X.; Gao J.; Shi W.; Zhou Z.; Wang H.; Zhang D.; Deng B.; Yu R. Synthesis of Carbon Dots@Mg(OH)2 Solid-State Composites with Blue, Red Emitting for Horticultural Application. J. Alloys Compd. 2021, 873, 159663 10.1016/j.jallcom.2021.159663.

[ref544] Sen D.; Błoński P.; Otyepka M. Band-Edge Engineering at the Carbon Dot-TiO2 Interface by Substitutional Boron Doping. J. Phys. Chem. C 2019, 123, 5980–5988. 10.1021/acs.jpcc.8b11554.

[ref545] Amin M. A.; Khaled K. F.; Fadl-Allah S. A. Testing Validity of the Tafel Extrapolation Method for Monitoring Corrosion of Cold Rolled Steel in HCl Solutions - Experimental and Theoretical Studies. Corros. Sci. 2010, 52, 140–151. 10.1016/j.corsci.2009.08.055.

[ref546] Salestan S. K.; Seyedpour S. F.; Rahimpour A.; Shamsabadi A. A.; Tiraferri A.; Soroush M. Molecular Dynamics Insights into the Structural and Water Transport Properties of a Forward Osmosis Polyamide Thin-Film Nanocomposite Membrane Modified with Graphene Quantum Dots. Ind. Eng. Chem. Res. 2020, 59, 14447–14457. 10.1021/acs.iecr.0c00330.

[ref547] Ai L.; Yang Y.; Wang B.; Chang J.; Tang Z.; Yang B.; Lu S. Insights into Photoluminescence Mechanisms of Carbon Dots: Advances and Perspectives. Sci. Bull. 2021, 66, 839–856. 10.1016/j.scib.2020.12.015. 36654140

[ref548] Li S.; Li L.; Tu H.; Zhang H.; Silvester D. S.; Banks C. E.; Zou G.; Hou H.; Ji X. The Development of Carbon Dots: From the Perspective of Materials Chemistry. Mater. Today 2021, 51, 188–207. 10.1016/j.mattod.2021.07.028.

[ref549] Liu C.; Zhang F.; Hu J.; Gao W.; Zhang M. A Mini Review on PH-Sensitive Photoluminescence in Carbon Nanodots. Front. Chem. 2021, 8, 605028 10.3389/fchem.2020.605028. 33553104 PMC7862559

[ref550] Zhang H.; Liu J.; Wang B.; Liu K.; Chen G.; Yu X.; Li J.; Yu J. Zeolite-Confined Carbon Dots: Tuning Thermally Activated Delayed Fluorescence Emission via Energy Transfer. Mater. Chem. Front. 2020, 4, 1404–1410. 10.1039/C9QM00549H.

[ref551] Sun Y.; Liu J.; Pang X.; Zhang X.; Zhuang J.; Zhang H.; Hu C.; Zheng M.; Lei B.; Liu Y. Temperature-Responsive Conversion of Thermally Activated Delayed Fluorescence and Room-Temperature Phosphorescence of Carbon Dots in Silica. J. Mater. Chem. C 2020, 8, 5744–5751. 10.1039/D0TC00507J.

[ref552] Chen T. H.; Tseng W. L. Self-Assembly of Monodisperse Carbon Dots into High-Brightness Nanoaggregates for Cellular Uptake Imaging and Iron(III) Sensing. Anal. Chem. 2017, 89, 11348–11356. 10.1021/acs.analchem.7b02193. 28971671

[ref553] Butler K. T.; Davies D. W.; Cartwright H.; Isayev O.; Walsh A. Machine Learning for Molecular and Materials Science. Nature 2018, 559, 547–555. 10.1038/s41586-018-0337-2. 30046072

